# 5th International Symposium on Focused Ultrasound: North Bethesda, MD, USA. 28 August- 1 September 2016

**DOI:** 10.1186/s40349-016-0076-5

**Published:** 2016-11-21

**Authors:** Menashe Zaaroor, Alon Sinai, Dorit Goldsher, Ayelet Eran, Maria Nassar, Ilana Schlesinger, Jonathon Parker, Vinod Ravikumar, Pejman Ghanouni, Sherman Stein, Casey Halpern, Vibhor Krishna, Amelia Hargrove, Punit Agrawal, Barbara Changizi, Eric Bourekas, Michael Knopp, Ali Rezai, Brian Mead, Namho Kim, Panagiotis Mastorakos, Jung Soo Suk, Wilson Miller, Alexander Klibanov, Justin Hanes, Richard Price, Shutao Wang, Oluyemi Olumolade, Tara Kugelman, Vernice Jackson-Lewis, Maria Eleni (Marilena) Karakatsani, Yang Han, Serge Przedborski, Elisa Konofagou, Kullervo Hynynen, Isabelle Aubert, Gerhard Leinenga, Rebecca Nisbet, Robert Hatch, Anneke Van der Jeugd, Harrison Evans, Jürgen Götz, Jürgen Götz, Rebecca Nisbet, Ann Van der Jeugd, Harrison Evans, Gerhard Leinenga, Paul Fishman, Paul Yarowsky, Victor Frenkel, Shen Wei-Bin, Ben Nguyen, Carlos Sierra Sanchez, Camilo Acosta, Cherry Chen, Shih-Ying Wu, Maria Eleni (Marilena) Karakatsani, Elisa Konofagou, Muna Aryal, Iason T. Papademetriou, Yong-Zhi Zhang, Chanikarn Power, Nathan McDannold, Tyrone Porter, Zsofia Kovacs, Saejeong Kim, Neekita Jikaria, Farhan Qureshi, Michele Bresler, Joseph Frank, Henrik Odéen, George Chiou, John Snell, Nick Todd, Bruno Madore, Dennis Parker, Kim Butts Pauly, Mike Marx, Pejman Ghanouni, Sumeeth Jonathan, William Grissom, Costas Arvanitis, Nathan McDannold, Gregory Clement, Dennis Parker, Joshua de Bever, Henrik Odéen, Allison Payne, Douglas Christensen, Guillaume Maimbourg, Mathieu David Santin, Alexandre Houdouin, Stéphane Lehericy, Mickael Tanter, Jean Francois Aubry, Kim Butts Pauly, Christian Federau, Beat Werner, Casey Halpern, Pejman Ghanouni, Dong-Guk Paeng, Zhiyuan Xu, John Snell, Anders Quigg, Matt Eames, Changzhu Jin, Ashli Everstine, Jason Sheehan, M. Beatriz Lopes, Neal Kassell, John Snell, Anders Quigg, James Drake, Karl Price, Lior Lustgarten, Vivian Sin, Charles Mougenot, Elizabeth Donner, Emily Tam, Mojgan Hodaie, Adam Waspe, Thomas Looi, Samuel Pichardo, Wonhye Lee, Yong An Chung, Yujin Jung, In-Uk Song, Seung-Schik Yoo, Wonhye Lee, Hyun-Chul Kim, Yujin Jung, Yong An Chung, In-Uk Song, Jong-Hwan Lee, Seung-Schik Yoo, Charles Caskey, Wolf Zinke, Josh Cosman, Jillian Shuman, Jeffrey Schall, Christian Aurup, Shutao Wang, Hong Chen, Camilo Acosta, Elisa Konofagou, Hermes Kamimura, Antonio Carneiro, Nick Todd, Tao Sun, Yong-Zhi Zhang, Chanikarn Power, Navid Nazai, Sam Patz, Margaret Livingstone, Nathan McDannold, Todd Mainprize, Yuexi Huang, Ryan Alkins, Martin Chapman, James Perry, Nir Lipsman, Allison Bethune, Arjun Sahgal, Maureen Trudeau, Kullervo Hynynen, Hao-Li Liu, Po-Hung Hsu, Kuo-Chen Wei, Tao Sun, Chanikarn Power, Yong-Zhi Zhang, Jonathan Sutton, Phillip Alexander, Muna Aryal, Eric Miller, Nathan McDannold, Thiele Kobus, Yong-Zhi Zhang, Nathan McDannold, Alexandre Carpentier, Michael Canney, Alexandre Vignot, Kevin Beccaria, Delphine Leclercq, Cyril Lafon, Jean Yves Chapelon, Khe Hoang-Xuan, Jean-Yves Delattre, Ahmed Idbaih, Zhiyuan Xu, David Moore, Alexis Xu, Paul Schmitt, John Snell, Jessica Foley, Matt Eames, Jason Sheehan, Neal Kassell, Jonathan Sukovich, Charles Cain, Zhiyuan Xu, Aditya Pandey, John Snell, Neeraj Chaudhary, Sandra Camelo-Piragua, Steven Allen, Dong-Guk Paeng, Jon Cannata, Dejan Teofilovic, Jim Bertolina, Neal Kassell, Timothy Hall, Zhen Xu, Shih-Ying Wu, Maria Eleni (Marilena) Karakatsani, Julien Grondin, Carlos Sierra Sanchez, Vincent Ferrera, Elisa Konofagou, Gail ter Haar, Petros Mouratidis, Elizabeth Repasky, Kelsie Timbie, Lena Badr, Benjamin Campbell, John McMichael, Andrew Buckner, Jessica Prince, Aaron Stevens, Timothy Bullock, Richard Price, Karin Skalina, Chandan Guha, Franco Orsi, Guido Bonomo, Paolo Della Vigna, Giovanni Mauri, Gianluca Varano, George Schade, Yak-Nam Wang, Venu Pillarisetty, Joo Ha Hwang, Vera Khokhlova, Michael Bailey, Tatiana Khokhlova, Vera Khokhlova, Ilya Sinilshchikov, Petr Yuldashev, Yulia Andriyakhina, Wayne Kreider, Adam Maxwell, Tatiana Khokhlova, Oleg Sapozhnikov, Ari Partanen, Jonathan Lundt, Steven Allen, Jonathan Sukovich, Timothy Hall, Charles Cain, Zhen Xu, Tobias Preusser, Sabrina Haase, Mario Bezzi, Jürgen Jenne, Thomas Langø, Massimo Midiri, Michael Mueller, Giora Sat, Christine Tanner, Stephan Zangos, Matthias Guenther, Andreas Melzer, Arianna Menciassi, Selene Tognarelli, Andrea Cafarelli, Alessandro Diodato, Gastone Ciuti, Sven Rothluebbers, Julia Schwaab, Jan Strehlow, Senay Mihcin, Christine Tanner, Steffen Tretbar, Tobias Preusser, Matthias Guenther, Jürgen Jenne, Thomas Payen, Carmine Palermo, Steve Sastra, Hong Chen, Yang Han, Kenneth Olive, Elisa Konofagou, Matthew Adams, Vasant Salgaonkar, Serena Scott, Graham Sommer, Chris Diederich, Joan Vidal-Jove, Eloi Perich, Antonio Ruiz, Manuela Velat, David Melodelima, Aurelien Dupre, Jeremy Vincenot, Chen Yao, David Perol, Michel Rivoire, Samantha Tucci, Lisa Mahakian, Brett Fite, Elizabeth Ingham, Sarah Tam, Chang-il Hwang, David Tuveson, Katherine Ferrara, Stephen Scionti, Lili Chen, Dusica Cvetkovic, Xiaoming Chen, Roohi Gupta, Bin Wang, Charlie Ma, Kenneth Bader, Kevin Haworth, Adam Maxwell, Christy Holland, Narendra Sanghvi, Roy Carlson, Wohsing Chen, Christian Chaussy, Stefan Thueroff, Claudio Cesana, Carlo Bellorofonte, Qingguo Wang, Han Wang, Shengping Wang, Junhai Zhang, Alberto Bazzocchi, Alessandro Napoli, Robert Staruch, Chenchen Bing, Sumbul Shaikh, Joris Nofiele, Debra Szczepanski, Michelle Wodzak Staruch, Noelle Williams, Theodore Laetsch, Rajiv Chopra, Pejman Ghanouni, Jarrett Rosenberg, Rachelle Bitton, Alessandro Napoli, Suzanne LeBlang, Joshua Meyer, Mark Hurwitz, Kim Butts Pauly, Ari Partanen, Pavel Yarmolenko, Ari Partanen, Haydar Celik, Avinash Eranki, Viktoriya Beskin, Domiciano Santos, Janish Patel, Matthew Oetgen, AeRang Kim, Peter Kim, Karun Sharma, Alexander Chisholm, James Drake, Dionne Aleman, Adam Waspe, Thomas Looi, Samuel Pichardo, Alessandro Napoli, Alberto Bazzocchi, Roberto Scipione, Michael Temple, Adam Waspe, Joao Guilherme Amaral, Yuexi Huang, Ruby Endre, Maria Lamberti-Pasculli, Joost de Ruiter, Fiona Campbell, Jennifer Stimec, Samit Gupta, Manoj Singh, Charles Mougenot, Sevan Hopyan, Kullervo Hynynen, Gregory Czarnota, James Drake, David Brenin, Carrie Rochman, Roussanka Kovatcheva, Jordan Vlahov, Katja Zaletel, Julian Stoinov, Yang Han, Shutao Wang, Elisa Konofagou, Matthew Bucknor, Viola Rieke, Jenny Shim, Robert Staruch, Korgun Koral, Rajiv Chopra, Theodore Laetsch, Brian Lang, Carlos Wong, Heather Lam, Roussanka Kovatcheva, Jordan Vlahov, Katja Zaletel, Julian Stoinov, Alexander Shinkov, Jim Hu, Karun Sharma, Xi Zhang, Jonathan Macoskey, Kimberly Ives, Gabe Owens, Hitinder Gurm, Jiaqi Shi, Matthew Pizzuto, Charles Cain, Zhen Xu, Allison Payne, Christopher Dillon, Ivy Christofferson, Elaine Hilas, Jill Shea, Paul Greillier, Bénédicte Ankou, Francis Bessière, Ali Zorgani, Mathieu Pioche, Wojciech Kwiecinski, Julie Magat, Sandrine Melot-Dusseau, Romain Lacoste, Bruno Quesson, Mathieu Pernot, Stefan Catheline, Philippe Chevalier, Cyril Lafon, Fabrice Marquet, Pierre Bour, Fanny Vaillant, Sana Amraoui, Rémi Dubois, Philippe Ritter, Michel Haïssaguerre, Mélèze Hocini, Olivier Bernus, Bruno Quesson, Pamela Tebebi, Scott Burks, Saejeong Kim, Blerta Milo, Joseph Frank, Michael Gertner, Jimin Zhang, Andrew Wong, Brett Fite, Yu Liu, Azadeh Kheirolomoom, Jai Seo, Katherine Watson, Lisa Mahakian, Sarah Tam, Hua Zhang, Josquin Foiret, Alexander Borowsky, Katherine Ferrara, Doudou Xu, Andreas Melzer, Maya Thanou, Miguell Centelles, Mike Wright, Maral Amrahli, Po-Wah So, Wladyslaw Gedroyc, Miguell Centelles, Mike Wright, Wladyslaw Gedroyc, Maya Thanou, Esther Kneepkens, Edwin Heijman, Jochen Keupp, Steffen Weiss, Klaas Nicolay, Holger Grüll, Brett Fite, Andrew Wong, Yu Liu, Azadeh Kheirolomoom, Lisa Mahakian, Sarah Tam, Josquin Foiret, Katherine Ferrara, Scott Burks, Matthew Nagle, Saejeong Kim, Blerta Milo, Joseph Frank, Oleg Sapozhnikov, Anastasia V. Nikolaeva, Marina E. Terzi, Sergey A. Tsysar, Adam Maxwell, Bryan Cunitz, Michael Bailey, Pierre Mourad, Matthew Downs, Georgiana Yang, Qi Wang, Elisa Konofagou, Scott Burks, Matthew Nagle, Ben Nguyen, Michele Bresler, Saejeong Kim, Blerta Milo, Joseph Frank, Scott Burks, Matthew Nagle, Saejeong Kim, Blerta Milo, Joseph Frank, Johnny Chen, Justin Farry, Adam Dixon, Zhongmin Du, Ali Dhanaliwala, John Hossack, Alexander Klibanov, Ashish Ranjan, Danny Maples, Rajiv Chopra, Chenchen Bing, Robert Staruch, Rachel Wardlow, Michelle Wodzak Staruch, Jerry Malayer, Akhilesh Ramachandran, Joris Nofiele, Hirofumi Namba, Motohiro Kawasaki, Masashi Izumi, Katsuhito Kiyasu, Ryuichi Takemasa, Masahiko Ikeuchi, Takahiro Ushida, Calum Crake, Iason T. Papademetriou, Yong-Zhi Zhang, Tyrone Porter, Nathan McDannold, Satya V. V. N. Kothapalli, Wan Leighton, Zhaorui Wang, Ari Partanen, H. Michael Gach, William Straube, Michael Altman, Hong Chen, Young-sun Kim, Hyo Keun Lim, Hyunchul Rhim, Young-sun Kim, Hyo Keun Lim, Hyunchul Rhim, Johanna van Breugel, Manon Braat, Chrit Moonen, Maurice van den Bosch, Mario Ries, Cristina Marrocchio, Susan Dababou, Rachelle Bitton, Kim Butts Pauly, Pejman Ghanouni, Jae Young Lee, Jae Young Lee, Hyun Hoon Chung, Soo Yeon Kang, Kook Jin Kang, Keon Ho Son, Dandan Zhang, Matthew Adams, Vasant Salgaonkar, Juan Plata, Peter Jones, Aurea Pascal-Tenorio, Donna Bouley, Graham Sommer, Kim Butts Pauly, Chris Diederich, Aaron Bond, Robert Dallapiazza, Diane Huss, Amy Warren, Scott Sperling, Ryder Gwinn, Binit Shah, W. Jeff Elias, Colleen Curley, Ying Zhang, Karina Negron, Wilson Miller, Alexander Klibanov, Roger Abounader, Jung Soo Suk, Justin Hanes, Richard Price, Maria Eleni (Marilena) Karakatsani, Gesthimani Samiotaki, Shutao Wang, Tara Kugelman, Camilo Acosta, Elisa Konofagou, Zsofia Kovacs, Tsang-Wei Tu, Georgios Papadakis, Dima Hammoud, Joseph Frank, Matthew Silvestrini, Frank Wolfram, Daniel Güllmar, Juergen Reichenbach, Denis Hofmann, Joachim Böttcher, Harald Schubert, Thomas G. Lesser, Scott Almquist, Dennis Parker, Douglas Christensen, Francisco Camarena, Sergio Jiménez-Gambín, Noé Jiménez, Elisa Konofagou, Jin Woo Chang, Vandiver Chaplin, Rebekah Griesenauer, Michael Miga, Charles Caskey, Nicholas Ellens, Raag Airan, Alfredo Quinones-Hinojosa, Keyvan Farahani, Ari Partanen, Xue Feng, Samuel Fielden, Li Zhao, Wilson Miller, Max Wintermark, Kim Butts Pauly, Craig Meyer, Sijia Guo, Xin Lu, Jiachen Zhuo, Su Xu, Rao Gullapalli, Dheeraj Gandhi, Changzhu Jin, Omer Brokman, Matt Eames, John Snell, Dong-Guk Paeng, Hongchae Baek, Hyungmin Kim, Steven Leung, Taylor Webb, Kim Butts Pauly, Nathan McDannold, Yong-Zhi Zhang, Natalia Vykhodtseva, Thai-Son Nguyen, Jonathan Sukovich, Timothy Hall, Zhen Xu, Charles Cain, Chang Kyu Park, Sang Man Park, Na Young Jung, Min Soo Kim, Won Seok Chang, Hyun Ho Jung, Jin Woo Chang, Samuel Pichardo, Kullervo Hynynen, Michael Plaksin, Yoni Weissler, Shy Shoham, Eitan Kimmel, Anders Quigg, John Snell, Dong-Guk Paeng, Matt Eames, Oleg Sapozhnikov, Pavel B. Rosnitskiy, Vera Khokhlova, Shy Shoham, Steve Krupa, Eilon Hazan, Omer Naor, Yoav Levy, Noam Maimon, Inbar Brosh, Eitan Kimmel, Itamar Kahn, Jonathan Sukovich, Zhen Xu, Timothy Hall, Steven Allen, Charles Cain, Jessica Cahill, Tao Sun, Yong-Zhi Zhang, Chanikarn Power, Margaret Livingstone, Nathan McDannold, Nick Todd, Elodie Constanciel Colas, Adrian Wydra, Adam Waspe, Thomas Looi, Roman Maev, Samuel Pichardo, James Drake, Amirah Aly, Tao Sun, Yong-Zhi Zhang, Ozge Sesenoglu-Laird, Linas Padegimas, Mark Cooper, Nathan McDannold, Barbara Waszczak, Seruz Tehrani, Wilson Miller, Craig Slingluff, James Larner, Kumari Andarawewa, Matthew Bucknor, Eugene Ozhinsky, Rutwik Shah, Roland Krug, Viola Rieke, Roel Deckers, Sabine Linn, Britt Suelmann, Manon Braat, Arjen Witkamp, Paul Vaessen, Paul van Diest, Lambertus W. Bartels, Clemens Bos, Maurice van den Bosch, Nicolas Borys, Gert Storm, Elsken Van der Wall, Chrit Moonen, Navid Farr, Moez Alnazeer, Pavel Yarmolenko, Prateek Katti, Ari Partanen, Avinash Eranki, Peter Kim, Bradford Wood, Alexis Farrer, Scott Almquist, Christopher Dillon, Dennis Parker, Douglas Christensen, Allison Payne, Cyril Ferrer, Lambertus W. Bartels, Baudouin Denis de Senneville, Marijn van Stralen, Chrit Moonen, Clemens Bos, Yu Liu, Jingfei Liu, Brett Fite, Josquin Foiret, J. Kent Leach, Katherine Ferrara, Roohi Gupta, Dusica Cvetkovic, Charlie Ma, Lili Chen, Sabrina Haase, Stephan Zidowitz, Andreas Melzer, Tobias Preusser, Hsin-Lun Lee, Fang-Chi Hsu, Chia-Chun Kuo, Shiu-Chen Jeng, Tung-Ho Chen, Nai-Yi Yang, Jeng-Fong Chiou, Shiu-Chen Jeng, Yi-tzu Kao, Chia-Hsin Pan, Jing-Fu Wu, Tung-Ho Chen, Fang-Chi Hsu, Hsin-Lun Lee, Jeng-Fong Chiou, Fang-Chi Hsu, Yi-Chieh Tsai, Hsin-Lun Lee, Jeng-Fong Chiou, Sara Johnson, Dennis Parker, Allison Payne, Dawei Li, Ye He, Senay Mihcin, Ioannis Karakitsios, Jan Strehlow, Michael Schwenke, Sabrina Haase, Daniel Demedts, Yoav Levy, Tobias Preusser, Andreas Melzer, Senay Mihcin, Sven Rothluebbers, Ioannis Karakitsios, Xu Xiao, Jan Strehlow, Daniel Demedts, Ian Cavin, Giora Sat, Tobias Preusser, Andreas Melzer, Emilee Minalga, Allison Payne, Robb Merrill, Dennis Parker, Rock Hadley, Pascal Ramaekers, Mario Ries, Chrit Moonen, Martijn de Greef, Kian Shahriari, Mohammad Hossein Parvizi, Kiana Asadnia, Marzieh Chamanara, Seyed Kamran Kamrava, Hamid Reza Chabok, Michael Schwenke, Jan Strehlow, Daniel Demedts, Christine Tanner, Sven Rothluebbers, Tobias Preusser, Jan Strehlow, Ruben Stein, Daniel Demedts, Michael Schwenke, Sven Rothluebbers, Tobias Preusser, Daniel Demedts, Sabrina Haase, Sébastien Muller, Jan Strehlow, Thomas Langø, Tobias Preusser, Jeremy Tan, Cornel Zachiu, Pascal Ramaekers, Chrit Moonen, Mario Ries, Frank Wolfram, Daniel Güllmar, Harald Schubert, Thomas G. Lesser, Hans-Peter Erasmus, Elodie Constanciel Colas, Adam Waspe, Charles Mougenot, Thomas Looi, Glen Van Arsdell, Lee Benson, James Drake, Kee W. Jang, Tsang-Wei Tu, Neekita Jikaria, Matthew Nagle, Mary Angstadt, Bobbi Lewis, Farhan Qureshi, Scott Burks, Joseph Frank, Hailey McLean, Allison Payne, Martijn Hoogenboom, Dylan Eikelenboom, Martijn den Brok, Pieter Wesseling, Arend Heerschap, Jurgen Fütterer, Gosse Adema, Kevin Wang, Ying Zhang, Pei Zhong, Xu Xiao, Joyce Joy, Helen McLeod, Andreas Melzer, Chenchen Bing, Robert Staruch, Joris Nofiele, Debra Szczepanski, Michelle Wodzak Staruch, Theodore Laetsch, Rajiv Chopra, Chenchen Bing, Robert Staruch, Pavel Yarmolenko, Haydar Celik, Joris Nofiele, Debra Szczepanski, Peter Kim, Harry Kim, Matthew Lewis, Rajiv Chopra, Rutwik Shah, Eugene Ozhinsky, Viola Rieke, Matthew Bucknor, Chris Diederich, Vasant Salgaonkar, Peter Jones, Matthew Adams, Arda Ozilgen, Peter Zahos, Dezba Coughlin, Xinyan Tang, Jeff Lotz, Kathleen Jedruszczuk, Amitabh Gulati, Stephen Solomon, Elena Kaye, Samuel Fielden, John Mugler, Wilson Miller, Kim Butts Pauly, Craig Meyer, Gaetano Barbato, Gian Luca Scoarughi, Cristiano Corso, Alessandro Gorgone, Ilaria Giuseppina Migliore, Zachary Larrabee, Arik Hananel, Matt Eames, Jean-Francois Aubry, Avinash Eranki, Navid Farr, Ari Partanen, Karun Sharma, Pavel Yarmolenko, Bradford Wood, Peter Kim, Navid Farr, Satya V. V. N. Kothapalli, Avinash Eranki, Ayele Negussie, Emmanuel Wilson, Reza Seifabadi, Peter Kim, Hong Chen, Bradford Wood, Ari Partanen, Hyungwon Moon, Jeeun Kang, Changbeom Sim, Jin Ho Chang, Hyuncheol Kim, Hak Jong Lee, Noboru Sasaki, Mitsuyoshi Takiguchi, Lukas Sebeke, Xi Luo, Bram de Jager, Maurice Heemels, Edwin Heijman, Holger Grüll, Jan Strehlow, Michael Schwenke, Daniel Demedts, Tobias Preusser, Helen McLeod, Christopher Abraham, Samuel Pichardo, Laura Curiel, Pascal Ramaekers, Martijn de Greef, Rémi Berriet, Chrit Moonen, Mario Ries, Christopher Dillon, Margit Janát-Amsbury, Allison Payne, Joseph Corea, Patrick Peiyong Ye, Ana Clauda Arias, Kim Butts Pauly, Micheal Lustig, Bryant Svedin, Allison Payne, Dennis Parker

**Affiliations:** 1 Rambam Health Care Campus, Haifa, Israel; 2 Stanford University, Stanford, CA USA; 3 University of Pennsylvania, Philadelphia, PA USA; 4 The Ohio State University, Columbus, OH USA; 5 University of Virginia, Charlottesville, VA USA; 6 Center for Nanomedicine/Wilmer Eye Institute, Johns Hopkins University, Baltimore, MD USA; 7 Columbia University, New York, NY USA; 8 Sunnybrook Health Sciences Centre, Toronto, ON Canada; 9 Sunnybrook Research Institute, Toronto, ON Canada; 10 Clem Jones Centre for Ageing Dementia Research, Queensland Brain Institute, The University of Queensland, St Lucia, QLD Australia; 11 The University of Queensland, Brisbane (St Lucia Campus), QLD Australia; 12 University of Leuven, Leuven, Belgium; 13 University of Maryland School of Medicine/Baltimore VAMC, Baltimore, MD USA; 14 Research & Development Service, VA Maryland Healthcare System and University of Maryland School of Medicine, Baltimore, MD USA; 15 University of Maryland School of Medicine, Baltimore, MD USA; 16 Brigham and Women’s Hospital, Boston, MA USA; 17 Harvard Medical School, Boston, MA USA; 18 Boston University, Boston, MA USA; 19 National Institutes of Health, Bethesda, MD USA; 20 National Institutes of Health Clinical Center, Bethesda, MD USA; 21 University of Utah, Salt Lake City, UT USA; 22 Focused Ultrasound Foundation, Charlottesville, VA USA; 23 Vanderbilt University, Nashville, TN USA; 24 Georgia Institute of Technology, Atlanta, GA USA; 25 Cleveland Clinic, Cleveland, OH USA; 26 Institut Langevin Ondes et Images, ESPCI ParisTech, CNRS UMR 7587, INSERM U979, Paris, France; 27 CENIR, ICM, CNRS U7225, INSERM U975, Paris, France; 28 University Children’s Hospital Zurich, Zurich, Switzerland; 29 Jeju National University, Jeju, Republic of Korea; 30 Hospital for Sick Children, Toronto, ON Canada; 31 Centre for Image Guided Innovation and Therapeutic Intervention, Toronto, ON Canada; 32 Philips Healthcare Canada, Toronto, ON Canada; 33 Toronto Western Hospital, Toronto, ON Canada; 34 Thunder Bay Regional Research Institute, Thunder Bay, ON Canada; 35 Incheon St. Mary’s Hospital, The Catholic University of Korea, Incheon, Republic of Korea; 36 Department of Brain and Cognitive Engineering, Korea University, Seoul, Republic of Korea; 37 Washington University in St. Louis, St. Louis, MO USA; 38 Universidade de Sao Paolo, Sao Paulo, Brazil; 39 Physical Sciences Platform, Sunnybrook Research Institute, Toronto, ON Canada; 40 University of Toronto, Toronto, ON Canada; 41 Chang Gung University, Taoyuan, Taiwan; 42 Chang Gung Memorial Hospital, Taoyuan, Taiwan; 43 Tufts University, Medford, MA USA; 44 Radboud University Medical Center, Nijmegen, Netherlands; 45 Hopital de la Pitié-Salpêtrière, Assistance Publique Hopitaux de Paris, Paris, France; 46 CarThera, Denver, CO USA; 47 CarThera, Lyon, France; 48 INSERM Unit 1032, Lyon, France; 49 Institut du Cerveau et de la Moelle épinière, ICM, Paris, France; 50 University of Virginia Health System, Charlottesville, VA USA; 51 University of Michigan, Ann Arbor, MI USA; 52 HistoSonics, Inc., Ann Arbor, MI USA; 53 The Institute of Cancer Research, Sutton, Surrey, UK; 54 Roswell Park Cancer Institute, Buffalo, NY USA; 55 Albert Einstein College of Medicine, Bronx, NY USA; 56 Montefiore Medical Center, Bronx, NY USA; 57 Istituto Europeo di Oncologia, Milan, Italy; 58 University of Washington, Seattle, WA USA; 59 M.V. Lomonosov Moscow State University, Moscow, Russian Federation; 60 Philips, Bethesda, MD USA; 61 Fraunhofer MEVIS, Bremen, Germany; 62 Jacobs University Bremen, Bremen, Germany; 63 Universita Degli Studi Di Roma La Sapienza, Rome, Italy; 64 mediri GmbH, Heidelberg, Baden-Württemberg Germany; 65 Stiftelsen SINTEF, Trondheim, Norway; 66 University of Palermo, Palermo, Italy; 67 IBSmm Engineering spol. s r.o., Brno, Czech Republic; 68 GE Medical Systems Israel Ltd, Tirat Carmel, Israel; 69 ETH Zurich, Computer Vision Laboratory, Zurich, Switzerland; 70 Johann Wolfgang Goethe-Universität, Frankfurt, Germany; 71 University of Dundee, Dundee, UK; 72 Scuola Superiore Sant’Anna - The BioRobotics Institute, Pontedera, Italy; 73 University of Dundee - TRANS-FUSIMO, Dundee, Scotland UK; 74 Fraunhofer IBMT, St. Ingbert, Germany; 75 UCSF Thermal Therapy Research Group, San Francisco, CA USA; 76 University of California Sunnyvale, Sunnyvale, CA USA; 77 University of California San Francisco, San Francisco, CA USA; 78 Hospital University Mutua Terrassa, Barcelona, Spain; 79 Institut Khuab Barcelona, Barcelona, Spain; 80 LabTAU - INSERM U1032, Lyon, France; 81 Centre Leon Berard, Lyon, France; 82 University of California Davis, Davis, CA USA; 83 Cold Spring Harbor Laboratory, Cold Spring Harbor, NY USA; 84 Scionti Prostate Center, Sarasota, FL USA; 85 Fox Chase Cancer Center, Philadelphia, PA USA; 86 University of Cincinnati, Cincinnati, OH USA; 87 SonaCare Medical, LLC, Indianapolis, IN USA; 88 Ulthera, Inc., Mesa, AZ USA; 89 Caritas Hospital St. Josef, University of Regensburg, Strasslach-Dingharting, Germany; 90 Klinikum Muenchen Harlaching, Munich, Germany; 91 Clinica Columbus, Milan, Italy; 92 Shanghai First People’s Hospital, Shanghai, China; 93 Shanghai Cancer Center, Shanghai, China; 94 Shanghai Huashan Hospital, Shanghai, China; 95 The “Rizzoli” Orthopaedic Institute, Bologna, Italy; 96 Policlinico Umberto I, University of Rome – La Sapienza, Rome, Italy; 97 Philips Research, Dallas, TX USA; 98 University of Texas Southwestern Medical Center, Dallas, TX USA; 99 University of Oklahoma Health Sciences Center, Oklahoma City, OK USA; 100 University of Rome, Rome, Italy; 101 Jefferson University Hospitals, Philadelphia, PA USA; 102 Sheikh Zayed Institute for Pediatric Surgical Innovation, Children’s National Health System, Washington, DC USA; 103 Children’s National Health System, Washington, DC USA; 104 University of Toronto, Toronto, ON Canada; 105 Hospital for Sick Children, Toronto, ON Canada; 106 Centre for Image Guided Innovation and Therapeutic Intervention, Toronto, ON Canada; 107 Thunder Bay Regional Research Institute, Thunder Bay, ON Canada; 108 Policlinico Umberto I, University of Rome – La Sapienza, Rome, Italy; 109 The “Rizzoli” Orthopaedic Institute, Bologna, Italy; 110 University of Rome – La Sapienza, Rome, Italy; 111 Hospital for Sick Children, Toronto, ON Canada; 112 Sunnybrook Health Sciences Center, Toronto, ON Canada; 113 Philips Healthcare Canada, Toronto, ON Canada; 114 University of Virginia, Charlottesville, VA USA; 115 University Hospital of Endocrinology, Sofia, Bulgaria; 116 University Medical Centre Ljubljana, Ljubljana, Slovenia; 117 Columbia University, New York, NY USA; 118 University of California San Francisco, San Francisco, CA USA; 119 Children’s Health, Decatur, GA USA; 120 Philips Research, Dallas, TX USA; 121 University of Texas Southwestern Medical Center, Dallas, TX USA; 122 University of Hong Kong, Hong Kong, Hong Kong SAR; 123 Theraclion Asia Pacific Limited, Hong Kong, Hong Kong SAR; 124 University Medical Centre Ljubljana, Ljubljana, Slovenia; 125 University Hospital of Endocrinology, Sofia, Bulgaria; 126 Medical University Sofia, Clinical Center of Endocrinology, Sofia, Bulgaria; 127 Weill Cornell Medicine, New York, NY USA; 128 Children’s National Health System, Washington, DC USA; 129 University of Michigan, Ann Arbor, MI USA; 130 University of Utah, Salt Lake City, UT USA; 131 INSERM / Université de Lyon, Lyon, France; 132 Hospices Civils de Lyon, Lyon, France; 133 Institut Langevin Ondes et Images, ESPCI ParisTech, CNRS UMR 7587, INSERM U979, Paris, France; 134 IHU Liryc, Electrophysiology and Heart Modeling Institute, Pessac-Bordeaux, France; 135 Station de Primatologie-UPS846-CNRS, Rousset sur Arc, France; 136 University of Virginia, Charlottesville, Virginia, United States and INSERM Unit 1032, Lyon, France; 137 IHU Liryc, Electrophysiology and Heart Modeling Institute, Pessac-Bordeaux, France; 138 National Institutes of Health, Bethesda, MD USA; 139 National Institutes of Health Clinical Center, Bethesda, MD USA; 140 Kona Medical, Inc., Bellevue, WA USA; 141 University of California Davis, Davis, CA USA; 142 Chongqing Changlin Science & Technology Ltd., Chongqing, China; 143 University of Dundee, Dundee, UK; 144 King’s College London, London, UK; 145 Imperial College Healthcare London, London, UK; 146 King’s College London, London, UK; 147 Imperial College Healthcare London, London, UK; 148 Eindhoven University of Technology, Eindhoven, Netherlands; 149 Philips Research, Eindhoven, Netherlands; 150 Philips Research, Hamburg, Germany; 151 University of California Davis, Davis, CA USA; 152 National Institutes of Health Clinical Center, Bethesda, MD USA; 153 University of Washington, Seattle, WA USA; 154 Moscow State University, Moscow, Russian Federation; 155 University of Washington, Seattle, WA USA; 156 Columbia University, New York, NY USA; 157 National Institutes of Health Clinical Center, Bethesda, MD USA; 158 National Institutes of Health Clinical Center, Bethesda, MD USA; 159 National Institutes of Health, Bethesda, MD USA; 160 University of Virginia, Charlottesville, VA USA; 161 Oklahoma State University, Stillwater, OK USA; 162 University of Texas Southwestern Medical Center, Dallas, TX USA; 163 Philips Research, Dallas, TX USA; 164 University of Oklahoma Health Sciences Center, Oklahoma City, OK USA; 165 Kochi University, Kochi Medical School, Nankoku, Kochi Japan; 166 Multidisciplinary Pain Center, School of Medicine, Aichi Medical University, Nagakute, Aichi Japan; 167 Brigham and Women’s Hospital, Boston, MA USA; 168 Harvard Medical School, Boston, MA USA; 169 Boston University, Boston, MA USA; 170 Washington University in Saint Louis, Saint Louis, MO USA; 171 Philips, Bethesda, MD USA; 172 Samsung Medical Center, Seoul, Republic of Korea; 173 Samsung Medical Center, Seoul, Republic of Korea; 174 University Medical Center – Utrecht, Utrecht, Netherlands; 175 University of Rome – La Sapienza, Rome, Italy; 176 Stanford University, Stanford, CA USA; 177 Seoul National University Hospital, Seoul, Republic of Korea; 178 Alpinion Medical Systems, Seoul, Republic of Korea; 179 The First Affiliated Hospital of Harbin Medical University, Harbin, China; 180 University of California San Francisco, San Francisco, CA USA; 181 Stanford University, Stanford, CA USA; 182 University of Virginia, Charlottesville, VA USA; 183 Swedish Neuroscience Institute, Seattle, WA USA; 184 University of Virginia, Charlottesville, VA USA; 185 Johns Hopkins University, Baltimore, MD USA; 186 Center for Nanomedicine/Wilmer Eye Institute, Johns Hopkins University, Baltimore, MD USA; 187 Columbia University, New York, NY USA; 188 National Institutes of Health Clinical Center, Bethesda, MD USA; 189 University of California Davis, Davis, CA USA; 190 SRH Wald-Klinikum Gera / Clinic of Thoracic Surgery, Gera, Germany; 191 Institute of Diagnostic and Interventional Radiology, University Hospital - Friedrich Schiller University, Jena, Germany; 192 SRH Wald-Klinikum Gera, Gera, Germany; 193 Institute of Animal Experimentation, Friedrich Schiller University, Jena, Germany; 194 University of Utah, Salt Lake City, UT USA; 195 Universitat Politècnica de València, Gandia, Spain; 196 LUNAM Universite. Université du Maine, Le Mans, Spain; 197 Columbia University, New York, NY USA; 198 YUMC Severance Hospital, Seoul, Republic of Korea; 199 Vanderbilt University Institute of Imaging Science, Nashville, TN USA; 200 Vanderbilt University, Nashville, TN USA; 201 Johns Hopkins University, Baltimore, MD USA; 202 Stanford University, Stanford, CA USA; 203 National Cancer Institute, Bethesda, MD USA; 204 Philips, Bethesda, MD USA; 205 University of Virginia, Charlottesville, VA USA; 206 Stanford University, Stanford, CA USA; 207 University of Maryland Baltimore, Baltimore, MD USA; 208 Focused Ultrasound Foundation, Charlottesville, VA USA; 209 InSightec Ltd, Tirat Carmel, Israel; 210 Korea Institute of Science and Technology, Seoul, Republic of Korea; 211 Stanford University, Stanford, CA USA; 212 Brigham and Women’s Hospital, Harvard Medical School, Boston, MA USA; 213 Harvard Medical School, Boston, MA USA; 214 University of Michigan, Ann Arbor, MI USA; 215 Severance Hospital, Yonsei University, Seoul, Republic of Korea; 216 YUMC Severance Hospital, Seoul, Republic of Korea; 217 Thunder Bay Regional Research Institute, Thunder Bay, ON Canada; 218 Sunnybrook Health Sciences Centre, Toronto, ON Canada; 219 Technion – Israel Institute of Technology, Haifa, Israel; 220 Focused Ultrasound Foundation, Charlottesville, VA USA; 221 University of Virginia, Charlottesville, VA USA; 222 Jeju National University, Jeju, Republic of Korea; 223 University of Washington, Seattle, WA USA; 224 Moscow State University, Moscow, Russian Federation; 225 InSightec Ltd, Tirat Carmel, Israel; 226 Technion - Israel Institute of Technology, Haifa, Israel; 227 University of Michigan, Ann Arbor, MI USA; 228 Georgia Institute of Technology, Atlanta, GA USA; 229 Brigham and Women’s Hospital, Harvard Medical School, Boston, MA USA; 230 Harvard Medical School, Boston, MA USA; 231 Brigham and Women’s Hospital, Boston, MA USA; 232 Centre for Image Guided Innovation and Therapeutic Intervention, Toronto, ON Canada; 233 The Institute for Diagnostic Imaging Research, Windsor, ON Canada; 234 Hospital for Sick Children, Toronto, ON Canada; 235 Thunder Bay Regional Research Institute, Thunder Bay, ON Canada; 236 Northeastern University, Boston, MA USA; 237 Brigham and Women’s Hospital, Harvard Medical School, Boston, MA USA; 238 Harvard Medical School, Boston, MA USA; 239 Coprenicus Therapeutics, Inc., Cleveland, OH USA; 240 University of Virginia, Charlottesville, VA USA; 241 University of California San Francisco, San Francisco, CA USA; 242 University Medical Center – Utrecht, Utrecht, Netherlands; 243 Celsion Corporation, Lawrenceville, NJ USA; 244 Utrecht University, Utrecht, Netherlands; 245 National Institutes of Health, Bethesda, MD USA; 246 The Sheikh Zayed Institute for Pediatric Surgical Innovation, Washington, DC USA; 247 Philips, Bethesda, MD USA; 248 Children’s National Health System, Washington, DC USA; 249 University of Utah, Salt Lake City, UT USA; 250 University Medical Center – Utrecht, Utrecht, Netherlands; 251 University of California Davis, Davis, CA USA; 252 Fox Chase Cancer Center, Philadelphia, PA USA; 253 Fraunhofer MEVIS, Bremen, Germany; 254 University of Dundee, Dundee, UK; 255 Jacobs University Bremen, Bremen, Germany; 256 Taipei Medical University Healthcare System, Taipei, Taiwan; 257 Taipei Medical University Healthcare System, Taipei, Taiwan; 258 Taipei Medical University Healthcare System, Taipei, Taiwan; 259 University of Utah, Salt Lake City, UT USA; 260 Shanghai A&S Science Technology Development Co., LTD., Shanghai, China; 261 IMSAT, Dundee, UK; 262 University of Dundee, Dundee, UK; 263 Fraunhofer MEVIS, Bremen, Germany; 264 InSightec Ltd, Tirat Carmel, Israel; 265 Jacobs University Bremen, Bremen, Germany; 266 IMSAT, Dundee, UK; 267 Fraunhofer MEVIS, Bremen, Germany; 268 University of Dundee, Dundee, UK; 269 University of Dundee - FUTURA, Dundee, UK; 270 NHS, Dundee, UK; 271 GE Medical Systems Israel Ltd., Tirat Carmel, Israel; 272 Jacobs University Bremen, Bremen, Germany; 273 University of Utah, Salt Lake City, UT USA; 274 University Medical Center – Utrecht, Utrecht, Netherlands; 275 Islamic Azad University, Science and Research Branch, Tehran, Iran; 276 Iran University of Medical Sciences, Tehran, Iran; 277 University of Southern California Los Angeles, Los Angeles, CA USA; 278 Fraunhofer MEVIS, Bremen, Germany; 279 ETH Zurich, Computer Vision Laboratory, Zurich, Switzerland; 280 Jacobs University Bremen, Bremen, Germany; 281 Fraunhofer MEVIS, Bremen, Germany; 282 Jacobs University Bremen, Bremen, Germany; 283 Fraunhofer MEVIS, Bremen, Germany; 284 Stiftelsen SINTEF, Trondheim, Norway; 285 Jacobs University Bremen, Bremen, Germany; 287 University Medical Center – Utrecht, Utrecht, Netherlands; 288 SRH Wald-Klinikum Gera, Gera, Germany; 289 Institute of Diagnostic and Interventional Radiology, Friedrich Schiller University, Jena, Germany; 290 Institute of Animal Experimentation, Friedrich Schiller University, Jena, Germany; 291 University of Rome – La Sapienza, Rome, Italy; 292 Centre for Image Guided Innovation and Therapeutic Intervention, Toronto, ON Canada; 293 Hospital for Sick Children, Toronto, ON Canada; 294 Philips Healthcare Canada, Toronto, ON Canada; 295 National Institutes of Health Clinical Center, Bethesda, MD USA; 296 National Institutes of Health, Bethesda, MD USA; 297 University of Utah, Salt Lake City, UT USA; 298 Radboud University Medical Center, Nijmegen, Netherlands; 299 Virginia Tech, Blacksburg, VA USA; 300 Duke University, Durham, NC USA; 301 University of Dundee - FUTURA, Dundee, UK; 302 University of Dundee, Dundee, UK; 303 University of Texas Southwestern Medical Center, Dallas, TX USA; 304 Philips Research, Dallas, TX USA; 305 University of Oklahoma Health Sciences Center, Oklahoma City, OK USA; 306 University of Texas Southwestern Medical Center, Dallas, TX USA; 307 Philips Research, Dallas, TX USA; 308 Sheikh Zayed Institute for Pediatric Surgical Innovation, Washington, DC USA; 309 Children’s National Health System, Washington, DC USA; 310 Texas Scottish Rite Hospital for Children, Dallas, TX USA; 311 University of California San Francisco, San Francisco, CA USA; 312 University of California San Francisco, Sunnyvale, CA USA; 313 University of California San Francisco, Napa, CA USA; 314 Memorial Sloan-Kettering Cancer Center, New York, NY USA; 315 University of Virginia, Charlottesville, VA USA; 316 Stanford University, Stanford, CA USA; 317 Promedica Bioelectronics SRL, Rome, Italy; 318 FUS Mobile Inc., Alpharetta, GA USA; 319 Children’s National Health System, Washington, DC USA; 320 National Institutes of Health, Bethesda, MD USA; 321 Philips, Bethesda, MD USA; 322 The Sheikh Zayed Institute for Pediatric Surgical Innovation, Washington, DC USA; 323 National Institutes of Health, Bethesda, MD USA; 324 Washington University in Saint Louis, Saint Louis, MO USA; 325 Children’s National Health System, Washington, DC USA; 326 Philips, Bethesda, MD USA; 327 Seoul National University Bundang Hospital, Seongnam, Republic of Korea; 328 Sogang University, Seoul, Republic of Korea; 329 Hokkaido University, Sapporo, Hokkaido Japan; 330 Uniklinik Koeln, Cologne, Germany; 331 Eindhoven University of Technology, Eindhoven, Netherlands; 332 Philips Research, Eindhoven, Netherlands; 333 Fraunhofer MEVIS, Bremen, Germany; 334 Jacobs University Bremen, Bremen, Germany; 335 Univerisity of Dundee, Dundee, UK; 336 Thunder Bay Regional Research Institute, Thunder Bay, ON Canada; 337 University Medical Center – Utrecht, Utrecht, Netherlands; 338 IMASONIC SAS, Voray sur l’Ognon, France; 339 University of Utah, Salt Lake City, UT USA; 340 University of California Berkeley, Berkeley, CA USA; 341 Stanford University, Stanford, CA USA; 342 University of Utah, Salt Lake City, UT USA

## A1 Treatment of essential tremor and Parkinson’s disease tremor by MRI guided Focused Ultrasound: a report of 38 consecutive cases in a single center

### Menashe Zaaroor, Alon Sinai, Dorit Goldsher, Ayelet Eran, Maria Nassar, Ilana Schlesinger

#### Rambam Health Care Campus, Haifa, Israel

**Objectives**

Thalamotomy of the ventral intermediate nucleus (VIM) is effective in alleviating medication resistant tremor in patients with essential tremor (ET) and Parkinson’s disease (PD). MRI guided Focused Ultrasound (MRgFUS) is an innovative technology that enables non-invasive thalamotomy via thermal ablation.

**Methods**

Thirty eight ET and PD patients with severe medication resistant tremor underwent MRgFUS underwent unilateral VIM thalamotomy using MRgFUS. Effect was evaluated using clinical Rating Scale of Tremor (CRST) in ET patients and Unified PD Rating Scale motor part (UPDRS) in PD patients. Quality of life was assessed by Quality of life in ET Questionnaire (QUEST) and PD Questionaire (PDQ-39).

**Results**

Tremor stopped in the treated hand in 37 patients immediately following the treatment. In one patients tremor was modified but not abolished. At one month post-treatment, the ET patients’ CRST score decreased from 38.6 ± 12.0 to 9.3 ± 7.7 (p < 0.001) and QUEST scores decreased from 44.8 ± 17.8 to 13.1 ± 15.9 (p < 0.001). In PD patients UPDRS-motor part decreased from 26.2 ± 8.7 to 16.3 ± 11.0 (p = 0.0087) and PDQ39 decreased from 40.8 ± 18.2 to 26.5 ± 15.1 (p = 0.027). During follow up of 1-24 months (mean 10.9 ± 8.1 months) tremor reappeared in seven of the patients, but in all but three, to a lesser degree than before the procedure.

Adverse events that transiently occurred during sonication included: Headache (n = 11), short lasting vertigo (n = 17) and dizziness (n = 4), nausea (n = 4), burning scalp sensation (n = 3), vomiting (n = 3) and lip paresthesia (n = 2). Adverse events that lasted after the procedure included gait ataxia (n = 5), unsteady feeling when walking (n = 4,) unilateral taste disturbances (n = 3) and hand ataxia (n = 3). All adverse events were transient and none lasted beyond 3 months.

**Conclusions**

MRgFUS VIM thalamotomy to relieve medication resistant tremor was safe and effective in ET, and PD. Current results emphasize its low adverse events profile and high efficacy in treating tremor. Large randomized studies are needed to assess prolonged efficacy and safety.

## A2 Focused Ultrasound likely dominates deep brain stimulation and stereotactic radiosurgery for medically-refractory essential tremor: an initial decision and cost-effectiveness analysis

### Jonathon Parker^1^, Vinod Ravikumar^1^, Pejman Ghanouni^1^, Sherman Stein^2^, Casey Halpern^1^

#### ^1^Stanford University, Stanford, California, USA; ^2^University of Pennsylvania, Philadelphia, Pennsylvania, USA

**Objectives**

Essential Tremor (ET) is one of the most common neurologic conditions, and conservative measures are frequently suboptimal. Recent data from a multi-institution, randomized controlled clinical trial demonstrated that Magnetic Resonance-guided Focused Ultrasound (MRgFUS) thalamotomy improves upper limb tremor in medically refractory ET. This study assesses the cost-effectiveness of this novel therapy in comparison to existing procedural options.

**Methods**

PubMed and Cochrane Library searches were performed for studies of MRgFUS, Deep Brain Stimulation (DBS), and Stereotactic Radiosurgery (SRS) for ET. Pre- and post-operative tremor-related disability scores were collected from 32 studies involving 83 MRgFUS, 615 DBS, and 260 SRS cases. Utility (defined as percent change in functional disability) was calculated, and Medicare reimbursements were collected as a proxy for societal cost – costs of MRgFUS for ET were derived from a combination of available costs of approved indications and SRS costs where appropriate. A decision and cost-effectiveness analysis was then constructed, implementing meta-analytic techniques.

**Results**

MRgFUS thalamotomy resulted in significantly higher utility scores compared with DBS and SRS based on estimates of Medicare reimbursement (p < 0.001). MRgFUS was also the most inexpensive procedure out of the three (p < 0.001).

**Conclusions**

Preliminary experience with MRgFUS for ET suggests that this novel therapeutic may be more effective than available alternatives and potentially less costly for society. It thus will likely “dominate” DBS and SRS as a more cost-effective option for medically refractory ET. Our findings support further investigation of MRgFUS for ET and broad adoption.

## A3 Tractography-based VIM identification for Focused Ultrasound thalamotomy: initial results

### Vibhor Krishna, Amelia Hargrove, Punit Agrawal, Barbara Changizi, Eric Bourekas, Michael Knopp, Ali Rezai

#### The Ohio State University, Columbus, Ohio, USA

**Objectives**

The ventral intermediate nucleus (VIM) is not visible on conventional Magnetic Resonance Imaging (MRI). A novel method for tractography-based VIM identification has recently been described. We report the short-term clinical results of prospective VIM targeting with tractography in a cohort of patients undergoing Focused Ultrasound thalamotomy.

**Methods**

**Fig. 1 (abstract A3).**
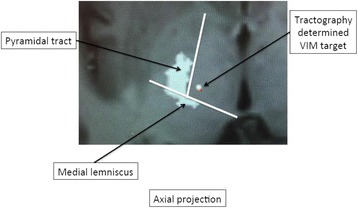
Axial T1 projection showing the relation of VIM target 3 mm medial and anterior to pyramidal tract and medial lemniscus respectively

**Fig. 2 (abstract A3).**
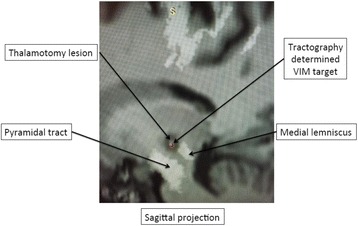
Postoperative sagittal T1 projection demonstrating the relationship between pyramidal tract and medial lemniscus in relation to thalamotomy lesion

**Fig. 3 (abstract A3).**
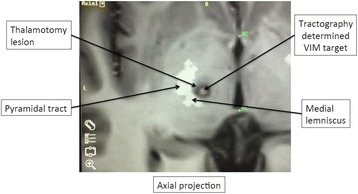
Postoperative axial T1 projection demonstrating the relationship between pyramidal tract and medial lemniscus in relation to thalamotomy lesion

All patients underwent structural and diffusion weighted imaging (60 diffusion directions, 2 mm isovoxel) with 3 Tesla MRI scanner (Philips Ingenia CX). The images were processed using streamline tractography (Stealth Viz, Medtronic Inc.). The lateral and posterior borders of VIM were defined by tracking the pyramidal tract and medial lemniscus respectively. A VIM region of interest (ROI) was placed 3 mm away from these borders (Figs. 1, 2 and 3). The structural connectivity of this VIM ROI was confirmed to the motor cortex (M1) and cerebellum. The coordinates of tractography-based VIM in relation to posterior commissure were noted for surgical targeting. The parameters analyzed include a clinical tremor scale (pre-, intraoperative, and post operative), operative time, and number of sonications.

**Results**

Tractography-based VIM targeting was successful in 7 out of 8 patients. The coordinates of tractography-based VIM were significantly different from the standard coordinates (3-D distance 3.9 ± 2.4 mm). Therapeutic sonication (>55 °C temperature, 10 seconds) at the tractography target resulted in >50 % tremor improvement with intraoperative objective tremor assessment without any motor or sensory side-effects. The mean operative time was 78 ± 3.3 minutes with 12.8 ± 3.9 average sonications. Overall the tremor scores significantly improved one month after surgery (preop CRST total 62.1 ± 15.5 *versus* 30.3 ± 14.1, two tailed t-test p = 0.006). None of the patients experienced sensory deficits or motor weakness during follow-up.

**Conclusions**

We report that prospective tractography-based VIM targeting is safe and feasible. The short-term clinical results are satisfactory. Long-term tremor efficacy outcomes are desirable to further assess the usefulness of this technique.

## A4 Targeted delivery of brain-penetrating non-viral GDNF gene vectors to the striatum with MRI-guided Focused Ultrasound reverses neurodegeneration in a Parkinson’s disease model

### Brian Mead^1^, Namho Kim^2^, Panagiotis Mastorakos^2^, Jung Soo Suk^2^, Wilson Miller^1^, Alexander Klibanov^1^, Justin Hanes^2^, Richard Price^1^

#### ^1^University of Virginia, Charlottesville, Virginia, USA; ^2^Center for Nanomedicine/Wilmer Eye Institute, Johns Hopkins University, Baltimore, Maryland, USA

**Objectives**

Parkinson’s disease (PD) is characterized by the degeneration of dopaminergic neurons in the motor control pathways of the brain. Gene therapy using glial cell derived neurotrophic factor (GDNF) has shown some limited promise for treating PD; however, we hypothesize that outcomes could be further improved by enhancing gene vector distribution. We previously developed a gene therapy approach that entails delivering systemically administered non-viral gene-bearing nanoparticles (BPN) across the Blood-Brain Barrier with MRI-guided Focused Ultrasound (FUS). BPN rapidly penetrate brain tissue due to a dense coat of polyethylene glycol, and this approach mediates efficient and localized transgene expression in the brain of healthy rats. Here, we tested whether the FUS-mediated delivery of GDNF plasmid-bearing BPN (GDNF-BPN) reverses neurodegeneration in the rat 6-OHDA PD model.

**Methods**

6-OHDA rats were ultrasonically coupled to a 1.15 MHz MRI-compatible FUS transducer. T2 and T2* pre-treatment scans were obtained to allow FUS targeting of striatum. Microbubbles (2x105/g) and 100 μg of ~50 nm non-viral GDNF plasmid-bearing BPN (polyethylene glycol/polyethylenimine) were co-injected i.v. and FUS was applied at 0.6 MPa, with a 0.5 % duty cycle, for 2 min. Contrast T1 and T2* images allowed semi-real time confirmation of BBB disruption and safety, respectively. Efficacy was assessed using an ELISA for GDNF, tyrosine hydroxylase (TH) and VMAT2 immunolabeling for neural degeneration, HPLC for dopamine, and behavioral analysis (i.e. apomorphine-induced rotational asymmetry and forepaw use bias in 6-OHDA rats).

**Results**

**Fig. 4 (abstract A4).**
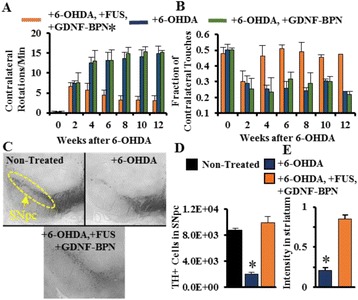
Graphs of rotational bias (**a**) or forepaw use bias (**b**) following 6-OHDA injection. (**c**) Representative images of TH-immunlolabeled sections through the SNpc. Graphs represent TH+ cell number in SNpc (**d**) and staining intensity in the striatum (**e**). * p < 0.05

Striatum-targeted delivery of GDNF plasmid-bearing BPN with FUS led to an ~80 % reduction in apomorphine-induced rotational asymmetry, eliminated forepaw use bias (Fig. 4a,b), and fully restored TH+ dopaminergic neuron density in both the substantia nigra pars compacta (SNpc) and striatum compared to untreated 6-OHDA rats (Fig. 4c,d). T2* MRI confirmed safety of the BBB opening approach.

**Conclusions**

FUS-mediated delivery of systemically circulating non-viral GDNF-BPN to the striatum of 6-OHDA rats confers a significant behavioral benefit as well as a restoration of TH+ cell number in the nigrostriatal pathway, indicating cessation and/or reversal of neurodegeneration. Our studies indicate that delivery of GDNF-BPN with FUS may provide a powerful, non-invasive and highly tailorable gene therapy approach to slow or stop the neurodegenerative process in PD.

## A5 Focused ultrasound facilitated gene delivery for neuro-restoration in Parkinson’s disease mice

### Shutao Wang, Oluyemi Olumolade, Tara Kugelman, Vernice Jackson-Lewis, Maria Eleni (Marilena) Karakatsani, Yang Han, Serge Przedborski, Elisa Konofagou

#### Columbia University, New York, New York, USA

**Objectives**

Not released for publication

**Methods**

Not released for publication

**Results**

Not released for publication

**Conclusions**

Not released for publication

## A6 MRI-g-FUS for the treatment of Alzheimer’s disease

### Kullervo Hynynen^1^, Isabelle Aubert^2^

#### ^1^Sunnybrook Health Sciences Centre, Toronto, Ontario, Canada; ^2^Sunnybrook Research Institute, Toronto, Ontario, Canada

**Objectives**

Over the past ten years consistent effort has been put forward at the University of Toronto to develop Focused Ultrasound methods for the treatment of AD. This talk will review the progress made so far.

**Methods**

The first studies demonstrated safe antibody delivery in AD mouse model with significant reduction in the plaque load. A follow up studies with two-photon microscopy showed that blood vessels with plague deposits showed a different type of opening than vessels in normal brain but large molecule delivery into the brain was still possible in these animals. Another study demonstrated that plaque reduction can be achieved by just opening the BBB with microbubbles.

**Results**

The histology revealed stimulation of neurogenesis. Multiple treatments of old mice resulted in memory rescue without any observable side-effects. A follow up study demonstrated that this neurogenesis was not induced with exposures that did not cause observable BBB opening even with the presence of the microbubbles. An ongoing study in large animals has shown that half-brain BBB opening can be safely and repeatable performed indicating the feasibility of clinical translation.

## A7 Scanning Focused Ultrasound disruption of the blood-brain barrier as an Alzheimer’s disease therapy

### Gerhard Leinenga^1^, Rebecca Nisbet^2^, Robert Hatch^1^, Anneke Van der Jeugd^3^, Harrison Evans^2^, Jürgen Götz^2^

#### ^1^Clem Jones Centre for Ageing Dementia Research, Queensland Brain Institute, The University of Queensland, St Lucia, Queensland, Australia; ^2^The University of Queensland, Brisbane (St Lucia Campus), QLD, Australia; ^3^University of Leuven, Leuven, Belgium, Belgium

**Objectives**

Alzheimer’s disease is the most common form of dementia. Pathological abnormalities in the Alzheimer’s disease brain includes the presence of amyloid-beta plaques, hyperphosphorylation and intracellular aggregation of tau and synaptic degeneration. Focused ultrasound combined with intravenous injection of microbubbles has been shown to reversibly open the blood-brain-barrier (BBB). By moving the focus in scanning mode we are able to open the BBB throughout the brain of a mouse. Here we tested the effects of repeated scanning ultrasound (SUS) in APP23 amyloid plaque-bearing mice, pR5 tau mice and wild-type mice to determine the effects of SUS on amyloid, tau and dendritic spines.

**Methods**

The device used was the Therapy Imaging Probe System (TIPS, Philips Research), which has an eight-element annular array transducer with a focal length of 80 mm a radius of curvature of 80 mm, a 33 mm central opening, and a motorized 3D positioning system. The focus 6 dB size was 1.5 mm x 1.5 mm x 12 mm at 1 MHz. Settings that were applied were 1 MHz centre frequency, 0.7 MPa peak rarefactional pressure applied outside the skull, 10 Hz pulse repetition frequency, 10 ms pulse length and 10 % duty cycle immediately after retroorbital injection of in-house made microbubbles. APP23 mice that accumulate amyloid beta, pR5 mice that overexpress FTD-mutant tau, and wild-type C57Bl/6 mice were treated weekly by scanning ultrasound (SUS) for periods of 4 to 7 weeks.

**Results**

In APP23 mice we used repeated scanning ultrasound (SUS) treatments of the mouse brain to remove amyloid-beta. Spinning disk confocal microscopy revealed extensive internalization of Abeta into the lysosomes of activated microglia in mouse brains subjected to SUS. Plaque burden was reduced in SUS-treated AD mice compared to sham-treated animals. Treated AD mice also displayed improved performance on three memory tasks.

In PR5 mice we investigated the efficacy of a novel tau isoform-specific single chain antibody fragment, RNX, delivered by passive immunization in the P301L human tau transgenic pR5 mouse model. When administration of RNX was combined with scanning ultrasound (SUS), RNX delivery into the brain and uptake by neurons were markedly increased, as were reductions in tau phosphorylation and anxiety-like behavior.

In wild-type mice we investigated the effects of SUS on neuronal excitability and morphology. We performed patch-clamp recordings from hippocampal CA1 pyramidal neurons in wild-type mice 2 and 24 hours after a single SUS treatment, and one-week and three months after six weekly SUS treatments. No change in CA1 neuronal excitability was observed compared to sham-treated neurons at any time-point. Multiple SUS treatments had the effect of preventing the loss of CA1 synapses that occurred in sham-treated neurons.

**Conclusions**

We show that scanning Focused Ultrasound disruption of the BBB has multiple biological effects in the brain which make it an attractive candidate for an Alzheimer disease therapy. SUS reduced plaque burden and amyloid-beta levels in APP23 amyloid mice, through activation of microglia, and improved performance on tests of memory function. In pR5 tau mice SUS alone reduced hyperphosphorylation of tau, and enhanced the delivery of anti-tau antibodies resulting in improved reductions in pathology and behavioral abnormalities. In wild-type mice SUS was shown to have no effect on the firing of hippocampal neurons or their morphology, but prevented spine loss at 3 months after six weekly SUS treatments. If these effects on Abeta and tau pathology, and dendritic morphology are recapitulated in human patients SUS may emerge as a promising AD therapy.

## A8 Scanning ultrasound as a treatment tool of proteinopathies including Alzheimer’s disease

### Jürgen Götz^1^, Rebecca Nisbet^1^, Ann Van der Jeugd^1^, Harrison Evans^1^, Gerhard Leinenga^2^

#### ^1^The University of Queensland, Brisbane (St Lucia Campus), QLD, Australia; ^2^Clem Jones Centre for Ageing Dementia Research, Queensland Brain Institute, The University of Queensland, St Lucia, Queensland, Australia

**Objectives**

Neurological disorders constitute a substantial social and economic burden, as they cause considerable ill health but few direct deaths. Treatment strategies for neurodegenerative diseases are hampered by the fact that the Blood-Brain Barrier (BBB) establishes an efficient barrier for therapeutic agents (Leinenga et al., Nature Reviews Neurology 2016). We have recently shown that scanning ultrasound (SUS) allows microglial-mediated clearance of extracellularly deposited amyloid-beta in APP mutant APP23 mice and restores memory functions in three cognitive tests to wild-type levels, in the absence of overt damage to the brain (Leinenga and Götz, Science Translational Medicine 2015) However, it had not been determined whether SUS treatment reduces the intracellular tau pathology that together with amyloid deposition characterizes Alzheimer’s disease.

**Methods**

We investigated the efficacy of a novel tau-specific single chain antibody fragment, delivered by passive immunization in the human tau transgenic pR5 mouse model, a model of the tau pathology of Alzheimer’s disease (Götz et al., Science 2001). To further assess the efficacy and drug-delivering ability of SUS, we established four experimental groups, using the novel anti-tau antibody that was injected weekly over four weeks, either on its own, or together with SUS. A third group used SUS only, and a fourth was the anaesthesia control group. The mice were analysed on the elevated plus maze, histologically and biochemically. Furthermore, uptake of the antibody by the brain was determine using fluorescently labelled single-chain antibody fragments.

**Results**

A histological and biochemical analysis of the pR5 tau transgenic mice revealed that SUS as well as the employed antibody ameliorated the tau pathology that characterizes the pR5 mice. In addition, the anxiety-like behaviour that characterizes pR5 mice was significantly reduced. We furthermore found enhanced delivery of the antibody using SUS yielding a synergistic therapeutic effect as determined by histology and using the elevated plus maze.

**Conclusions**

Our study suggests that SUS is a method that benefits diseases with protein aggregates more generally, whether they are intra- or extracellular. The therapeutic delivery combined with SUS could offer significant clinical benefits for the treatment of patients with Alzheimer’s disease and related tauopathies. Considering that the yearly costs of passive immunotherapy for AD is expected to exceed $25,000 per patient, combining SUS with antibody delivery could drastically reduce these costs.

## A9 Enhancement of FUS mediated delivery of stem cells to the brain

### Paul Fishman^1^, Paul Yarowsky^2^, Victor Frenkel^3^, Shen Wei-Bin^3^, Ben Nguyen^3^

#### ^1^University of Maryland School of Medicine/Baltimore VAMC, Baltimore, Maryland, USA; ^2^Research & Development Service, VA Maryland Healthcare System and University of Maryland School of Medicine, Baltimore, Maryland, USA; ^3^University of Maryland School of Medicine, Baltimore, Maryland, USA

**Objectives**

FUS mediated Blood-Brain Barrier disruption (BBBD) can enable even large therapeutics such as stem cells to enter brain from the bloodstream and could be a major advance in cell delivery over current invasive methods of brain injection. The efficiency of cellular entry after FUS mediated BBBD alone however is low. We hypothesized that this process could be enhanced by combining it with a complementary strategy termed magnetic targeting. Stem cells can be safely loaded with super-paramagnetic iron oxide nanoparticles (SPION) in culture, allowing cells to be attracted by an external magnet. Our previous study showed SPION loaded stem cells to have enhanced brain retention near a magnet on the skull in a rat model of traumatic brain injury, where BBBD also occurs. The goal of our current project was to determine if magnetic attraction of SPION loaded stem cells would also enhance their delivery to brain after FUS mediated BBBD.

**Methods**

With a small animal MRI guided FUS device (Image Guided Therapy, IGT and 7 T Bruker MRI), we sonicated young adult rats (~120 g) with both radiologic (enhancement of the target region with gadolinium on post-sonication TI MRI), and histologic (staining with Evans’ blue dye) evidence of BBBD, without tissue damage or hemorrhage. Confirmation of the cells within brain as those injected was performed by staining with Perl’s reagent for iron and by immuno-histochemistry with a human specific antigen. The procedure was then combined with the application of a powerful magnet to the head directly after IV injection of hNPCs.

**Results**

With BBBD alone human neuro-progenitor cells (hNPCs) loaded with SPION were observed in rat brain after intravenous (IV) injection directly after sonication only within the treated regions. To demonstrate the effect of magnetic attraction, we injected equal numbers of SPION and non-SPION labeled cells, where each cell type was labeled with a different fluorophore. In animals that had FUS mediated BBBD followed by a magnet applied to the head, significantly greater numbers of SPION labeled cells were observed compared to the non-labeled cells. This result was most pronounced in regions of the brain close to the skull (cerebral cortex) and magnet surface. More powerful magnets including magnetic arrays resulted in more effective retention of SPION labeled cells in even deeper brain regions such as the striatum. There, 90 % of hNPCs observed contained SPIONs compared to 60-70 % with a less powerful magnet.

**Conclusions**

These results demonstrate that the use of magnetic attraction can substantially enhance delivery of stem cells after BBBD. In prior published work, stem cells were delivered to brain after FUS mediated BBBD using cells injected directly into the carotid artery. In an effort to accomplish this goal in a safer and less invasive manner, our study utilized IV cell injection (tail vein), supporting the view that the combination of FUS mediated BBBD and magnetic attraction can allow stem cells to enter brain with a minimally invasive strategy.

## A10 Fluorescent lipid microbubbles for targeted brain drug delivery through the Focused Ultrasound-induced blood-brain barrier opening in vivo

### Carlos Sierra Sanchez, Camilo Acosta, Cherry Chen, Shih-Ying Wu, Maria Eleni (Marilena) Karakatsani, Elisa Konofagou

#### Columbia University, New York, New York, USA

**Objectives**

Focused ultrasound (FUS) in the presence of lipid microbubbles can induce non-invasive, transient and reversible Blood-Brain Barrier (BBB) opening. This study entailed assessment of the feasibility of fluorescently loaded microbubbles, labeled with the fluorophore 5-dodecanoylaminfluorescein (C-12), as a vector for targeted brain drug delivery. Compared to prior studies by our group, where fluorescently-labeled dextrans were co-administered with microbubbles, this new methodology improves the safety and allows a more targeted drug delivery with potentially lower toxicity, avoiding systemic exposure. The main objective was thus to determine feasibility and safety of using the loaded microbubbles as carriers towards targeted brain drug delivery with simultaneous cavitation monitoring.

**Methods**

A spherical, single-element, FUS transducer (center frequency 1.5 MHz) was used. A pulse-echo transducer (center frequency 10 MHz), confocally mounted at the center of the FUS transducer, was utilized for passive cavitation detection (PCD). FUS (pulse length 10,000 cycles; pulse repetition frequency 5 Hz; duration 5 minutes; acoustic pressure 450-750 kPa) targeted mouse brains *in vivo*, in combination with fluorescent microbubbles for C-12 delivery, which was evaluated by *in vivo* transcranial PCD, through the quantification of inertial (ICD) and stable harmonic (SCDh) and ultraharmonic (SCDu) cavitation doses at 30, 60 and 300 s; together with *ex vivo* fluorescence imaging. The BBB opening was verified using *in vivo* T1-w Magnetic Resonance Imaging (MRI). The safety of this technique was assessed through *ex vivo* hematoxylin & eosin staining for microhemorrhage detection and immunohistochemistry (Iba-1 for microglial activation) together with *in vivo* T2-w MRI for edema assessment.

**Results**

**Fig. 5 (abstract A10).**
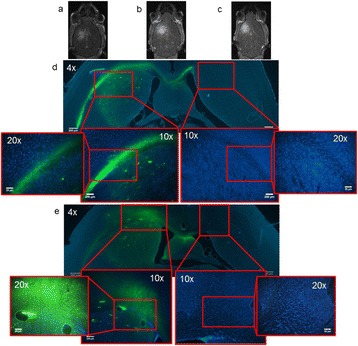
BBB opening and fluorescence delivery: T1-w MRI showing BBB opening at pressures (**a**) 450, (**b**) 600 and (**c**) 750 kPa. Fluorescence delivery (green) and DAPI (blue) in two horizontal sections of mouse brains sonicated at (**d**) 600 and (**e**) 750 kPa

Successful targeted C-12 delivery was achieved at 600 and 750 kPa in six out of 14 cases (Fig. 5). Comparison of ICD, SCDh and SCDu between successful and unsuccessful cases yielded a statistically significant linear relationship between the successful targeted drug delivery and CD and specific thresholds for efficient delivery were identified.

No edema was detected in mice sacrificed on Day 0 but edema appeared on Day 1 on mice sacrificed on Day 7. In all cases cases (except one) it was repaired within a week. Microhemorrhages were observed after sonication in some cases but were also cleared within the first week. However, a higher number of cell nuclei was observed in the sonicated region compared to the unsonicated side in some mice survived up to one week after opening. Iba-1 immunohistochemistry also showed microglial activation.

**Conclusions**

FUS was applied in conjunction with fluorescent microbubbles and, for the first time, the existence of CD thresholds for assessing successful drug delivery was defined. For CD above these thresholds, significant fluorescent enhancement was observed, demonstrating C-12 targeted delivery. One week after sonication, edema was cleared out but microglial activation was observed in certain cases. Therefore, this study indicates the feasibility and safety of a new methodology of FUS-induced BBB opening for targeted albeit potentially riskier brain drug delivery and provides a platform for predicting successful delivery via PCD.

## A11 Ultrasound-mediated delivery of gadolinium and fluorescent–labelled liposomes through the Blood-Brain Barrier

### Muna Aryal^1,2^, Iason T Papademetriou^3^, Yong-Zhi Zhang^2^, Chanikarn Power^1,2^, Nathan McDannold^1,2^, Tyrone Porter^3^

#### ^1^Brigham and Women’s Hospital, Boston, Massachusetts, USA; ^2^Harvard Medical School, Boston, Massachusetts, USA; ^3^Boston University, Boston, Massachusetts, USA

**Objectives**

The main objectives of this study were: 1) to examine whether gadolinium and fluorescent labelled liposomes can extravasate into the brain parenchyma after ultrasound mediated Blood-Brain Barrier disruptions; and 2) to test whether extravasated liposomes were size dependent or not. The liposomes were labelled with gadolinium (Gd) and fluorophore, thus enabling detection of extravasated liposomes via MRI *in vivo* and fluorescence methods in tissue, respectively.

**Methods**

Liposomes labelled with gadolinium and fluorophore were prepared using lipid film hydration and extrusion to two different sizes; ~70-85 nm and ~ 130-150 nm. Animals were divided into two different groups based on the use of particle sizes; group A (~70-85 nm) and group B (~130-150 nm). Focused ultrasound mediated Blood-Brain Barrier disruption (BBBD) was produced in one hemisphere in 15 mice. Particles were injected before sonication. Sonications (0.69 MHz at 0.42 MPa) were performed in two locations combine with Definity (10 μl/kg). Acoustic emissions were recorded during FUS. T1-weighted contrast enhanced and T2*-weighted MRI were used to confirm Gd leakage and damage detection respectively. Mice were euthanized 5-24 hours after FUS and post-process for fluorescence measurement.

**Results**

In T1-weighted contrast enhanced MRI, gadolinium-leakage was able to detect on sonicated area at 5-24 after FUS but not on non-sonicated area (control). Detection of fluorescence signal from brain tissue homogenates confirm the liposomal particles extravasation on sonicated locations. On group A, gadolinium and fluorescence signal intensities on sonicated locations were increased by 26 % and 62 % respectively as compared with control and signal enhancement were statistically significant compared with control ( p = 0.017 and p = 0.02 respectively, two-tailed, paired ttest). On group B, gadolinium and fluorescence signal intensities on sonicated locations were increased by 24 % and 40 % respectively as compared with control. Comparison of fluorescence signal intensities between two groups on sonicated location was statically significant whereas it was not significant on controls (p < 0.05 and p = 0.07 respectively, one-tailed unpaired ttest).

**Conclusions**

Overall, this work demonstrates that ultrasound can deliver upto ~ 150 nm liposomes that labelled with gadolinium and fluorophore through the Blood-Brain Barrier. The results indicate that the extravasation of liposomes were size dependent.

## A12 Sterile inflammatory response (SIR) in the brain following exposure to low intensity pulsed Focused Ultrasound and microbubble infusion

### Zsofia Kovacs^1^, Saejeong Kim^1^, Neekita Jikaria^1^, Farhan Qureshi^2^, Michele Bresler^2^, Joseph Frank^2^

#### ^1^National Institutes of Health, Bethesda, Maryland, USA; ^2^National Institutes of Health Clinical Center, Bethesda, Maryland, USA

**Objectives**

Magnetic Resonance Imaging (MRI)-guided pulsed Focused Ultrasound (pFUS) in combination with systemic injection of microbubbles (MB) is being advocated to increase drug or gene delivery by causing localized Blood-Brain Barrier (BBB) disruption (D). The objective of this study is to investigate the molecular and cellular responses following pFUS + MB associated with BBBD in the rat brain.

**Methods**

**Fig. 6 (abstract A12).**
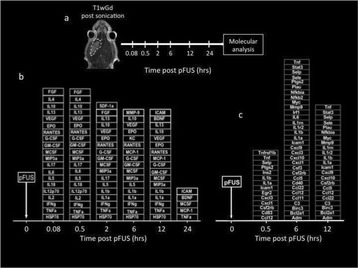
pFUS + MB elicits a transient microenvironmental response in the brain that reflects a sterile inflammation

Female Sprague Dawley rats (<200 g) were sonicated at 0.3 MPa acoustic pressures with 10 ms burst length and 1 % duty cycle (9 focal points, 120 sec/9 focal points) using a single-element spherical FUS transducer (589.636 kHz; FUS Instruments). 100 μl Optison™ MB (GE Healthcare) was administered intravenously. Gadofosveset-enhanced T1w images were obtained with a 3.0 T MRI (Phillips). Proteomic and mRNA expression in the brain following pFUS + MB were analyzed with ELISA, Western blot, quantitative real-time PCR or immunofluorescent staining (Fig. 6a). Proteomics were normalized to sham and statistical analysis was performed by one-way ANOVA corrected for multiple comparisons. Rats were also injected with 8 mg/kg Rhodamine encapsulated magnetic polymers (MicroTRACK™; BioPal) 3 days prior to sonication to label splenic macrophages (CD68) to monitor tropism to the brain.

**Results**

Post contrast T1w MRI and histology showed open BBB without evidence of microhemorrhage. Within 5 minutes following sonication, increased expression of pro-inflammatory and anti-inflammatory cytokines, chemokines and trophic factors (CCTF) was detected in the parenchyma lasting up to 24 hours (Fig. 6b). Increases in heat shock protein 70 (HSP70), tumor necrosis alpha (TNFa), and interleukin (IL) 1a, 1b and 18 consistent with damage associated molecular patterns (DMAP)1 and activation of nuclear factor kappa-light-chain-enhancer of activated B cells (NFkB) inflammatory pathways were observed with SIR to injury2 (Fig. 6b). NFkB pathway-related gene activation along with anti-apoptotic genes, immune cell chemoattractants, selectins, and cell adhesion molecules showed significant (>2fold) increases in mRNA expression (Fig. 6c). Histological analysis showed significant increases (p < 0.05) in the following: number of TUNEL positive cells within 6 hrs, GFAP and Iba1 staining for activated astrocytes and microglia (1-24 hrs) and increase of ICAM up to 24 hrs post sonication. We also detected a >4 fold greater (p < 0.05) CD68 positive cells on day 6 post sonication containing intracellular fluorescent beads within the pFUS + MB treated hemisphere compared to contralateral hemisphere.

**Conclusions**

The temporal molecular response to pFUS + MB is indicative of SIR2 originating from the parenchyma. The pattern of cytokines immediately after pFUS + MB is initiated by cellular release of DAMPs and TNFa observed with mild cerebral trauma or ischemia [1,2]. Increases in monocyte chemoattractant protein (MCP-1), vascular endothelial growth factors (VEGF), stromal derived factor 1 (SDF-1), erythropoietin (EPO) and brain derived neurotropic factor (BDNF) are associated with BBBD stimulating angiogenesis, neurogenesis and stem cell migration observed with ischemia and trauma. These results indicate that pFUS + MB rapidly affects the cerebral vasculature as evident by BBBD in addition to the shockwave from MB collapse that induces mild stress within various cellular elements in the parenchyma inducing a SIR.

**References**

1. Chen, G. Y., et al. 2010 Nat Rev Immunol 10(12):826-37.

2. Gadani, S. P., et al. 2015 Neuron 87(1):47-62.

## A13 Volumetric MR thermometry in a clinical transcranial MR guided Focused Ultrasound system

### Henrik Odéen^1^, George Chiou^2,3^, John Snell^4^, Nick Todd^2^, Bruno Madore^2,3^, Dennis Parker^1^

#### ^1^University of Utah, Salt Lake City, Utah, USA; ^2^Brigham and Women’s Hospital, Boston, Massachusetts, USA; ^3^Harvard Medical School, Boston, Massachusetts, USA; ^4^Focused Ultrasound Foundation, Charlottesville, Virginia, USA

**Objectives**

Transcranial MR-guided Focused Ultrasound (tcMRgFUS) applied within a small central brain volume has achieved excellent outcomes in treatment of movement disorders (Elias NEJM 2013). Although transducers used in tcMRgFUS have been designed with large apertures to spread the beam energy over as much skull as possible to reduce skull and cortex heating, currently used MR temperature imaging (MRTI) methods cannot monitor the temperature increase over the entire insonified brain volume. Instead, temperatures are typically measured in one 2D slice, leaving the majority of the insonified brain volume unmonitored. We have previously developed and published methods to achieve fully 3D MRTI covering the entire insonified brain with good spatial and temporal resolution (Todd MRM 2009/2010) but the techniques have not been evaluated on clinical tcMRgFUS systems. In this work-in-progress study we demonstrate the value of volumetric MRTI with two different pulse sequences applied during heating on a clinical tcMRgFUS system.

**Methods**

PRF MRTI was performed with a product 3D gradient recalled echo (GRE) pulse sequence and a custom-implemented 3D GRE segmented echo planar imaging (EPI) pulse sequence on a 3 T MRI (Discovery 750 T, GEMS). tcFUS heatings were performed in an *ex vivo* human skull filled with tissue-mimicking gel (ATS Laboratories) in a clinical tcMRgFUS system (ExAblate Neuro, Insightec).

Pulse sequences parameters are listed in Table 1. All data were zero filled interpolated (ZFI) to 1-mm isotropic spacing (GRE data additionally ZFI to 0.5-mm spacing). The skull was physically positioned in the FUS system, and the focus electronically steered, to target deep brain-structures located outside the normal treatment envelope. FUS sonications were applied at 940 W for 30/60 s while imaging with the GRE/EPI sequences, respectively. The EPI sequence used an echotrain length of 16 with bi-polar readout gradient, sampling 192 phase encodings in each direction, so that full “positive only” and “negative only” images could be reconstructed.

k-space data were retrospectively down sampled by a factor of R = 4 and 8 (split into multiple time-frames without throwing any data away) for the GRE and EPI experiments, respectively, giving acquisitions times of 3.6 and 7.8 s, and reconstructed with a temporally constrained reconstruction algorithm (Todd MRM 2009).

**Results**

**Fig. 7 (abstract A13).**
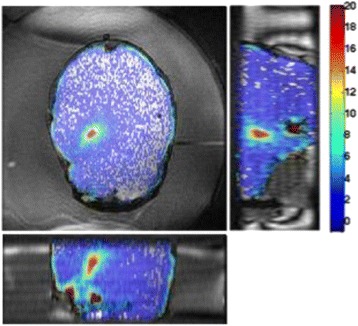
Three orthogonal views of GRE temperature maps overlaid on magnitude image

**Fig. 8 (abstract A13).**
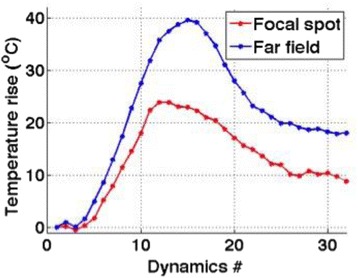
Temporal evolution of heating comparing focal spot to far-field next to petrous bone

Figure 7 shows three orthogonal views of temperature maps overlaid on magnitude images in the GRE experiment. Attempting to focus this far outside the normal treatment envelope results in severe near- and far-field heating. Heating on both the cortex and in the far-field along the petrous bone is visible. In Fig. 8 the temperature evolution at the focal spot is compared to that near the petrous bone. The gel near the bone shows a delayed and greater maximum temperature compared to the focus.

**Fig. 9 (abstract A13).**
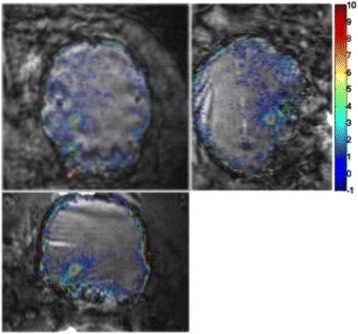
Three orthogonal views of EPI temperature maps overlaid on magnitude image

Three orthogonal views of the larger FOV in the segmented EPI experiment are shown in Fig. 9. Heating along large parts of the cortex can be seen. In the underlying magnitude images it can be seen that the EPI sequence experiences more artifacts than the GRE sequence.

The focal spot position (evaluated as the temperature center-of-mass) was tracked as a function of time during the heating in the GRE data ZFI to 0.5 mm (data not shown). The focal spot did not experience any shift to within the finer ZFI spacing.

**Conclusions**

This study shows that volumetric thermometry over a FOV covering the focal spot and the skull base can be achieved with readily available pulse sequences. With custom implemented pulse sequences the fully insonified FOV (from skull cap to skull base) can be covered. Even though the reconstruction is done retrospectively, the described methods are valuable as research tools in e.g. treatment envelope evaluations. By utilizing 3D imaging ZFI can be performed in all directions to minimize partial volume effects, and very accurate dynamic focal spot localization can be performed.

Future studies will compare the accuracy and precision of the described methods to standard 2D MR thermometry. Experiments comparing 3D MRTI with fiber optic probe measurements at potential target positions outside the currently available treatment envelope will also be performed (Monteith JNS 2016).

**Table 1 (abstract A13).** MR scan parameters. TR – Repetition time, TE – Echo time, FA – Flip angle, BW – Bandwidth (readout), FOV – Field of view, Res – Resolution, Tacq – Acquisition time (before subsampling)

	TR (ms)	TE (ms)	FA (deg)	BW (kHz)	FOV (mm)	Matrix	Res. (mm)	Tacq (s)
GRE	9.2	4	12	15.63	240x240x48	192x96x16	1.25x2.50x3.00	14.3
EPI	52	14.4	30	250	220x220x145	192x192x50	1.15x1.15x2.90	62

## A14 Multi-echo MR thermometry compared to single echo MR thermometry in the treatment of essential tremor

### Kim Butts Pauly, Mike Marx, Pejman Ghanouni

#### Stanford University, Stanford, California, USA

**Objectives**

The choice of receive bandwidth in MR thermometry acquisitions needs to balance two competing choices. Low bandwidths improve SNR, while high bandwidths reduce spatial shift of the hotspot due to the temperature off-resonance. The low bandwidth MR thermometry sequence in use in essential tremor MRgFUS treatments required repeated swapping of phase and frequency directions since the location of the hotspot could only be trusted in the phase encode direction.

A solution is to use multiple high bandwidth acquisitions, which when averaged, regain much of the SNR of the lower bandwidth image. Such a multiecho sequence has recently become available for clinical use. The purpose of this work was to compare the performance of the multi-echo (ME) MR thermometry to the single echo (SE) MR thermometry in clinical treatments of essential tremor.

**Methods**

Fifteen patients were treated for essential tremor, 12 with only single echo thermometry (TE and BW/pix = 12.8 and 44), 2 with only multi-echo thermometry (TE and BW/pix = 3, 8, 13, 18, 22 and 278), and 1 with both sequences. All other image parameters remained the same, including TR = 100. All thermometry images were processed offline in Matlab with a single baseline subtraction (α = -0.00909), followed by referenceless processing for constant and linear terms. The multi-echo thermometry was further processed with a phase unwrapping algorithm in the TE dimension. The multiple echoes were then combined with a weighting by the square of the temperature SNR of each image.

**Results**

The decrease in sampling time for the ME thermometry dictates a theoretical decrease in temperature SNR of only 11 % due to the decrease in sampling time alone. In the one patient that had both sequences in the same scan planes for two sonications, there was a 9 % decrease in SNR in the ME thermometry as measured in the frame used for the referenceless processing, comparing well with theory.

**Fig. 10 (abstract A14).**
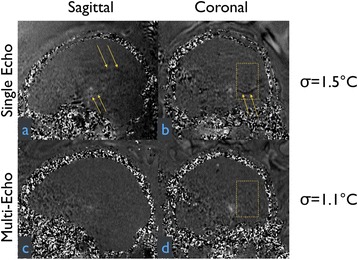
Comparison of MR thermometry images when phase encoding is S/I. The single echo thermometry (**a**,**b**) demonstrates numerous ghosting artifacts (yellow arrows) that are not seen in ME thermometry (**c**,**d**). (**a**,**b**,**d**) are from the same patient

A review of all sonications with ME thermometry when phase encoding was S/I revealed much reduced artifacts over SE thermometry with phase encoding in the S/I direction. Example images are provided in Fig. 10. The region of interest measurement indicated a 30 % improvement in temperature SNR for the ME over the single echo, due to this artifact reduction.

The spatial shifts should theoretically be reduced by a factor of 6.3 in the ME thermometry, as compared to the SE thermometry, due to the increase in receive BW. For example, a 23 °C temperature rise would result in a 0.6 mm shift with the single echo, and only a 0.1 mm shift with the multi-echo.

**Conclusions**

When phase encoding is S/I, multi-echo thermometry is superior to single echo thermometry due to a reduction in ghosting artifacts and spatial shifts. Alignment of the focal spot should not require swapping phase and frequency directions. Future work will include a prospective study to verify this. In the other directions, ME thermometry is comparable to SE thermometry with the reduction of spatial shifts coming with a loss of temperature SNR of about 10 %.

## A15 High-resolution whole-brain MR thermometry with a 3D EPI stack-of-stars pulse sequence

### Sumeeth Jonathan, William Grissom

#### Vanderbilt University, Nashville, Tennessee, USA

**Objectives**

Real-time whole-brain MR thermometry is needed for transcranial MR-guided Focused Ultrasound to accurately track rapid heating at the focus and to monitor for unsafe heating in the near- and far-field. Previous efforts to meet this need have been based on 3D segmented echo planar imaging (EPI) and spiral MR readouts (Fielden et al., 2014). Here, we propose a 3D EPI stack-of-stars temperature mapping pulse sequence that enables greater volume coverage than has been reported with previous approaches. The sequence allows flexible adjustment of the scan acceleration factor and does not require a high density of receive coils for high frame rate thermometry.

**Methods**

**Fig. 11 (abstract A15).**
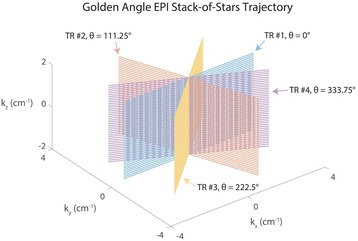
Illustration of the 3D k-space trajectory. A 2D EPI plane is scanned each TR and rotated by 111.25 degrees between TRs to sample a 3D volume. Scan acceleration is achieved by using a small number of consecutive rotated planes/TRs for reconstruction

Readout Trajectory: Fig. 11 shows the proposed readout trajectory. Each TR comprises a 2D EPI plane that is frequency-encoded in the axial (x-y) and phase-encoded in the slice (z) dimensions. The plane is rotated between TRs by 111.25 degrees to sample a golden angle stack-of-stars k-space. The sequence was implemented on a Philips Achieva 3 Tesla scanner with parameters: field-of-view = 28.0 x 28.0 x 11.9 cm, 43 slices, in-plane resolution = 1.50 x 1.50 x 2.75 mm, 17 ms TE/45 ms TR, 14.5° flip angle, spectrally-selective fat suppression.

*In vivo* Experiment: To confirm the achievable brain volume coverage and image quality, with local IRB approval, brain images in a healthy volunteer were acquired without heating using the pulse sequence with one, two, and three interleaved shots and with an 8-channel receive coil array. Fully-sampled images were reconstructed using the density-compensated conjugate gradient method.

Phantom Heating Experiment: A tissue-mimicking gel phantom was sonicated using a clinical MR-HIFU system (Philips Sonalleve) operated at 1.2 MHz and 80 W for 12 s, while scanning with the proposed sequence with one shot and with 5 abdominal receive coils. Temperature maps were reconstructed in one second increments using the k-space hybrid method at three scan acceleration factors.

**Results**

**Fig. 12 (abstract A15).**
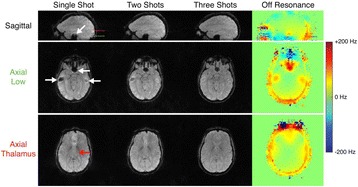
Brain images acquired with the proposed sequence. The sagittal and lower axial slice images contain distortions and dropout in regions with large frequency offsets (white arrows), which are diminished when multiple shots are used to shorten each readout

*In vivo* Results: Distortions and signal dropouts caused by off resonance are visible in the sagittal and lower axial single-shot brain images in Fig. 12 (white arrows). The distortions can be reduced by increasing the number of EPI shots per angle, which increases the pixel bandwidth in z. An axial slice positioned for monitoring Focused Ultrasound thalamotomy (red arrow) is also shown, which contains no visible distortions at any multishot factor.

**Fig. 13 (abstract A15).**
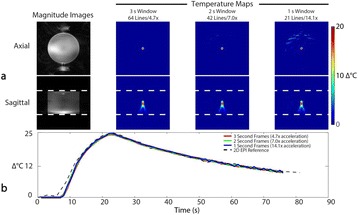
Phantom temperature map reconstructions (**a**) and hot spot temperature curves (**b**) at three acceleration factors. Significant aliasing does not appear until the scan is accelerated by 14.1x. There is good reference standard agreement at all accelerations

Phantom Heating Results: Fig. 13a shows reconstructed sagittal and axial temperature maps through the middle of the phantom at peak heat. Significant temperature aliasing does not appear until the scan is accelerated by a factor of 14.1 or a 1 second window width. The hotspot temperature curve plots in Fig. 13b show that the reconstructed temperatures all coincide, though the 14.1x curve appears noisier. Importantly, at all acceleration factors, there is agreement with a hotspot measurement obtained by a 2D multislice Cartesian EPI temperature mapping pulse sequence (7 slices, same in-plane resolution) which was used as a reference standard.

A video of the entire sonication monitored using this pulse sequence can be viewed at https://www.youtube.com/watch?v=z3cVXjkgyLg.

**Conclusions**

A 3D EPI stack-of-stars temperature mapping pulse sequence was proposed and validated against a 2D multislice Cartesian EPI temperature mapping pulse sequence. The sequence enables fine spatiotemporal resolution with large volume coverage, without requiring temporally-regularized reconstruction or a large number of receive coils.

## A16 Ultrafast and sensitive volumetric passive acoustic mapping

### Costas Arvanitis^1^, Nathan McDannold^2,3^, Gregory Clement^4^

#### ^1^Georgia Institute of Technology, Atlanta, Georgia, USA; ^2^Brigham and Women’s Hospital, Boston, Massachusetts, USA; ^3^Harvard Medical School, Boston, Massachusetts, USA; ^4^Cleveland Clinic, Cleveland, Ohio, USA

**Objectives**

Not released for publication

**Methods**

Not released for publication

**Results**

Not released for publication

**Conclusions**

Not released for publication

## A17 Tissue stiffness imaging with interleaved multiple-point MR-ARFI

### Dennis Parker, Joshua de Bever, Henrik Odéen, Allison Payne, Douglas Christensen

#### University of Utah, Salt Lake City, Utah, USA

**Objectives**

Although Focused Ultrasound (FUS) has the potential to treat a number of pathologies the methods used to guide treatment are still limited in the ability to identify effective treatment endpoints. Changes in MRI parameters such as proton density and T1 and T2 relaxation times may result from reversible edema instead of tissue death. While dynamic contrast enhanced and late Gd-enhanced MRI are more specific, Gd contrast cannot be administered multiple times during a procedure to monitor treatment progression. MRI acoustic radiation force impulse imaging (MR-ARFI) can monitor tissue stiffness, but the acquisition is relatively slow, and must be repeated to measure tissue displacement at more than a single point [1]. The purpose of this work was to develop an efficient method to use MR-ARFI to measure tissue displacement volumetrically with an array of points as a first step towards monitoring tissue stiffness changes during MRgFUS procedures.

**Methods**

A 3D segmented gradient echo (GRE) echo-planar pulse sequence, designed to simultaneously measure tissue displacement with MR-ARFI and the corresponding tissue heating [2–5], was further modified to allow interleaved acquisition of multiple point MR-ARFI (mpMR-ARFI) volumes. Phase change due to displacement was separated from that due to temperature by complex subtraction of an interleaved volume acquired with no FUS applied. Temperature was obtained using the proton resonance frequency method with a referenceless background phase subtraction [6].

Experiments were performed in a gelatin phantom and excised pig brain on 3 T MRI scanners (Siemens Tim Trio and PrismaFit). Pulse sequence parameters were: TR/TE = 73/43 ms, FA = 30°, readout bandwidth = 752 Hz/pixel, echotrain length = 7, FOV = 160x114x55mm, matrix = 128x91x22 giving voxel dimensions of 1.25x1.25x2.5 mm before zero filled-interpolation. Bipolar motion-encoding gradients (MEG) of 15 ms duration (each bipolar lobe) were applied prior to signal readout. Ultrasound pulses of 50 acoustic watts were applied during the second 15 ms bipolar MEG lobe. Navigator echoes were used to measure and compensate for B0 field drift due to gradient heating caused by high MEG strength and duty cycle. The same navigators echoes can be used for respiratory correction *in vivo* [7].

**Results**

**Fig. 14 (abstract A17).**
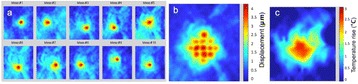
**a** Individual images, **b** Composite displacement, **c** simultaneous heating

Results from a13-point mpMR-ARFI acquisition in a gelatin phantom using the Siemens Tim Trio and MEG = 20mT/m are given in Fig. 14. An example cropped single slice from 10 of the 13 acquired displacement volumes is shown in Fig. 14a with a cropped slice of the mpMR-ARFI composite of all 13 displacement volumes shown in Fig. 14b. The corresponding temperature increase measured during the mpMR-ARFI acquisition is shown in Fig. 14c and was relatively low (<3 °C). (For all mpMR-ARFI images, point separation is 5 mm).

**Fig. 15 (abstract A17).**
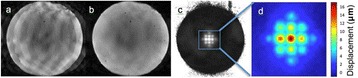
Ghosting (**a**) is corrected by navigator echoes (**b**) allowing clean mpMR-ARFI displacement measurement (**c**,**d**)

Figure 15 shows the improved image quality achieved by the phase-navigator correction in mpMR-ARFI measurements obtained using a PrismaFit 3 T MRI scanner with MEG = 40 mT/m. The ghosting artifact in Fig. 15a due to B0 field drift caused by high amplitude (40mT/m) and duty cycle (40 %) MEG gradients during mpMR-ARFI acquisition. After correction (Fig. 15b) mpMR-ARFI displacement images have negligible artifact.

**Fig. 16 (abstract A17).**
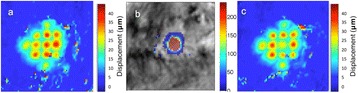
mpMR-ARFI displacement measurements in an excised pig brain before (**a**) and after (c) MRgFUS ablation with estimated cumulative dose in CEM (**b**)

Results of the displacement measured in excised pig brain using the Siemens PrismaFit 3 T MRI scanner and MEG = 40 are shown in Fig. 16 before (Fig. 16a) and after (Fig. 16c) FUS ablation. An estimate of the cumulative thermal dose is shown in Fig. 16b. Note the decreased displacement at central point.

**Conclusions**

Measuring tissue stiffness (displacement as a function of ultrasound intensity) and changes in displacement before, during, and after the procedure provides the unique potential to remotely palpate the ablated volume to monitor the formation of lesions created with MRgFUS. It will also provide a crucially needed assessment of tissue change in regions of fat where conventional MR thermometry fails.

**References**

1. Gerstenmayer M, Magnin R, Fellah B, Le Bihan D, Larrat B. MAGNETIC RESONANCE ACOUSTIC RADIATION FORCE IMAGING FOR IN VIVO ESTIMATION OF ULTRASONIC TRANSMISSION FACTOR THROUGH RAT SKULLS. ISTU; 2016; Tel Aviv, Isreal. P 95.

2. de Bever J, Farrer A, Odeen H, Parker DL. A 3D multi-contrast pulse sequence for acquisition of MR acoustic radiation force imaging concurrently with proton resonance shift thermometry. ISTU; 2014; Las Vegas, Nevada.

3. de Bever J, Farrer A, Odeen H, Parker DL. Simultaneous Acquisition of MR Acoustic Radiation Force Imaging and Proton Resonance Shift Thermometry with 3D Multi-Contrast Pulse Sequence. ISMRM; 2014; Milan, Italy.

4. de Bever J, Odeen H, Parker DL. Measurement of dynamic tissue response to focused ultrasound using 3D MR-ARFI. ISTU; 2014 April 17, 2015; Utrecht, The Netherlands. p Abstract: 2174218

5. de Bever J, Odeen H, Parker DL. Simultaneous Acquisition of Acoustic Radiation Force Imaging and Proton Resonance Frequency Shift Thermometry Using Interleaved Acquisition with Temporally Constrained Reconstruction for Increased Temporal Resolution. ISMRM; 2016; Singapore.

6. Rieke V, Vigen KK, Sommer G, Daniel BL, Pauly JM, Butts K. Referenceless PRF shift thermometry. Magn Reson Med 2004;51(6):1223-1231.

7. Svedin BT, Payne A, Parker DL. Respiration artifact correction in three-dimensional proton resonance frequency MR thermometry using phase navigators. Magn Reson Med 2015; Early View; PMCID: PMC4752934

## A18 Binary localization of cavitation activity based on harmonic content for transcranial brain therapy

### Guillaume Maimbourg^1^, Mathieu David Santin^2^, Alexandre Houdouin^1^, Stéphane Lehericy^2^, Mickael Tanter^1^, Jean Francois Aubry^1^

#### ^1^Institut Langevin Ondes et Images, ESPCI ParisTech, CNRS UMR 7587, INSERM U979, Paris, France; ^2^CENIR, ICM, CNRS U7225, INSERM U975, Paris, France

**Objectives**

Cavitation activity may occur during BBB disruption (due to UCA injections) and also during HIFU treatment due to nucleation under high negative pressure. The corresponding microbubble activity has to be monitored to assess the safety and efficiency of brain treatments by ultrasound. The purpose of this study is to binary discriminate the position of microbubbles, inside or outside the skull, in order to know whether cavitation occurs in the brain of the patient or not.

Binary localization is achieved here by taking advantage of the attenuation properties of the skull. The skull acts indeed as a low pass filter for acoustic signals. Thus we hypothesize that the harmonic content of signals from cavitation events could be used as a binary indicator of their localization: inside or outside the brain case.

**Methods**

A wideband Passive Cavitation Detector (PCD) recorded the acoustic signals from microbubbles activity.

**Fig. 17 (abstract A18).**
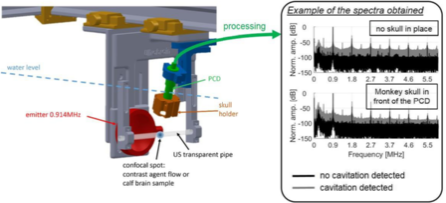
In vitro experimental setup for mimicking BBB opening

An *in vitro* setup (Fig. 17) mimics a BBB opening configuration (contrast agent flow with 0.6 MPa sonication during 10 ms at focus) or a thermal ablation (calf brain sample at focus with 3-4 MPa sonication during 0.3 s at focus). Experiments were performed either with no skull in place, or with human (6 samples) or monkey (1 sample) skull in front of the PCD.

*In vivo* BBB opening were conducted on macaque (900 kHz, 0.6 MPa at focus) with the same PCD mounted on the monkey head.

The spectra are computed from data recorded by the PCD and reveal harmonics, subharmonic and ultraharmonics of the excitation frequency (900 kHz in all cases).

The ratio of a high frequency harmonics over a lower frequency harmonic was then calculated. This ratio is expected to decrease when the acoustic signal from cavitation activity crosses through the skull. In order to achieve binary localization, two types of ratio have been computed: ultraharmonic ratios (e.g. 5/2 over 1/2) and harmonic ratios (e.g. 4 over 2).

**Results**

**Fig. 18 (abstract A18).**
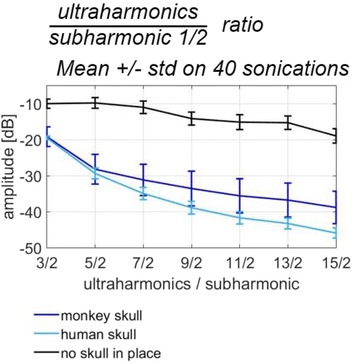
Ultraharmonics ratio (3/2 to 15/2 over 1/2) recorded during in vitro thermal ablation

The ultraharmonics ratio obtained during *in vitro* thermal-like ablation is plotted in Fig. 18. This ratio significantly decreases when acoustic signal crosses through the skull. Thus a threshold can be introduced to binary localize cavitation inside/outside the skull.

Table 2 summarises the results for harmonic ratio 4 over 2 and ultraharmonic ratio 5/2 over 1/2 obtained *in vitro* and *in vivo* during BBB opening and thermal necrosis. For BBB opening, neither the sub- nor the ultraharmonics appears *in vivo*, probably due to the confinment of the largest microbubbles by the vasculature. Thankfully *in vitro* experiments point out that the harmonic ratio remains relevant to binary localize microbubbles in the human case. Indeed the harmonic ratio 4 over 2 exhibits a -30 ± 3 dB decrease when crossing through human skull.

**Fig. 19 (abstract A18).**
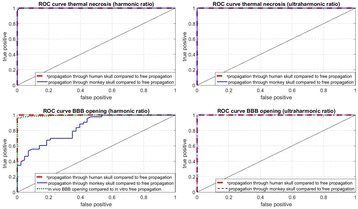
Receiver operating characteristics for ultraharmonic (5/2 over 1/2) ratio and harmonic ratio (4 over 2) during thermal ablation and BBB opening

In order to ensure the repeatability of this method, each configuration was statistically investigated by plotting receiver operating characteristics (ROC) curves. These plots (Fig. 19) illustrate the performance of this approach. Except for *in vivo* BBB opening on monkey, ROC curves demonstrate that a sensitivity and a specificity which are simultaneously nearly 100 % can be obtained for the binary localization of cavitation activity.

**Conclusions**

This preliminary study, mainly done *in vitro*, shows that a low-cost and easy-to-use PCD can binary localize cavitation activity inside/outside the skull using the filtrating effect of the skull on the harmonic content of the spectra. The statistical study shows that the method is able to localize microbubble activity inside or outside the skull with high sensibility and high sensitivity. We look forward to testing extensively this technique *in vivo* for both BBB opening and thermal necrosis.

**Table 2 (abstract A18).** ultraharmonic (5/2 over 1/2) and harmonic (4 over 2) ratio during thermal necrosis and BBB opening in dB scale

			Harmonic 4 Harmonic 2	Ultraharmonic 5/2 Subharmonic 1/2
BBB opening (UCA + US)	in vitro	Reference: no skull	-5	1
		Monkey skull	-8	-21
		Human skull	-35	-26
	in vivo	Macaque	-13	no signal
Thermal necrosis (US only in calf brain)	in vitro	Reference: no skull	-10	-14
		Monkey skull	-29	-38
		Human skull	-28	-30

## A19 Inflection of temperature vs. power curve in tcMRgFUS: correlation with lesion location

### Kim Butts Pauly^1^; Christian Federau^1^; Beat Werner^2^; Casey Halpern^1^; Pejman Ghanouni^1^

#### ^1^Stanford University, Stanford, California, USA; ^2^University Children’s Hospital Zurich, Zurich, Switzerland

**Objectives**

**Fig. 20 (abstract A19).**
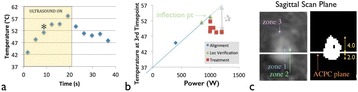
**a** Temperature in the third temperature image (*) **b** does not always rise monotonically with power. **c** The distance from ACPC plane to the top and bottom edges of zone 2 were measured on images thresholded at the zone 1 signal intensity

During tcMRgFUS, power levels are progressively increased to align, verify treatment location, and create a durable ablation. It is predicted that the measured temperature at the focus for a consistent timepoint will increase monotonically with power. However, the temperature rise is often observed to actually decrease with increasing power, creating an inflection in the peak temperature vs. power graph (Fig. 20). The purpose of this work was to show this behavior in the temperature rise is correlated with a lack of alignment between the thermal lesion and the monitored scan plane.

**Methods**

Fifteen ET datasets were included in this study. Post-treatment 3D T2-weighted FSE images (FOV 24 cm, matrix 320x320, slice thickness 1 mm) were thresholded at the signal intensity of zone 1 (Fig. 20). Measurements from the targeted ACPC plane to the top and bottom edges of zone 2 were made in the sagittal plane.

MR thermometry (TE/TR = 12.8/100 (n = 13); TE/TR = 3,8,13,18,22/100, (n = 2)) was processed with a single baseline subtraction (α = -0.00909), followed by referenceless processing for constant and linear terms. The maximum temperature of the third time point (k-space center 8.8 s after sonication initiation) in the axial scan plane was plotted vs. power. This time point was chosen because all sonications were at least 10s in duration. The number of sonications after any inflection was noted.

In 12 cases, temperature-power curves from early sonications in the axial scan plane were extrapolated to higher power levels. The amount that the measured temperature was below the estimated temperature was measured.

**Results**

**Fig. 21 (abstract A19).**
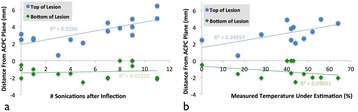
**a** The lesion top edge is moving superior to the ACPC plane with increasing sonication number. **b** As the lesion is moving out of the measured scan plane, the temperature is increasingly underestimated, giving rise to a temperature inflection

The results are shown in Fig. 21. While the distance from the treatment plane to the inferior aspect of the lesion remains essentially constant, the distance from the treatment plane to the superior aspect of the lesion increases with number of sonications after inflection. While it is expected that multiple sonications at the same location may increase lesion size, this data demonstrates that lesion size increases preferentially in the direction of the transducer as we increase sonications after inflection.

The movement of the lesion superiorly from the AC-PC plane is correlated with the difference between the actual and estimated temperature (✰). Although this was quantitated only in the axial scan plane, the inflection was seen in all three scan planes. Prior work demonstrated that the lesion is double oblique (the superior aspect of the lesion is posterior and medial); therefore, movement of the focal spot towards the effective transducer aperture moves it also out of the monitored sagittal and coronal scan planes.

**Conclusions**

One interpretation of this data is that an increase in the acoustic absorption effectively shields the focal spot, with subsequent hotspots located closer to the transducer. A second explanation is that the acoustic properties of the skull may be changing during treatment.

An additional implication of this work is for comparison of simulation with thermometry. To simplify this comparison without the complication of the alignment of the thermometry plane, a sonication early in the treatment should be used for comparison.

## A20 MR-guided Focused Ultrasound pig brain tissue and its histology as a function of thermal dose

### Dong-Guk Paeng^1,3^, Zhiyuan Xu^2^, John Snell^3^, Anders Quigg^3^, Matt Eames^3^, Changzhu Jin^3^, Ashli Everstine^2^, Jason Sheehan^2^, M. Beatriz Lopes^2^, Neal Kassell^3^

#### ^1^Jeju National University, Jeju, Republic of Korea; ^2^University of Virginia, Charlottesville, Virginia, USA; ^3^Focused Ultrasound Foundation, Charlottesville, Virginia, USA

**Objectives**

This study is to investigate the effects of Magnetic Resonance-guided Focused Ultrasound (MRgFUS) on *in vivo* pig brain tissue by comparison of the tissue damage in histology with the changes in MR images as a function of thermal dose (TD) up to 200 cumulative equivalent minutes (CEM) at 43 °C.

**Methods**

We have implemented a PI (proportional-Integral) control system in a laptop with an Arduino-based controller to modulate pulse duration of a FUS system (ExAblate 4000 Neuro 650 kHz system, InSightec) based on the temperature difference between target temperature and focal temperature as measured by an MRI system (Discovery MR75-3.0 T, GE Medical systems) with proton resonant frequency (PRF) thermometry. Accumulated thermal dose in CEM was calculated every 3.7 seconds and used to stop the sonication when a prescribed thermal dose of interest was reached and delivered to the target brain tissue. After tuning of a closed loop control system in a phantom, one acute and seven chronic pig experiments with three-day survival were conducted to investigate the correlation of lesions between the MR images of pig brain tissue with the corresponding histology. Craniectomy was performed to create an acoustic window, and sonication was applied on 4 spots in the thalamus of each pig. Absolute temperature in pig brain tissue was computed based on the MR thermometry using the rectal temperature as a baseline. TD varied from 7 to 195 CEM with target temperature between 46 and 52 °C at appropriate acoustic powers. This pig study was approved by the University of

**Results**

From the acute pig experiment, we observed one large and 2 small lesions on MR images 1 hour after a sonication and subsequent histology showed 4 lesions of target. For the chronic pig experiments, 22 sonication spots in 6 pigs were analyzed through MR images and histology. One pig was excluded due to air bubbles introduced between the dura and scalp during the surgery procedure, and 2 sonication spots were failed to generate due to technical problems. Results show that large brain tissue damage was observed in MR images in all 7 spots with doses larger than 100 CEM and the corresponding histology results confirmed infarction with necrotic center for all except one with a dose of 101 CEM. The diameter of those lesions on T2-weighted axial MR images was measured to 2.9 ± 0. 4 mm (mean ± SD,) with a mean volume of 30.7 ± 12.9 mm3. All with TD lower than 17 CEM produced no visible lesions in either MR images or histology. There was a discrepancy in generating lesions with TD between 18 and 100 CEM, so that six smaller lesions (3 in volume) were shown except one large change in MR images at

**Conclusions**

In conclusion, large tissue damages were observed on MR images and histology for all TD above104 CEM, but no change was shown for all TD below 17 CEM. There is a variability in tissue changes between these TD levels. These results may contribute toward prescription of thermal dose rather than peak temperature or acoustic power for brain treatments, and expand the treatment envelope beyond the current limitations in selecting targets and patients.

## A21 Visualization tools for transcranial Focused Ultrasound procedure planning, simulation and analysis

### John Snell, Anders Quigg

#### Focused Ultrasound Foundation, Charlottesville, Virginia, USA

**Objectives**

The interactions between particular transcranial transducer and skull geometries are challenging to understand without interactive visual computing tools. Such a tool has been created to allow the visual exploration of the estimated treatment envelope of a transcranial transducer given a patient specific imaging dataset. The impetus for developing such a system is to aid in understanding treatment envelope constraints and to serve as a tool for specifying the input to various acoustic simulation systems.

**Methods**

A visualization application was created which makes heavy use of a modern GPU to interactively display a transcranial Focused Ultrasound transducer, patient specific brain and skull anatomy, and the interaction of each transducer element beam path with the skull. The incident angle of each transducer element beam axis with the outer table of the skull is calculated and displayed in an interactive fashion as the natural focus of the transducer is moved within the intracranial volume. Skull geometry is derived from a treatment compatible CT dataset. An MR dataset is registered with the CT for targeting. Due to the interactive nature of the tool, presumptive targets can be rapidly and intuitively explored and understood in terms of geometric constraints and estimated treatment efficiency.

**Results**

The visualization system functions will be demonstrated including targeting, transducer positioning and assessment of transducer efficiency in terms of effective transducer element count.

**Conclusions**

An interactive visualization system has proven valuable for facilitating understanding of the complex interaction of transducer and skull geometries. Future applications of this system may include patient screening, post-treatment analysis, indication feasibility screening and acoustic simulation initialization.

## A22 HIFU for Pediatric Operations (HOPE) – a pediatric neurosurgical treatment system

### James Drake^1^, Karl Price^2^, Lior Lustgarten^1^, Vivian Sin^2^, Charles Mougenot^3^, Elizabeth Donner^1^, Emily Tam^1^, Mojgan Hodaie^4^, Adam Waspe^1^, Thomas Looi^2^, Samuel Pichardo^5^

#### ^1^Hospital for Sick Children, Toronto, Ontario, Canada; ^2^Centre for Image Guided Innovation and Therapeutic Intervention, Toronto, Ontario, Canada; ^3^Philips Healthcare Canada, Toronto, Ontario, Canada; ^4^Toronto Western Hospital, Toronto, Ontario, Canada; ^5^Thunder Bay Regional Research Institute, Thunder Bay, Ontario, Canada

**Objectives**

Pediatric patients have distinctive neuroanatomic features and specific disorders that make them unique candidates for transcranial MR-guided Focused Ultrasound treatment. Children have thinner skulls, and neonates in particular, possess a natural acoustic window through their fontanelle. This results in lower phase aberration and decreases the need for the larger hemispherical dome transducers used for current adult transcranial procedures. These systems also require fixation of the head in a stereotactic frame, which is dangerous for their fragile skull. In addition, neonates often require MR-compatible incubators and dedicated neuro-interventional coils for imaging. Focal brain ablation/disconnection for medical refractory epilepsy and lysis of intra-ventricular hemorrhage are some of the proposed noninvasive treatment options for this population. To provide such treatment, we have developed HOPE – HIFU for Pediatric Operations – an integrated neonatal HIFU treatment system.

**Methods**

**Fig. 22 (abstract A22).**
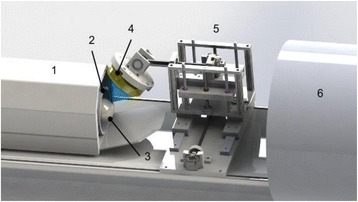
HOPE Concept (1 – incubator, 2 – coil, 3 – neonatal patient, 4 – transducer, 5 – robot and 6 – MR bore)

HOPE is composed of multiple elements: a 5 degree of freedom MR-compatible robot positioning device, a 256 element phased-array transducer, an 8 channel neuro-interventional coil, and a real-time Python-based treatment planning and delivery software system. The specifications for HOPE were determined by a team of clinicians and researchers as follows: compatible with clinical neonatal incubators and MRI techniques, imaging coil integrated into the incubator, positioning system to couple and deliver the HIFU treatment and real-time control/monitoring of the treatment (Fig. 22). The hardware elements are designed and tested with a Philips Achieva 3.0TX MRI with an Imasonic 256 element transducer. For coupling, a custom designed water bag system was created to interface with the patients’ head. The software platform includes robot kinematics, robot visualization, registration, transducer control and MRI communication. A calibration procedure was developed using a series of Vitamin E markers visible in a 3D gradient echo sequence without fat suppression; the markers were attached to an extension of the robot that recreates the acoustic cone of the HIFU beam. A user-interface module was developed to calculate the center of mass of each marker and to co-register the coordinate systems of the MRI and the robot.

**Results**

**Fig. 23 (abstract A22).**
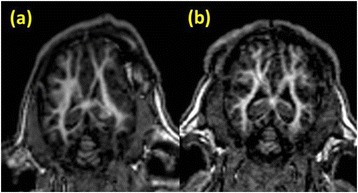
Coil comparison in a T1-TFE image of a porcine brain in vivo (Left: Prototype 8 channel neuro-interventional coil, Right: Philips clinical 32 channel head coil)

**Fig. 24 (abstract A22).**
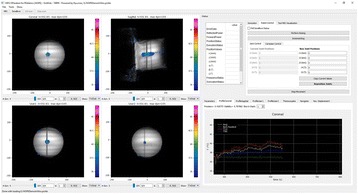
HOPE software interface with robot, treatment planning and real-time monitoring of HIFU exposure in phantom material

The hardware system of HOPE has been designed and tested to show it can perform T1, T2 and DTI imaging that is comparable to clinical coils while delivering HIFU treatment in a phantom model. The neuro-interventional coils provide high resolution images for treatment planning (Fig. 23). The MR-conditional robotic system positions the transducer to an accuracy of 0.59 +/- 0.25 mm and delivers thermal ablation treatment to targets in a Philips HIFU quality assurance phantom. The custom water coupler bag provided a transmission path for the HIFU with minimal energy loss. The HOPE software platform controls each of the robotic positioning axes with hardware safety switches in a real-time Python interface (Fig. 24). The software registration of the patient, MRI and robot frame allows the user to select specific brain targets. During treatment, real-time thermometry is displayed.

**Conclusions**

HOPE is an MR-guided Focused Ultrasound system aimed at delivering HIFU therapy (both thermal ablation and cavitation-based treatment) for neonatal and pediatric patients. The system has been designed to operate within an incubator and clinical MRI system which minimizes the impact to the patient. Future work involves characterizing the treatment accuracy and performing *in vivo* animal studies to test the overall system feasibility and usability for treatment of epilepsy and IVH clots. Other treatments that will be investigated are thermal ablation of brain tumors, hyperthermia and targeted drug delivery.

## A23 Simultaneous stimulation of the human primary and secondary somatosensory cortices using transcranial Focused Ultrasound

### Wonhye Lee^1^, Yong An Chung^2^, Yujin Jung^2^, In-Uk Song^2^, Seung-Schik Yoo^1^

#### ^1^Brigham and Women’s Hospital, Boston, Massachusetts, USA; ^2^Incheon St. Mary’s Hospital, The Catholic University of Korea, Incheon, Republic of Korea

**Objectives**

Low-intensity transcranial Focused Ultrasound (FUS) is making progress as a new mode of non-invasive brain stimulation, having potential for superior spatial selectivity and depth penetration compared to transcranial direct current stimulation (tDCS) or transcranial magnetic stimulation (TMS). With accumulating evidences of the FUS-mediated neuromodulatory effects in small and large animal models, the sonication given to the primary somatosensory cortex (SI) has recently shown to be capable of eliciting tactile sensations in humans. Here, we further investigated the creation of tactile sensations induced by simultaneous FUS stimulation of the secondary somatosensory cortex (SII) and SI of the hand.

**Methods**

Ten healthy volunteers (two females, ages = 23–34, average of 27.8 ± 4.1 yrs) participated, and all procedures were conducted under the approval of the local Institutional Review Board of the Catholic University of Korea. For targeting of FUS focus to the individual functional neuroanatomy, each participant’s brain image was acquired using a 3-T MR scanner with anatomical (T1-weighted) and functional MRI (fMRI, T2*-weighted) protocols. The SI and SII areas were mapped while using four types of external tactile stimuli to the palm of the right hand—vibrotactile, pressure, warmth, and coolness. CT scan of the head was also acquired for the planning of transcranial sonication path. As guided by these individual-specific neuroimage data, FUS sonication (210 kHz, single-element FUS transducer with focal length of 38 mm) was administered to the SI and SII simultaneously (or separately), with an incident acoustic intensity of 35 W/cm2 Isppa, tone-burst-duration of 1 ms, pulse-repetition frequency of 500 Hz (yielding a 50 % duty cycle), and a sonication duration of 500 ms. The SII was targeted as divided into four sub-regions that are specifically activated by the external tactile stimuli.

**Results**

Across the differential selective stimulations (i.e., SI only, SII only, or SI and SII simultaneously), participants felt various types of elicited tactile sensations (while ‘tingling’ was dominant) from the hand areas contralateral to the sonication, such as the palmar/dorsal side of the hand or as single/multiple adjacent fingers. These results were similar to our previous study on FUS stimulation of the SI, while the elicitations of ‘vibrotactile’ and ‘warmth’ sensations were newly reported in the present study. The types of tactile sensations did not match to the sensations that are associated with the specific sub-regions in the SII. However, two individuals reported matching types of sensations (‘vibrotactile’, ‘pressure’, and ‘warmth’) during stimulation of the SI/SII simultaneously or the SII only. The stimulatory effects of the FUS were transient and reversible, and the sonication procedures did not induce any discomforts or adverse changes in the subjects’ physical/mental status.

**Conclusions**

Simultaneous stimulation of the SI/SII in the same hemisphere was achieved by using multiple FUS transducers, which elicited various types of tactile sensations. Stimulation of the SII only also induced the creation of tactile sensations. The ability to stimulate multiple region-specific brain areas may shed light on examining the causal relationships between regional brain activities and subsequent behavioral/cognitive outcomes.

## A24 Transcranial Focused Ultrasound stimulation of the primary visual cortex in humans

### Wonhye Lee^1^, Hyun-Chul Kim^2^, Yujin Jung^3^, Yong An Chung^3^, In-Uk Song^3^, Jong-Hwan Lee^2^, Seung-Schik Yoo^1^

#### ^1^Brigham and Women’s Hospital, Boston, Massachusetts, USA; ^2^Department of Brain and Cognitive Engineering, Korea University, Seoul, Republic of Korea; ^3^Incheon St. Mary’s Hospital, The Catholic University of Korea, Incheon, Republic of Korea

**Objectives**

Transcranial Focused Ultrasound (FUS) has been suggested as a new non-invasive modality of regional brain stimulation, with potential to be more spatially-selective and to reach deep cortical/subcortical areas compared to the conventional methods of transcranial magnetic stimulations (TMS) or transcranial direct current stimulation (tDCS). In humans, low-intensity FUS sonication has been demonstrated to temporarily change the neural activities in the primary somatosensory cortex (SI), based on the observations of subjective sensory manifestations and electrophysiological responses. However, functional neuroimaging evidence of increased neural activity in the stimulated region, as well as the associated network-wide brain responses, has not yet been shown in humans. Here, we administered stimulatory FUS to the primary visual cortex (V1) as guided by the individual-specific neuroanatomy. Concurrent functional MRI was acquired to assess the brain regions that were activated due to the stimulation.

**Methods**

19 healthy volunteers (five females, ages 20–45, average 26.1 ± 5.4 yrs) participated, and all procedures were conducted under the approval of the Institutional Review Boards of both the Catholic University of Korea and Korea University. Functional MRI (fMRI; for mapping of the visual areas) and cranial CT were obtained from each participant to provide the individual-specific V1 location for sonication planning/targeting. Then, in a separate session, an MR-compatible sonication setup (270 kHz, single-element FUS transducer with radius-of-curvature of 30 mm) was used to deliver FUS to the V1 under a clinical 3-T MR scanner for the image-guidance and the simultaneous acquisition of fMRI data. Separate from the FUS-fMRI session, electroencephalographic (EEG) potentials elicited by the FUS stimulation were also measured. We used a pulsing scheme having a sonication duration of 300 ms with a tone-burst-duration of 1 ms repeated at a pulse repetition frequency of 500 Hz (yielding a 50 % duty cycle). The incident acoustic intensity at the FUS focus was 16.6 W/cm2 Isppa. Retrospective numerical simulation of the transcranial acoustic wave propagation was performed proximal to the sonicated area to estimate the *in situ* acoustic intensity and spatial accuracy of sonication.

**Results**

Simultaneous acquisition of fMRI during FUS sonication to the V1 revealed the elicited activation not only from the sonicated brain area, but also from the network of regions involved in visual and higher-order cognitive processes. Accompanying phosphene perception was also reported. The EEG responses showed distinct peaks associated with the sonication, having similarities with the classical visual evoked potentials (VEP) generated by photic stimulation. The procedures did not induce any discomforts or adverse effects from the participants, based on the subjective reporting and neuroradiological/neurological examinations. Retrospective numerical simulation of the transcranial FUS suggested the variability in individual responsiveness to the stimulation.

**Conclusions**

Simultaneous fMRI acquisition during FUS application to the V1 revealed the functional neuroimaging-based evidence in humans that the FUS stimulation activates the sonicated brain area and concurrently elicits the associated phosphene perception. Successful stimulation of the V1 was also supported by the presence of the evoked EEG potentials associated with FUS. The individual variability in responsiveness to the stimulation suggested needs for an elaborate image-guidance.

## A25 Focused Ultrasound modulation of visual search performance and associated EEG in monkeys

### Charles Caskey, Wolf Zinke, Josh Cosman, Jillian Shuman, Jeffrey Schall

#### Vanderbilt University, Nashville, Tennessee, USA

**Objectives**

Focused ultrasound (FUS) is a promising tool for neuromodulation because of its noninvasivness and better spatial precision compared to other noninvasive methods, such as transcranial magnetic stimulation (TMS) or transcranial direct current stimulation (tDCS). FUS neuromodulation has been demonstrated in multiple animal models, including a prior study where ultrasound was applied transcranially over macaque frontal eye field (FEF) to influence saccade response time in an anti-saccade task. In this work, we applied FUS through a craniotomy over the macaque FEF while measuring saccade response times and EEG signals associated with selective attention.

**Methods**

A single element focused transducer was positioned through a craniotomy over FEF, a cortical area that plays a key role in the eye movement and attention systems. FUS stimulation was applied during a complex visual search task where the monkey was required to shift its gaze to a target among distractors. We alternated blocks of trials with or without FUS stimulation (300 ms of pulsed FUS with a 50 % duty cycle starting 150 ms before search display onset, center frequency 500 kHz, repetition frequency 2 kHz, pulse duration 0.25 ms, peak negative pressures of 250 kPa or 425 kPa, warming of brain tissue < 1.5 °C). Saccade response time and intracranial EEG recordings were acquired in two monkeys performing 9 sessions each.

**Results**

In both monkeys, event-related potentials (ERPs) associated with selective attention (N2pc) were significantly reduced during stimulation with both intensities. FUS stimulation attenuated the N2pc over the entire session block, rather than on a trial-by-trial basis. In one monkey with a craniotomy positioned directly over FEF, the mean saccade response times were reduced by 5 ms by FUS stimulation at 425 kPa (p < 0.001) when the target appeared in the upper hemifield contralateral to the FUS stimulation, while another animal with a craniotomy more ventrally did not show such a systematic behavioral modulation.

**Conclusions**

We are continuing to explore potential spatial relationships between stimulation location and behavioral modulations in ongoing work. Overall, our findings demonstrate prolonged FUS modulation of attention ERPs and suggest potential spatial selectivity based on the location of stimulation.

## A26 Ultrasound-mediated modulation of motor and ocular responses in anesthetized mice in vivo

### Christian Aurup^1^, Shutao Wang^1^, Hong Chen^2^, Camilo Acosta^1^, Elisa Konofagou^1^, Hermes Kamimura^3^, Antonio Carneiro^3^

#### ^1^Columbia University, New York, New York, USA; ^2^Washington University in St. Louis, St. Louis, Missouri, USA; ^3^Universidade de Sao Paolo, Sao Paulo, Brazil

**Objectives**

Focused ultrasound has been identified as a non-invasive technique for modulating brain activity. Most studies involving sedate rodents utilize frequencies in the kilohertz-range, which allow for optimal transmission of acoustic power through the skull. The tradeoff with using lower frequencies involves producing larger acoustic foci and resultant poor target-specificity. Megahertz-range frequencies can therefore be used to improve target-specificity. This study demonstrates that Focused Ultrasound in the megahertz range can be used to evoke motor and ocular responses in mice under deep anesthesia by targeting cortical and subcortical structures, respectively. Contralateral-paired hind limb movements were observed when stimulating cortical regions, demonstrating the ability of megahertz-range FUS to stimulate activity in highly-targeted regions. Additionally, pupil dilation was observed when deep-seated anxiety-related structures were targeted, demonstrating the ability of FUS to modulate activity in a small subcortical structures.

**Methods**

For this study, wild-type adult male mice were anesthetized with intraperitoneal injections of sodium pentobarbital (65 mg/kg) and fixed in a stereotaxic frame. A single-element FUS transducer with fundamental frequency of 1.94 MHz was fixed to a 3D positioning system for accurate navigation through the brain. A 6x6 mm grid centered +2 mm anterior of the lambda skull suture was sonicated in a random order using a center frequency of 1.9 MHz, pulse repetition frequency of 1 kHz, 50 % duty cycle, 1 second pulse duration, 1 second inter-pulse interval for a total of 10 pulse repetitions. The acoustic pressure applied was varied in order to evaluate thresholds for eliciting physiological responses like motor movement, eye movement, or pupil dilation. Motor movements were validated using video recordings and intramuscular electromyography recordings from the biceps femoris in both hind limbs. Pupil movement and dilation from subcortical modulation were evaluated using a high-resolution camera aimed at the right eye and frame-by-frame processing technique.

**Results**

**Fig. 25 (abstract A26).**
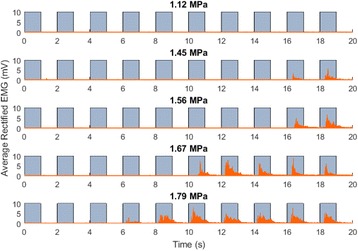
Evaluation of the pressure threshold when applying FUS to location within the somatosensory cortex. This location resulted in contralateral hind-limb movement relative to the sonication site

**Fig. 26 (abstract A26).**
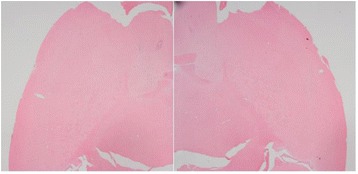
Histological evaluation of brain at 1.93 MPa (left) and 3 MPa (right) revealed now red blood cell extravasation

The minimum peak rarefactional pressure required to elicit hind limb movements was 1.45 MPa when targeting cortical regions, calibrated using an excised mouse skull. Higher pressures increased the success rate from 20 % (at the 1.45 MPa threshold) to 70 % (1.79 MPa) (Fig. 25). Targeting eye-motor and anxiety-related regions of the brain elicited eye movements and pupil dilations up to 20 %. Sonicating the superior colliculus resulted in both eye movement and pupil dilation at a lower threshold pressure (1.20 MPa) than the hippocampus and locus coeruleus, which required pressures greater than 1.80 MPa. A histological evaluation performed in five mice at 1.93 MPa and 3 MPa peak rarefactional pressure resulted in no red blood cell extravasation (Fig. 26).

**Conclusions**

This study successfully demonstrated that megahertz-range Focused Ultrasound can be used to elicit motor and ocular responses with high specificity in mice *in vivo*. It was also shown that the success rate of stimulation increased with acoustic pressure for motor movements associated with cortical modulation but depends greatly on the region of the brain targeted. These findings emphasize the complex and yet to be determined mechanism of action involved in ultrasonic neuromodulation.

## A27 Non-invasive neuromodulation via targeted delivery of neurotransmitter chemicals

### Nick Todd^1^, Tao Sun^1,2^, Yong-Zhi Zhang^2^, Chanikarn Power^1,2^, Navid Nazai^3^, Sam Patz^1^, Margaret Livingstone^2^, Nathan McDannold^1,2^

#### ^1^Brigham and Women’s Hospital, Boston, Massachusetts, USA; ^2^Harvard Medical School, Boston, Massachusetts, USA; ^3^Boston University, Boston, Massachusetts, USA

**Objectives**

Focused ultrasound (FUS)-microbubble treatment has been used to open the Blood-Brain Barrier (BBB) for targeted delivery of a wide variety of therapeutics. Here we propose to deliver neurotransmitter chemicals such as GABA or glutamate for the purpose of non-invasive neuromodulation. These chemicals function to transmit or suppress signals across the chemical synapses that connect neurons in the brain. This novel approach affects signaling between neurons, as opposed to existing neuromodulation techniques that affect the transmission of electrical signals along neurons. Such an approach could be an important new complimentary tool for basic neuroscience or lead to new therapies for neurological disorders.

Previously, we used electrophysiology measurements to demonstrate functional blockade via BBB disruption and GABA administration. Here we present initial results demonstrating the proof of concept in a rodent model using delivered GABA to modulate neuronal activity and functional MRI to measure the effects.

**Methods**

Sprague-Dawley rats underwent bilateral hindpaw electrical stimulation (1-5 mA, 0.3 ms duration, 2 Hz) to elicit a functional response of the somatosensory network. Varying levels of GABA were systemically injected under conditions No BBB opening and BBB opening. Functional activity in the thalamus and S1 was measured using fMRI to quantify any effects of neuromodulation.

BBB opening: Microbubbles injected (Optison, 200 μl/kg), 274 kHz dual aperture transcranial FUS with 32 ms bursts applied at 4 Hz for 60 seconds.

GABA delivery: Systemic tail vein bolus injection in doses from 10 mg/kg to 50 mg/kg.

fMRI: Images acquired on a Bruker 7 T scanner with a single shot EPI sequence (TR = 1.5 s, TE = 18 ms, 18 slices, 300 images). Stimulation performed in a 40 s OFF, 20 s ON block design over 7.5 total minutes. T-scores obtained using general linear model analysis in SPM 12.

**Results**

**Fig. 27 (abstract A27).**
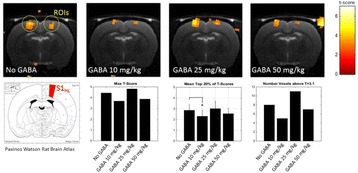
fMRI results without BBB opening. Top: T-score values overlaid on a T1w image. Bottom: Paxinos/Watson rat brain atlas with hindleg S1 area colored red and bar plots for t-score metrics comparing the activity for the various GABA doses. * = p < 0.05

BBB Closed: Fig. 27 shows activation results in S1 for the case of No BBB opening. Compared to the baseline case of No GABA injected, a GABA injection of 10 mg/kg showed significant decrease in activity (p < 0.05) but GABA injections of 25 mg/kg and 50 mg/kg did not.

**Fig. 28 (abstract A27).**
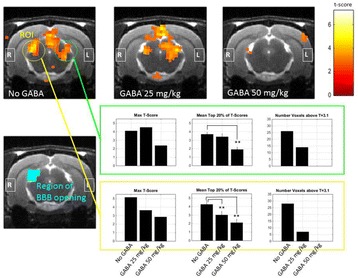
fMRI results with BBB opening in right thalamus. Top: T-score values overlaid on a T1w image. Left: Extent of BBB opening. Bar plots show t-score metrics from right ROI (yellow/opened) and left ROI (green/closed). ** = p < 0.001

BBB Open: Fig. 28 shows activation results in the thalamus for the case of BBB opening. BBB opening was targeted, and confirmed through gadolinium imaging, in the right hemisphere. Bilateral activation was seen in the thalamus for the baseline case of No GABA injected. For GABA injection of 25 mg/kg, a significant decrease in activation was seen in the right (opened) ROI (p < 0.001), but not the left (unopened) ROI. For GABA injection of 50 mg/kg, a significant decrease in activation was seen in both ROIs (p < 0.001).

**Conclusions**

More experiments on a number of rats are necessary to confirm and expand these findings. However, these very preliminary results are a promising indicator that a neurotransmitter such as GABA can be delivered through the opened BBB for targeted manipulation of neuronal activity.

## A28 Initial experience in a pilot study of Blood-Brain Barrier opening for chemo-drug delivery to brain tumors by MR-guided Focused Ultrasound

### Todd Mainprize^1^, Yuexi Huang^2^, Ryan Alkins^3^, Martin Chapman^3^, James Perry^4^, Nir Lipsman^1^, Allison Bethune^1^, Arjun Sahgal^4^, Maureen Trudeau^4^, Kullervo Hynynen^1^

#### ^1^Sunnybrook Health Sciences Centre, University of Toronto, Toronto, Ontario, Canada; ^2^Physical Sciences Platform, Sunnybrook Research Institute, Toronto, Ontario, Canada; ^3^University of Toronto, Toronto, Ontario, Canada; ^4^Sunnybrook Research Institute, Toronto, Ontario, Canada

**Objectives**

Magnetic Resonance-guided Focused Ultrasound (MRgFUS) has been shown to reversibly open the Blood-Brain Barrier (BBB) for targeted drug delivery [1]. Research on animal models, including non-human primates [2], has been conducted to investigate the effectiveness and characteristics of BBB openings. Here we describe our initial experience in a pilot clinical study to establish the feasibility, safety and preliminary efficacy of Focused Ultrasound to temporarily open the BBB to deliver chemotherapy to brain tumors.

**Methods**

**Fig. 29 (abstract A28).**
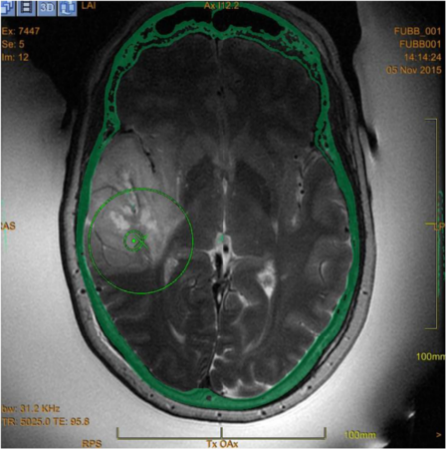
Intraoperative T2w MR image showing the tumor and the first BBB target

This phase-one clinical trial of BBB opening by Focused Ultrasound was approved by Health Canada. A modified clinical MRgFUS brain system (ExAblate 4000, 230 kHz, Insightec, Tirat Carmel, Israel) was used with a 3 T MR scanner (Signa MR750, GE Healthcare, Milwaukee, WI, USA). Two hours before the procedure, liposomal doxorubicin Caelyx (Janssen, Toronto, Ontario, Canada) was intravenously infused over 1 hour at a dose of 30 mg/m2. The patient’s head was then shaved and positioned in the FUS array with a stereotactic frame. Two targets close to the posterior margin of the glial tumor were chosen based on T2 images (Fig. 29). Each target consisted of a 3x3 grid of 9 spots at 3 mm spacing. For each spot, 2.6 ms on, 30.4 ms off FUS pulses were repeated for 300 ms before steering to the next spot. The pattern was repeated periodically resulting in an overall pulse repetition frequency (PRF) for each spot of 0.9 %. A bolus injection of 4 ul/kg of Definity microbubbles (Lantheus Medical Imaging, N. Billerica, MA, USA) was applied simultaneously with each sonication (1/5th of the clinical dose for ultrasound imaging). With the first injection of microbubbles, 10s short sonications at 5 W, 7 W and 9 W acoustic power were applied to find the appropriate power level based on feedback of cavitation signals. Cavitation signals were detected by two receivers and sampled at a rate of 2 MHz. Spectrum integration from 75 kHz to 155 kHz was calculated and two threshold levels of the spectrum integration were defined as a safety mechanism based on pre-clinical studies on a trans-human skull pig model [3]. 9 W was found to be adequate for these targets. 50 s sonications at 9 W were then applied at each target, with a separate bolus injection of microbubbles for each. Post sonication, Gd (Gadovist, Bayer)-enhanced 3D FSPGR images were acquired to verify the BBB openings, and T2*-weighted GRE images (TE = 15 ms) were collected to detect potential hemorrhage. After the treatment, the patient was released from the head frame and MR scans were repeated with an 8-channel head coil for better quality images. The patient underwent routine tumor resection the next day and tissue samples at the two BBB opening targets were collected for quantification of chemotherapy drug concentration.

**Results**

**Fig. 30 (abstract A28).**
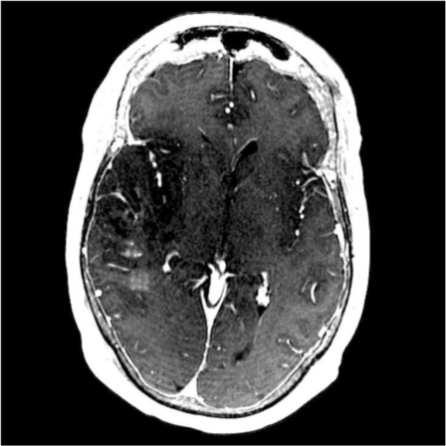
**a** Axial Gd-enhanced T1w MR images showing the first (top arrow) and second (bottom) BBB openings

**Fig. 31 (abstract A28).**
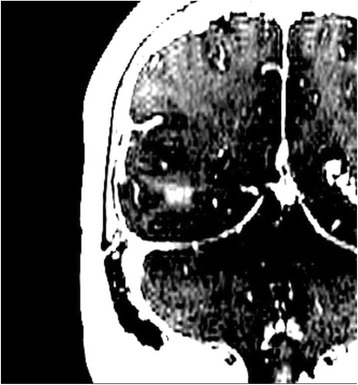
**b** Coronal view across the second target

**Fig. 32 (abstract A28).**
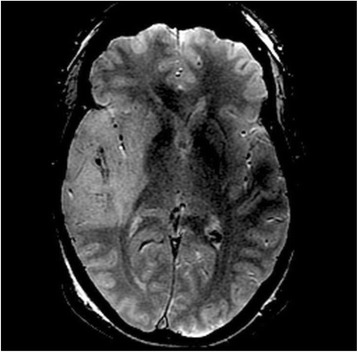
T2*w image shows low level of RBC extravasation (small dark spots) within the two target volumes

The opening of the BBB was successful at both locaitons with clear Gd enhancement in the 3x3 grid pattern. (Figs. 30 and 31). Despite using the same power level, the actual acoustic pressure at the 2nd target was lower than the first due to steering of the FUS beam. Low-level extravasation of red blood cells were seen as small dark signals within individual sonicated sponts in the T2* image (Fig. 32). The quantification of drug concentration is pending further analysis.

**Conclusions**

The 3 mm spacing of the 9 spots was intentionally designed to form a grid pattern of Gd enhancement for easier confirmation in heterogeneous tumors for the initial cases. We do not expect an impact on other parameters if the spacing needs to be reduced for a more uniform drug distribution within the BBB opening volume. There was a small level of RBC extravasation but this was not a concern in the tumor enviromment. Our animal experiments have shown that the cavitation signal can be used during the sonications to control the power level for eliminating the RBC exravasations [4]. The current system did not use this method during sonications.

The tumor in this patient was in the right temporal lobe adjacent to the skull. The two targets were ~4 cm lateral from the midline of the brain, and the 2nd target was also ~2.5 cm posterior. Thermal ablations by FUS at these off-centre locations are technically challenging due to excessive skull heating. However, successful BBB openings at these locations were demonstrated at low powers at 230 kHz. If these results can be repeated in other patients without complications, then the method may provide a new way to deliver therapeutic agents into brain for the treatment of tumors and other brain diseases.

**References**

1. Hynynen K et al. Radiology 2001;220:640-6.

2. McDannold N et al. Cancer Research 2012;72:3652-63.

3. Huang Y et al. ISMRM 2015, abstract 37.

4. O’Reilly MA et al. Radiology 2012;263:96-106.

## A29 Enhanced bevacizumab delivery to the CNS by Focused Ultrasound induced blood-brain barrier opening for malignant glioma treatment

### Hao-Li Liu^1^, Po-Hung Hsu^1^, Kuo-Chen Wei^2^

#### ^1^Chang Gung University, Taoyuan, Taiwan; ^2^Chang Gung Memorial Hospital, Taoyuan, Taiwan

**Objectives**

Malignant glioma is the most severe form of primary brain tumors with an extremely high recurrence rate and the poorest prognosis. Current anti-angiogenic monoclonal antibody (mAb) treatment failed to show therapeutic efficacy due to transient “vascular normalization” stage that restores BBB integrity in tumor regions and restricts anti-angiogenic mAb penetration, preventing angiogenic suppression of tumor cells in the CNS and diminishing the improvement in overall survival in clinical treatment observation. The purpose of this study is to demonstrate that transcranial Focused Ultrasound (FUS) enhances Blood-Brain Barrier (BBB) permeability of the antiangiogenic monoclonal antibody, bevacizumab, for glioblastoma multiforme (GBM) treatment.

**Methods**

**Fig. 33 (abstract A29).**
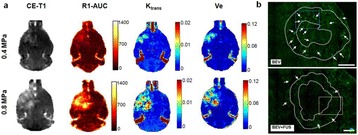
**a** Representative imaging indexes obtained from DCE -MRI analysis (T1-WI, R1-AUC, Ktrans, and Ve). **b** CD-31 IHC fluorescent microcopies. Bar = 100 μm

Transcranial FUS in the presence of microbubbles was used to transiently open BBB, and enhance CNS penetration of bevacizumab in normal and glioma-bearing mice. Bevacizumab was quantitated by high-performance liquid chromatography (HPLC), and Western blotting confirmed bevacizumab in the CNS. Bevacizumab permeability was estimated *in vivo* via contrast-enhanced Magnetic Resonance Imaging (CE-MRI), and glioma progression was longitudinally followed via T2-MRI. Morphological changes and vascular inhibition were confirmed histologically with H&E and CD-31 immunohistochemistry (Fig. 33).

**Results**

**Fig. 34 (abstract A29).**
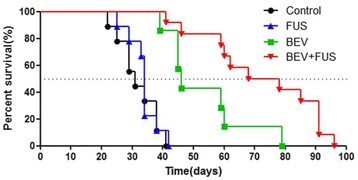
Kaplan–Meier plot to demonstrate animal survival among each experimental groups. BEV = Bevacizumab

HPLC confirmed that FUS significantly enhanced CNS delivery of bevacizumab from 5.7- to 56.7-fold. The high correlations between CE-MRI imaging indices and bevacizumab concentration (r2 = 0.56-0.7378) suggested feasible non-invasive *in vivo* imaging of large-molecule BBB penetration. FUS-enhanced bevacizumab delivery significantly inhibited glioma progression, and improved median survival (ISTmedian = 135 %, compared to 48 % in bevacizumab-administration alone, Fig. 34).

**Conclusions**

In conclusion, anti-angiogenic glioma therapy is enhanced via our proof-of-concept study that FUS enhances large molecule bevacizumab BBB permeability, and combining Focused Ultrasound to open the Blood-Brain Barrier with bevaczumab delivery can overcome bevaczumab vascular normalization and potentiate bevacizumab’s anti–angiogenic tumor therapy effect.

## A30 Closed-loop control of targeted drug delivery across the blood-brain barrier in rat glioma models

### Tao Sun^1,2^, Chanikarn Power^1,2^, Yong-Zhi Zhang^2^, Jonathan Sutton^1,2^, Phillip Alexander^1,2^, Muna Aryal^1,2^, Eric Miller^3^, Nathan McDannold^1,2^

#### ^1^Brigham and Women’s Hospital, Boston, Massachusetts, USA; ^2^Harvard Medical School, Boston, Massachusetts, USA; ^3^Tufts University, Medford, Massachusetts, USA

**Objectives**

Microbubble-mediated Focused Ultrasound (FUS) can induce targeted drug delivery through Blood-Brain Barrier disruption (BBBD). Real-time feedback control of cavitation is critical to realize desired treatment outcome while avoiding tissue damage. Here, we propose an acoustic emissions-based controlling paradigm that can sustain stable cavitation (harmonic emission, HE) while suppressing inertial cavitation (broadband emission, BE). Our objective is to deliver desired drug dose by controlling HE strength during BBBD, while keeping the brain damage-free.

**Methods**

A dual-aperture FUS setup (f  =  274.3 kHz) produced a sub-centimeter focal depth in rats’ brain (n = 50) *in vivo*, and a passive detector (fcentral = 650 kHz) monitored cavitation activity. HE and BE were analyzed during 32-ms bursts in real time and fed back for control of the next pulse. The impact of multiple FUS parameters and microbubble (Optison) injection protocol on the controller performance was studied. To avoid inertial cavitation, the pressure was reduced if BE was detected and terminated if it crossed a set threshold. Both wild type and F98 glioma (ATCC # CRL-2397) models have been used in this study. Delivery of a model drug (Trypan Blue; 960 Da) and chemotherapeutic drug (Doxorubicin) was assessed using fluorescent imaging of formalin-fixed tissue blocks 1-h post sonication.

**Results**

**Fig. 35 (abstract A30).**
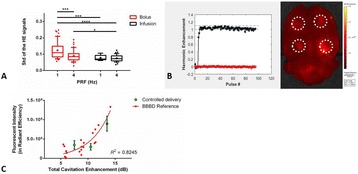
**a** Signal stability assessment (*: P < 0.05, ***: P < 0.001, ****: P < 0.0001); **b** Cavitation control profile (HE in black and BE in red) and Trypan blue delivery; **c** Calibrated BBBD correlation (in red) and controlled delivery results (in green)

Pilot study demonstrated the HE-pressure linearity (R2 = 0.93) and found that the BE threshold decreased as bubble dose (up to 400 μl/kg) was augmented. To optimize controller performance in sustaining HE while suppressing BE, a Phase-1 study demonstrated that: 1) 4-Hz PRF (compared to 1-Hz, P < 0.001) significantly suppressed the HE signal variance; 2) Infusing microbubbles after an initial bolus prevented the decline of HE and a corresponding increase in pressure, and further improved HE stability (P < 0.05 for all comparisons, Fig. 35a) while reducing the likelihood of BE (33.7 % vs. 16.7 %).

Using optimal settings, a Phase-2 study investigated HE control and Trypan Blue delivery (Fig. 35b). Integrated HE was exponentially correlated with epi-fluorescence intensity (R2 = 0.82; red symbols in Fig. 35c). Based on this calibration, a Phase-3 study tested if we could deliver a desired amount of drug by sonicating until the HE reached a preset goal. The resulting fluorescent intensity matched well with the reference curve for three different goals (n > 5 per group, green symbols in Fig. 35c).

**Conclusions**

Our proposed controlling system and method has been demonstrated to effectively sustain the stable cavitation behavior while suppressing the inertial cavitation at a minimum level. Moreover, this real-time closed-loop controller can enable the reliable delivery of a pre-determined amount of drug to the brain.

## A31 MR-guided Focused Ultrasound for antibody delivery in a brain metastasis model

### Thiele Kobus^1^, Yong-Zhi Zhang^2^, Nathan McDannold^2,3^

#### ^1^Radboud University Medical Center, Nijmegen, Netherlands; ^2^Harvard Medical School, Boston, Massachusetts, USA; ^3^Brigham and Women’s Hospital, Boston, Massachusetts, USA

**Objectives**

Treatment options for breast cancer patients with metastases in the brain are limited. The blood-brain barrier (BBB) prevents most drugs from entering the brain and tumor. Although survival in patients with extracranial metastases of HER2-positive breast cancer can be prolonged by the use of antibody therapies like trastuzumab and pertuzumab, the response of brain metastases to these drugs is poor [1]. We aim to improve antibody delivery by temporary disruption of the BBB using magnetic resonance (MR)-guided focused ultrasound (MRgFUS) in combination with microbubbles. The treatment benefit was evaluated using MR imaging.

**Methods**

The treatment effect of the antibodies trastuzumab and pertuzumab in combination with MRgFUS to disrupt the BBB was evaluated. MDA-MB-361 cells (HER2-positive human cancer cells) were injected in the brain. Six weekly treatments started 5 weeks later. Three groups of 10 nude rats were included: a control-group that received no treatment; a group that only received both antibodies; and a FUS + antibody-group that received the antibodies in combination with BBB disruption using MRgFUS. The ultrasound treatments took place in a 7 T MR-system using a spherically-focused 690 kHz-transducer. Before and after the sonications, T2-weighted (T2w) and T1w images were obtained for targeting and confirmation of BBB disruption. At the start of each sonication (duration 60s, 10-ms bursts, burst repetition frequency 1 Hz), the ultrasound contrast agent Optison (100 μl/kg) was injected. The complete tumor was treated in 4 to 14 sonications using peak negative pressures between 0.46 and 0.62 MPa.

After the sonications gadolinium (Magnevist) was injected and T1w was repeated to confirm BBB disruption. The difference in signal intensity change in pre- and post-contrast T1w images was determined between the tumor and contralateral brain region (=ΔSI%). In two animals tumor leakiness was studied before the tumors were sonicated and quantified similarly.

Every other week, high-resolution T2w imaging was performed to determine tumor volume. The volumes were fitted with: volume(*t*) = a*exp(r*t), in which *r* is the growth rate and *t* is the time in days. *r* was determined for the treatment period (week 5 to 11) and the follow-up period (week 11 till sacrifice). An animal was classified as ‘responder’ if the growth rate *r* was lower than the mean *r* of the control animals minus two standard deviations.

The animal was euthanized if its condition was poor or the tumor diameter exceeded 13 mm. Brains were stained for hematoxylin and eosin (H&E) and HER2.

**Results**

BBB disruption was successful in all sessions with an average ΔSI% of 21.2 % (range 4.5–77.6 %). The mean ΔSI% of two tumors before BBB disruption during the six treatment weeks were 0.4 % and 0.6 %, indicating that the tumors were not leaky before disruption.

In the FUS + antibody-group, 4/10 animals were classified as responders during the treatment period with an average growth rate of 0.010 ± 0.007, compared to 0.043 ± 0.013 for the non-responders. There was no difference in the average ΔSI% of the responding rats (21.8 % ± 16.7) and the non-responding rats (20.7 % ± 9.7). None of the control or antibody-only animals were classified as responder. For the follow-up period, none of the animals was classified as responder.

High-resolution T2w imaging showed that the tumor was homogenous in most animals till week 13-15, when cystic and necrotic areas started to develop. The tumors showed also a heterogeneous appearance on H&E-stained sections and the complete tumor was HER2-expressing in the examined brains (Fig. 37).

**Conclusions**

In this study, we demonstrate that BBB disruption using MRgFUS in combination with antibody therapy can slow down the growth of breast cancer brain metastasis. As the tumors were not leaky before BBB disruption and there were no responders in the antibody-only group, the disruption of the BBB is necessary for drug delivery to these brain metastasis. Part of the rats responded to the treatment, while the other animals had the same growth rate as the control-group. This is in line with a previous study [2], where antibody therapy was combined with FUS in a different breast cancer brain metastasis model and only in part of the animals a response was observed. The difference in response could not be explained by the tumor volume at the start of the treatment, nor was there a difference in contrast-enhancement after BBB disruption or in HER2-expression. Better understanding of why certain animals respond is needed and will help in translating this technique to the clinic.

**References**

1. Pieńkowski and Zielinski. 2009. Ann Oncol: 917–24.

2. Park et al. 2012. J. Control. Release: 277–284

**Fig. 36 (abstract A31).**
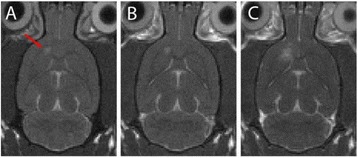
**a** T1weighted images before contrast administration. The red arrow indicates the tumor. **b** No difference in enhancement of the tumor is observed after contrast administration (ΔSI = 0.4 %). **c** After focused ultrasound-mediated Blood-Brain Barrier disruption, the tumor enhances after contrast administration (ΔSI = 30.1 %)

**Fig. 37 (abstract A31).**
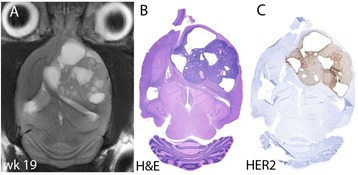
**a** T2w image of an animal that not responded to the therapy and was euthanized after imaging (at week 21 after implantation). The tumor shows a heterogeneous appearance with cysts **b** The H&E-stained section shows a similar appearance. **c** HER2-stained section shows that the complete tumor is HER2-expressing

## A32 Phase I/IIa clinical trial of blood-brain barrier disruption by pulsed ultrasound

### Alexandre Carpentier^1^, Michael Canney^2^, Alexandre Vignot^3^, Kevin Beccaria^1^, Delphine Leclercq^1^, Cyril Lafon^4,5^, Jean Yves Chapelon^5^, Khe Hoang-Xuan^6^, Jean-Yves Delattre^1^, Ahmed Idbaih^1^

#### ^1^Hopital de la Pitié-Salpêtrière, Assistance Publique Hopitaux de Paris, Paris, France; ^2^CarThera, Denver, Colorado, USA; ^3^CarThera, Lyon, France; ^4^University of Virginia, Charlottesville, Virginia, USA; ^5^INSERM Unit 1032, Lyon, France; ^6^Institut du Cerveau et de la Moelle épinière, ICM, Paris, France

**Objectives**

The Blood-Brain Barrier (BBB) limits the delivery and efficacy of systemically administered drugs to the brain. Pre-clinical studies have demonstrated that ultrasound-induced disruption of the BBB can significantly increase the intracerebral concentration of chemotherapy. To harness this approach, our group has developed an implantable ultrasound device, SonoCloud®, that allows for simple and repeated disruption of the BBB. The goal of this work was to determine the safety of repeated disruption of the BBB using the SonoCloud® device in patients with recurrent glioblastoma prior to receiving carboplatin chemotherapy.

**Methods**

A Phase 1/2a clinical trial was initiated at the Hospital Pitie Salpetriere in Paris, France in July 2014. Patients with recurrent glioblastoma were implanted with an 11.5-mm diameter biocompatible 1 MHz ultrasound transducer. The device was fixed to the skull bone in a standard burr hole, after which the skin was closed. Once a month, the device was connected to an external generator system using a transdermal needle connection and patients received a 150 second ultrasound sonication (25,000 cycles/burst, 1 Hz) in combination with systemic administration of an ultrasound contrast agent. BBB disruption was monitored immediately after sonication using gadolinium-enhanced Magnetic Resonance Imaging (MRI). Systemic intravenous injection of carboplatin chemotherapy (AUC 4-6) was administered immediately following acquisition of each patient’s post-sonication MRI (<1 hr after BBB disruption). Patients followed a progression of ultrasound dose in which the acoustic pressure was increased from 0.5 to 1.1 MPa throughout the course of the study.

**Results**

Fifteen patients were included in this on-going study from July 2014-January 2016. A total of 41 BBB disruption sessions were performed. Contrast-enhanced MRI indicated that the BBB was disrupted at acoustic pressure levels up to 1.1 MPa without detectable adverse effects on MRI or clinical examination.

**Conclusions**

Our preliminary results from this Phase I/IIa study indicate that repeated disruption of the BBB using the SonoCloud® device is safe and well tolerated in patients with recurrent GBM and may improve chemotherapy delivery in the brain.

## A33 Preliminary report on sonodynamic therapy for C6 glioma rat model

### Zhiyuan Xu^1^, David Moore^2^, Alexis Xu^1^, Paul Schmitt^3^, John Snell^2^, Jessica Foley^2^, Matt Eames^2^, Jason Sheehan^1^, Neal Kassell^2^

#### ^1^University of Virginia, Charlottesville, Virginia, USA; ^2^Focused Ultrasound Foundation, Charlottesville, Virginia, USA; ^3^University of Virginia Health System, Charlottesville, Virginia, USA

**Objectives**

Not released for publication

**Methods**

Not released for publication

**Results**

Not released for publication

**Conclusions**

Not released for publication

## A34 In vivo porcine histotripsy brain treatments

### Jonathan Sukovich^1^, Charles Cain^1^, Zhiyuan Xu^2^, Aditya Pandey^1^, John Snell^3^, Neeraj Chaudhary^1^, Sandra Camelo-Piragua^1^, Steven Allen^1^, Dong-Guk Paeng^4^, Jon Cannata^5^, Dejan Teofilovic^5^, Jim Bertolina^5^, Neal Kassell^3^, Timothy Hall^1^, Zhen Xu^1^

#### ^1^University of Michigan, Ann Arbor, Michigan, USA; ^2^University of Virginia, Charlottesville, Virginia, USA; ^3^Focused Ultrasound Foundation, Charlottesville, Virginia, USA; ^4^Jeju National University, Jeju, Jeju, Republic of Korea; ^5^HistoSonics, Inc., Ann Arbor, Michigan, USA

**Objectives**

Focused ultrasound thermal therapies have been explored for brain applications including treatment of essential tremors, Parkinson’s disease, and stroke. Histotripsy, a cavitation based ultrasound therapy, is being investigated for treatment of brain tumor and intracerebral hemorrhage, but there are concerns that excessive hemorrhage may be induced in the brain during histotripsy treatment by disrupting blood vessels. This study investigates the *in vivo* feasibility and safety of generating targeted lesions in the porcine brain using histotripsy. The goal is to demonstrate that lesion generation may be accomplished safely and without excess hemorrhage, edema, or other major complications associated with treatment. We also seek to investigate the histotripsy dose required to fully ablate target brain regions.

**Methods**

Histotripsy treatments were delivered to brains of 11 pigs using a 1.5 MHz focused transducer following a craniotomy. Lesions generated were targeted in the cortex at depths of 5 to 20 mm from the exposed surface of the brain. Using ultrasound imaging guidance, treatment regions were targeted to be contained within individual gyri to avoid perforations into the sulci. During dosage studies, single lesions were generated using between 1 and 200 histotripsy pulses delivered at ≤10Hz PRF with an estimated peak negative pressure of 45 MPa. Large lesions were generated by incrementally repositioning the histotripsy target site during treatment to ablate entire target volumes. 7 acute pigs were sacrificed within 6 hours of treatment and brains were removed for MRI and histology. 4 sub-acute pigs were survived for 3 days after treatment. To assess lesion damage, hemorrhage and edema surrounding the lesions, MR images of the brains were acquired at 1 hour and at 3 days after treatment for sub-acute pigs. After euthanization brains were dissected for histology.

**Results**

In 4 acute pigs, with ultrasound guidance, histotripsy was used to generate precise single lesions of 1.5x2.5 mm within the gyri. Dosage studies revealed that a single histotripsy pulse was sufficient to generate identifiable damage in the brain in MRI and complete cell fractionation in lesions was achieved by 50 pulses. In 3 acute pigs, 5 larger lesions measuring up to 7 mm diameter were generated in the brain. MRI and histology revealed that lesions confined to within the gyri show no hemorrhage or other major complication associated with treatment. In 4 sub-acute pigs, 6 single lesions were generated by 10 and 50 pulses, and two larger square lesions of 3.5 mm were created. MRI following treatment showed no hemorrhage or damage outside the target regions and at 3 days showed no additional hemorrhage and only minor edema near the lesion boundary. No midline shift or brain herniation was observed. Histology showed damage confined to within target volumes, with well demarcated boundaries between treated and untreated tissues and no evidence of hemorrhage, ischemic changes, encephalitis, or acute, sub-acute or chronic inflammatory infiltrate beyond the areas of the lesion.

**Conclusions**

The results of this study demonstrate that histotripsy may be used to generate targeted lesions in the brain without causing excess hemorrhage, edema, or other major complications associated with treatment. Lesions were observed to have well defined boundaries between treated and untreated tissues, with little damage beyond the confines of the lesion area. Tissues surrounding the lesions appear very viable with no acute, sub-acute or chronic inflammatory infiltrate, and tissues in adjacent gyri appear uninvolved and unremarkable. These results demonstrate that histotripsy may be applied in the brain without causing major complications and suggest the potential of histotripsy for use in brain therapy applications.

## A35 Neuronavigation-guided Focused Ultrasound and real-time acoustic mapping: evaluation in non-human primates with blood-brain barrier opening

### Shih-Ying Wu, Maria Eleni (Marilena) Karakatsani, Julien Grondin, Carlos Sierra Sanchez, Vincent Ferrera, Elisa Konofagou

#### Columbia University, New York, New York, USA

**Objectives**

**Fig. 38 (abstract A35).**
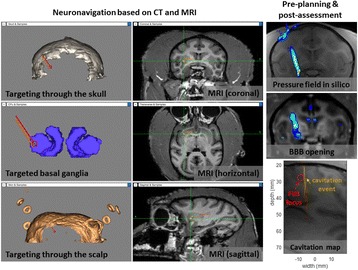
Treatment preplanning, on-line targeting and monitoring, and post assessment

Focused ultrasound (FUS) has shown great promise for noninvasive brain treatment, including surgical ablation, Blood-Brain Barrier (BBB) opening and drug delivery, and neuromodulation. Accurate and precise targeting with personalized planning is the key for treatment success. Furthermore, repetitive procedure is often required for BBB opening and drug delivery as well as neuromodulation, which entails the demand for a portable system apart from the current MR-guided FUS system requiring a dedicated and custom-built suite. In this study, a neuronavigation-guided FUS system was developed (Fig. 38), combined with in silico preplanning based on CT and MRI as well as real-time acoustic mapping in visualizing the location and intensity of the cavitation events associated with BBB opening. The system was evaluated in non-human primates with BBB opening, with the treatment preplanning and targeting accuracy validated both *in silico* and *in vivo*.

**Methods**

Three rhesus macaques were sonicated (N = 15) and the subcortical structure of the basal ganglia was targeted, which is associated with neurodegenerative diseases including Parkinson’s and Huntington’s disease. Both CT and MRI were acquired for personalized pre-planning and neuronavigation guidance (Brainsight). The 3D numerical simulation of the acoustic pressure field was performed for pre-planning and post comparison with the BBB opening. A single-element, 0.5-MHz FUS transducer (diameter: 64 mm) with in-house microbubbles were used for sonication, and a programmable data acquisition system (Verasonics) with an array of acoustic detectors for real-time passive cavitation mapping. Both the FUS and cavitation mapping were guided with the neuronavigation system in real time during the FUS procedure. After sonication, the contrast enhanced T1-weighted MRI was used to confirm the location and size of BBB opening. The accuracy of both the FUS targeting and cavitation mapping were assessed.

**Results**

The target shift was on average 2.0 mm laterally and 3.5 mm axially in the in vivo experiment, and the shift due to the skull was predicted to be 0-1 mm laterally and 1.0-5.5 mm axially in silico. Real-time cavitation mapping confirmed the sonicated area with and without BBB opening, and distance between the centroid of the cavitation map and that of the resulting BBB opening was under 2 mm. In order to achieve the desired BBB opening volume, the pressure and focal spot size in situ were estimated in silico and tailored for targeting in each subject. Simulation results showed a smaller focal spot size through the skull (2.6 mm laterally and 16.7 mm axially, compared with the original size of 4.0 mm laterally and 35.3 mm axially), which corresponded to the BBB opening volume under specific peak negative pressures (NHP 1 at 200 kPa, NHP 2 at 600 kPa). This pressure difference between individuals was due to the difference in skull attenuation, since the in silico pressure reduction was estimated to be 30.9 % ± 12.4 % and 53.9 % ± 14.3 % in NHP 1 and NHP 2, respectively.

**Conclusions**

In conclusion, the new portable neuronavigation-guided FUS with in silico preplanning and real-time cavitation mapping was shown feasible and could facilitate the BBB opening and neuromodulation applications while maintaining translational capability to a clinical setting.

## A36 Definition of basic properties of physical immunotherapy in pancreatic cancer using HIFU and immune checkpoint inhibition

### Gail ter Haar^1^, Petros Mouratidis^1^, Elizabeth Repasky^2^

#### ^1^The Institute of Cancer Research, Sutton, Surrey, United Kingdom; ^2^Roswell Park Cancer Institute, Buffalo, New York, USA

**Objectives**

The latest advances in cancer immunotherapy have improved our understanding of how to stimulate the patient’s immune system to fight their own disease. Notwithstanding the unparalleled success of treating melanoma tumours and non-small cell lung cancer with immune checkpoint inhibitors, patients are not all getting the same benefit. In this context, pancreatic cancer presents unique challenges. Its dense stroma characteristics result in limited immune cell infiltration in the tumour microenvironment. In addition the low number of mutations in pancreatic cancer cells result in few tumour antigens, thereby reducing their immunogenicity. HIFU may address some of these limitations by ablating segments of the tumour to induce a stronger inflammatory response and degrade the stroma to facilitate enhanced diffusion of tumour antigens out of the tumour, and increased penetration of lymphocytes to the tumour. We propose to co-treat pancreatic tumours with HIFU and immune checkpoint inhibitors to elicit an effective anti-tumour immune

**Methods**

In vitro characterization of the effects of heat on the presentation of cell surface receptors associated with the immune response has been undertaken in human colon cancer HCT116 cells and mouse pancreatic cancer Panc02 cells using FACS analysis. A KPC-derived pancreatic cancer cell line will be transplanted in C57BL/6 subjects to form syngeneic subcutaneous and orthotopic pancreatic tumours. These tumours will be treated with HIFU, and within vivo monoclonal anti-CLTA-4 and anti-PD-1 antibodies. The phenotype of markers of the adaptive immune response will be assessed to identify whether HIFU exposures can initiate intrinsic anti-tumoural cellular immunity at the site of treatment. Analysis by flow cytometry and immunohistochemistry of both effector cells (cytotoxic CD8+ T cells, dendritic cells), and immunosuppressive cells (Tregs, myeloid derived suppressor cells), as well as cytokines and chemokines at various time points post HIFU will be performed. Tumour growth will be determined using high resolution ultrasound imaging, and the results will be correlated with overall survival.

**Results**

Thermal exposure of cancer cells results in a time-dependent regulation of the transmembrane receptor CD47. CD47 decreases immediately after, and 1 day after treatment, and increases 3 and 4 days after treatment relative to the sham-exposed cancer cells. Further data on the effects of thermal exposures on the regulation of immune-associated pancreatic cancer cell surface receptors will be presented.

**Conclusions**

Detailed pre-clinical studies in various mouse models of pancreatic cancer can provide a mechanistic understanding of the combinatorial effects of HIFU and immune checkpoint inhibitors. Strong evidence that HIFU stimulates endogenous immunity against pancreatic tumor cells, and augments the immunotherapy treatment of poorly immunogenic pancreatic tumours could be used as proof-of-concept for the initiation of Phase 1 clinical trials of HIFU-enhanced immunotherapy in patients with pancreatic cancer.

## A37 Melanoma growth control via ultrasound depends on the adaptive immune system and surpasses αPD-1

### Kelsie Timbie^1,2^, Lena Badr^2^, Benjamin Campbell^2^, John McMichael^2^, Andrew Buckner^2^, Jessica Prince^2^, Aaron Stevens^2^, Timothy Bullock^2^, Richard Price^2^

#### ^1^Focused Ultrasound Foundation, Charlottesville, Virginia, USA; ^2^University of Virginia, Charlottesville, Virginia, USA

**Objectives**

Melanoma incidence continues to rise, while Stage IV 5 year survival remains below 20 %. Targeted immunotherapy approaches, designed to enhance natural anti-tumor mechanisms or inhibit the immunosuppressive tumor microenvironment, have shown promise in early clinical use. The programmed death receptor and its ligand (PD-1 and PD-L1) have drawn considerable attention. PD-L1 is known to inhibit the activity of anti-tumorigenic cytotoxic T cells, promoting a pro-tumor immune profile, and ultrasound (US) can promote the infiltration of immune cells into the tumor. Here, we tested the hypothesis that a combination of pulsed US, microbubbles (MBs) and anti-PD-1 will improve tumor growth control in a mouse model of melanoma in a manner dependent on the adaptive immune system. Melanoma incidence continues to rise, while Stage IV 5 year survival remains below 20 %. Targeted immunotherapy approaches, designed to enhance natural anti-tumor mechanisms or inhibit the immunosuppressive tumor microenvironment, have shown promise in early clinical use.

**Methods**

Eight week old immunocompetent C57BL/6 male mice were inoculated unilaterally with B16-F10 melanoma cells and divided into four groups: Control (untreated), US + MBs, αPD-1, or US + MBs + αPD-1. Immunocompromised Rag1-/- mice were similarly inoculated and divided into two groups: Control (untreated) or US + MBs. On day 11 post-inoculation, animals receiving US were anesthetized and sonicated with a 0.75” unfocused 1 MHz transducer as previously described1 for 60 minutes. MBs (105/g b.w.) were injected i.v. throughout the sonication. The αPD-1 groups were given an IP injection of 250 μg of αPD-1 on Days 10, 13 and 16. Tumors were monitored until Day 18, when tumors were resected for flow cytometry. Helper T cells (CD4+), cytotoxic T cells (CD8+), T regulatory cells (Treg), macrophages and natural killer cells (NK) were counted in immunocompetent mice, and dendritic cells (DC), natural killer cells, M1 and non-M1 macrophages were counted in immunocompromised mice.

**Results**

**Fig. 39 (abstract A37).**
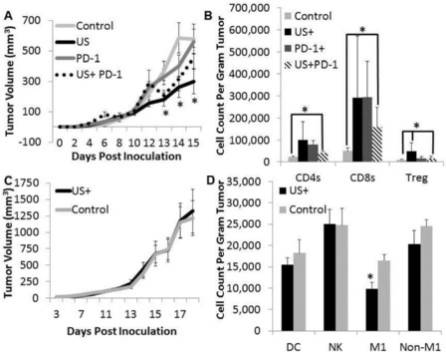
**a** Tumor growth in immunocompetant C57BL/6 hosts. *P < 0.05 vs. control. **b** Immune cell representation in tumors in C57BL/6 hosts. **c** Tumor growth in immunocompromised Rag1-/- hosts. **d** Immune cell representation in tumors in Rag1-/- hosts

In immunocompetent mice, treatment with US + MBs provided improved tumor growth control compared to all other groups (Fig. 39a) as well as a significant survival benefit (not shown). Additionally, US + MBs generated a significant increase in macrophages (not shown) and Tregs (Fig. 39b) compared to control. Interestingly, the αPD-l and US + MBs groups performed similarly in CD4+ and CD8+ cell counts (Fig. 39b), but the combined US + MBs + αPD-1 treatment generated fewer T cells than either treatment alone. In immunocompromised animals, treatment with US + MBs did not provide tumor growth control (Fig. 39c) or a survival benefit (not shown), but did generate a significant decrease in M1 macrophages within the tumor (Fig. 39d), although total myeloid cell counts remained the same (not shown).

**Conclusions**

US indiscriminately increases the numbers of immune cells within subcutaneous melanoma; therefore, we expected that US-enhanced delivery of αPD-1 would provide an additional benefit; namely, a shift towards an anti-tumorigenic immune profile. However, αPD-1 did not synergize with US + MB treatment. While the mechanism is not yet understood, low power noninvasive US + MBs provided excellent tumor growth control and may serve as a stand-alone clinical treatment. Furthermore, this anti-tumor effect is clearly dependent on the adaptive immune system, since US + MBs treatment in immunocompromised mice produced no benefit. A better understanding of this mechanism may identify adjunct treatments that would synergize with US and further enhance the anti-tumor effect demonstrated here.

**Reference**

1. Burke, C. W. et al. Ultrasound-activated agents comprised of 5FU-bearing nanoparticles bonded to microbubbles inhibit solid tumor growth and improve survival. Mol. Ther. 22, 321–8 (2014).

## A38 Stress response of cancer cells after low intensity Focused Ultrasound

### Karin Skalina^1^, Chandan Guha^2^

#### ^1^Albert Einstein College of Medicine, Bronx, New York, USA; ^2^Montefiore Medical Center, Bronx, New York, USA

**Objectives**

Low intensity Focused Ultrasound (LOFU) results in sublethal stress to tumor cells. We investigated the cell surface expression of immunomodulatory molecules in three murine cell lines (Lewis lung, breast and prostate adenocarcinoma). The immunomodulation was examined by flow cytometry of cell surface stress proteins: HSP70 & calreticulin, of immunomodulatory co-activating signals: CD40, CD80, CD86 and other receptors: MHC I, Fas). Surface expression of these molecules trigger activation of or phagocytosis by dendritic cells. Additionally, the activation of the unfolded protein response (indicated by phosphorylation of PERK or IRE1α or cleavage of ATF6α) can trigger apoptosis – a programmed cellular death pathway which can also activate the immune system. We hypothesize that by altering the total acoustic power of LOFU, we can modulate the stress signals both on the cell surface and within the cells.

**Methods**

LOFU treatment was performed on the Philips Therapy and Imaging Probe System (TIPS, Philips Research Briarcliff, USA) using 3 W or 5 W, 100 % duty cycle, 1.5 seconds, 1 mm spacing. LOFU treatment was performed on a cell pellet which were then replated and incubated at 37 °C, 5 % CO2 for the specified time. At the end of the incubation time, cells were lightly scraped for flow cytometry staining or lysis buffer was added to the adherent cells and then scraped for Western blot analysis.

**Results**

The analysis of murine lung and breast cancer indicates that there is a significant surface localization of both HSP70 and calreticulin as early as 4 hours after LOFU treatment and this localization diminishes by 24 hours post treatment. For murine prostate cancer cells, however, the surface localization of HSP70 increases at 4 hours after treatment and increases further at 24 hours. Additionally, there is a significant increase in surface localization of both HSP70 and calreticulin with a higher output power of 5 W compared to 3 W. For immunomodulatory co-activating signals, there is increased surface expression in Lewis lung carcinoma and prostate adenocarcinoma, but not breast carcinoma. Results of the activation of the unfolded protein response are still pending at the time of submission, but preliminary results indicate the both phosphorylation of PERK and IRE1α are increased following LOFU treatment.

**Conclusions**

Tumor stress response to LOFU treatment can be altered by the total acoustic power of LOFU treatment. The increased surface localization of HSP70, calreticulin, CD40, CD80 and CD86 have the capacity to augment the immune response to the tumor. Therefore, LOFU can be used in combination with ablative therapies in order to induce anti-tumor immunity and destroy the primary tumor.

## A39 The “abscopal” effect after USgHIFU treatment of advanced pancreatic cancer

### Franco Orsi, Guido Bonomo, Paolo Della Vigna, Giovanni Mauri, Gianluca Varano

#### Istituto Europeo di Oncologia, Milan, Italy

**Objectives**

Pancreatic cancer is considered one of the main big “killers” in Oncology, with still a very poor prognosis, both in patients amenable to resection and of course in more advanced stage disease. More than 50 % of patients are diagnosed with an advanced stage disease, where chemotherapy is usually the main therapy option. External radiotherapy is the most common palliative loco-regional treatment in pancreatic cancer, but in the last few years HIFU has been proposed as an alternative option for palliation in advanced stage.

From our specific experience of USgHIFU for pancreatic cancer, we report few cases of ABSCOPAL effect: where after a local treatment not only the target tumor but also the distant deposits will shrink

**Methods**

From 2008 until April 2016, 72 consecutive patients affected by pancreatic tumors were selected by a dedicated Tumor Board for receiving treatment with USgHIFU, for local tumor control and palliation. 64 % of patients were affected by pancreatic adenocarcinoma and 84 % of all the treated tumors were located at the level of pancreatic head. Majority of the patients had at least two lines of chemotherapy and 45 % of them received concurrent and/or consequent external RT. USgHIFU treatment was performed always during general anesthesia with GI catheter and abdominal compression with a dedicated water balloon in order to reduce the distance between the HIFU probe and the target and for push the bowel loop away from the acoustic treatment window. Patients were than evaluated by clinical and instrumental follow up.

**Results**

**Fig. 40 (abstract A39).**
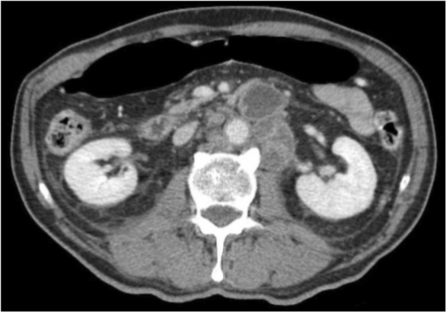
Multiple retroperitoneal lymphnodes in patient with pancreatic cancer

**Fig. 41 (abstract A39).**
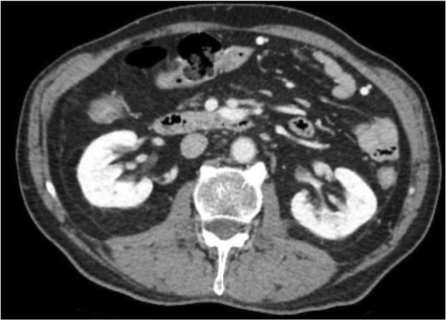
18 months after HIFU, CT shows the complete response at the level of metastatic lymphnodes

All the procedures were technically successful, with only one main complication due to a complete portal thrombosis developed 24 hrs after treatment. Objective response (OR) at MDCT, MRI and PET was 64 % with a disease control (DC) up to 90 % of all the patients. Pain was palliated in 23/27 of symptomatic patients. In four patients (4/46 with adenocarcinoma) the imaging follow up after the treatment revealed the shrinkage of metastases located in other sites: 2 retroperitoneal lymphnode (Figs. 40 and 41), 1 liver and 1 lung.

**Conclusions**

In advanced pancreatic carcinoma, HIFU can have a role in locally advanced unresectable disease. Based on our limited experience, HIFU could be also suggested in a metastatic setting (Stage IV), together with systemic therapy, with the potential benefit of being effective also outside the treated target (abscopal effect). There are few papers reporting the effect of HIFU as a booster of immune response in cancer patients, but there are still no data supporting the use of this non-invasive approach in advanced cancer patient just for this purpose. Specific dedicated clinical trial should be conducted for better understand the biological mechanism behind it.

## A40 Characterizing the immune response to boiling histotripsy ablation of renal carcinoma in the Eker rat

### George Schade, Yak-Nam Wang, Venu Pillarisetty, Joo Ha Hwang, Vera Khokhlova, Michael Bailey, Tatiana Khokhlova

#### University of Washington, Seattle, Washington, USA

**Objectives**

Boiling histotripsy (BH) is an experimental non-invasive Focused Ultrasound (US) technology. BH uses milliseconds-long US pulses at low duty cycle to mechanically homogenize targeted tissue. We aim to evaluate the adaptive immune response to BH ablation of spontaneous renal tumors in the Eker rat hereditary renal carcinoma (RCC) model.

**Methods**

Genotyped Eker rats (Tsc2 heterozygotes) were monitored for de novo RCCs with serial US until tumors were ≥8 mm. Syngeneic Wild-type (WT) (planned total n = 36) and Eker rats (planned total n = 48) were then randomly assigned to BH (n = 10 total to date) or an US SHAM procedure (n = 22 to date). BH was performed extracorporeally using US-guided small animal FUS system (VIFU-2000, Alpinion). The 1.5 MHz transducer was operated at duty cycle of 1 %, 10 ms pulses, 525-550 W electric power. Treatments targeted a ~0.5 cc area of the lower pole in WT rats and the largest RCC with a margin of normal kidney in the Eker rats. Blood samples were collected immediately before treatment and serially post-treatment. Rats were euthanized at 48 hrs, 7 days, 14 days, or 56 days following treatment. At euthanasia, ipsilateral and contralateral kidneys, hilar lymph nodes, and the spleen were collected. Intrarenal and circulating plasma cytokines were assessed using a 10 cytokine multiplex assay. Renal/tumor infiltrating leukocyte populations were assessed using immunohistochemistry. Leukocyte populations in tumor draining hilar lymph nodes and spleens were assessed with flow cytometry.

**Results**

**Fig. 42 (abstract A40).**
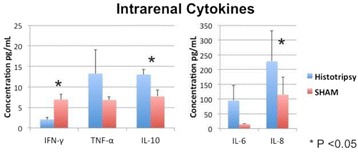
Intrarenal cytokine concentrations 48 hours after BH and SHAM treatment

**Fig. 43 (abstract A40).**
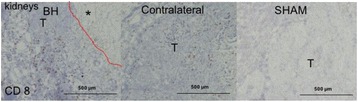
Immunohistochemistry appearance of CD8+ T-cells in BH treated (*) tumor (T) kidney, in the BH treated contralateral kidney, and in SHAM treated

BH treatment has been successful in all treated (n = 4 Eker, n = 6 WT to date) subjects, producing hypoechoic regions on US consistent with BH treatment effect. BH treatment was associated with significantly increased mean relative-plasma TNF-α vs. sham treatment at 0.25 (p = 0.02), 1 (p0.04), and 24 (p = 0.05) hours. At 48 hours, significant differences in intra-renal cytokines were observed in BH (n = 4) vs SHAM (n = 4) treated rats: IFN-gamma, IL-10, and IL-8 with trend towards differences in TNF-alpha and IL-6 (Fig. 42). On immunohistochemistry, BH treated Eker rats had increased CD8+ T-cells in both the treated and contralateral kidney compared to SHAM treated rats at 48 hours (Fig. 43). Additionally, increased infiltration of f4/80+ M1 macrophages into the treated kidney, was observed in 2/4 BH treated rats vs. SHAM at 48 hours. Longer-term data is pending at this time.

**Conclusions**

These data represent preliminary findings indicating differences in cytokines and tumor infiltrating leukocytes between BH treated and SHAM treated Eker rats up to 48 hours post-treatment. Assessment of longer-term immune effects of BH treatment and their significance is on-going and will be reported.

## A41 Use of shock-wave exposures for accelerating thermal ablation of localized tissue volumes

### Vera Khokhlova^1^, Ilya Sinilshchikov^2^, Petr Yuldashev^2^, Yulia Andriyakhina^2^, Wayne Kreider^1^, Adam Maxwell^1^, Tatiana Khokhlova^1^, Oleg Sapozhnikov^1^, Ari Partanen^3^

#### ^1^University of Washington, Seattle, Washington, USA; ^2^M.V. Lomonosov Moscow State University, Moscow, Russian Federation; ^3^Philips, Bethesda, Maryland, USA

**Objectives**

In High Intensity Focused Ultrasound (HIFU) applications, nonlinear acoustic effects can result in the formation of high-amplitude shock fronts in focal waveforms, with amplitudes exceeding 100 MPa. The presence of such shocks leads to increased tissue heating which can be beneficial for HIFU thermal therapy. The goal of this work was to evaluate the efficacy of different shock-wave exposures to accelerate thermal ablation of tissue volumes while enabling safer conditions for intervening tissues.

**Methods**

**Fig. 44 (abstract A41).**
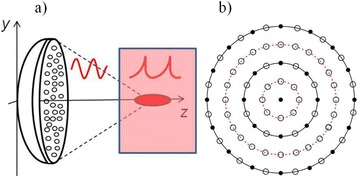
**a** Geometry of the numerical experiment: US beam is focused at the center of a 5 cm thick tissue layer. **b** Trajectory of the focus in the focal plane comprising 4 circles of 1, 2, 3, and 4 mm radii with 1 mm distance between the foci in each circle

Simulation studies were performed for a multi-element 1.2 MHz HIFU phased array of a clinical system (Fig. 44a, Sonalleve V1, Philips, Vantaa, Finland) for three different peak intensity levels at the array elements of 1.2, 8, and 15 W/cm2. A pulsing scheme was combined with discrete electronic steering of the array focus over a series of foci separated about 1 mm and arranged in 4 concentric circles (Fig. 44b). The circles were positioned in a plane at 25 mm depth in liver tissue of 50 mm thickness (Fig. 44a). The period between consecutive pulses was 20 ms. Pulse duration was varied to keep a constant time-average intensity at the array elements: 20 ms at 1.2 W/cm2, 3 ms at 8 W/cm2, and 1.6 ms at 15 W/cm2. The Westervelt equation combined with the bioheat equation was used for modeling temperature and thermal dose in tissue. Initial temperature in tissue was 20 °C, sonication was performed starting from the center and spiraling outward until a thermal dose of 1.76 s at 56 °C (equivalent to 240 CEM at 43 °C) was reached at each circle of the trajectory.

**Results**

The focal waveforms simulated in tissue for different peak intensities representing quasilinear sonication (1.2 W/cm2), sonication with a fully developed shock of 95 MPa at the focus (8 W/cm2), and sonication with higher focal shock amplitude of 118 MPa (15 W/cm2). Tissue ablation was achieved much faster at higher peak intensities with more clearly defined boundary between treated and untreated tissue. While temperature distributions and thermal dose contours in the focal plane were similar in shape for each trajectory and intensity level, corresponding distributions in the axial plane varied greatly. Slower heating at 1.2 W/cm2 resulted in strong heat diffusion in the axial direction and much larger ablation volumes. On the contrary, with rapid shock-wave heating, the ablated tissue volume followed the geometry of the targeted heat deposition. Although overall ablated volumes were smaller at higher peak intensities, the ablation rate was the highest at 15 W/cm2.

**Conclusions**

In comparison with conventional HIFU treatments, shock-wave exposures provide higher ablation rates at target sites with less heat diffusion to the surrounding tissues. Shock-wave heating regimes may therefore be clinically advantageous for accelerating thermal HIFU treatments, reducing the heating of surrounding tissues, and providing sharper margins of lesion volumes.

## A42 Effects of dosing and focal spacing in rapid ablation of large tissue volume using histotripsy with electronic focal steering

### Jonathan Lundt, Steven Allen, Jonathan Sukovich, Timothy Hall, Charles Cain, Zhen Xu

#### University of Michigan, Ann Arbor, Michigan, USA

**Objectives**

Current tumor ablation techniques, typically thermal-based, are either limited to treating tumors <3 cm in diameter (RFA and microwave) or are very slow (HIFU). Because histotripsy uses microsecond-length pulses separated by up to seconds of off-time for a given focus, it is possible to electronically steer the therapy focus of a phased array transducer to excite cavitation events throughout a large volume consisting of many foci during the off-time. We hypothesize that histotripsy combined with electronic focal steering can achieve rapid ablation of a large volume. This hypothesis was investigated in this paper, and the effects of number of pulses per focal location and focal spacing on tissue damage generated using histotripsy with electrical focal steering were studied.

**Methods**

**Fig. 45 (abstract A42).**
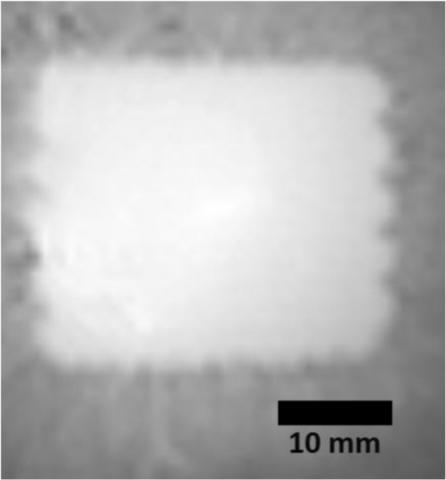
MRI of ex vivo sample following treatment. Scan plane orthogonal to transducer acoustic axis and positioned at geometric focus

**Fig. 46 (abstract A42).**
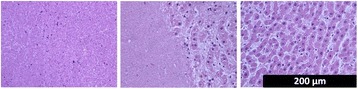
H&E histology. Left to right: inside lesion; lesion boundary; > 1 mm outside lesion boundary

Histotripsy was applied using a 250 kHz, 256-element hemispherical phased array transducer with a 15 cm focal distance, generating 1.5-cycle, 6-microsecond pulses. Each element produced ~0.5 MPa P- individually, but focal pressure produced by all elements could not be measured due to cavitation. To establish treatment parameters including pulse repetition frequency (PRF) and number of pulses (dose) to deliver, a single-focus lesion was generated in tissue-mimicking phantoms containing a thin layer of red blood cells (RBCs) and monitored by optical imaging. Based on these results, 35 *ex vivo* bovine hepatic tissue samples were treated by electronically scanning the therapy focus at 200 Hz over 1000 sites (or .2 Hz per focal site) (Fig. 45). To evaluate the dose required for complete tissue fractionation, dose was varied between 20-120 pulses per focus. Focal spacing was varied from 2.5, 3.15, and 3.5 mm in the lateral direction and held constant at 4.1 mm in the axial direction to assess the extent of overlap between adjacent foci required to achieve complete homogenization. Lesion size was assessed by gross morphology and Magnetic Resonance Imaging (MRI). The degree of tissue fractionation was examined by histology (Fig. 46). Surrounding tissue temperature was measured by thermocouples 1 cm from the lesion.

**Results**

RBC phantom results suggested that fractionation efficiency was optimal near 0.2 Hz and that 120 pulses were sufficient to achieve complete homogenization. Using lateral spacing ≥ 3.15 mm or < 100 pulses, unfractionated tissue structures were observed between foci. Using 2.5 mm lateral focal spacing and 120 pulses per focal location, complete, homogeneous tissue ablation of 43 +/- 6 mL region was achieved within 10 minutes, resulting in an ablation rate of 4.3 mL/min. A temperature increase of ~4 ° C was measured at the surrounding tissue. This work demonstrates that histotripsy combined with electronic focal steering can achieve rapid ablation of large volume at a rate more than double the rate of current available ablation techniques such as RFA.

**Conclusions**

Treatment of large and multiple tumor nodules remains a challenge for current tumor interventions, which are mostly thermal-based. This work demonstrates that histotripsy combined with electronic focal steering achieved homogenous and complete ablation of a large target volume at a rate greater than two-fold faster than microwave and RF ablation. Since histotripsy is non-thermal, the treatment should not be affected by the heat sink effect and is expected to remain effective and efficient even in highly vascular organs. With the capability of achieving rapid, homogenous cell disruption, histotripsy has the potential to substantially improve upon current tumor ablation methods.

## A43 The TRANS-FUSIMO system — towards a prototype for HIFU treatment of the liver under breathing motion

### Tobias Preusser^1,2^, Sabrina Haase^1^, Mario Bezzi^3^, Jürgen Jenne^4^, Thomas Langø^5^, Massimo Midiri^6^, Michael Mueller^7^, Giora Sat^8^, Christine Tanner^9^, Stephan Zangos^10^, Matthias Guenther^1^, Andreas Melzer^11^

#### ^1^Fraunhofer MEVIS, Bremen, Germany; ^2^Jacobs University Bremen, Bremen, Germany; ^3^Universita Degli Studi Di Roma La Sapienza, Rome, Italy; ^4^mediri GmbH, Heidelberg, Germany; ^5^Stiftelsen SINTEF, Trondheim, Norway; ^6^University of Palermo, Palermo, Italy; ^7^IBSmm Engineering spol. s r.o., Brno, Czech Republic; ^8^GE Medical Systems Israel Ltd., Tirat Carmel, Israel; ^9^ETH Zurich, Computer Vision Laboratory, Zurich, Switzerland; ^10^Johann Wolfgang Goethe-Universität, Frankfurt, Germany; ^11^University of Dundee, Dundee, United Kingdom

**Objectives**

The movement of the liver under breathing and its partial occlusion by the rib cage challenge the application of High Intensity Focused Ultrasound (HiFU/MRgFUS) for the thermal treatment of tumors. To explore the full potential of extracorporeal FUS to safely and precisely destroy tissue in the depth of the moving liver requires sophisticated technology. The European FUSIMO consortium (www.fusimo.eu) has shown in a proof of concept experiment that patient specific image processing, mathematical modeling of the organ’s motion, ultrasound propagation in the moving organ, and tissue heating is such enabling technology that empowers a physician to perform safe, effective and efficient ablation of tumors and to facilitate prediction of the outcome. In TRANS-FUSIMO (www.trans-fusimo.eu) the consortium is currently developing a prototype of a fully integrated system for the FUS treatment of the liver, which will be evaluated in an animal study and a patient study.

**Methods**

**Fig. 47 (abstract A43):**
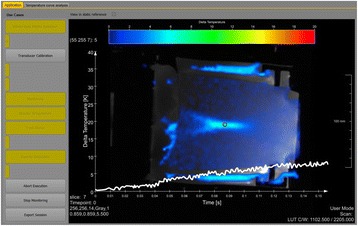
The screenshot shows the TRANS-FUSIMO treatment system during the execution of a sonication in a phantom

The TRANS-FUSIMO system comprises three sub-systems:

1. a planning software that incorporates image processing, motion modeling and prediction, as well as FUS simulations. This part of the system allows to plan and to assess the feasibility of the treatment;
2. a treatment system that performs the actual treatment control using previously evaluated treatment plans, as well as model based MR/US motion tracking and prediction and MR thermometry (Fig. 47);
3. a training system which acts as a treatment simulator that is based on real cases and the simulation of FUS propagation, tissue heating, and organ motion.

Thereby, the treatment system fully integrates with the GE MR scanner and the InSightec ExAblate Conformal Bone System (CBS). The control system and the model components have been validated in phantom and *ex vivo* experiments. Currently, the safety, efficacy and efficiency of the system are being evaluated in an *in vivo* animal study. Following, a two-arm study (neoadjuvant TRANS-FUSIMO MRgFUS + resection, TRANS-FUSIMO MRgFUS only) for human patients with metastases or HCC will show the feasibility of the TRANS-FUSIMO prototype for the clinical setting.

**Results**

The core of the TRANS-FUSIMO system comprises patient specific models for the motion of the liver under breathing, ultrasound propagation, and thermal damage. On the one hand the models are used for the planning of the treatment and in the training system. On the other hand the models are used to augment US/MR data during the treatment in order to achieve motion compensation and thermal monitoring.

**Conclusions**

The *ex vivo* validations show the safety and efficacy of the system under regulated motion of phantoms. Furthermore the validations show that the system fulfills all specification requirements. The currently running animal study will be the basis for a following two arm patient study.

## A44 Multifunctional robotic platform for USgFUS

### Arianna Menciassi, Selene Tognarelli, Andrea Cafarelli, Alessandro Diodato, Gastone Ciuti

#### Scuola Superiore Sant’Anna - The BioRobotics Institute, Pontedera, Italy

**Objectives**

Despite its promising results, FUS suffers from limited flexibility in therapy delivery, which narrows the applicability to non-moving and non-essential organs, mainly under Magnetic Resonance Imaging (MRI). We strongly believe that a robotic-assisted approach may offer the chance to overcome the current limitations of FUS by guaranteeing high robustness, flexibility/adaptability and precision of therapy. In this framework, the FUTURA (Focused Ultrasound Therapy Using Robotic Approaches) project (www.futuraproject.eu) is developing an autonomous, multi-functional and multi-robotic assisted platform able to perform non-invasive FUS on abdominal organs under US monitoring (USgFUS).

**Fig. 48 (abstract A44).**
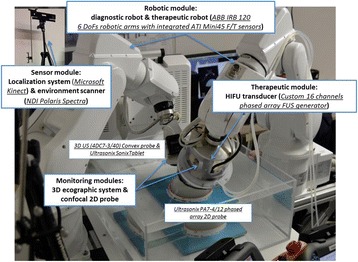
FUTURA platform

The FUTURA platform (Fig. 48) consist of: i) two six degrees of freedoms anthropomorphic manipulators, ii) a dedicated broadband 16 channels wave generator and a 16 channels annular array HIFU transducer; iii) two different US probes both connected to an acquisition system (SonixTablet, Analogic Ultrasound). The control architecture is implemented by using Robot Operating System software (ROS) with a purposely developed HMI.

**Methods**

The FUTURA architecture allows to maximize the flexibility of the procedure both for organs targeting and during the sonication procedure and lesion assessment. In addition, the FUTURA robotic integrated approach allows for safe interaction and cooperation between robots, patient and medical staff by using environmental reconstruction sensors.

In order to demonstrate the unique features of the FUTURA platform, several tests have been performed in *in vitro* and *ex vivo* static conditions by using a home-made tissue-mimicking phantom made up by a bulk of agar gel with internal cylinders (representing the targets of the therapy), based on polyacrylamide gel (PAA) mixed with egg white and *ex vivo* material (i.e. porcine liver or breast chicken instead of PAA cylinders into an Agar structure).

**Fig. 49 (abstract A44).**
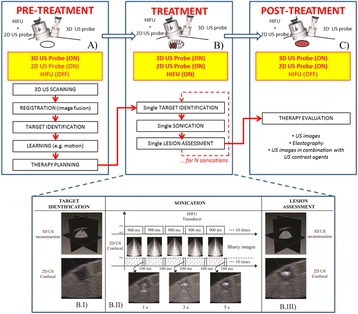
Workflow of the robotic-assisted FUS procedure that includes: **a** Pre-Treatment; **b** Treatment and **c** Post-Treatment phases

The complete workflow of the FUTURA procedure is reported in Fig. 49; more specifically, the effective use of two US imaging probes during different phases of the lesion treatment has been carried out. The sonication is composed by 10 repetitions of 1 second with 90 % duty cycle. The value of frequency and acoustic power of the sonication are 1200 kHz and 120 Watts, respectively.

**Results**

**Fig. 50 (abstract A44).**
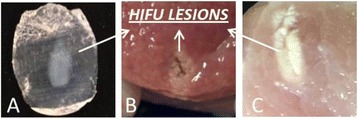
Optical images of the lesion induced by the HIFU transducer during the sonication phase into a tissue-mimicking phantom (**a**) and into ex-vivo tissues - porcine liver (**b**) and breast chicken (**c**)

**Fig. 51 (abstract A44).**
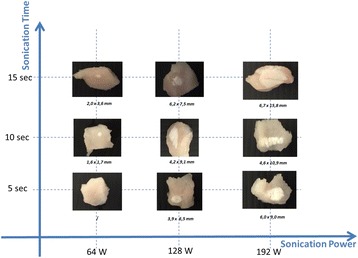
Optical images and typical dimensions of the lesions induced by the HIFU transducer into breast chicken at different output power and duration of exposure. In the first image, sonication power of 64 W for 5 sec, the lesion is imperceptible

During the target identification phase, both the US imaging probes are used. Although the US confocal probe is rigidly attached to the HIFU transducer, the FUTURA platform maintains US imaging flexibility thanks to the 3D US probe mounted on the second manipulator. The 3D US phantom reconstruction and the related 2D US confocal images acquired before sonication show the target without any lesion. In addition, the estimated HIFU transducer focus, located in the center of the target, assesses the correctness in positioning of the therapeutic manipulator (intersection of blue lines in pictures B.I-II-III). During the sonication process an on-line monitoring of the lesion is performed by exploiting a PWM signal for the HIFU transducer generator. Finally, the 3D US phantom reconstruction and the related 2D US confocal images acquired after sonication allow the lesion assessment (i.e., accuracy better than 1 mm). An optical assessment on the sectioned targets in proximity of the performed lesion was also carried out (Fig. 50). Finally, a specific analysis of the HIFU properties has been performed by observing the typical dimensions of the lesions induced by the transducer into the breast chicken tissue at different output power and duration of exposure (Fig. 51).

**Conclusions**

The most important features of FUTURA platform have been demonstrated. This work assesses the possibility to use a robotic platform for USgFUS treatment, using two US imaging probe for the different phases of the procedure. Synchronizing the HIFU transducer shot with the US imaging acquisition enables to monitor the lesion progress during the sonication phase, which is very important for patient safety. In addition, the use of both US probes during the sonication phase enables the tracking of moving 3D organs.

Future work will be carried out to assess the accuracy of USgFUS treatment under dynamic condition and a qualitative temperature estimation technique (e.g., by means of elastography) for the assessment of thermal effects will be integrated in the analysis.

## A45 Real time MR and US motion tracking for abdominal US/MRgFUS

### Sven Rothluebbers^1^, Julia Schwaab^2^, Jan Strehlow^1^, Senay Mihcin^3^, Christine Tanner^4^, Steffen Tretbar^5^, Tobias Preusser^1,6^, Matthias Guenther^1^, Jürgen Jenne^1^

#### ^1^Fraunhofer MEVIS, Bremen, Germany; ^2^mediri GmbH, Heidelberg, Baden-Württemberg, Germany; ^3^University of Dundee - TRANS-FUSIMO, Dundee, Scotland, United Kingdom; ^4^ETH Zurich, Computer Vision Laboratory, Zurich, Switzerland; ^5^Fraunhofer IBMT, St. Ingbert, Germany; ^6^Jacobs University Bremen, Bremen, Germany

**Objectives**

The ablation with HIFU/FUS would be a fantastic tool for liver tumor treatment and would lead to an invaluable benefit for patients suffering from malignant liver metastasis or hepatocellular carcinoma. However, FUS of liver has still not yet reached a significant clinical spread, particularly in the western world. There are two major challenges for FUS ablation of the liver: the rib cage and the permanent motion of the liver, e.g. due to respiration. Both, MRI as well as diagnostic US imaging are suitable to provide real-time information about the liver positon. For several years, we have been developing and improving US and MR based motion tracking for HIFU/FUS, e.g. currently in the European TRANS-FUSIMO project (www.trans-fusimo.eu). A further goal of the presented work is the automated detection of suited tracking features and the assessment of feature quality on MR and US image data.

**Methods**

US or MR image streams are analyzed in real-time to track liver motion. In a first step, pronounced structures, like the diaphragm, or landmarks/features like liver vessels are automatically detected or manually defined on 2D US or MR-EPI images. For motion tracking, a particle filter-based algorithm evaluates state hypotheses of local affine transformations to follow these features through the image stream. Data fidelity is assured by not only tracking each landmark separately, but also incorporating information about global motion. For each landmark, an estimate of the current position and the current uncertainty of that estimate is provided. Features with high uncertainty may then be ignored in following processing steps or replaced by more stable landmarks by the algorithm. For use in an application, the output of the algorithm is sent to the HIFU/FUS treatment unit, which can incorporate the data into further motion compensating components. This may either be established through direct beam steering of the FUS focus or by gating the therapy.

**Results**

**Fig. 52 (abstract A45).**
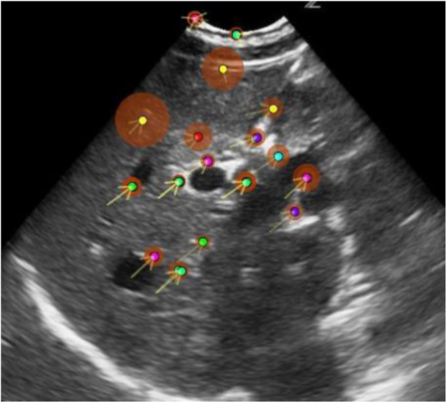
Liver motion tracking on US image. Arrows show the displacement of the feature related to a reference image due to liver motion. The red discs show a relative measure of positional uncertainty

**Fig. 53 (abstract A45).**
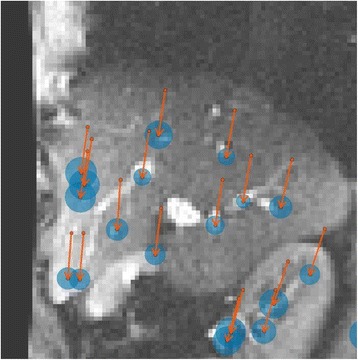
Liver motion tracking on MR-EPI images. The arrows show the displacement from the reference frame. Blue discs show the position uncertainty

US as well as MR based liver tracking was evaluated in different phantom experiments and on data from volunteers. Figures 52 and 53 show US and MR liver images with sample data provided by the algorithm. The arrows show the displacement of the feature related to a reference image due to liver motion. The discs indicate a relative measure of positional uncertainty. Automated feature detection, e.g. of blood vessels in EPI liver images, enabled fast and reliable definition of tracking features. Automated tracking quality assessment allowed a reliable detection of poor quality features which were replaced to allow a stable and reliable tracking over longer time periods. The mean tracking error on US liver images was 1.5 mm in 2D and 2.79 mm in 3D with a processing time of about 2 ms/frame. 1.7 mm was the mean error of MR based liver tracking with a computing time of 2 ms/frame and feature.

**Conclusions**

Quasi real-time liver motion tracking in 2D and 3D based on diagnostic ultrasound image streams as well as on ultra-fast MR image data is feasible, reliable and offers a sufficient precision for motion compensated HIFU/FUS therapy. Besides regular and irregular respiratory motion also liver drifts due to peristalsis and gravity on longer time scales can be compensated, since this technique is based on live image data. Integration of the tracking into existing clinical USgFUS and MRgFUS therapy units should be possible without great efforts. The presented tracking method is currently being integrated into an MRgFUS therapy setup for the treatment of liver tumors within the TRANS-FUSIMO project. A first clinical trial is planned at the beginning of 2017.

## A46 Pancreatic tumor monitoring and treatment using Harmonic Motion Imaging for Focused Ultrasound (HMIFU) in a transgenic mouse model

### Thomas Payen^1^, Carmine Palermo^1^, Steve Sastra^1^, Hong Chen^2^, Yang Han^1^, Kenneth Olive^1^, Elisa Konofagou^1^

#### ^1^Columbia University, New York, New York, USA; ^2^Washington University in St. Louis, St. Louis, Missouri, USA

**Objectives**

Pancreatic ductal adenocarcinoma (PDA) is one of the deadliest cancers with the lowest prognosis due to late diagnosis. PDA is characterized by an unusually dense stroma limiting chemotherapy perfusion. Harmonic Motion Imaging (HMI) assesses tissue mechanical properties by inducing localized oscillation resulting from a periodic acoustic radiation force. The amplitude of the induced displacement is directly related to the underlying tissue stiffness. The sonication is kept short for imaging (HMI) without tissue damage or the duration is prolonged for simultaneous HMI and HIFU treatment (Harmonic Motion Imaging for Focused Ultrasound or HMIFU).

This study first aimed at using HMI for characterizing the development of pancreatic tumors as a function of size and fibrosis extent in the KPC genetically-engineered mouse model. Secondly, the tumors were treated using HMIFU. HMI measurements were then resumed to monitor the mechanical changes resulting from the treatment.

**Methods**

A 4.5-MHz Focused Ultrasound transducer (FUS) generated an amplitude-modulated beam resulting in harmonic tissue oscillations at its focus. Axial tissue displacement was estimated using 1D cross-correlation of RF signals acquired with a confocally aligned, 7.8-MHz diagnostic transducer (P12-5, ATL) using a plane-wave beam sequence at a framerate of 1 kHz. Imaging was performed with 0.2 s sonication for each scan position, when treatment required 60-s long sonications which were shown to generate lesion in this model according to previous work by our group. KPC mice were genetically-engineered to develop pancreatic tumors with pathophysiological and molecular features similar to those of human PDA. Pancreatic tumor growth was monitored for 15 days with HMI scans performed every two days. For the second part of the study, HMIFU was performed on 5-mm tumors. The success of the treatment was assessed by measuring both the tumor area and its elasticity up to 14 days.

**Results**

**Fig. 54 (abstract A46).**
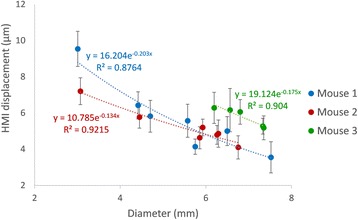
Pancreatic tumor development in 3 KPC mice over time during the early growth. The HMI displacement which is inversely related to the tissue stiffness is plotted against the diameter measured on Bmode images

**Fig. 55 (abstract A46).**
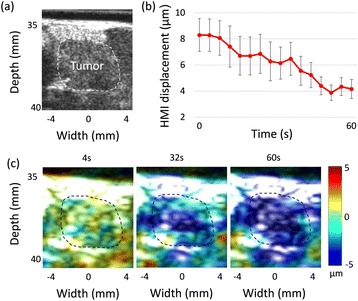
HMIFU results in a KPC tumor. The tumor is delineated on the high-resolution Bmode image (**a**). The variation of the mean HMI displacement in the tumor during ablation is monitored (**b**). Corresponding HMI maps with values relative to the baseline at t = 0 are shown for t = 4 s, 32 s and 60 s (**c**) showing the progressive stiffening of the ablated tissue

HMI demonstrated its capability to provide reproducible elasticity measurements in murine pancreatic tumors. Figure 54 shows that stiffening occurs progressively during pancreatic tumor growth from the very early stages. When plotting the HMI displacement against the tumor size, an exponential trend was fitted to the data with R2 > 0.87 in accordance to general models. When ablated with HMIFU for 60s, the tumor stiffened displaying a decrease in HMI displacement (Fig. 55). The lesion was confirmed by histology. The follow-up of the HMIFU treatment is performed with HMI to assess long-term tissue mechanical changes after treatment.

**Conclusions**

These results demonstrate the capability of HMI to provide elasticity measurements in the KPC murine pancreatic tumor model. The technique monitored the tumor growth as well as its stiffening when treated with HMIFU. Post-treatment mechanical changes could also be assessed with HMI. This study underlines the potential of HMI for monitoring tumor growth, treatment and follow-up changes in elasticity.

## A47 Preliminary investigation of integrating deployable reflectors and fluid lenses with endoluminal ultrasound to enhance and dynamically adjust focal gain and depth

### Matthew Adams^1^, Vasant Salgaonkar^2^, Serena Scott^2^, Graham Sommer^3^, Chris Diederich^4^

#### ^1^UCSF Thermal Therapy Research Group, San Francisco, California, USA; ^2^University of California, San Francisco, Sunnyvale, California, USA; ^3^Stanford University, Stanford, California, USA; ^4^University of California San Francisco, San Francisco, California, USA

**Objectives**

Endoluminal and endovascular catheter-based high-intensity ultrasound offer spatially-controlled thermal ablation in tissue targets adjacent to body lumens, such as the pancreas, liver, prostate, heart, etc. Due to the size constraints necessary for applicators to traverse through luminal passages, the integrated therapeutic transducers have minimal aperture size, limiting the depth of energy and thermal penetration typically to within a few centimeters beyond lumen borders. This study introduces concepts for a deployable applicator that features inflatable balloon reflectors and adjustable fluid-lenses to permit compact delivery and expansion at target sites (e.g. stomach, bladder, colon) to augment the effective therapeutic aperture for deeper and more selective heating generation. A preliminary theoretical analysis of potential designs, incorporating acoustic and thermal parametric studies, was performed along with benchtop proof-of-concept experiments as an initial investigation of the feasibility and capabilities of this design strategy.

**Methods**

**Fig. 56 (abstract A47).**
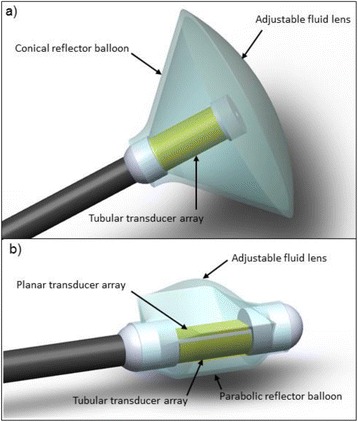
Endoluminal ultrasound applicators with deployable balloon reflectors and adjustable fluid lenses, in either **a**) end-firing or **b**) side-firing configurations

Two applicator designs, representing end-firing and side-firing configurations, were conceptualized and modeled, as shown in Fig. 56. The end-firing configuration consists of an array of tubular transducers surrounded by an expandable conical balloon that reflects acoustic energy into a fluid lens at the applicator’s distal tip. The side-firing configuration consists of an array of planar and tubular transducer segments surrounded by a parabolic reflector balloon that diverts energy through a fluid lens adjacent to the assembly. Acoustic simulations of the two assemblies were performed using a MATLAB implementation of the rectangular radiator method and incorporation of reflection/refraction of wave-fronts at material interfaces through the method of secondary sources. Thermal modeling, implemented in COMSOL, was used to generate resulting temperature distributions in homogenous and heterogenous tissue models. Parametric studies were performed to investigate performance of different potential lens fluids, and to evaluate acoustic focal gain and heating as functions of applicator/reflector/lens dimensions and geometries. Hydrophone beam plot measurements were made for a proof-of-concept end-firing applicator, consisting of a mounted tubular transducer centered in a conical brass reflector and covered by a perfluorocarbon fluid lens with adjustable radius-of-curvature (ROC).

**Results**

**Fig. 57 (abstract A47).**
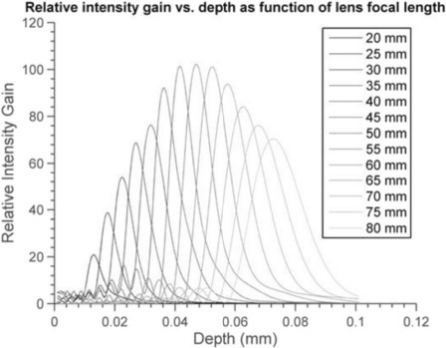
Intensity gain along the central longitudinal axis for the end-firing assembly as a function of lens focal length, as simulated in water. The 1.5 MHz tubular transducer was 8 mm OD x 20 mm height, leading to a 45 mm reflector/lens aperture diameter

**Fig. 58 (abstract A47).**
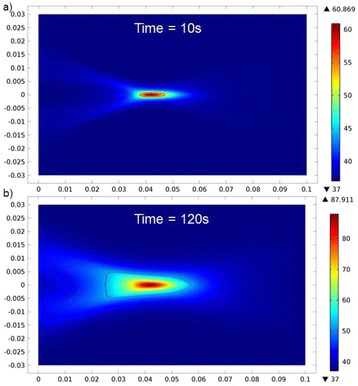
Simulated temperature distributions and 52 °C contour (black) in muscle tissue using the end-firing assembly, with specifications as in Fig. 57. Sonication duration was for (**a**) 10 s and (**b**) 120 s at 5 W/cm2 constant surface intensity input

**Fig. 59 (abstract A47).**
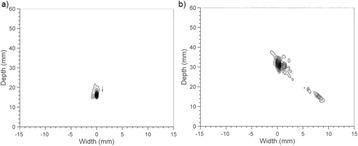
Hydrophone pressure-squared contours for the proof-of-concept end-firing assembly, showing tight focusing capabilities at two distinct lens focal lengths, **a** ~20 mm and **b** ~35 mm

Of the three potential lens fluids investigated (perfluorocarbon, silicone oil, and chloroform), perfluorocarbon offers the best acoustic transmission at lens focal lengths ranging from 0-100 mm, due to its greater speed-of-sound mismatch as compared to water or tissue. As demonstrated in Fig. 57, simulated achievable focal gain for either device configuration increases as a function of lens focal length to a maximum between ~20-50 mm, depending on the overall deployable reflector/lens size and tissue attenuation. Thermal simulations in homogenous muscle tissue with the end-firing configuration indicate capabilities of creating localized (~1 cm long X 5 mm wide) or volumetric (~3 cm long X 2 cm wide) ellipsoid thermal lesions at variable depths up to and beyond 5-6 cm in the tissue, as demonstrated in Fig. 58. Hydrophone beam plots of the preliminary proof-of-concept assembly illustrated capability of dynamically adjusting the focal plane depth between ~1-5 cm by adjusting the lens ROC, as shown in Fig. 59.

**Conclusions**

Preliminary theoretical and experimental investigations illustrate capabilities of using reflector and fluid lens assemblies coupled with endoluminal ultrasound to produce deeper and dynamically-variable acoustic focuses and thermal lesions.

## A48 Concurrent low dose Focused Ultrasound with chemotherapy in abdominal tumors

### Joan Vidal-Jove^1^, Eloi Perich^2^, Antonio Ruiz^2^, Manuela Velat^2^

#### ^1^Hospital University Mutua Terrassa, Barcelona, Spain; ^2^Institut Khuab Barcelona, Barcelona, Spain

**Objectives**

Systemic therapies for advanced stage IV abdominal tumors as standard chemotherapy regimens usually lack efficacy, and often this is substantially reduced by means of toxicity. Hyperthermic therapies have demonstrated efficacy alone by themselves and as well as concurrent treatments along with chemotherapy and/or radiotherapy. Focused ultrasound has been increasingly used as a method to enhance drug delivery. Several previous experiences have used this approach to improve tumor control in different types of tumors and with different chemotherapy drugs.

**Methods**

This is a feasibility study to increase delivery of the chemotherapy drug to the tumor. We have employed Ultrasound guided High Intensity Focused Ultrasound (USgFUS) along with concurrent standard chemotherapy treatments in a clinical setting with abdominal tumors. We have included advanced stage IV liver metastases from colo-rectal tumors, advanced stage IV pancreatic tumors and advanced stage IV retroperitoneal sarcomas. All patients were previously treated with and failed at least to 2 chemotherapy lines. Patients should not be candidates for tumor ablation. Patients will receive 3 FUS treatments concurrent with their chemotherapy infusions. FUS therapies are delivered the day of the chemotherapy infusion, the day before and the day after the chemotherapy. FUS treatments are on the range of 100 Watts for 45 minutes average. The objective is to cover maximum volumetric treatment.

**Results**

We measure performance status, complications rate, quality of life, symptoms control, pain control, and time to progression. Blood samples for functional studies and tumor markers as well as immunological studies: lymphocyte profile, immunoglobulines, complement, CRP were taken at daily basis during FUS treatments. MRI images were obtained previously and immediate after FUS treatments.

**Conclusions**

Study is in progress. We will report updated results at the meeting.

## A49 Clinical experience of intra-operative High Intensity Focused Ultrasound in patients with colorectal liver metastases. Results of a Phase II study

### David Melodelima^1^, Aurelien Dupre^2^, Jeremy Vincenot^1^, Chen Yao^2^, David Perol^2^, Michel Rivoire^2^

#### ^1^LabTAU - INSERM U1032, Lyon, France; ^2^Centre Leon Berard, Lyon, France

**Objectives**

Surgical resection is the only curative option for colorectal liver metastases (CLM), however, only 10 to 20 % of patients are suitable for surgery. High intensity focused ultrasound (HIFU) has been proven effective in a wide range of clinical applications. The liver is a particularly challenging organ for HIFU treatment due to the combined effect of respiratory-induced liver motion and partial blocking by the rib cage. A HIFU device based on toroidal transducers has been developed to enable the destruction of large liver volumes. Preliminary in vitro and in vivo studies demonstrated the feasibility, efficacy and safety of such HIFU ablations. This preclinical work is now translated into clinical practice. The aim of this study was to assess the feasibility, safety and accuracy of HIFU ablation in patients undergoing hepatectomy for CLM.

**Methods**

This study was a prospective, single-centre phase I/II study. The HIFU transducer has a toroidal shape and was divided into 32 concentric rings of equal surface (0.13 cm2). The diameter of the transducer and its radius of curvature were 70 mm. The operating frequency was 3 MHz. A 7.5 MHz ultrasound imaging probe was placed in the center of the device. The imaging plane was aligned with the HIFU acoustic axis. Nineteen patients were included in Phase I-IIa. In each patient two HIFU ablations were created. In step 1, ablations were centered in a target previously identified in ultrasound images. In step 2, ablations were created at distance from a target. Eight patients were included in Phase IIb and ten metastases (20 mm maximal diameter) were treated until now. HIFU ablations were created to ablate metastases with safety margins in all directions. The exposure time was adjusted from 40 seconds to 370 seconds according to the diameter of the metastases. For this first evaluation of the device, ablations were performed within the areas scheduled for resection. This study is registered with Clinical-Trials.gov (NCT01489787).

**Results**

The dimensions of ablations measured on ultrasound imaging were correlated (r = 0.88, p < 0.001) with dimensions measured on gross pathology. The average dimensions of HIFU ablations obtained in 40 seconds (Phase I) were a diameter of 21.0 ± 3.9 mm and a long axis of 27.5 ± 6.0 mm. The phase IIa study showed both that the area of ablation could be precisely created in the liver with a precision of 1-2 mm. It was possible to target about 90 % of the liver and all major anatomical structures. In Phase IIb, one metastasis of 10 mm in diameter was ablated in 40 seconds with safety margins. Using electronic focusing nine metastases of 2 cm in diameter were ablated with safety margins (>3 mm in all directions) in 370 seconds. The dimensions of these HIFU ablations were a diameter of 48.0 ± 4.9 mm and a long axis of 51.0 ± 3.4 mm. No lesions occurred in surrounding tissues. Two patients had a post-operative complication (pneumonia with pleural effusion and a urinary tract infection) not related to the HIFU treatment. There were no significant changes in hemodynamic and respiratory parameters.

**Conclusions**

This HIFU device safely achieved large and fast volume of liver ablation, with a precision of one to two millimeters. HIFU ablations of metastases with a diameter of less than 2 cm were successfully created with safety margins of at least 3 mm in all directions. This study is the first clinical use of intra-operative HIFU in patients with CLM. Reducing the risk of potential important complications associated with an extracorporeal approach was among the reasons we developed an intra-operative device. An open procedure seems also more appropriate since 15-20 % of additional metastases are discovered intraoperatively. From these preliminary results, intra-operative use of a HIFU toroidal transducer appears feasible, safe and effective in ablating liver metastases.

## A50 [18 F]-FDG PET and contrast MRI for enhanced guidance of Focused Ultrasound ablation in a syngeneic orthotopic model of murine pancreatic adenocarcinoma

### Samantha Tucci^1^, Lisa Mahakian^1^, Brett Fite^1^, Elizabeth Ingham^1^, Sarah Tam^1^, Chang-il Hwang^2^, David Tuveson^2^, Katherine Ferrara^1^

#### ^1^University of California Davis, Davis, California, USA; ^2^Cold Spring Harbor Laboratory, Cold Spring Harbor, New York, USA

**Objectives**

Pancreatic ductal adenocarcinoma (PDA) is the most lethal cancer and there is a desperate need for new therapeutic strategies. Due to its characteristic hypovascularity, dense stromal architecture, rapid progression, and local invasiveness, conventional therapies fail to cure PAC. Magnetic Resonance guided Focused Ultrasound ablation (MRgFUS) offers a platform to build combination therapies due to its ability to debulk tumors, enhance delivery of chemotherapeutics, and stimulate an immune response. Here, the feasibility of MRgFUS is evaluated in a syngeneic orthotopic mouse model of PAC developed from the KrasLSL-G12D/+; Trp53LSL-R172H/+; Pdx-Cre (KPC) model 1. Further, [18 F]-FDG positron emission tomography (PET) is used to enhance guidance.

**Methods**

Murine mT4-2D cells (gift from the Tuveson laboratory, Cold Spring Harbor, NY) were injected orthotopically by a sterile laparotomy (n = 5, C57BL/6 female mice). Mice were treated with MRgFUS on day 16 post-implantation with an MR-compatible annular array (Imasonic SAS, 3 MHz center frequency, 4.7 Watt acoustic power, 5.6 MPa peak negative pressure, 0.5 x 0.5 x 1.5 mm3 focal volume) and positioning system (Image Guided Therapy) in a Bruker BioSpec 7 T. Tumors were ablated with a diameter of 2 mm and scanning speed of 1 revolution per second for ~40s. In a separate group (n = 6), on days 10 (n = 3 + 1 wild-type) and 14 (n = 3 + 2 wild-type), mice were injected with 200 uCi of [18 F]-FDG, rested for 30 minutes, and scanned with a Siemens Inveon DPET for 30 minutes. Following PET acquisition, T1w images were acquired post gadolinium contrast (TE/TR/FA = 11.7 ms/750 ms/180°, 4.3 x 4.3 cm2 FOV, 256 x 256 matrix, 17 slices). Mice were euthanized, organs of interest were harvested, and radioactivity was measured by Wizard 1470 Automatic Gamma Counter (PerkinElmer). Maximum intensity projections were registered with MR images and regions of interest (ROIs) were drawn on the tumors and normal pancreases using Siemens Inveon Research Workplace.

**Results**

**Fig. 60 (abstract A50).**
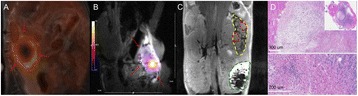
**a** [18 F]-FDG-PET overlaid on MR images **b** Real-time thermometry overlay acquired during MRgFUS ablation **c** T1w MRI confirms locally-invasive disease (red) in the spleen (yellow) **d** H&E confirms ablation, hemorrhage and immune infiltration

[18 F]-FDG-PET images enhanced the visualization of the tumor rim and demonstrated the heterogeneity of uptake within the tumor (Fig. 60a). In spite of the heterogeneity, ROI analysis indicated that primary pancreatic lesions (60.6 %ID/cc) exhibit a higher maximum uptake of [18 F]-FDG than the normal pancreas (17.8 %ID/cc) at day 14 of tumor progression (p < 0.05). Biodistribution confirmed these measurements, with 15.5 %ID/g and 16.9 %ID/g in the tumor-bearing pancreas at day 10 and day 14, respectively, as compared with 5.2 %ID/g in the normal pancreas (p < 0.5). Contrast enhanced T1w MRI facilitated visualization of lesions within both the pancreas and spleen (Fig. 60b-c). MRgFUS ablation was successfully completed under MR guidance (Fig. 60b), mice recovered well from treatment and the effect was confirmed on histopathology at 24 hours (Fig. 60d).

**Conclusions**

[18 F]-FDG facilitated visualization of the tumor rim. Feasibility of MRgFUS ablation of a syngeneic orthotopic murine model of PAC was established. Future studies will combine [18 F]-FDG-PET with MRgFUS to guide therapies and monitor disease progression.

## A51 Early experience with HIFU in the United States at Vituro Health, Sarasota

### Stephen Scionti

#### Scionti Prostate Center, Sarasota, Florida, USA

**Objectives**

Not released for publication

**Methods**

Not released for publication

**Results**

Not released for publication

**Conclusions**

Not released for publication

## A52 Investigation of the Therapeutic Effects of Pulsed Focused Ultrasound Combined with Encapsulated Chemotherapeutic Agents for Treatment of Prostate Cancer in vivo

### Lili Chen, Dusica Cvetkovic, Xiaoming Chen, Roohi Gupta, Bin Wang, Charlie Ma

#### Fox Chase Cancer Center, Philadelphia, Pennsylvania, USA

**Objectives**

To investigate the improvement of prostate cancer inhibition by combining pulsed Focused Ultrasound (pFUS) exposures and chemotherapeutic agents encapsulated nanodroplets under MR guidance for prostate cancer therapy.

**Methods**

Prostate cancer (LNCaP) cells were implanted orthotopically. First we developed nanodroplets encapsulated with chemotherapeutic agents for both paclitaxel (PTX) and docetaxel (DTX). Secondly, tumor–bearing mice were randomly divided into 5 groups (n = 5). Group 1 animals were treated with an i.v injection of DTX-encapsulated nanodroplets (DTX-ND) + pFUS. Group 2 were treated with pFUS alone. Group 3 were injected (i.v) with DTX-ND alone, Group 4 received free DTX and Group 5 was used as control. Ultrasound treatment parameters were 1 MHz, 25 W acoustic power, 10 % duty cycle and 60 seconds for each sonication. After treatment, animals were allowed to survive for 4 weeks. Tumor volumes were measured on MRI. Third, we repeated the experiment with PTX-ND. Finally we performed study on biodistribution of PTX-ND for prostate cancer and the treatment effects on tumor growth delay are being evaluated.

**Results**

With DTX-ND, significant tumor growth delay was observed in Group 1 with p = 0.039. There was no significant tumor growth delay observed for Group 2 (p = 0.477), Group 3 (p = 0.209) and Group 4 (p = 0.476). The results are consistent with earlier PTX-ND studies in which significant tumor growth delay was observed in Group 1 with p = 0.004. There was no significant tumor growth delay observed for Group 2 (p = 0.285), Group 3 (p = 0.452) and Group 4 (p = 0.158). The peak of drug update in tumor appeared at 4 h after injection in the biodistribution study.

**Conclusions**

Our results showed a great potential of targeted nanodroplets for prostate cancer therapy, which could be activated by pFUS. Our study also suggested that the optimal timing for applying pFUS is 4 h after i.v injection of PTX-ND and the treatment effect is being evaluated.

## A53 Monitoring histotripsy ablation with passive cavitation imaging

### Kenneth Bader^1^, Kevin Haworth^1^, Adam Maxwell^2^, Christy Holland^1^

#### ^1^University of Cincinnati, Cincinnati, Ohio, USA; ^2^University of Washington, Seattle, Washington, USA

**Objectives**

Histotripsy is a form of therapeutic Focused Ultrasound that mechanically ablates tissue. Pre-clinical studies have explored the potential for histotripsy to treat fetal septal defects, deep vein thrombosis, liver cancer, and benign prostatic hyperplasia (BPH). Histotripsy technology is also being tested in a clinical trial for the treatment of BPH. The bubble clouds generated by histotripsy are hyperechoic, enabling standard B-mode ultrasound imaging to be used for image guidance of tissue ablation. The mechanical oscillations of the bubbles within the cloud generate acoustic emissions. Information about the amplitude and location of these acoustic emissions would provide an additional means to monitor the treatment progress from histotripsy. Passive cavitation imaging (PCI) is an ultrasound imaging modality currently under development that spatially maps the power of acoustic emissions generated by cavitation. In this study, the ability of PCI and plane wave B-mode imaging to predict the location and spatial extent of histotripsy

**Methods**

**Fig. 61 (abstract A53).**
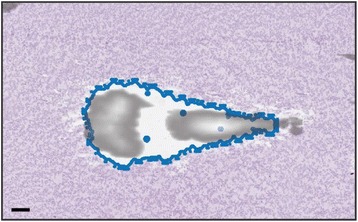
Comparison of B-mode image with the histotripsy ablation zone (outlined in blue). The bar in the lower right corner corresponds to 1 mm

**Fig. 62 (abstract A53).**
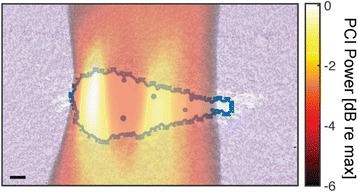
Comparison of passive cavitation image with the histotripsy ablation zone (outlined in blue). The bar in the lower right corner corresponds to 1 mm

Histotripsy pulses were generated in a prostate tissue phantom with a 1-MHz, 8 annular element array with a 10-cm aperture and 9-cm focal length. All elements of the transducer were simultaneously driven in parallel by a custom class D amplifier and matching network. Histotripsy pulses over a range of pulse durations (5-20 microseconds) and peak negative pressures (12-23 MPa) were explored in this study. During histotripsy insonation of the phantom, a 128 element linear array controlled with an ultrasound research scanner was used to monitor cavitation activity with both PCI and plane wave B-mode imaging (Figs. 61 and 62). After application of the histotripsy pulses, the phantom was sectioned and stained with a periodic acid-Schiff stain to delineate the ablation zone. The passive cavitation images and plane wave B-mode images were co-registered with the histological images. A receiver operating characteristic curve was used to quantify the comparison between each imaging modality and the spatial extent of the ablation zone.

**Results**

The area under the receiver operating characteristic (AUROC) was significantly greater than 0.5 for both plane wave B-mode and PCI, indicating both approaches can be used to predict the spatial extent of the ablation zone. The AUROC and accuracy were significantly greater for PCI compared to plane wave B-mode imaging. Furthermore, the sensitivity for predicting lesion formation was a factor of 3 larger for PCI compared to plane wave B-mode imaging.

**Conclusions**

Overall, these results indicate PCI provides an improved prediction of lesion formation from histotripsy.

## A54 Real time Tissue Changes Monitoring during the treatment of prostate cancer with HIFU

### Narendra Sanghvi^1^, Roy Carlson^1^, Wohsing Chen^2^

#### ^1^SonaCare Medical, LLC, Indianapolis, Indiana, USA; ^2^Ulthera, Inc., Mesa, Arizona, USA

**Objectives**

Backscattered RF ultrasound signals have been used clinically in differentiating normal and infarcted heart condition. Similarly, backscattered signals from pre and post hifu site are significantly different in magnitudes that provide correlation on degree of tissue changes. This is a validation study of Tissue Change Monitoring (TCM) algorithm performed with real-time thermometry. TCM performs spectral analysis of the RF backscattered (pulse-echo) ultrasound signals acquired before and immediately following the HIFU exposure. The RF signal analysis generates energy spectra of these signals and the difference of the energy magnitude is used as an estimator for tissue changes caused by the HIFU tissue ablation. TCM results are color coded and displayed on the each HIFU ablative site in Orange, Yellow and Green indicating tissue temperatures in the range from 75-100, 60-75 and 37-60 degrees C respectively.

**Methods**

Five (5) patients with histologically confirmed, organ confined prostate cancer were enrolled with approved protocol for this study. Four patients with focal cancer at stereotactic saturation biopsy had hemiablation only and one had a whole gland ablation. The Sonablate 500 HIFU device with Tissue Change Monitoring (TCM) software was used for the ablative treatment. Specifically designed 18 gauge needles containing three thermocouples per needle separated by a 1 cm distance were placed transperineally under TRUS guidance in the prostate. 4 or 5 needles were placed to cover the ablative sites. Temperatures from all thermocouples were recorded simultaneously at 0.5 seconds interval by a multi-channel thermometry system. Temperature data were acquired from a total of 56 sites,(26 focal zone sites, 22 posterior sites to the focal zone and 8 sites in the lateral gland of the prostate where there was no HIFU applied). Sixteen RF signals lines, pre and immediately post HIFU, for each ablative site were acquired at 50 MHz sampling rate. All RF data lines were processed by the SB-500 computer in the frequency domain to derive average energy spectra and change in magnitude for pre and post HIFU. The magnitude change and temperature from the site were used to derive cross correlation function and color code to overlay on the ablative site.

**Results**

The measured temperatures (Average, Max, and Min) in the HIFU treatment zones were 84, 114 and 70 degree C respectively. TCM energy readings were 1.05, 2.6 and 0.4 resulting in 83 % orange (75-100 degree C) and 17 % yellow (60-75 degree C) indicating an estimated average temperature of 91 degree C. Outside the focal zone, average recorded temperature was 50 degree C. The temperature recorded in the lateral lobe where no HIFU was applied was 40.7 degrees C.

**Conclusions**

The backscattered RF data analysis is capable of estimating tissue changes reliably during the HIFU procedures in real-time and can be used as an aid in the operating procedure. TCM was found highly sensitive to tissue motion. When the tissue reached boiling temperature, TCM output resulted in higher energy and was beyond the linear range of temperature monitoring from the RF backscattered signals.

## A55 Focal therapy with High Intensity Focused Ultrasound in low risk prostate cancer

### Christian Chaussy^1^, Stefan Thueroff^2^, Claudio Cesana^3^, Carlo Bellorofonte^3^

#### ^1^Caritas Hospital St. Josef, University of Regensburg, Strasslach-Dingharting, Germany; ^2^Klinikum Muenchen Harlaching, Munich, Germany; ^3^Clinica Columbus, Milano, Italy

**Objectives**

Prostate cancer (PCa) therapy undeniably exposes patients to a risk of impotence and incontinence. Therefore, low-risk localized prostate cancer is increasingly managed with watchful waiting or active surveillance, although this management bears some psychological burden and the risk of under staging and under grading. Alternatively, High Intensity Focused Ultrasound (HIFU) therapy allows non-invasive partial ablation of detected PCa in a single session (focal therapy). Aim of this study was to evaluate clinical efficacy and side effects of a single focal HIFU session in low risk PCa patients.

**Methods**

66 patients with localized prostate cancer who refused both definitive radical therapy and conservative management and had a strong desire to preserve potency were treated with focal HIFU. Men with strong obstructive symptoms, severe calcifications in the target area or severe prostatitis/abscess history were excluded. Treatments were performed with Ablatherm® intergrated imaging.

**Results**

53 % of the patients had positive staging biopsies on the right, 47 % on the left lobe. Initial PSA (PSAi = at diagnosis) was median 5.58 (1.02- 10.43) and increased to a PSA at treatment (PSAtr) of median 6.01 (0.2-11.4) within 4 months (period of first diagnosis until HIFU treatment), corresponding to a median pretherapeutic PSA velocity of 1,29 ng spinal anesthesia in median 50 (27-75) min with 16 french urethral catheter in place. Applied HIFU lesions were median 138 (86-338). There were no significant therapy related intra-/postoperative side effects within the follow-up period of median 4.4 (0.25-9.85) years, according to CLAVIEN Score . Based on patient interviews 3 months after HIFU treatment potency was: worse (4.5 %), equal (93.5 %); better (0 %). PDE-5 inhibitors were used by 74 % of patients. Urinary continence was preserved in all cases, 3 patients used a safety pad before and after treatment. There were no rectal disorder, fistulae or rectal incontinence. Post-HIFU catheter time was 22.7 % 5 days occurred. No late HIFU induced side effects were observed. PSA was reduced from median PSAtr (6.45) to a median Nadir of 1.45 ng/ml within median 12.5 weeks and showed a trend to relapse with a post-HIFU PSA velocity

**Conclusions**

Focal HIFU - a non invasive single session therapy performed under spinal anesthesia - demonstrated high potency- and continence preservation while treating diagnosed prostate cancer lesions. There were no significant therapy related intra-/postoperative side effects within the follow-up period. Focal HIFU results in a significant deceleration of PSA velocity from pre-HIFU 1.29 to post-HIFU 0.12 ng/ml/year by treating only 25 % of the prostatic volume. PCa diagnosis and consecutive watchful waiting and active surveillance bears an imminent psychological burden for the patient. The option of therapeutic efficacy, the wish to avoid therapeutic side effects and to preserve potency as well influences patients` decision for focal therapy with HIFU.

## A56 Factors affecting the efficacy of the Magnetic Resonance guided Focused Ultrasound ablation for painful bone metastases: results from a multicenter study in China

### Qingguo Wang^1^, Han Wang^1^, Shengping Wang^2^, Junhai Zhang^3^

#### ^1^Shanghai First People’s Hospital, Shanghai, China; ^2^Shanghai Cancer Center, Shanghai, China; ^3^Shanghai Huashan Hospital, Shanghai, China

**Objectives**

Bone is the third most common target organ that carcinomas metastasize to after lung and liver. Bone pain induced by cancer metastases contributes substantially to morbidity and mortality in patients with malignant carcinomas. Magnetic Resonance guided Focused Ultrasound (MRgFUS) is considered as one of the most promising therapeutic modality for tumor ablation. But, not all patients could obtain satisfied therapeutic efficacy. Some factors such as the number of bone metastasis, KPS and NPVR score can affects the results. The purpose of this non-randomized study was to evaluate the factors affecting the efficacy of the Magnetic Resonance guided Focused Ultrasound (MRgFUS) ablation for painful bone metastases treatment, and the safety and efficacy of this new non-invasive treatment modality.

**Methods**

One hundred and thirty one patients with painful bone metastases were screened for this study from June 2014 to September 2015. Seventy-one patients were finally enrolled in our study. All 71 patients underwent MRgFUS and none of them received radiotherapy or chemotherapy for pain palliation in the past two weeks. The Numerical Rating Scale for pain (NRS), the Brief Pain Inventory (BPI-QoL) score, the karnofsky performance scale (KPS), morphine equivalent daily intake dose (MEDID), and the adverse events (AEs) were respectively recorded before and 1-week, 1-month, 2-month, 3-month after the treatment.

**Results**

1) Seventy-one metastatic bone lesions in 71 patients were treated by MRgFUS and the treatment data was as follows: the mean treatment time was (83.94 ± 27.87) minutes, the mean sonication number was (12.74 ± 7.30) minutes. 2) Adverse events included: pain in therapy area in 3 patients and sciatica in 1 patient, which were obviously reduced after physiotherapy. 3) The NRS before treatment and at 1-week, 1-month, and 3-month after treatment was 6.70 ± 1.82, 4.37 ± 2.55, 3.96 ± 2.83, 3.70 ± 2.81 respectively. The NRS significantly decreased after treatment at all time points (P < 0.01). 4) The BPI-QoL score before treatment and at 1-week, 1-month, and 3-month after treatment respectively was 5.70 ± 2.09, 4.62 ± 2.36, 4.42 ± 2.60, and 4.42 ± 2.70. The BPI-QoL score significantly decreased after the treatment at all time points (P < 0.01). 5) After treatment, at 3-month, the mean ΔBPI-QoL score improvement in patients with solitary bone metastasis was statistically superior to that in patients with multiple bone metastases (P < 0.05). 6) At 1-week and 1-month, the mean ΔBPI-QoL score improvement in patients with osteogenic bone metastasis was statistically superior to other two groups (P < 0.05).

**Conclusions**

MRgFUS can be used as a non-invasive, safe, and effective method for treating painful bone metastases. The patients with solitary bone metastasis or osteogenic bone metastasis can achieve better therapeutic efficacy than those with multiple bone metastasis or osteolytic bone metastasis.

## A57 Can MRgFUS achieve local control of bone metastases?

### Alberto Bazzocchi^1^, Alessandro Napoli^2^

#### ^1^The “Rizzoli” Orthopaedic Institute, Bologna, Italy; ^2^Policlinico Umberto I, University of Rome – La Sapienza, Rome, Italy

**Objectives**

Magnetic Resonance Imaging guided Focused Ultrasound (MRgHIFU or MRgFUS) treatment of bone metastases is effective and safe for pain palliation. In a few circumstances, MRgFUS showed a partial or complete local control of the tumoral mass affecting bone. The aim of the work was to understand the efficacy of MRgFUS in terms of local tumor control of bone metastases and to explore this opportunity and the reasons for success or failure.

**Methods**

Patients with painful bone metastases were enrolled and submitted to MRgFUS (ExAblate 2100, InSightec Ltd, Israel), with imaging (CT/MRI) before and 3, 6 and 12 months after treatment. The primary endopoint was the number of lesions with partial or complete response at 3 months according to MD Anderson criteria.

**Results**

Out of 65 patients, 18 were lost and missed the 3-month imaging check. Forty-seven patients with 49 lesions were evaluated. The procedure was successful in terms of local control at 3 months in 24 lesions (49 %) – complete in 11, partial in 13. A stable disease was observed in 20 lesions and a progression in 5. Lesions with osteolytic (or mixed) pattern, with complete accessibility of the margins to the ultrasound beam are perfect candidate for ablation with the intent to control the lesion. Results were statistically independent of histology (primary cancer), previous radiation therapy (59 %), and size (up to 14 cm), though smaller lesions were generally more associated with complete or wide accessibility. Body mass index, age and sex of patients did not influence the outcome.

**Conclusions**

At present, MRgFUS for painful bone metastases may produce a local control of the lesion. In the future, patients with bone metastases should be selected for MRgFUS with three different intents, and the target should be clearly established before the treatment according to patient conditon, prognosis, symptoms and to the features of the lesion: 1) palliation of pain; 2) palliation and local tumor control; or 3) ablation / local control.

## A58 Effect of heating duration on therapeutic ratio in MR-HIFU hyperthermia mediated drug delivery using thermosensitive liposomes in rabbit Vx2 tumors

### Robert Staruch^1^, Chenchen Bing^2^, Sumbul Shaikh^2^, Joris Nofiele^2^, Debra Szczepanski^2^, Michelle Wodzak Staruch^3^, Noelle Williams^2^, Theodore Laetsch^2^, Rajiv Chopra^2^

#### ^1^Philips Research, Dallas, Texas, USA; ^2^University of Texas Southwestern Medical Center, Dallas, Texas, USA; ^3^University of Oklahoma Health Sciences Center, Oklahoma City, Oklahoma, USA

**Objectives**

Localized drug delivery using MR-HIFU hyperthermia to trigger the release of chemotherapeutic agents from thermosensitive liposomes (TSLs) has the potential to increase the therapeutic ratio between antitumor effect and systemic toxicity. For doxorubicin (DOX), which is active in many pediatric solid tumors, the late effects of cumulative dose to the heart are treatment-limiting. In this study, we investigate the effect of mild hyperthermia duration (10 vs. 40 minutes) on the ratio of DOX deposited in heated tumors compared to cardiac muscle, using a clinical formulation of TSL-DOX in a rabbit Vx2 tumor model.

**Methods**

**Fig. 63 (abstract A58).**
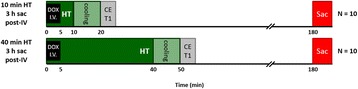
Experiment timeline. Rabbits infused with thermosensitive liposomal doxorubicin during 10 or 40 minutes of MR-HIFU hyperthermia. At 3 hours post injection, animals were sacrificed and tissues were harvested for doxorubicin quantification

Rabbits had Vx2 tumor cells injected into each thigh 12 days before therapy. For each rabbit, mild hyperthermia (10 or 40 minutes, 42 °C) was delivered to a 10 mm diameter region in one tumor using a clinical MR-HIFU system (Sonalleve V2, Philips Healthcare) incorporated into a 3 T MRI (Ingenia, Philips Healthcare). Feedback control of hyperthermia sonications was performed by modified software designed for mild hyperthermia, using MR thermometry data acquired in 6 slices every 3.2 seconds. During heating, TSL-DOX (Thermodox, Celsion Corporation) was administered intravenously at 2.5 mg/kg over 5-6 minutes. Rabbits were sacrificed and perfused with saline for tissue harvest 3 hours after the start of TSL-DOX infusion (Fig. 63). Tissue DOX concentrations were quantified using liquid chromatography-mass spectrometry against a daunorubicin standard following homogenization in cell lysis buffer and extraction in silver nitrate.

**Results**

Vx2 tumors treated with 10 vs. 40 minutes of mild hyperthermia (n = 10 per group) had largest dimensions of 24.3 and 29.9 mm, and body temperatures of 36.6 °C and 36.6 °C before sonication. Heating quality in the two groups was similar: target region temperatures averaged 41.9 and 41.8 °C, respectively, with T90 of 41.1 and 41.0 °C, and T10 of 42.7 and 42.7 °C. The average duration that target region voxels exceeded 40 °C in the 10 and 40 min heating groups was 11.3 vs. 40.7 min.

**Fig. 64 (abstract A58).**
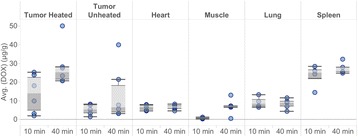
Doxorubicin biodistribution in rabbits following MR-HIFU hyperthermia mediated drug release

In non-targeted organs (heart, lung, spleen, muscle) DOX concentrations demonstrated no significant differences between the two heating groups (ANOVA with selected Bonferroni post-tests). This indicates that systemic toxicity does not increase with heating duration (Fig. 64).

**Fig. 65 (abstract A58).**
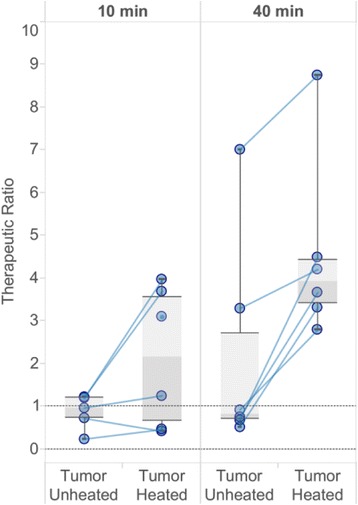
Therapeutic ratio of doxorubicin concentration in tumor/heart in rabbits that underwent 10 or 40 minutes of MR-HIFU hyperthermia

In both heated and unheated tumors, tumor DOX concentrations were higher for 40 vs. 10 min (25.4 vs. 14.0 μg/g and 6.7 vs. 5.4 μg/g, Fig. 65). Similarly, therapeutic ratio (DOX in tumor / DOX in heart) increased with heating duration (3.9 vs 2.2 times). Heated tumors had higher DOX concentrations and therapeutic ratios than unheated tumors (p = 0.03, 2-way ANOVA with Bonferroni). Therapeutic ratio and DOX concentration for heated tumors had a significant correlation with duration above 40 °C.

**Conclusions**

Our results confirm the theory that longer heating duration increases doxorubicin deposition in the targeted tumor without increasing doxorubicin accumulation in critical structures such as the heart. This will have an impact on hyperthermia protocols used in clinical trials of MR-HIFU hyperthermia mediated drug delivery.

## A59 Technical and imaging parameters predicting successful Magnetic Resonance guided Focused Ultrasound treatment of painful osseous metastases

### Pejman Ghanouni^1^, Jarrett Rosenberg^1^ Rachelle Bitton^1^, Alessandro Napoli^2^, Suzanne LeBlang^3^, Joshua Meyer^4^, Mark Hurwitz^5^, Kim Butts Pauly^1^

#### ^1^Stanford University, Stanford, California, USA; ^2^University of Rome, Rome, Italy; ^3^Focused Ultrasound Foundation, Charlottesville, Virginia, USA; ^4^Fox Chase Cancer Center, Philadelphia, Pennsylvania, USA; ^5^Jefferson University Hospitals, Philadelphia, Pennsylvania, USA

**Objectives**

**Fig. 66 (abstract A59).**
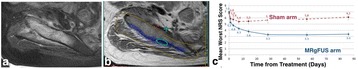
Axial MR images of the right iliac bone showing **a** T2 hyperintense permeative metastasis from lung cancer. The lesion had been treated with radiation and was still causing severe pain. **b** Multiple sonications (green oval) result in deposition of thermal dose (blue) along the targeted bone contour. **a** Change from baseline to 3 months in NRS score demonstrating a durable halving of pain scores in the treatment arm of the Phase III study

Magnetic Resonance guided Focused Ultrasound (MRgFUS) has proven to be effective in the treatment of painful osseous metastases. The results of the phase III randomized clinical trial comparing MRgFUS to sham treatment of bone metastases demonstrated a 64 % response rate in the treatment group, compared to a 20 % response rate in the sham arm (Fig. 66). We hypothesized that this intention to treat analysis included patients who were suboptimally treated. We reviewed images from the patient procedures to identify imaging features that indicated technically successful treatment, and assessed technical parameters for predictors of pain relief.

**Methods**

All images were anonymized such that the reviewer was blinded to the treatment outcome. Imaging features (size of tumor, osteolytic vs blastic tumor, location of tumor, T2 signal intensity, presence of edema around the tumor after treatment, devascularization of the periosteum around the tumor, tumor devascularization, etc.) and technical parameters of the treatments (number of sonications, total energy, average energy, sonication time, type of anesthesia etc.) were documented for each patient. After de-anonymization, imaging features and technical parameters were then correlated with successful pain relief (defined as at least a 2 point decline in pain score without significant increase in analgesic medication).

**Results**

**Fig. 67 (abstract A59).**
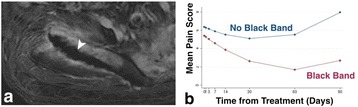
**a** Subtracted contrast enhanced image of the same patient as in Fig. 66 shows lack of perfusion of the treated bone surface (black band), which is an immediate post-procedural indicator of successful ablation of the periosteal nerves and **b** predicts subsequent pain relief. In this case in a), some of the cancer within the bone was also treated

We identified an imaging feature that we call the black band (BB), which represents devascularization of the periosteum around the tumor, as best seen on subtracted contrast enhanced T1 weighted fat saturated images obtained immediately after treatment (Fig. 67). The presence of the black band correlated with response to treatment (Odds Ratio of Complete or Partial Response with BB = 5.97; 95 % CI: 1.08 – 33.0; p = 0.041; sensitivity: 96 %, 95 % CI: 88-99 %; specificity: 22 %, 95 % CI: 9 – 40 %). The presence of the BB after treatment predicted a successful treatment (positive predictive value = 72 %, 95 % CI: 61 – 81 %).

We also identified a simple technical parameter, the Energy Density on Bone Surface (EDBS), which is the total energy delivered normalized by the bone surface area. EDBS was the only parameter that independently correlated with the likelihood of achieving complete or partial pain relief (OR of CR/PR with EDBS > 5 J/mm2 = 5.51; 95 % CI: 1.70 – 17.8; sensitivity = 57 %, 95 % CI: 44- 69 %; specificity = 81 %, 95 % CI: 61- 92 %). The black band was significantly correlated with high EDBS (EDBSmean with BB was 5.7 ± 2.8 J/mm2; without BB was 2.7 ± 0.9 J/mm2, p < 0.001).

**Conclusions**

With a technically successful treatment (BB+, EDBS > 5), the positive clinical response rate was 86 %, higher than in the published report. Those sites that had previous experience using MRgFUS to treat bone metastases and that used deep anesthesia used the highest mean EDBS (6.8 ± 3.1 J/mm2, p = 0.013), and also had the highest response rate (88 %, p = 0.009, Table 3). We also noted that the response rate (22 %) after suboptimal treatments (BB-, EDBS < 5) was the same as in the sham arm of the trial. Utilizing this information will help optimize MRgFUS treatment of osseous metastases.

**Table 3 (abstract A59).** Percentage of patients with complete or partial response

	Anesthesia		Total
	Not deep	Deep	
No prior experience	70 %	36 %	58 %
	(4/11)	(14/20)	(18/31)
Prior experience	48 %	88 %	73 %
	(12/25)	(36/41)	(48/66)
Total	58 %	77 %	68 %
	(26/45)	(40/52)	(66/97)

## A60 Technical aspects, feasibility, safety, and initial efficacy of osteoid osteoma ablation with MR-guided High Intensity Focused Ultrasound

### Ari Partanen^1^, Pavel Yarmolenko^2^, Ari Partanen^2^, Haydar Celik^2^, Avinash Eranki^3^, Viktoriya Beskin^3^, Domiciano Santos^3^, Janish Patel^3^, Matthew Oetgen^3^, AeRang Kim^3^, Peter Kim^3^, Karun Sharma^3^

#### ^1^Philips, Bethesda, Maryland, USA; ^2^Sheikh Zayed Institute for Pediatric Surgical Innovation, Children’s National Health System, Washington, DC, USA; ^3^Children’s National Health System, Washington, DC, USA

**Objectives**

Osteoid osteoma (OO) is a benign bone tumor that causes dull, but often debilitating pain, affecting mainly children and young adults. It is most commonly treated with computed tomography (CT)-guided radiofrequency ablation (RFA). However, RFA is invasive and CT guidance requires exposure to ionizing radiation. These shortcomings may be addressed through the use of Magnetic Resonance Imaging-guided High Intensity Focused Ultrasound (MR-HIFU), which allows non-invasive thermal ablation under real-time MRI guidance, with high spatial precision and without ionizing radiation. MR-HIFU may be used to reduce or eliminate pain through destruction of periosteal nerves and the OO nidus, without need for incisions or drilling. In an ongoing Phase I clinical trial, eight of the planned total of 12 children with symptomatic OOs have been treated at the Children’s National Medical Center (Washington D.C.). Herein we report on the technical aspects, feasibility, safety, and preliminary efficacy of OO MR-HIFU therapy.

**Methods**

Treatments were performed using the Sonalleve V2 clinical MR-HIFU system (Philips, Vantaa, Finland). The system includes a 256-element phased-array transducer, a positioning system with 5 degrees of freedom, and integrated MRI receive coils. Prior to therapy, patients were anesthetized or sedated, and positioned on the HIFU tabletop using gel pads, ultrasound gel, and degassed water for acoustic coupling. T2w and T1w MR images were acquired as a 3D stack, and subsequently used for treatment planning. Sonications of 16-48 s duration were performed at a frequency of 1.2 MHz, targeting regions 4-12 mm in diameter with acoustic powers of 20-160 W. During sonications, real-time MRI temperature maps were acquired using a fast-field-echo pulse sequence and the proton resonance frequency shift method (3 orthogonal slices centered on the target region and an additional slice in the near field). Post-therapy, T1w contrast-enhanced MR images were acquired to report on ablated regions. While complete follow-up is still pending, we assess technical aspects of OO MR-HIFU therapy. Specifically, the following were evaluated: patient positioning, sonication parameters, tissue temperature and thermal dose, tissue perfusion changes, treatment times, and other technical data.

**Results**

Patient positioning on the HIFU table was feasible in all eight cases, with some modification to the shape of standard ultrasound standoff pads to provide acoustic coupling to extremities. Lesion locations included the tibia (n = 3), femur (n = 3), talus (n = 1), and the great toe (n = 1). Distance between OO nidus center and skin ranged 1.1-7.7 cm, with estimated 43 ± 16 % power remaining at the bone surface due to soft tissue attenuation. Average acoustic power was 50 ± 30 W per sonication and total energy deposition per therapy session was on average 10 ± 7 kJ, resulting in complete lack of MR contrast enhancement of the OO nidus on T1w-imaging in six of eight patients, and partial enhancement in two patients. Immediately after the treatment, nonperfused volume extended 7 ± 4 mm into the bone and 4 ± 6 mm into adjacent soft tissue. For seven of the eight patients, complete or nearly complete resolution of OO-related pain followed within days and all patients were able to cease using medication 28 days following treatment. Sonications, including cool-down times, took 30-77 min, with 101-195 min of MRI suite time required. The treatments required less than 4 hours of total anesthesia and/or sedation and they were well-tolerated without any serious adverse events.

**Conclusions**

With complete symptom resolution in all patients evaluated thus far, MR-HIFU has the potential to offer a fast, safe, completely noninvasive, and radiation-free treatment option for children with painful OO. Sonication parameters were chosen conservatively in this initial clinical trial, and overall treatment quality may be improved and its duration reduced through optimization of sonication power, duration, and frequency, as well as improved, or more specialized MRI equipment.

## A61 Bone thermal modelling for MRgFUS of osteoid osteoma — validation with human clinical data

### Alexander Chisholm^1^, James Drake^2^, Dionne Aleman^1^, Adam Waspe^2^, Thomas Looi^3^, Samuel Pichardo^4^

#### ^1^University of Toronto, Toronto, Ontario, Canada; ^2^Hospital for Sick Children, Toronto, Ontario, Canada; ^3^Centre for Image Guided Innovation and Therapeutic Intervention, Toronto, Ontario, Canada; ^4^Thunder Bay Regional Research Institute, Thunder Bay, Ontario, Canada

**Objectives**

MR-guided High Intensity Focused Ultrasound (MRgFUS) has recently been applied to patients with bone lesions such as osteoid osteomas (OO), which are painful benign tumours. While initial results are positive, there is much uncertainty about the ideal target location, effective thermal dose, overall bone heating and possible inadvertent injury. Simulation of MRgFUS heat transfer within and around bone can be used as a pre-treatment planning tool that could optimize and improve the safety and efficacy of this therapy.

**Methods**

To address the uncertainties of MRgFUS treatment, a computer simulator was developed. The simulator has three main components: 1) an automatic bone segmentation tool; 2) an acoustic propagation simulation to calculate the acoustic velocity distribution at the target; and 3) a heat distribution model, calculated by the Pennes Bioheat Equation, which simulates the temperature distribution at the target location. Concurrently, a clinical pilot study is being conducted, to treat OO patients with MRgFUS. The MR imaging and MRgFUS sonication data from the first four patients from this study was reprocessed with the simulator and the results were compared with the measured clinical thermal results.

**Results**

**Fig. 68 (abstract A61).**
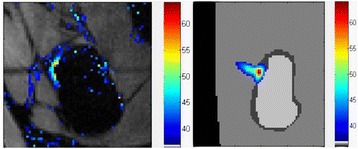
Comparison of MR thermometry (left) with simulated results (right)

**Fig. 69 (abstract A61).**
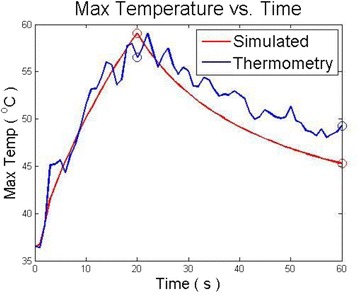
Max temperature vs. time of simulated and MR thermometry treatment data

Automatically segmented femurs were compared to the manually segmented ones. The average volume overlap (VO) and sensitivity (S) between the automatic and manual segmentations were 92.2 % and 96.3 %, respectively. The calculations for VO and S are as follows: VO = 1-(FP + FN)/Vm and S = TP/Vm, were FP is the number of false positives, FN is the number of false negatives, TP is the number of true positives and Vm is the manually segmented volume. The average maximum temperature difference between the simulation and clinical data at a region near the sonication focus, after 20 seconds of sonication, was 1.4oC; after 40 seconds of cool down time, the difference was 7.3oC. The max temperature is defined to be the average of the hottest 9 voxels. The average distance between ablation centroids was 2.9 mm. Figure 68 represents the clinical MR thermometry distribution (left) and the simulated temperature distribution (right) from a representative sonication in one patient. Figure 69 represents the max temperature comparison over 60 seconds for this sonication.

**Conclusions**

The calculated segmentation validation metrics suggests that the automatically segmented data is comparable to the manually segmented data. The average difference between simulated and thermometry data at peak temperature suggests that the simulator can accurately predict the extent of ablation near the focus during sonication. However, the average cool down temperature difference suggests the simulation is consistently under-predicting the temperature near the focus after 40 seconds of cool down. The distance between ablation centroids suggests that the simulator can predict the site of ablation to within 3 image voxels. A robust MRgFUS bone thermal simulation platform has the potential to significantly improve efficiency, safety and outcomes of patients with bone lesions.

## A62 Non-invasive therapy for osteoid osteoma: double site, long term, prospective development study with MR guided Focalized Ultrasound

### Alessandro Napoli^1^, Alberto Bazzocchi^2^, Roberto Scipione^3^

#### ^1^Policlinico Umberto I, University of Rome – La Sapienza, Rome, Italy; ^2^The “Rizzoli” Orthopaedic Institute, Bologna, Italy; ^3^University of Rome – La Sapienza, Rome, Italy

**Objectives**

Percutaneous or surgical therapy can lead to non-negligible side-effects and limited efficacy for paediatric population or young adults with a benign, self-limiting pathology such as osteoid osteoma. Over a period of 6- years we followed treated patients for at least 3-year for clinical, imaging and rehabilitation process. The aim of the study was to demonstrate that a completely non-invasive radiation-free focal ablation of osteoid lesions with MRgFUS can be a safe, effective and durable treatment option.

**Methods**

Patients were eligible for this dual-centre prospective development study if they had both clinical and radiological diagnosis of non-vertebral osteoid osteoma (typical nocturnal-worsening pain relieved with NSAIDs, pain score [VAS] >4, CT and MRI specific findings), and if they could safely undergo MRI, focalized US procedure and anesthetic. Patients received non-invasive therapy using MRI guided high-intensity Focused Ultrasound, delivered to all known osteoid lesions, identified on MRI, CT, or both. Feasibility, patient safety (adverse events), and clinical relevance profiles of MRgFUS were considered as primary outcomes; tumour control at imaging was considered as secondary outcome. Analyses were done on a per-protocol basis. This study is registered with ClinicalTrials.gov, number NCT02302651.

**Results**

Among 50 subjects recruited with both clinical and radiological diagnosis of OO, 45 underwent MRgFUS treatment and were included in the analysis. After intervention, no complications related to treatment or anaesthesia occurred and every patient leaved our hospital within 12-24 hours. Even during follow-up, no long-term complication related to treatment was reported. Clinical benefit was effective in all patients and overall VAS median value shifted from 8 (IQR 7-9) points before treatment to 0 at 1-week, 1-month, 6-month, 12-month, 24-month and 36-month follow-up. Similarly, VAS Scores median values for sleep disturbances, overall physical limitation, sport limitation and daily activities limitation dropped to 0 within the first month after treatment, and didn’t increase in subsequent controls. Quality of Life, assessed with FACT-BP Score, rose from a median value of 28 (22-34) before treatment to 55 at 1-week, 59 at 1-month, 60 at 6-month, 12-month, 24-month and 36-month follow-up. There was a complete response (a zero pain score one month after treatment, without recurrent symptoms, stable at follow up) in 80.00 % of cases. 4 patients required a second line of intervention. Overall, patients who reached and maintained a stable 0 VAS score during our 3-year observation with MRgFUS treatment alone were 86.67 %. At final

**Conclusions**

MRgFUS has shown complete absence of adverse events, persistent clinical efficacy and great tolerance profile. These strong features may induce a change in the current paradigm of OO management, making MRgFUS the treatment of choice, whilst CTgRFA role may shift to a second-line intervention.

## A63 MRI-guided High Intensity Focused Ultrasound treatment of osteoid osteoma in pediatric patients: preliminary results

### Michael Temple^1^, Adam Waspe^1^, Joao Guilherme Amaral^1^, Yuexi Huang^2^, Ruby Endre^2^, Maria Lamberti-Pasculli^1^, Joost de Ruiter^1^, Fiona Campbell^1^, Jennifer Stimec^1^, Samit Gupta^1^, Manoj Singh^1^, Charles Mougenot^3^, Sevan Hopyan^1^, Kullervo Hynynen^2^, Gregory Czarnota^2^, James Drake^1^

#### ^1^Hospital for Sick Children, Toronto, Ontario, Canada; ^2^Sunnybrook Health Sciences Center, Toronto, Ontario, Canada; ^3^Philips Healthcare Canada, Toronto, Ontario, Canada

**Objectives**

**Fig. 70 (abstract A63).**
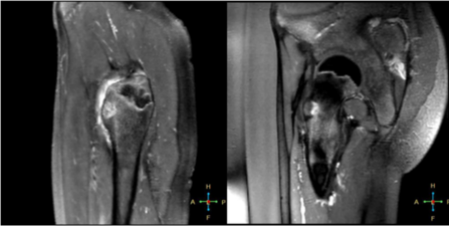
Representative pre and post HIFU sagittal MRI of an osteoid osteoma lesion with gadolinium enhancement. The lesion is fully enhanced before HIFU treatment, and shows non-perfused regions following treatment

Osteoid osteoma (OO) is the most common benign bone tumor in children. The vascularized, central nidus (Fig. 70) contains cells that produce pain-inducing prostaglandin hormones. While pain can be managed with non-steroidal anti-inflammatory drugs, many patients choose to undergo CT-guided thermal ablation using radiofrequency or laser, the standard-of-care for definitive treatment. Minimally invasive thermal ablation techniques include risks from ionizing radiation, fracture, infection and transmitted thermal damage along the needle. Non-target tissue injury is a concern as temperature cannot be measured with CT, and the treatment induces temperatures around 90 °C that are maintained for several minutes. Magnetic Resonance guided High Intensity Focused Ultrasound (MRgHIFU) is an alternative modality to successfully treat OO. There is no mechanical penetration of the bone, reducing the chance of pathologic fractures and reducing the potential for infection. We present our initial experience with OO MRgHIFU treatment in six pediatric pilot study patients.

**Methods**

**Fig. 71 (abstract A63).**
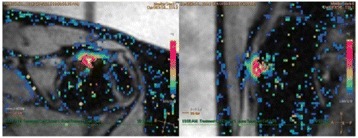
Representative MRI thermal imaging during HIFU treatment from a 30 W, 20s exposure (axial and sagittal views). Temperatures in the nidus reached >70 °C, indicating thermal ablation of the lesion

The study was performed to determine the impact of focused ultrasound treatment on bone pain, medication usage and health-related quality of life (HRQL). Standardized metrics, such as physical, emotional, social, and school functioning were collected through the use of age-appropriate and validated surveys (Pediatric Ouch, and PedsQL^TM^). An initial planning MRI was performed to ensure lesion accessibility/patient eligibility. MR thermometry (Fig. 71) was used during treatment to measure temperature in the target and surrounding tissue to ensure patient safety. Procedures were performed under general anesthesia or deep sedation. Post procedure, contrast enhanced MRI, to assess the non-perfused tissues that correspond to the ablated tissue volume (Fig. 70). Pain, HRQL and drug usage surveys were recorded on days 1, 7, 14, 30, 90 and 180 post treatment. Clinical visits on days 30, 90 and 180 included physical examination and diagnostic MRI of the target lesion.

**Results**

Between July 2014 and May 2016, 6 patients underwent 7 MR-HIFU procedures. Subject 2 underwent MR-HIFU twice due to an inadequate pain response after the first procedure. The average age of patients enrolled was 13 ± 4.1 years (range: 8 – 17) and weight was 52.2 ± 19.4 kg (range: 24 – 74). During this period, a total of 12 patients were referred for OO treatment. Reasons for exclusion from the study included acoustically inaccessible locations (proximity to nerve or growth plate) in 6 patients and patient refusal in 1. The overall technical success rate was 83 % (5/6 subjects). Subject 4 could not be treated due to poor acoustic access to the lesion. Complete response (treatment success) was seen in of 80 % (4/5) and partial response in 20 % (1/5) of the treated subjects. Pain scores dropped from an average of 7.8 prior to treatment to 1.2 by day 7, as shown in Table 4. Prior to treatment, the time to fall asleep was reported as 30-60 minutes. Following MR-HIFU, this decreased to 7-38 minutes. There were no significant adverse events.

**Conclusions**

This study shows that noninvasive MRgHIFU treatment of OO can be used to successfully treat osteoid osteoma in pediatric patients. When the cortex is intact, MR-HIFU heats the bone surface. Treatment of OO lesions results from heat conduction through bone from the cortical surface. In intact bone, MR-HIFU heats the cortical surface with deeper areas being heated by conduction. Subjects 1, 3 and 5 had superficial lesions and responded very well to MRgHIFU therapy. Subject 2 had a large, sclerotic medullary lesion that presented a challenge for heat conduction through the bone and required a second session with a more aggressive MR-HIFU treatment. Further research is necessary to define the optimal treatment parameters and limitations of MRgHIFU. A prospective registry is currently being designed to compare MRgHIFU with other types of thermal ablation.

**Table 4 (abstract A63).** Pain scores pre and post HIFU treatment (at days 1, 2, 7, 14, 30 and 90) for all treated patients. Patient 4 could not be treated, and exited the study. Only the data from the first treatment of Patient 2 was used

	Pre HIFU				Post HIFU				
Patient	Age	Weight	1 Day	Day 1	Day 2	Day 7	Day 14	Day 30	Day 90
HIFU-OO-001	16	60	8	1	1	0	0	0	0
HIFU-OO-002	17	64	5	3	2	6	5	4	7
HIFU-OO-003	8	24	7	4	0	0	0	0	0
HIFU-OO-004	17	74	N/A	N/A	N/A	N/A	N/A	N/A	N/A
HIFU-OO-005	10	58	10	6	5	0	0	0	0
HIFU-OO-006	10	33	9	2	3	0	1	0	Pending
Average	13	52.2	7.8	3.2	2.2	1.2	1.2	0.8	1.8
SD	4.1	19.4	1.9	1.9	1.9	2.7	2.2	1.8	3.5

## A64 Treatment of breast fibroadenoma with High Intensity Focused Ultrasound: update of feasibility study IDE #G130252

### David Brenin, Carrie Rochman

#### University of Virginia, Charlottesville, Virginia, USA

**Objectives**

Fibroadenomas are common in the United States and many patients find their fibroadenoma bothersome, and thus opt for surgical excision.

Study objectives are to evaluate the safety and feasibility of Ultrasound guided High Intensity Focused Ultrasound (USgHIFU) delivered by the Echopulse device (Theraclion, Paris) for treatment of breast fibroadenomas. General patient safety, cosmetic outcome, tumor response, patient experience, physician/operator experience, and device performance are being assessed.

**Methods**

Twenty female patients diagnosed with palpable breast fibroadenomas 1 cm or larger were enrolled in a single arm study and underwent treatment of their tumor utilizing a computer-driven, continuously cooled, extra-corporal HIFU probe mounted on an arm moved by motors, and guided in real-time with an integrated ultrasound imaging scanner. The integrated probe is positioned by the operator and the lesion is imaged. Treatment planning is automated and presented for review and approval on an integrated computer screen. Patients had tumors meeting the following criteria: Distance from the skin of ≤ 23 mm to the posterior border of the fibroadenoma, ≥ 5 mm from the anterior border of the fibroadenoma, and ≥ 11 mm from the focal point of the HIFU treatment. The chest wall must be more than 1 cm from the posterior margin of the tumor, and tumor volume must be between 0.3 cc and 10 cc.

Subjects are assessed immediately after treatment and at 3, 6, and 12 months.

**Results**

The study remains open to accrual, the following are PRELIMINARY RESULTS, thus the denominators vary by data point: Enrolment at the time of the writing of the abstract is16 patients. Six patients remain in follow-up. To date, there have been no grade 3 adverse events, nor any skin burns, persistent changes in skin appearance, nor other significant toxicities/morbidities observed in the patients treated. Mean patient-rated pain score during treatment on a scale from 0 to 100 was 17.8. One patient has reported persistent mild pain at 6 months follow-up (2 on a scale from 0 to 100). The most common toxicity observed was pain (reported by 9 of 16 patients). Preliminary patient satisfaction was 4.65 on a scale of 1-5 (5 = most satisfied), 10 of 11 patients reported they would undergo the procedure again, and 11/11 reported they would recommend the procedure to a friend or family member. Reduction in the size of the palpable mass was reported by both the patient and evaluating physician in almost all cases. Similar findings were found on ultrasound in the majority of cases. Cosmesis can be excellent, and unchanged from baseline in all cases to date.

**Conclusions**

To-date, USgHIFU, delivered by the Echopulse device for treatment of breast fibroadenomas in the IDE G130252 study has been well tolerated by patients, resulted in minimal toxicity, and appears to have been effective.

## A65 Long-term efficacy and tolerability of one or two US-guided HIFU treatment of breast fibroadenoma

### Roussanka Kovatcheva^1^, Jordan Vlahov^1^, Katja Zaletel^2^, Julian Stoinov^1^

#### ^1^University Hospital of Endocrinology, Sofia, Bulgaria; ^2^University Medical Centre Ljubljana, Ljubljana, Slovenia

**Objectives**

Breast fibroadenoma (FA) is the most prevalent benign tumor, accounting for up to 70 % of benign breast lesions [1,2]. They affect females in the reproductive period with two peaks of incidence in the third and in the fifth decade of life. During the follow-up, a minority of FA decrease in size or disappear, more than half of them remain unchanged, and some of them significantly increase [3].

Ultrasound (US)-guided high-intensity focused ultrasound (HIFU) is the only non-surgical and non-invasive procedure, where thermal destruction is achieved by precisely delivered energy to the target, without interrupting skin integrity. Recently, a multicentre study established that US-guided HIFU treatment of 51 FA resulted in 72.5 % volume reduction at 1 year [4].

The purpose of our study was to compare the long-term efficacy and tolerability of one or two HIFU treatments in patients with breast FA.

**Methods**

Twenty patients with 26 FA were selected for US-guided HIFU. The therapy was performed with the system EchoPulse (Theraclion, France) on an outpatient basis, in one or two sessions, under conscious sedation. FA volume was assessed before and followed up to 24 months after the last HIFU treatment. After each procedure, adverse events were evaluated. Written informed consent was acquired from all patients.

**Results**

In 19/26 FA (73.1 %) one HIFU was performed (group 1), whereas 7/26 FA (26.9 %) received second HIFU (group 2) 6-9 months (median, 7 months) after the first session. In group 1 and 2, FA volume decreased significantly at 1-month (p < 0.001) and 3-month follow-up (p = 0.005), respectively, and continued to reduce until 24-month follow-up (p < 0.001 and p = 0.003, respectively). At 24 months, mean volume reduction was 77.32 % in group 1 and 90.47 % in group 2 (p = 0.025). Mild subcutaneous oedema was observed in 4 patients and skin irritation in 3 patients.

**Conclusions**

US-guided HIFU represents a promising non-invasive method with sustainable FA volume reduction and patient’s tolerability. Although one treatment is highly efficient, the volume reduction can be increased with second treatment.

**References**

1. Olu-Eddo AN, Ugiagbe EE (2011) Benign breast lesions in an African population: A 25-year histopathological review of 1864 cases. Niger Med J 52:211-216

2. Aslam HM, Saleem S, Shaikh HA, Shahid N, Mughal A, Umah R (2013) Clinico- pathological profile of patients with breast diseases. Diagn Pathol 8:77

3. Dixon JM, Dobie V, Lamb J, Walsh JS, Chetty U (1996) Assessment of the acceptability of conservative management of fibroadenoma of the breast. Br J Surg 83:264-265

4. Kovatcheva R, Guglielmina JN, Abehsera M, Boulanger L, Laurent N, Poncelet E (2015) Ultrasound-guided high-intensity focused ultrasound treatment of breast fibroadenoma-a multicenter experience. J Ther Ultrasound 3:1

## A66 Harmonic motion imaging for characterization and Focused Ultrasound ablation monitoring of post-surgical human breast tumors

### Yang Han, Shutao Wang, Elisa Konofagou

#### Columbia University, New York, New York, USA

**Objectives**

Each year in the United States, over 60,000 women are diagnosed with breast cancer. High-Intensity Focused Ultrasound (HIFU) holds promise as a non-invasive and targeted therapeutic technique for breast cancer patients. To facilitate its widespread translation to the clinic, however, there is still a need for a real-time and cost-effective device that can reliably monitor HIFU ablation. Harmonic Motion Imaging for Focused Ultrasound (HMIFU) is an all-ultrasound radiation-force-based technique, which can be used for real-time HIFU ablation monitoring by tracking relative stiffness change at the treated area without interrupting HIFU.

**Methods**

Specimen collection and handling of post-surgical breast tissues were approved by the Institutional Review Board (IRB) board of Columbia University and informed consent was obtained from all enrolled patients. HMIFU was performed in 10 normal, 10 malignant tumor (invasive ductal carcinoma, namely IDC) and one benign tumor (fibroadenoma, namely FA) specimens. The HMIFU setup consists of a 93-element, 4.5-MHz HIFU transducer and a confocally-aligned 64-element, 2.5-MHz phased array imaging probe, which is connected to an ultrasound imaging research system. All HIFU elements were synchronously excited by a 25 Hz amplitude-modulated signal to vibrate the tissue at 50 Hz. A GPU-based fast image reconstruction method was used to monitor lesion development in real-time.

**Results**

**Fig. 72 (abstract A66).**
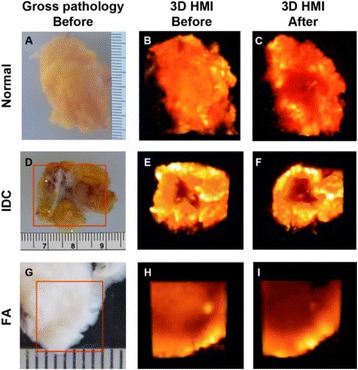
Gross pathology and 3D HMI displacement images of normal breast tissue (**a**-**c**), IDC (**d**-**f**) and FA (**g**-**i**) before and after HMIFU ablation. The brighter the color is indicates the higher HMI displacement and lower relative stiffness, and vice versa

For real-time monitoring, the displacement map and lesion map were streamed on the computer screen at a display frame rate of 2.4 Hz during treatment without interruption. Following a 2D raster scan, 3D HMI displacement maps were reconstructed representing the relative stiffness of the tissue (Fig. 72). The mean HMI displacement within the ROI decreased by 60 % from 24.73 ± 10.97 μm to 9.83 ± 6.46 μm in normal breast tissue (n = 10, p = 0.0048), decreased by 58 % from 12.77 ± 3.50 μm to 5.35 ± 2.74 μm (n = 10, p = 0.045) in IDC and decreased by 20 % from 2.56 μm to 2.06 μm (n = 1) in FA. There were statistically significant differences between before and after HMIFU ablation in both the normal and tumor specimens.

**Conclusions**

HMIFU is shown to be capable of differentiating relative stiffness between normal and abnormal breast tissues prior to treatment. HMIFU can also successfully monitor thermal lesions formation in breast tissue in real-time.

## A67 MRgFUS for desmoid tumors within the thigh: Early clinical experiences

### Matthew Bucknor, Viola Rieke

#### University of California San Francisco, San Francisco, California, USA

**Objectives**

Desmoid tumors are benign but locally aggressive tumors derived from fibroblasts. Surgery, chemotherapy, and radiation therapy have historically been the mainstay of treatment but recurrence is common and side effects can result in significant morbidity. Desmoid tumors of the thigh are particularly difficult to treat with conventional approaches and have a higher recurrence rate. MRgFUS has increasingly been used for management of desmoid tumors because of the favorable side effect profile. In this case series we highlight our experiences performing treatments in the thigh, including strategies for optimizing ablation size and safety.

**Methods**

Since December 2014, 14 MRgFUS treatments for desmoid tumors were performed at our institution in seven patients. 9 of these treatments were completed in three patients with large tumors within the posterior thigh. The first was a 7 year-old boy who had previously been treated with surgical resection, intraoperative radiation, along with courses of vinblastine/methotrexate and sorafenib. The second was a 21 year-old woman who had previously taken sulindac and celecoxib but had no other therapy. The third patient was a 14 year-old girl with no prior treatment. Treatment efficacy was evaluated by calculation of post-contrast ablation volume immediately following treatment and in diagnostic studies between treatments. Side effects and complications were also documented for each treatment.

**Results**

Case 1: Pre-treatment tumor volume 770 cc with 75 % non-enhancing volume following the initial treatment. However, first treatment was complicated by a third degree burn along the far-field skin. Despite the complication, the family and referring clinicians wished to proceed with additional treatments. Enhanced safety measures were implemented to protect the far-field skin including water bags and fiber-optic temperature probes, as well as interval skin checks. The patient had four subsequent treatments over 14 months, without complication, with non-perfused volume of 90 % most recently.

Case 2: Pretreatment tumor volume 740 cc. Notably, the sciatic nerve courses along the tumor’s anterior margin. Left lateral decubitus position was used to minimize the amount of energy through the sciatic nerve. Enhanced safety measures as above. First treatment resulted in a relatively low non-perfused volume of 30 %, likely related to a prominent fascial band. Second treatment resulted in 75-80 % ablation of the target.

Case 3: Pretreatment tumor volume was approximately 440 cc. The sciatic nerve was encased by the anteromedial portion of the mass. Left lateral decubitus position and enhanced safety measures again used. First treatment resulted in a relatively low non-perfused volume of 30 %, likely related to low energies. Second treatment resulted in 70-80 % ablation.

**Conclusions**

MRgFUS is an effective treatment for desmoid tumors with a favorable side effect profile, allowing for repeated treatments if necessary. Ablation size and safety can be improved by use of far-field coupling devices, careful patient positioning, and optimized sonication planning.

Consent to publish had been obtained from the patients and guardians.

## A68 Anatomical distribution of pediatric sarcoma and neuroblastoma: Targetability with MR-HIFU

### Jenny Shim^1,2^, Robert Staruch^2^, Korgun Koral^3^, Rajiv Chopra^3^, Theodore Laetsch^3^

#### ^1^Children’s Health, Decatur, Georgia, USA; ^2^Philips Research, Dallas, Texas, USA; ^3^University of Texas Southwestern Medical Center, Dallas, Texas, USA

**Objectives**

Not released for publication

**Methods**

Not released for publication

**Results**

Not released for publication

**Conclusions**

Not released for publication

## A69 A prospective study on the efficacy of single High Intensity Focused Ultrasound treatment of patients with benign symptomatic thyroid nodule

### Brian Lang^1^, Carlos Wong^1^, Heather Lam^2^

#### ^1^University of Hong Kong, Hong Kong, Hong Kong SAR; ^2^Theraclion Asia Pacific Limited, Hong Kong, Hong Kong SAR

**Objectives**

Benign thyroid nodules are prevalent among the general population and some nodules may exhibit growth over time leading to compression symptoms or cosmetic concerns. Although surgery remains the treatment of choice for symptomatic thyroid nodule, it is associated with a 2 %–10 % risk of complications and requires a general anesthesia. The aims of the present study were to assess the efficacy of a single treatment of High Intensity Focused Ultrasound (HIFU) in reducing benign thyroid nodule volume and to evaluate the changes in health-related quality of life (HRQL) following a single HIFU treatment.

**Methods**

After obtaining IRB approval, consecutive patients with symptomatic thyroid nodule were assessed for eligibility. Inclusions were nodule(s): 1) without signs of malignancy (i.e. no suspicious clinical and ultrasonic features and benign cytology on fine needle aspiration); 2) measuring ≥10 mm on ultrasound (USG) in three orthogonal dimensions; and 3) menable to HIFU. Exclusions were nodule(s): 1) measuring < 40 mm (by largest dimension); and 2) located <2 mm from trachea, esophagus or recurrent laryngeal nerve (where ablation might pose thermal injury). Eligible patients were offered a choice of HIFU treatment, active observation or surgical resection. HIFU treatment was conducted with the USG-guided Echopulse (Theraclion SA, France). Primary outcome was a change in index thyroid nodule volume 6 months after HIFU. To have 80 % power and 95 % confidence interval (two-sided) to detect minimal important difference of 20 %, 20 patients were needed. Assuming a 10 % incomplete and withdrawal rate, 22 patients were required. Thyroid volume (mL) was assessed at baseline (i.e. before ablation), 1-week, 3-month and 6-month while HRQL was assessed by the Chinese version of the SF-12v2 at baseline and 6-month. The HRQL measured by eight domain scores and two summary scores (physical and mental component summary) was then compared between those who received HIFU and those who chose active observation (controls) at 6-month.

**Results**

Over this period, 22 (52.4 %) chose to receive a single course of HIFU (HIFU group) while the other 20 patients chose to have active observation (controls). Among the HIFU group, the majority were females (90.9 %) and the majority (77.3 %) had their index nodule as the dominant nodule in a multinodular goiter. The mean base index nodule volume was 6.48 ± 4.34 mL (range: 0.92 - 16.76 mL). At 6-month following HIFU, the treated nodule volume decreased to 1.38 ± 1.31 mL (n = 22, p < 0.001). The average extent of nodule reduction was 71.58 ± 11.81 % (range: 54.45 – 90.09 %). However, there was no significant correlation between extent of 6-month volume reduction and basal volume (r = 0.096, p = 0.806), total treatment time (r = 0.503, p = 0.168), total energy delivered (r = 0.122, p = 0755) or mean delivered energy per treated volume tissue (r = 0.150, p = 0.700). Compared with controls, the HIFU group achieved a significantly greater improvement in four quality-of-life domains (17.57 ± 6.46, p = 0.009 for role physical; 18.01 ± 6.78, p = 0.011 for bodily pain; 24.77 ± 8.15, p = 0.004 for general health; 32.61 ± 8.93 for social functioning), and physical component summary of the SF-12v2 ((7.89 ± 2.83, p = 0.001).

**Conclusions**

USG-guided HIFU ablation is not only an effective and safe treatment option for patients with benign symptomatic thyroid nodules but has the potential of improving the HRQL of patients who do not wish to undergo surgical resection.

## A70 The effect of different treatment regimens with US-guided HIFU on thyroid nodule volume

### Roussanka Kovatcheva^1^, Jordan Vlahov^2^, Katja Zaletel^1^, Julian Stoinov^2^, Alexander Shinkov^3^

#### ^1^University Medical Centre Ljubljana, Ljubljana, Slovenia; ^2^University Hospital of Endocrinology, Sofia, Bulgaria; ^3^Medical University Sofia, Clinical Center of Endocrinology, Sofia, Bulgaria

**Objectives**

Thyroid nodules prevalence can reach 67 % on ultrasound (US) examination. Although 95 % of them are benign, 1/3 show continuous growth and should be treated because of clinical symptoms or cosmetic concerns (1). Surgery is still the main therapeutic strategy, in spite of the fact that it carries 2–10 % risk of complications (2).

US-guided high-intensity focused ultrasound (HIFU) is a non-invasive ablative technique, designed to decrease thyroid nodule size (3, 4). The purpose of our work was to compare the long-term efficacy and safety of one and two consecutive HIFU sessions for the ablation of benign solid thyroid nodules.

**Methods**

Twenty patients (mean age, 44.5 years) with euthyroid benign nodular goitre were treated with US-guided HIFU device (EchoPulse, Theraclion, France) under procedural sedation and analgesia. US volume measurement was performed at baseline, 3 and 12 months after the final treatment. Three months after the first treatment, 12 patients (group 1) continued the follow-up and 8 patients (group 2) received second HIFU treatment. Adverse events were evaluated after each HIFU procedure. Written informed consent was acquired from all patients.

**Results**

Concerning the baseline nodule volume and the energy applied per nodule volume, there was not significant difference between group 1 and group 2 (5.04 ± 2.70 ml and 4.83 ± 2.74 ml, respectively; 3.5 ± 1.4 kJ/mL and 4.1 ± 1.6 kJ/mL, respectively). At 12-month follow-up the mean nodule volume decreased significantly in both groups (2.35 ± 2.44 ml, p = 0.003, and 2.63 ± 1.85 ml, p = 0.017, respectively) with a maximal volume reduction of 95.4 % and 66 %, respectively. The mean percent of volume reduction at M3 after the first HIFU and at M12 after the final HIFU differed significantly between group 1 and 2 (47.4 % ± 20.8 vs 24.2 % ± 15.8, p = 0.02 at M3, and 55.5 % ± 28.4 vs 46 % ± 22, p = 0.011 at M12). In two patients, transient subcutaneous oedema and mild skin redness were registered after the first HIFU procedure and after the second procedure one patient developed transient Horner syndrom, which resolved in 6 months.

**Conclusions**

The long-term effect of one and two HIFU sessions in solid benign thyroid nodules is comparable. Larger studies are needed to explain the different thyroid nodule susceptibility to HIFU ablation.

**References**

1. Erdogan MF, Gursoy A, Erdogan G. Natural course of benign thyroid nodules in a moderately iodine-deficient area. Clin Endocrinol (Oxf) 2006;65(6):767-771.

2. Bergenfelz A, Jansson S, Kristoffersson A et al. Complications to thyroid surgery: results as reported in a database from a multicenter audit comprising 3,660 patients. Langenbecks Arch Surg 2008;393(5):667-673.

3. Esnault O, Franc B, Ménégaux F et al. High-intensity focused ultrasound ablation of thyroid nodules: first human feasibility study. Thyroid 2011;21(9):965-973.

4. Kovatcheva RD, Vlahov JD, Stoinov JI, Zaletel K. Benign Solid Thyroid Nodules: US-guided High-Intensity Focused Ultrasound Ablation-Initial Clinical Outcomes. Radiology, 2015, 276(2):597-605.

## A71 Prostate cancer HIFU — novelty or innovation?

### Jim Hu

#### Weill Cornell Medicine, New York, New York, USA

**Objectives**

Not released for publication

**Methods**

Not released for publication

**Results**

Not released for publication

**Conclusions**

Not released for publication

## A72 MR-HIFU ablation of osteoid osteoma: Experience in a pediatric tertiary care center

### Karun Sharma

#### Children’s National Health System, Washington, DC, USA

**Objectives**

To describe our experience with Magnetic Resonance-guided High Intensity Focused Ultrasound (MR-HIFU) ablation of painful Osteoid Osteoma (OO) in children.

**Methods**

Nine children with OO (7 M, 2 F; 16 ± 6 years) underwent MR-HIFU ablation through an FDA and IRB-approved safety and feasibility clinical trial using the Sonalleve V2 MR-HIFU system. Treatment feasibility, patient safety, and clinical response were evaluated in all patients over 28 days. Additional follow-up was performed to evaluate longer-term safety and durability of clinical response.

**Results**

MR-HIFU therapy was feasible in all nine patients without any serious treatment-related adverse events. Eight out of 9 patients reported complete response in terms of pain resolution and cessation of medication usage and one out of 9 patients reported partial response. All treatments were performed on an outpatient basis without overnight admission.

**Conclusions**

These findings show that MR-HIFU ablation of OO is feasible and safe in pediatric patients and offers clinical response rates similar to those seen with radiofrequency ablation, the standard of care therapy at most US hospitals. However, the completely noninvasive and radiation-free nature of MR-HIFU is advantageous over RFA, particularly in children and adolescents for whom collateral damage and radiation exposure may cause long-term morbidity.

## A73 Noninvasive thrombolysis using microtripsy in a porcine deep vein thrombosis model

### Xi Zhang, Jonathan Macoskey, Kimberly Ives, Gabe Owens, Hitinder Gurm, Jiaqi Shi, Matthew Pizzuto, Charles Cain, Zhen Xu

#### University of Michigan, Ann Arbor, Michigan, USA

**Objectives**

Histrotripsy is a novel therapeutic technique that uses ultrasound generated from outside the body to create controlled cavitation in a target tissue, and fractionates it into acellular debris. We have developed a new histotripsy approach, termed microtripsy, to improve targeting accuracy and to avoid collateral tissue damage. This *in vivo* study evaluates the efficacy and safety of microtripsy thrombolysis in a deep vein thrombosis (DVT) model.

**Methods**

Acute thrombi were formed in the left femoral veins of pigs (~35 kg) by occluding the vessel using two balloon catheters and infusing with thrombin. Guided by ultrasound imaging, microtripsy thrombolysis treatment was conducted in 14 pigs. 10 pigs were euthanized on the same day (acute) and 4 at 2 weeks (subacute). To evaluate the vessel damage, 30-min free-flow treatment (no thrombus) in the right femoral vein was also conducted in 8 acute pigs.

**Results**

**Fig. 73 (abstract A73).**
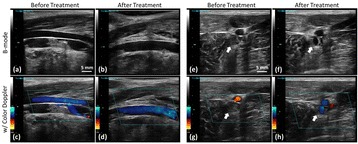
Representative US B-mode images and color Doppler images taken before and right after microtripsy thrombolysis treatment in an acute pig. **a**-**d**: Longitudinal sections of the femoral vein. **e**-**h**: Cross sections of the femoral veins

**Fig. 74 (abstract A73).**
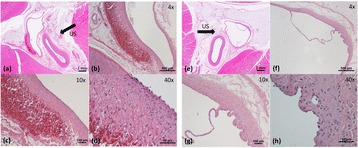
Histology of Femoral Veins. **a**-**d**: Right after a free-flow treatment. **e**-**h**: Two weeks after a thrombolysis treatment

Blood flow was restored or significantly increased after treatment in 13 out of the 14 pigs (Fig. 73). One treatment was not effective due to a technical issue with clot formation. The flow channels reopened by microtripsy had a diameter up to 64 % of the vessel diameter (~6 mm). The average treatment time was 16 minute per cm-long thrombus. Minor hemolysis was observed in both thrombolysis and free-flow treatments. Histology showed no vessel damage and only microscopic hemorrhage outside the veins for the free-flow treatments with nothing abnormal observed for the subacute treatments (Fig. 74).

**Conclusions**

Microtripsy is a safe and effective treatment for DVT in a porcine model. Further studies are warranted to study the role of this promising noninvasive thrombolytic method in human subjects.

## A74 Acute blood pressure response during MRgFUS renal denervation in a normotensive rat model

### Allison Payne, Christopher Dillon, Ivy Christofferson, Elaine Hilas, Jill Shea

#### University of Utah, Salt Lake City, Utah, USA

**Objectives**

Hypertension represents a critical health challenge for millions of people and despite the availability of numerous pharmaceutical agents, approximately 10 % of the patient population who are currently taking three or more medications continue to have high blood pressure and are identified with resistant hypertension. Renal sympathetic nerves are key in initiating and maintaining systemic hypertension. Many studies have demonstrated that performing renal denervation (RD) using both catheter-based and Focused Ultrasound have achieved significant decreases in blood pressure. Mixed clinical trial results and the lack of an acute metric that demonstrates successful RD highlight the need to find alternative technologies and monitoring techniques that can consistently result in RD.

This work evaluates using MRgFUS as a RD technique in a normotensive rat model. Real-time MR monitoring, acute invasive blood pressure probe measurements and histological results all demonstrate the potential of using MRgFUS to control resistant hypertension.

**Methods**

In this pilot study, both male and female (n = 6 treated/5 sham) normotensive Sprague-Dawley rats underwent bilateral RD with MRgFUS. All procedures were performed in a 3 T MRI scanner (Siemens 3 T Trio) using a small animal MRgFUS system (f = 3 MHz, Image Guided Therapy). Sonications were applied along the length of both renal arteries (8 points/animal, 2 W, 20s) and monitored in real-time with 3D MR thermometry. Pre- and post-RD procedure T1 maps were obtained evaluating the entire insonified area. In 5 animals (36 total sonications), mean arterial pressure (MAP) was monitored continuously using an invasive fiberoptic blood pressure probe (SA instruments) inserted in the tail artery. One month post-RD procedure, animals were euthanized and kidney medulla norepinephrine concentration, a proven, robust metric of successful denervation, was obtained using an ELISA (Rocky Mountain Diagnostics). Histological analysis was performed on both renal arteries and surrounding tissues and kidney function, as measured by blood urea nitrogen and urinary albumin secretion, was evaluated.

**Results**

**Fig. 75 (abstract A74).**
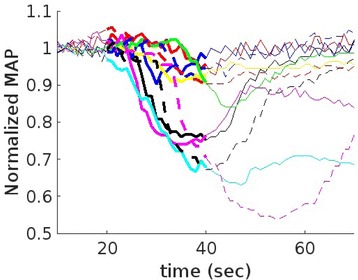
Normalized mean arterial pressure response for 11 sonications measured in 5 animals. Thick lines indicate the 20-second sonication time

**Fig. 76 (abstract A74).**
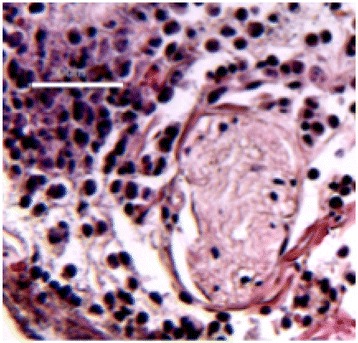
H&E stain of ablated renal nerve region with inflammatory cells, pyknotic nuclei and degraded nerve fiber indicated. Scale bar = 50 μm

**Fig. 77 (abstract A74).**
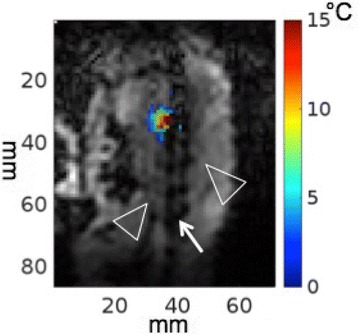
Coronal MR thermometry image showing the temperature rise measured in the back muscles (hollow arrows) during a single sonication. The white arrow indicates the spine

Acute blood pressure response: A transient decrease in MAP (>5 %) was observed during 11 of the 36 monitored sonications (~30 %). The normalized MAP measurements obtained during these 11 sonications are seen in Fig. 75. In all animals the effect was dependent on sonication location. In addition, the change in MAP occurred several seconds after the sonication start time indicating it was an accumulated effect. Treatment efficacy: Kidney medulla norepinephrine concentration was reduced by 36 % (p = 0.05) one-month post ablation when compared to the sham animals, indicating RD was successfully performed. Efficacy was further confirmed by the presence of inflammatory cells, pyknotic nuclei as well as degraded nerve architecture in the histological results (Fig. 76). Treatment safety: Comparison of the treated and sham RD animal’s blood urea nitrogen and urinary albumin/creatinine ratio resulted in no significant changes indicating kidney function was not affected by MRgFUS renal denervation. In addition, mean T1 values in both kidneys did not significantly change during the MRgFUS RD procedure. The peak mean temperature rise measured by real-time MR thermometry in the back muscle (Fig. 77) located in the near field of all animals ranged from 15.5 to 25.0 °C, confirming energy delivery and targeting accuracy.

**Conclusions**

Controlling resistant hypertension with renal denervation using Focused Ultrasound has been demonstrated in this and other studies to be a safe and potentially efficacious procedure. This work indicates that an acute, systemic response that is a function of sonication position can be detected during MRgFUS ablation of the renal sympathetic nerves. Future work will further evaluate this response in a hypertensive animal model and assess the effect of ultrasound parameters.

## A75 Trans esophageal HIFU for cardiac ablation: first experiment in non-human primate

### Paul Greillier^1^, Bénédicte Ankou^2^, Francis Bessière^2^, Ali Zorgani^1^, Mathieu Pioche^2^, Wojciech Kwiecinski^3^, Julie Magat^4^, Sandrine Melot-Dusseau^5^, Romain Lacoste^5^, Bruno Quesson^4^, Mathieu Pernot^3^, Stefan Catheline^1^, Philippe Chevalier^2^, Cyril Lafon^6^

#### ^1^INSERM / Université de Lyon, Lyon, France; ^2^Hospices Civils de Lyon, Lyon, France; ^3^Institut Langevin Ondes et Images, ESPCI ParisTech, CNRS UMR 7587, INSERM U979, Paris, France; ^4^IHU Liryc, Electrophysiology and Heart Modeling Institute, Pessac-Bordeaux, France; ^5^Station de Primatologie-UPS846-CNRS, Rousset sur Arc, France; ^6^University of Virginia, Charlottesville, Virginia, United States and INSERM Unit 1032, Lyon, France

**Objectives**

Atrial fibrillation and ventricular tachycardia are currently treated with catheter ablation using radiofrequency or cryoenergy. These endocardiac approaches are invasive and not fully satisfactory as the treatments are often incomplete and can be associated with serious side effects. The esophagus offers an excellent acoustic window to the heart and transesophageal HIFU were proposed as an alternative strategy. The present work describes the first attempt to perform thermal ablation with trans esophageal HIFU in the heart of a non-human primate.

**Methods**

For that purpose, an endoscope integrating a 5 MHz 64-element commercial transesophageal echocardiography (TEE) probe and a HIFU transducer was built. Anatomical configuration and numerical simulations (Constanciel 2013) allowed setting the features of the HIFU transducer: 8 elements truncated at 14 mm, 3 MHz operating frequency and 40 mm focal length. The focus could be steered electronically over a 15 to 55 mm range from the transducer. Circulation of water at 5 °C inside the endoscope ensured cooling of the front face of the HIFU transducer and acoustic coupling through the inflation of a latex balloon. The probe was tested *in vivo* in a 30 kg-baboon after preliminary TEE and CT-scanner demonstrated the acoustic access from the esophagus to the heart in this animal model. HIFU were delivered to the interatrial septum, the inferior wall of the left atrium and the upper posterior wall of the left ventricle (LV). A multi channel amplifier allowed delivering a focal intensity (Ispta) of 3000 W/cm2 applied 4 times for 16 s at each selected locations. B-mode, shear-wave (SWE) and passive elastography were performed before and after HIFU with an ultrafast scanner. MR imaging (T1 mapping and contrast) was performed one-day post HIFU. Animal experimentation approved under agreement 04938.04.

**Results**

The endoscope was successfully inserted in the esophagus of the baboon. The inflation of the latex balloon allowed acoustic transmission from the esophagus to the heart and visualizing satisfactorily the cardiac structures by TEE. HIFU could be delivered at the locations identified on the CT scans even though the heart was slightly shifted in the chest due to different positions of the animal during CT and HIFU procedures. The ECG was not impacted by the treatments. No change in echogenicity of treated zones could be evidenced after HIFU. SWE showed a stiffening of the LV myocardial wall after ablation (two-fold increase of the myocardial stiffness). Increase of stiffness could be observed similarly in the beating heart on passive elastogram. MRI of the atrial wall for detecting thermal ablation could not be achieved. A hypo signal zone could be evidenced on T1 maps in the lateral wall of the LV where one treatment was performed. Contrast MRI did not show local fibrosis in the heart. Endoscopic examination did not reveal esophageal damage. The baboon recovered properly from the protocol and no significant side effect could be evidenced for one month after treatment.

**Conclusions**

This experiment was a first attempt to perform thermal ablation in the heart with a trans esophageal probe *in vivo* in a non-human primate. While the procedure was safe and lesions seemed to be induced in the heart, developments will be necessary in order to 1- Deliver more power locally by HIFU, to compensate for motion and heat sink by blood circulation and to generate more pronounced lesions, 2- Improve the performance of monitoring techniques for the atrium (sensitivity and resolution) and for the ventricle (difficult to generate shearwave farther than 50 mm from the esophagus).

## A76 Ex vivo and in vivo non-invasive ultrasound-based cardiac pacing

### Fabrice Marquet, Pierre Bour, Fanny Vaillant, Sana Amraoui, Rémi Dubois, Philippe Ritter, Michel Haïssaguerre, Mélèze Hocini, Olivier Bernus, Bruno Quesson

#### IHU Liryc, Electrophysiology and Heart Modeling Institute, Pessac-Bordeaux, France

**Objectives**

Currently, no non-invasive cardiac pacing device acceptable for prolonged use in conscious patients exists. The main approach is invasive, employing intravascular catheters, which has associated risks. Focused ultrasound can be used to perform remote pacing using reversibility of electromechanical coupling of cardiomyocytes. This technique might be useful in the short term in the clinical settings in various conditions: temporary pacing for bradycardia or any clinical condition with risks of asystole; terminating or examining the inducibility of tachyarrhythmia; screening and optimization of cardiac resynchronization therapy. Here we described an extracorporeal cardiac stimulation device and study its efficiency and safety.

**Methods**

*Ex vivo* acoustic stimulation threshold was determined performing 756 sonications in 10 pig beating hearts. *In vivo* non-invasive stimulation was performed using 314 sonications in 4 anesthetized pigs. The animals were injected with ultrasound contrast agents. Experiments were performed using a Focused Ultrasound device (256 elements, 13/13 cm aperture/focal, operating at 1 MHz) under MR-guidance. At the end of each *in vivo* experiment, a navigated delayed inversion-recovery 3D Flash sequence was performed. Masson’s staining was performed to assess acute damages screening from acoustic stimulation of both *ex vivo* and *in vivo* experiments.

**Results**

**Fig. 78 (abstract A76).**
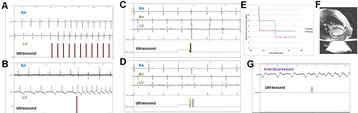
See text for description

Using HIFU it was possible to perform ventricular continuous pacing (Fig. 78a) or to induce ventricular tachycardia (Fig. 78b). Consecutive stimulations of different heart chambers with a single ultrasonic probe was shown, allowing to modify the resulting atrio-ventricular delay (Fig. 78c-d). The results of the 756 stimulation sites performed in the right atrium, and the left and right ventricles in 10 *ex vivo* beating hearts from pigs were processed to determine stimulation threshold for pulse duration ranging from 30 μs to 10 ms. Two different pressure thresholds were highlighted: one around 4 MPa peak negative for HIFU pulse durations above 1 ms and one around 6 MPa peak negative for HIFU pulses ranging from 50 μs to 1 ms (Fig. 78e). The same setup was used *in vivo* in 4 pigs to show clinical potential (Fig. 78f). Electrophysiological changes were also confirmed by arterial pressure modifications (Fig. 78g). The maximal peak negative pressure was estimated to be around 2 MPa at focus during *in vivo* experiments, due to the limited acoustic window. At this pressure level, stimulation of the LV was observed but with an insufficient success rate. Using ultrasound contrast agents, consistent cardiac stimulation was achievable for up to 1 hour sessions in 4 different animals. No damage was observed in the 4 animals.

**Conclusions**

To the best of our knowledge, this study is the first *ex vivo* and *in vivo* proof of feasibility of controlled non-invasive ultrasound-based cardiac stimulation in large animals. Preliminary safety results showed that this novel technology offers good prospects for clinical developments.

## A77 Restoring perfusion after critical hindlimb ischemia using pulsed Focused Ultrasound and mesenchymal stem cell in aged mouse

### Pamela Tebebi^1^, Scott Burks^2^, Saejeong Kim^2^, Blerta Milo^2^, Joseph Frank^2^

#### ^1^National Institutes of Health, Bethesda, Maryland, USA; ^2^National Institutes of Health Clinical Center, Bethesda, Maryland, USA

**Objectives**

Critical limb ischemia (CLI) is associated with a 5 year mortality rate in excess of 70 % with limited effective therapies. The goal of this study was to determine if pulsed Focused Ultrasound (pFUS) would enhance homing of mesenchymal stromal cells (MSC) to CLI in an aged mouse model and reestablish perfusion compared to pFUS or MSC alone.

**Methods**

CLI model was created by cauterizing the external iliac artery (EIA) on C3H mice (age = 10-12 months). Laser Doppler perfusion imaging (LDPI) was perform of lower extremities to confirm surgery and subsequently performed for weekly for 7 weeks post surgery. At 14 days post surgery, mice were divided into 4 groups: saline (n = 8), pFUS (n = 8), MSC (n = 8), and MSC + pFUS (n = 17). Mice received either 3 consecutive days of saline, pFUS , MSC, or MSC + pFUS starting on day 14 post surgery. 106 human MSC were administered IV. CLI mice (n = 3/group) were euthanized after one dose of either MSC + pFUS or MSC alone to determine if there was increased cell homing to ischemic muscle. pFUS exposures was performed with VIFU 2000 with a 1 MHz transducer; at 4 MPa; pulse repetition frequency, 1 Hz; DC 5 %; 100 pulses per point. Histology for vascular cell density from ischemic limb was performed at 7 weeks post EAI. Fluorescent microscopy was performed for human vascular endothelia growth factor (VEGF) and interleukin (IL) 10 one day after treatment with MSC alone or MSC + pFUS to determine if pFUS improved the potency in CLI.

**Results**

**Fig. 79 (abstract A77).**
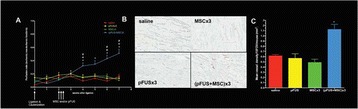
Contains the results from the LDPI studies at 7 weeks post surgical ligation of the external iliac artery demonstrating clear differenes in perfusion in the lower extremities for the saline control, pFUS and MSC alone groups versus the (pFUS + MSC)x3 group. Figure 79a is a graph of the ratio of measured perfusion in the ischemic limb/contralateral limb. The only group that showed an increase in perfusion starting 2 weeks after intervention with pFUS with our without MSC or MSC alone is the cohort of mice that received both treatments. The pFUS + MSCx3 group of mice had approximately 60 % recovery of perfusion to the hind limb compared to the other cohorts of animals. Figure 79b contains representative sections from the hamstring muscle stained for CD31+ endothelial cells (brown cells) clearly showing an increase in vascular density in the (pFUS + MSC)x3 group of mice compared to the other groups (Fig. 79c)

LDPI demonstrated significant (p < 0.01) differences between (MSC+ pFUS) *versus* saline, pFUS, and MSC groups when treatment was delayed 2 weeks after CLI (Fig. 79). Perfusion significantly increased with the MSC + pFUS treatment out to 7 weeks compared to other cohorts. Histological examination of muscle revealed significant increase (p < 0.05) in CD31+ cells and vascular density treated with MSC + pFUS compared to other groups (Fig. 79b,c). Mice were euthanized on day 15-post surgery and human cells were counted in CLI muscle to determine if MSC + pFUS would enhance homing of infused cells in CLI model. Following IV MSC alone, MSC detected in the ischemic limb 101.0 ± 67.4 compared to mice that received pFUS + MSC, MSC in the ischemic limb was 306 ± 175 (p < 0.01 for MSC alone vs. pFUS + MSC). We also observed ~4-6 fold increases (p < 0.05) in human VEGF and IL10 expression in MSC + pFUS compared MSC alone groups.

**Conclusions**

This study demonstrates that pFUS enhanced homing of IV MSC to targeted muscle resulting in reperfusion and neovascularization in CLI model compared to MSC alone. pFUS preconditioning effects in the ischemic muscle stimulated local molecular changes in the tissue microenvironment that when combined with MSC infusion increased stem cell numbers and potency by producing increased amount anti-inflammatory faction that lead to increased perfusion compared to MSC alone. The ability of pFUS to modulate the molecular microenvironment in chronic diseased tissue opens the possibilities for enhancing cellular therapies in regenerative medicine and potentially improves clinical outcomes in patients suffering from CLI.

## A78 A novel ultrasound guided therapeutic system for hypertension treatment

### Michael Gertner, Jimin Zhang

#### Kona Medical, Inc., Bellevue, Washington, USA

**Objectives**

Hypertension affects 1.2 billion people worldwide. A novel method to address this need is ablation of the renal (kidney) sympathetic nerves which run along the renal arteries (“RDN”). In contrast to catheter-based RDN, which relies on intravascular delivery energy through the wall of the artery to ablate renal nerves, the Kona Medical Surround Sound™ system delivers non-invasive Focused Ultrasound energy from outside the body.

**Methods**

Surround Sound® System is a fully-integrated, self-contained system which can be used in virtually any exam room in a hospital, office or clinic, and does not require a catheterization lab. The system administers focused therapeutic ultrasound to the renal nerve complex using a custom therapeutic transducer array with a unique shape consisting of 205 individually phased elements that can provide more than 800 W peak acoustic power. The clinical dose includes 14 lesions delivered over a 2.8 minute period. A separate ultrasound imaging probe is used to identify the renal artery position and an optical tracking system transforms the target location position via the imaging array to a motor that steers the therapy array to the selected target. A fully automated image tracking algorithm corrects for target motion while interleaved imaging and therapy pulses provide monitoring during the entire treatment. Beamforming and target motion tracking utilize fast, GPU computation to incorporate robotic, closed-loop motion control for 3D energy delivery, focusing, and positioning in real-time.

**Results**

69 patients with uncontrolled hypertension were treated in the first 3 clinical trials (WAVE I, II and III) using the Kona Surround Sound™ system. 64 out of 69 subjects have completed at least one year follow up with an average systolic and diastolic blood pressure reduction of 23.8 mmHg/10.3 mmHg at one year. No devices related unanticipated serious adverse events occurred. This data suggests that externally delivered ultrasound appears to be safe with an encouraging efficacy signal. A blinded, randomized, sham-controlled clinical trial (Wave IV; 132 patients) is currently enrolling at leading health care institutions in the United Kingdom, Czech Republic, Germany, New Zealand, and Poland. 81 eligible study participants have been treated to date.

**Conclusions**

The Surround Sound system, which is developed to provide a one-time, non-invasive procedure to treat hypertension, has the potential to greatly reduce cost, lower risk, and improve access to millions of hypertension patients worldwide who are not adequately controlled by drug therapy. The initial clinical data have shown that ablation of renal nerves using Surround Sound system can result in profound and lasting reduction in blood pressure. The technology platform in which the system targets specific tissue while automatically tracking movement and continuously enabling therapy delivery through a custom shaped high power therapeutic phased array, could be easily adapted and used in other clinical applications.

## A79 Ultrasound ablation enhances nanoparticle accumulation and survival in mammary carcinoma models

### Andrew Wong, Brett Fite, Yu Liu, Azadeh Kheirolomoom, Jai Seo, Katherine Watson, Lisa Mahakian, Sarah Tam, Hua Zhang, Josquin Foiret, Alexander Borowsky, Katherine Ferrara

#### University of California Davis, Davis, California, USA

**Objectives**

Magnetic Resonance-guided Focused Ultrasound (MRgFUS) ablation is a noninvasive method for treating solid tumors. However, ablation of the entirety of the tumor may not be possible due to constraints imposed by surrounding tissue, resulting in a small rim of viable tumor following thermal therapy. Therefore, augmentation of MRgFUS with chemotherapeutics such as liposomal doxorubicin may be necessary. These formulations reduce systemic toxicity by encapsulating non-bioactive copper-doxorubicin crystals which dissociate in the low pH of the tumor microenvironment. Our ultimate goal is to use Focused Ultrasound (US) within curative protocols in the mouse and in human medicine.

**Methods**

All studies were approved by the UC Davis Institutional Animal Care and Use committee. Tumors were generated in FVB/n mice expressing an activated form of ErbB2/neu, a system modeling human HER2 amplified breast cancer. These tumors were then transplanted into the mammary fat pads of wild type FVB/n mice. The results were validated in another mammary carcinoma model (4 T1 in BALB/c mice).

US ablation was accomplished under Magnetic Resonance (MR) guidance via either a 20 s insonation at a single point or a 60 s insonation in a circular pattern (16 element annular array with a 3 MHz central frequency, 3.1 MPa peak negative pressure (PNP)). Both protocols induced temperatures >65 °C and CEM43 > 5000. Hyperthermia (41 °C) was induced using a 192-element transducer with 128 therapeutic elements operated with a center frequency of 1.5 MHz and 1.1 MPa PNP, under US guidance and a needle thermocouple for temperature monitoring.

Temperature-sensitive and long-circulating doxorubicin liposomal formulations (Dox-TSL and Dox-LCL, respectively) were evaluated in combination with each ablation protocol. Accumulation of 64Cu-labelled liposomes was assessed with positron emission tomography (PET) and autoradiography following each of the ablation protocols.

**Results**

**Fig. 80 (abstract A79).**
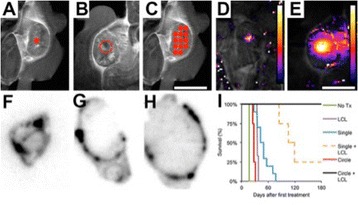
T1w MR localize ultrasound for (**a**) point, (**b**) circle, (**c**) grid ablation. MR thermometry following (**d**) 20 s point, (**e**) 60 s circle ablation. Autoradiography 48 hours after (**f**) point, (**g**) circle, (**h**) grid ablation. (**i**) Survival plot for all animal groups

Ablation enhanced accumulation of 64Cu-LCL in the remaining rim of viable tumor (Fig. 80), with a 5-fold increase in nanoparticle and 50-fold increase in local drug concentration. With the combination of a 5 mm circular ablation region and Dox-LCL, tumors were eliminated in all mice and recurrence was not observed within 180 days. Integrating this sphere of ablation with Dox-TSL achieved a durable response with 4 treatments in 75 % of mice treated. Alternatively, preapplication of hyperthermia to enhance release of Dox-TSL prior to the application of the spherical region of ablation produced durable response in 100 % of mice within 4 treatments.

**Conclusions**

In conclusion, initial results for the combination of hyperthermia, ablation and liposomal doxorubicin in a fully immunocompetent aggressive syngeneic model demonstrated multiple opportunities to achieve a durable response in local disease.

## A80 Targeted drug delivery with cyclodextrin-based nanocarriers and Focused Ultrasound triggering

### Doudou Xu^1^, Andreas Melzer^2^

#### ^1^Chongqing Changlin Science & Technology Ltd., Chongqing, China; ^2^University of Dundee, Dundee, UK

**Objectives**

The Nanoporation project set out to explore specific solutions to overcome the current challenges of targeted drug delivery (TDD) to tumours using Magnetic Resonance Imaging guided Focused Ultrasound (MRgFUS) to cavitate microbubbles (MBs) for increasing cell permeability and to open ‘drug nano-capsules’ to release proven active anticancer drugs directly to the tumour site with reduction of systemic drug dosage needed for the desired therapeutic effect. The work reported here aimed to develop novel nano-carriers for existing anticancer drugs, by establishment of human cancer cell models to evaluate the carriers’ encapsulation efficiency *in vitro* and *in vivo*, by using animal models and a clinical MRgFUS system to investigate the carrier-drug vehicles’ *in vivo* distribution and localised drug release / cellular drug uptake.

**Methods**

**Fig. 81 (abstract A80).**
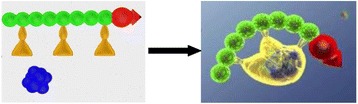
γ-CD based drug delivery vehicle

**Fig. 82 (abstract A80).**
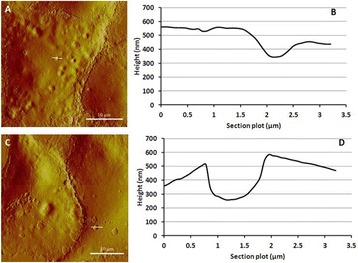
AFM cell surface morphology of KB cells after sonication with and without MBs

**Fig. 83 (abstract A80).**
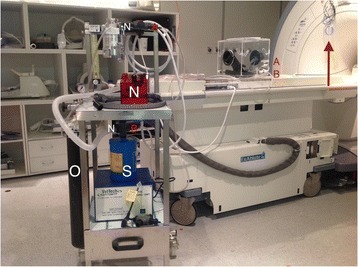
MRgFUS compatible small animal house for ex vivo and in vivo study

**Fig. 84 (abstract A80).**
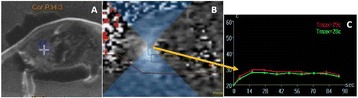
MRgFUS ex vivo study

A novel γ-cyclodextrin (γ-CD) based carrier for encapsulation of doxorubicin (DOX) was synthesised and fully characterized (Figs. 81 and 82). The encapsulation efficiency was assessed under various temperatures and pH levels by both chemical analysis and *in vitro* human cancer cell modeling with KB and HCT116 cells. A computer-controlled high-throughput *in vitro* FUS device facilitating exposure to High Intensity FUS fields in a standard 96-well plate was designed and applied. It allows maximum flexibility in choosing experimental parameters in combination with carrier-DOX inclusion. SonoVue® MBs was used to investigate TDD in cell monolayers. An extensive *in vitro* study had been carried out to investigate both mechanical and thermal effects of ultrasound. An unique MRgFUS system compatible anmial house were designed and built to allow *ex vivo* and *in vivo* experiments by using small rodents (Fig. 83). *Ex vivo* and *in vivo* trials were carried out with a clinically approved ExAblate MRgFUS system (InSightec, Israel) to establish a safe and efficient clinical TDD protocol on small rodents (Fig. 84).

**Results**

The desired γ-CD based carrier greatly reduced DOX’s toxicity and the carrier-DOX inclusion was highly stable under physiological temperature conditions as well as under a wide range of acidic conditions (pH 1.0 ~ 7.0); the encapsulated DOX is slowly released under hyperthermic conditions (up to 50 °C). In the presence of MBs, application of FUS with low mechanical indexes, under which no thermal effect was observed, enhanced the drug uptake into tumour cells for both encapsulated and free DOX. Optimal setups of MR parameters and FUS parameters were identified *ex vivo* and *in vivo*, allowing application of MRgFUS treatments to 4 live mice bearing tumours (human colorectal carcinoma, up to 1059.71 mm3) under anaesthesia with full recovery.

**Conclusions**

The study demonstrated the possibility of translation of the constructed γ-CD derivative to potential clinical use as a delivery vehicle for DOX using combined thermal and mechanical release mechanisms by clinically applicable MRgFUS– triggered TDD with the potential for cancer therapy. it provides better understanding of the mechanism of and potential for this novel delivery approach: MRI-guided, ultrasound-mediated, site-specific drug delivery assisted by MB contrast agents. The chemical modifications and *in vitro*, *ex vivo* and *in vivo* preclinical studies discussed here represent only a glimpse into the future of clinical application. It is likely that this novel technology will enter the clinical arena in the near future, based on the ever-increasing scientific contributions from researchers.

## A81 Image guided liposomes for Focused Ultrasound assisted anticancer drug delivery

### Maya Thanou^1^, Miguell Centelles^1^, Mike Wright^1^, Maral Amrahli^1^, Po-Wah So^1^, Wladyslaw Gedroyc^2^

#### ^1^King’s College London, London, UK; ^2^Imperial College Healthcare London, London, UK

**Objectives**

Thermosensitive liposomes (TSLs) have been reported to accumulate and deliver an effective therapeutic to tumours with the application of hyperthermia. However there is limited understanding of the effect of pharmacokinetics, the time frames and the duration of hyperthermia applications. For instance, the timing between drug injection and application of focused ultrasound (FUS) needs to be optimised considering nanoparticle biodistribution in the tumour and drug release. Near-Infrared Fluorescence (NIRF) imaging offers an easy and sensitive method to follow the biodistribution of dye-labelled theranostic nanoparticles and drug release in real time and could act as a useful preclinical surrogate for other forms of imaging to help understand the mechanism of drug kinetics in the tumours. In the present study we examined the biodistribution of novel dual MRI and NIRF-labelled thermosensitive liposomes (image guided; iTSLs), and we modulate their distribution with hyperthermia induced by a high intensity focused ultrasound transducer (FUS).

**Methods**

We have synthesized lipids that can be used for imaging in mice by MRI and NIRF. We have prepared iTSLs with the optimum concentration of imaging lipids for thermally triggered release and we developed them in combination with a FUS treatments regimen (thermal dosing). Labeling of liposomes for imaging can provide substantial information of the mechanism of tumor uptake (post injection) and provide insight on the reasons why this uptake is more enhanced in FUS treated tumors. We prepared iTSLs to encapsulate the anticancer drug topotecan (Hycamtin®), a chemotherapeutic agent which when released in vivo can be monitored by its intrinsic drug fluorescence. We have optimized drug encapsulation for maximum fluorescence signal difference (before /after release) for both in vitro and in vivo. FUS (TIPS Phillips) was applied using temperature feedback via subcutaneously placed fine-wire thermocouples to maintain hyperthermic temperatures. NIRF imaging was performed using multispectral analysis bioimaging (Maestro EX) following the emission of the NIRF lipid and topotecan. FUS was applied using imaging as guidance. FUS was applied 30 min post injection and the tumors were monitored. In a separate group of animals FUS was applied at 30 and 90 min post injection. MRI imaging confirmed observations obtained with NIRF imaging.

**Results**

**Fig. 85 (abstract A81).**
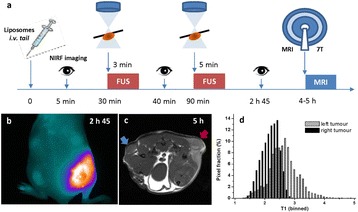
NIRF and MRI imaging after dual FUS application

iTSL tumour accumulation was detected using NIRF imaging immediately after liposome administration, this is due to the enhanced permeability and retention effect. Mice were bearing tumours at both flanks allowing one tumour to act for control (no FUS treatment). FUS-induced hyperthermia (3 min at 42 oC, 30 min post i.v.) greatly enhanced liposomal uptake as seen by imaging. A co-localised, enhancement of topotecan fluorescence emission was also observed immediately after application of hyperthermia indicating rapid thermally triggered drug release within the area of the tumour. Topotecan is used as an anticancer drug model and its intrinsic fluorescent properties allow to easily follow thermal drug release. The phenomena of increased iTSL accumulation and concomitant topotecan release appeared to be amplified by a second mild hyperthermia treatment applied 1 hour after the first. NIRF imaging detected the signal coming from the liposomes (NIRF incorporated lipid) indicating that liposomes accumulated in tumours post injection. This accumulation was substantially enhanced after each application of FUS. Topotecan fluorescent appeared transient in the tumour indicating phenomena of cell uptake and DNA binding. MR imaging also confirmed enhanced iTSLs uptake due to the FUS treatments in the same animals that were imaged using NIRF imaging (Fig. 85).

**Conclusions**

In this study we have formulated a theranostic novel dual MRI/NIRF labeled thermosensitive liposome (iTSL) that encapsulates the anti-cancer agent topotecan and enables real time/diagnostic imaging, making use of pre-clinically and clinically relevant imaging modalities. Image-guidance in turn enables the application of brief, moderate intensity FUS treatments that greatly increase iTSL tumor uptake and set up the possibility for substantial FUS triggered topotecan release within the tumor volume. FUS hyperthermia applied during the half-life of the iTSL in plasma promotes iTSL accumulation in the tumors and takes advantage of the high concentration of the liposomal drug in plasma seen immediately after injection. Should these effects be translated to the clinic, this suggests substantial benefits to cancer patients and improvements of the standard of the chemotherapy treatments for both primary and metastatic tumors.

## A82 Near infrared fluorescence imaging for Focused-Ultrasound mediated local drug delivery of doxorubicin

### Miguell Centelles^1^, Mike Wright^1^, Wladyslaw Gedroyc^2^, Maya Thanou^1^

#### ^1^King’s College London, London, UK; ^2^Imperial College Healthcare London, London, UK

**Objectives**

High-intensity Focused Ultrasound (FUS) is valuable clinical technique for non-invasive thermal tissue ablation. Its capability for localised drug delivery has also been investigated recently, with application to cancer therapies. The ability of thermosensitive liposomes (TSLs) to accumulate and deliver a therapeutic drug to tumours under application of hyperthermia has been well reported but there is limited understanding of the time frames and kinetics involved. For instance, the optimal delay (if any) between injection of the therapeutic and application of heat. Near-Infrared Fluorescence (NIRF) imaging offers a sensitive method to follow the biodistribution of dye-labelled theranostic nanoparticles in near real time and acts as a useful preclinical surrogate for other forms of imaging (such as PET or MRI).

**Methods**

**Fig. 86 (abstract A82).**
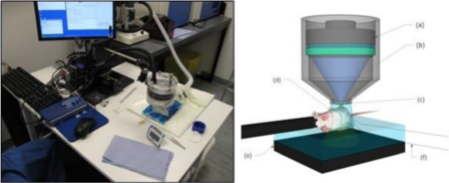
TIPS focused ultrasound (FUS); (left) overview of the equipment showing the water-filled transducer chamber, the thermocouple interface, and the control PC; (right) schematic of the in vivo configuration with the transducer (**a**) raised such that the ultrasound biconic (**b**) focuses just above the skin surface over the tumour (**c**). The mouse is surrounded with warmed, degassed ultrasound gel (**d**) and placed on an ultrasound absorbing mat (**e**) to prevent reflections off the table. Temperature monitoring is via two or three fine-wire thermocouples (**f**) implanted around the tumour

Our iTSLs (imageable TSL) containing doxorubicin (0.8 mg/mL) were formulated with different molar ratios of key lipids, and a dye-labelled lipid - XL750-DSA. In vitro studies were performed to characterise the temperature-triggered doxorubixin release by measuring changes in its intrinsic fluorescence. In vivo experiments were performed on SHO mice bearing subcutaneous MDA-MB-231 tumours on both flanks. For bioimaging experiments, iTSLs were injected i.v. with and without any hyperthermia induced by FUS (TIPS, Philips) and controlled by three thermocouples placed around the tumour (Fig. 86). TSLs and doxorubicin were respectively monitored by recording the emitted fluorescence in the NIR and the blue regions of the spectrum. Finally, tumour sizes were measured by calliper for 3-4 weeks following treatment.

**Results**

**Fig. 87 (abstract A82).**
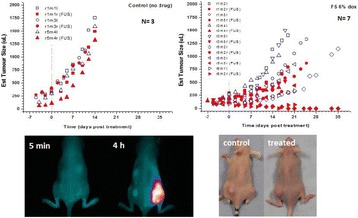
Treatment efficacy: Mice were injected i.v. with F5-XL750 (6 mg/kg) and were treated twice by FUS hyperthermia applied to the right side

**Fig. 88 (abstract A82).**
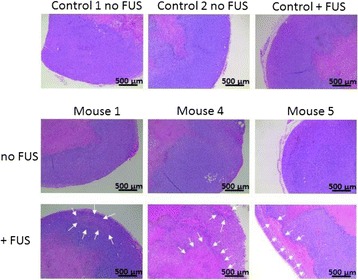
Histology (H&E). The tumours were excised 48 hours after the treatment. Four micrometer H&E slices of MDA-MB-231 tumors untreated and treated. The arrows indicate the large necrotic areas only found when the combination of drug and hyperthermia was applied

In vitro release studies showed minimal leakage of the drug at 37oC over a 10 min period, while the release was almost immediate when the temperature was raised to 41oC. In vivo, a first hyperthermia event (3 min at 43oC) was applied on one of the two tumours before iTSLs administration. After 30 min, the iTSLs were injected i.v. (tail vein) and their tumour accumulation was monitored by tracking the NIRF signal. A second hyperthermia treatment (3 min at 42 oC) was then applied 45 min later in order to release the doxorubicin. As a consequence of this regimen, the mice showed a very significant NIRF intensity increase in the treated area. At the same time, doxorubicin release was observed but only for a short period of time. The combination of this protocol & doxorubicin-iTSLs showed a significant impact on tumour growth, including tumour eradication in some cases (Figs. 87 and 88).

**Conclusions**

NIR fluorophores carried by the iTSLs allowed the monitoring of the liposomes’ biodistribution over a period of several weeks post-injection in our mouse model. Mild hyperthermia applications on the tumour greatly favoured nanoparticle uptake. The hyperthermia also had the effect of releasing encapsulated doxorubicin, therefore improving the drug delivery to the tumour. Finally, a strong correlation between thermal dosing and tumour targeting was observed with an increase of the NIRF signal in the heated area with a subsequent enhanced therapeutic effect.

## A83 Interleaved mapping of temperature and longitudinal relaxation rate monitor drug delivery from temperature sensitive liposomes during MR-HIFU induced hyperthermia

### Esther Kneepkens^1^, Edwin Heijman^2^, Jochen Keupp^3^, Steffen Weiss^3^, Klaas Nicolay^1^, Holger Grüll^2^

#### ^1^Eindhoven University of Technology, Eindhoven, Netherlands; ^2^Philips Research, Eindhoven, Netherlands; ^3^Philips Research, Hamburg, Germany

**Objectives**

Not released for publication

**Methods**

Not released for publication

**Results**

Not released for publication

**Conclusions**

Not released for publication

## A84 Ultrasound ablation transiently increases accumulation of small molecule gadoteridol within the ablated volume

### Brett Fite, Andrew Wong, Yu Liu, Azadeh Kheirolomoom, Lisa Mahakian, Sarah Tam, Josquin Foiret, Katherine Ferrara

#### University of California Davis, Davis, California, USA

**Objectives**

Magnetic Resonance guided Focused Ultrasound (MRgFUS) ablation is a nonvasive method to deliver a precise, and lethal, thermal dose to solid tumors. During thermal ablation, tissue temperature typically exceeds 50-60 °C and results in rapid coagulative necrosis. Such ablation often results in “heat fixing” of the tissue which is thought to restrict the diffusion of molecules into and out of the ablated region. In this study we used gadoteridol (MW 558D) to evaluate the extent to which small molecules could be delivered to tissue following thermal ablation.

**Methods**

All studies were approved by the UC Davis Institutional Animal Care and Use committee. MRgFUS ablation of syngeneic murine mammary carcinoma (FVB/n mice expressing an activated form of ErbB2/neu) was accomplished via a 60 s insonation in a circular pattern (16 element annular array with a 3 MHz central frequency, 3.1 MPa PNP). This ablation protocol resulted in temperatures >65 °C and CEM43 > 5000.

Animals were injected with intraperitoneal gadoteridol either immediately before or after treatment (0.05 mmol/kg) or with intravenous gadoteridol before or after treatment. Mice were then imaged with a T1w scan (TE/TR = 12.5/750 ms, FOV = 3.2 cm × 3.2 cm, MTX = 256 × 256, ST/SI = 1/1 mm, and 9 slices) at 0.5, 1.5, 3, 6, 20, and 48 hours following ablation. The ablated region and quadriceps were then manually segmented and the tumor-to-muscle (T/M) ratio measured. In addition, two control groups of mice were also imaged with T1w MRI, a set treated with circle pattern MRgFUS and but no gadoteridol and a set not treated with ultrasound, but injected with intraperitoneal gadoteridol.

**Results**

**Fig. 89 (abstract A84).**
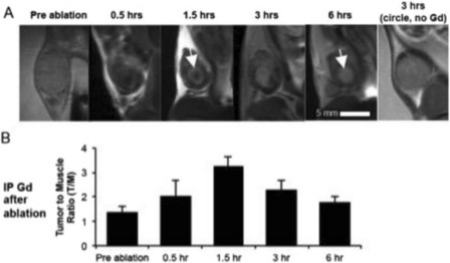
**a** T1w images of a tumor prior to, and 0.5 hours, 1.5 hours, 3 hours, and 6 hours following MRgFUS ablation. Control tumors that received ablation but no gadoteridol did not show regions of increased T1w signal. **b** Tumor to muscle ratio of T1w signal

Immediately following ablation, the T1w signal of the ablated volume increased, beginning with the rim. The T1w T/M signal ratio progressively increased until 1.5 hours post-ablation where it peaked (Fig. 89). The T/M ratio remained elevated compared to pre-ablation values for up to 6 hours post-ablation. Control animals treated with the same ablation protocol without gadoteridol injection did not exhibit an increase in T1w signal post-ablation. At 20 and 48 hours post-ablation, the T1w signal had decreased to pre-ablation levels and re-injection of gadoteridol at these time points had no effect on tumor T1w signal.

Thus, the small molecule gadoteridol accumulated within the tumor, specifically within the ablated volume, for several hours following ablation.

**Conclusions**

The results suggest that there is a window of time post-ablation when small molecule drugs can be successfully delivered to ablated tissue.

## A85 Pulsed Focused Ultrasound to kidneys induces Molecular changes that potentiate mesenchymal stem cells to further improve acute kidney injury

### Scott Burks, Matthew Nagle, Saejeong Kim, Blerta Milo, Joseph Frank

#### National Institutes of Health Clinical Center, Bethesda, Maryland, USA

**Objectives**

Pulsed Focused Ultrasound (pFUS) enhances homing of IV-infused mesenchymal stem cells (MSC) to kidneys during cisplatin (CIS)-induced acute kidney injury (AKI). pFUS serves as a neo-adjuvant to MSC therapy where sonicating kidneys with IV MSC leads to better AKI outcomes than MSC therapy alone. Nearly twice as many MSC home to pFUS-treated AKI kidneys, but >10 times as much interleukin (IL)-10 is produced by MSC that home to pFUS-treated kidneys. This result suggests that pFUS sonications modify the renal microenvironment to increase potency of MSC that home to pFUS-treated kidneys. Interferon-g (IFNg) increases MSC potency and is upregulated in kidneys by pFUS. This study investigates pFUS-induced IFNg expression in kidneys as an activator of MSCs that improves their therapeutic efficacy during AKI.

**Methods**

C3H or IFNg-ko mice received CIS (15 mg/kg ip), kidney pFUS (4 MPa; 5 % duty cycle) and/or MSC (106 human MSC). IV MSC injections were performed 3-4 hr post-pFUS. siRNA was used to knockdown IL-10 in some experiments. Experimental groups included AKI only, AKI + pFUS, AKI + MSC, AKI + pFUS + MSC, and healthy mice. Mice received CIS on Day (D)0 and pFUS/MSC on D1. Mice were euthanized on D4. One-way analysis of variance used Bonferroni post-hoc with p-values <0.05 considered significant.

**Results**

**Fig. 90 (abstract A85).**
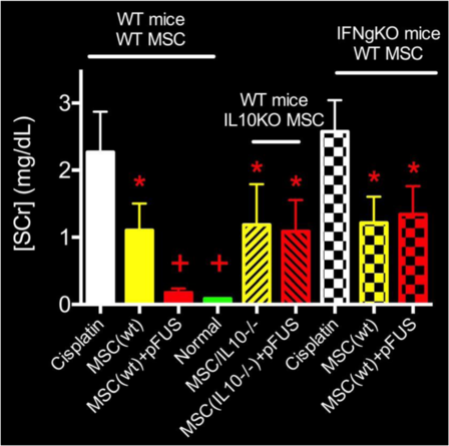
MSC better improve AKI with pFUS to increase renal IFNg and MSC IL10 expression. MSCs with reduced IL10 or IFNg-deficient mice to not see AKI benefit from pFUS + MSC

Following pFUS to the kidneys of IFNg ko mice, more MSC homed to sonicated kidneys (~2 fold) but did not lead to improved AKI [serum blood urea nitrogen (BUN) or serum Creatinine (SCr)] outcomes compared to mice that received MSC injections alone (Fig. 90). Levels of BUN and SCr, as well as expression of kidney injury molecule 1 (KIM1), were all significantly reduced by MSC treatment alone, but not further reduced by combination pFUS/MSC treatment like was previously observed in wild-type mice. Furthermore, the production of IL-10 by MSC seen in wild-type mice treated with pFUS + MSC was absent in IFNg-ko mice. Lastly, the improved AKI outcomes using pFUS + MSC in wild-type mice were erased when mice were given MSC that had been pretreated with siRNA to knockdown IL-10.

**Conclusions**

pFUS creates a molecular zip code in AKI kidneys that enhances MSC homing. While MSC infusions alone improve AKI in IFNg KO mice, pFUS + MSCs did not replicated the additional improvements in outcomes seen in wild-type mice. While pFUS-independent mechanisms of AKI repair by MSCs do not require renal IFNg, the pFUS-dependent mechanism that yields improved recovery does depend on preconditioning of the parenchyma. IFNg upregulated by pFUS mediates the neoadjuvant function of pFUS. Furthermore, the IFNg upregulated by pFUS serves to enhance MSC function by increasing MSC production of IL10, which has previously been shown to improve AKI. These data demonstrate that an IFNg/IL10 molecular axis is activated by pFUS and critical for improved AKI outcomes. Moreover, these results provide justification in incorporating pFUS as a treatment to improve MSC therapy during AKI, which often has limited clinical therapeutic options.

## A86 Use of focused beams of special structure for pushing and trapping kidney stones with acoustic radiation force

### Oleg Sapozhnikov^1^, Anastasia V. Nikolaeva^2^, Marina E. Terzi^2^, Sergey A. Tsysar^2^, Adam Maxwell^1^, Bryan Cunitz^1^, Michael Bailey^1^

#### ^1^University of Washington, Seattle, Washington, USA; ^2^Moscow State University, Moscow, Russian Federation

**Objectives**

Focused ultrasound not only causes lesions in biological tissue, but can effectively push the medium in the focal region. This effect, associated with the ability of ultrasound to remotely deliver and transform momentum to an absorbing or scattering target, is called radiation force. Until recently, therapeutic applications of this effect did not exist, although it has been employed in ultrasound imaging, e.g. shear wave elasticity imaging. Previously, our team developed a technology to reposition kidney stones with radiation force. In a clinical trial, the technology was used to transcutaneously facilitate passage of small stones and to relieve pain by dislodging larger obstructing stones. While successful, the trial revealed a need for optimization of the ultrasound beam structure, frequency, and intensity to make it more effective.

**Methods**

Two aspects of the kidney stone repositioning were studied. First, we investigated the optimal ultrasonic beam diameter vs. stone size. Numerical modeling was performed to calculate the force acting on an elastic sphere in liquid from a focused beam. Simulations were performed for a given acoustic power, changing the ratio of the beam width to the diameter of the stone. The second study investigated the feasibility of trapping and moving a stone in a direction transverse to the beam axis. Toward this goal, vortex beams were considered that produce a ring-like intensity distribution in the focal region with zero on-axis intensity. Theoretical modeling was performed using a previously developed algorithm. Acoustic trapping and manipulation of kidney stones in water was investigated using both a single-element transducer (combined with a sector-shaped phase plate) and sector arrays with operating frequencies 0.3–1.5 MHz. Human stones approximately 3–5 mm, as well as glass and aluminum beads, were displaced in a water bath.

**Results**

The numerical modeling indicated that pushing a kidney stone is strongest when the beam width is slightly wider than the stone diameter. This can be explained by more effective generation of shear waves inside the stone resulting from their effective coupling with the acoustic waves in liquid at the stone edges. During exposures with the vortex beams, stones could be drawn to the beam axis and then controllably translated along the surface in any direction transverse to the beam. The phase between sector elements could be used to control the vortex size and thus adjusted for trapping different-sized stones.

**Conclusions**

The intensity distribution in the focal region significantly influences the ability to manipulate kidney stones using radiation force. To achieve the strongest force with a predetermined acoustic power, it is necessary to select a mode in which the focal waist diameter slightly exceeds the stone diameter. For transverse confinement, it is convenient to use vortex beams radiated by sector arrays or single-element sources with an attached phase plate. In such an approach, the beam diameter can be controlled by choosing a proper phase distribution on the source surface.

## A87 Modulated Focused Ultrasound for treatment of de-myelinating axons in multiple sclerosis lesions — pilot animal studies

### Pierre Mourad

#### University of Washington, Seattle, Washington, USA

**Objectives**

Multiple sclerosis (MS) is a debilitating disease of the central nervous system whose symptoms arise from de-myelination of axons within brain and spinal cord tissue. De-myelination can lead to loss of central and peripheral function, including vision and muscular problems. There currently exists no cure for multiple sclerosis. In this exploratory study, we seek to induce re-myelination of axons de-myelinated by an MS model using pulsed Focused Ultrasound (pFU). There exists a rich history targeting the use of ultrasound to temporarily and non-destructively activate central neural circuits. Our own work has shown that pFU can induce detectable electroencephalography (EEG) signals in the brain. Recent work by Gibson et al. (2014) who showed increased myelin thickness of neurons that had been activated by laser light in an optogenetic mouse model. It seems plausible, therefore, that ultrasound induced neural activation could affect myelin growth and thickness.

**Methods**

We hypothesize that pFU activation of axons within MS lesions in a rodent model will decrease de-myelination and increase re-myelination. To test this, we selected the cuprizone model for MS, which causes de-myelination in the corpus callosum of the mouse brain and is reversible, allowing us to assess the effects of ultrasound during the phase of disease progression and during the phase of disease recovery. Rodents undergoing the MS model received five consecutive days of pFU therapy and underwent EEG monitoring. We applied pFU at two different time points, first during the demyelination process and second (in separate groups of animals) during recovery from demyelination. The pFU therapy protocols were inspired by the optogenetic stimulation protocols of Gibson et al (2014), and consisted of 30 seconds of pFU application followed by 90 seconds of rest, repeated 15 times. We tested three different pFU transducers with center frequencies of 0.65 MHz, 1.09 MHz, and 2.0 MHz, all with Ispta of 1.2 W/cm2*s. For each of those frequencies, we verified that each pFU protocol could activate brain. We collected Magnetic Resonance (MR) images at three time points and collected the brains for histological analysis at the end of the study.

**Results**

Initial results show that mice undergoing the MS model exhibit different shapes in their pFU-induced EEG signals than normal healthy mice. Further results of the therapeutic pFU treatment following analysis of MR and histological results will be presented.

**Conclusions**

If successful, this non-invasive therapy may lead to rapid advancements in the treatment of MS and other de-myelinating neurological disorders.

## A88 Noninvasive peripheral nerve stimulation via Focused Ultrasound in vivo

### Matthew Downs, Georgiana Yang, Qi Wang, Elisa Konofagou

#### Columbia University, New York, New York, USA

**Objectives**

The leading technique to treat peripheral neurological disorders is currently through implantation of electrodes along the peripheral nerve and stimulating the nerve with electrical current. Recently, Focused Ultrasound (FUS) has been shown to elicit modulation of both brain and peripheral neurons. While the effects of the FUS brain stimulation experiments have been shown *in vivo*, the majority of the peripheral nerve stimulation studies have been *in vitro*, or only investigating the effects of FUS stimulation on the nerve itself. Thus far, there have been no studies determining if non-invasive stimulation of peripheral nerves with FUS can elicit physiological effects *in vivo*.

**Methods**

Peripheral nerve stimulation was conducted on both hind limbs of 13 mice under general anesthesia (pentobarbital 65 mg/kg). Both the imaging and stimulation transducers were mounted interchangeably on a 3-DOF positioning system with 1-mm resolution. Targeting of the sciatic nerve was achieved using a 1282element imaging probe with a center frequency of 18.5 MHz. A single element transducer (3.57 MHz center frequency, 90 % duty cycle, 1 kHz PRF, 5-10 ms sonication duration, 3.65 x 0.45 mm focal area) was used for stimulation of the sciatic nerve. Evoked EMG activity in the Tibialis Anterior muscle was recorded via two stainless steel electrodes sampling at 2 kHz. Gross pathology was used to assess safety of the procedure. Behavioral testing occurred an hour before, and 24 hours after FUS stimulation. Electrophysiology experiments were used as positive controls with electrodes directly stimulating the sciatic nerve while recording EMG from the Tibialis Anterior muscle. Negative controls were conducted via administration of 5 mg/kg lidocaine to the stimulation region as well as clipping the nerves downstream from stimulation.

**Results**

Stimulation of the sciatic nerve was successful eliciting both muscle movement and EMG activity. Two distinct responses to stimulation were observed, a fast and slow activation (2.1 ± 2.6 ms and 18.8 ± 5.5 ms average delay following onset of FUS stimulation). The fast activation was accompanied by an average higher peak-to-peak EMG response than the slow responses (0.86 ± 0.87, 0.19 ± 0.63 mV respectively). EMG spike duration for both responses were similar and not significantly different (6.9 ± 2.6, 7.1 ± 2.2 ms respectively). Administration of lidocaine IM to the target region eliminated EMG responses from FUS stimulation. An hour following lidocaine administration EMG responses were detected again with FUS stimulation of the nerve. Electrical stimulation resulted in similar EMG spike durations to FUS stimulation, although at significantly higher peak-to-peak responses (7.3 ± 1.6 mV average). Gross pathology and behavioral testing revealed no significant damage (no red blood cell extravasation, significant difference in distance traveled, rotations or gap distance) with FUS stimulation at the powers used for stimulation.

**Conclusions**

In this study, we show physiological effects of *in vivo* FUS stimulation of peripheral nerves in the mouse for the first time. FUS stimulation can safely elicit muscle activation with no short-term behavioral effects. Positive controls verified EMG responses similar to that of electrical stimulation while negative controls verified FUS was stimulating the nerves and not the muscle tissue. These results support the further investigation of FUS-based techniques for the treatment of peripheral neurological disorders.

## A89 The mechanosensitive TRPC1 channel is activated by pulsed Focused Ultrasound to induce stem cell homing

### Scott Burks, Matthew Nagle, Ben Nguyen, Michele Bresler, Saejeong Kim, Blerta Milo, Joseph Frank

#### National Institutes of Health Clinical Center, Bethesda, Maryland, USA

**Objectives**

Stem cell therapies are promising regenerative medicine approaches. Pulsed Focused Ultrasound (pFUS) induces microenvironmental changes in normal and diseased tissues that enhance homing and efficacy of intravenously-infused mesenchymal stromal cells (MSC) to further improve disease outcomes. pFUS/tissue interactions that induce molecular changes is unclear. Mouse muscle and kidneys were sonicated at increasing powers while passive cavitation and tissue displacement were measured. Tissue was measured for cyclooxygenase-2 (COX2) to correlate with ultrasound effects, as COX2 signaling is critical for MSC homing. To test that mechanotransduction causes the necessary molecular changes, inhibitors of mechanosensitive channels were given before pFUS as well as pFUS to TRPC-knockout mice, as TRPC can function as a mechanosensitive receptor.

**Methods**

C3H or TRPC-ko mice received hamstring or kidney pFUS from a VIFU 2000 system. pFUS was at 1 MHz, 5 Hz pulse repitition frequency, 5 % duty cycle, and varying transducer output powers (ranging from 10-80 W). A built-in hydrophone passively detected cavitation. Mice were euthanized 4 hr post-pFUS and COX2 expression was measured by ELISA. GdCl3 (0.04mmoles/kg) or ruthenium red (RR) (0.01mmoles/kg) was given by intravenous injection before pFUS. One-way analysis of variance using Bonferroni post-hoc with p-values <0.05 were considered significant.

**Results**

**Fig. 91 (abstract A89).**
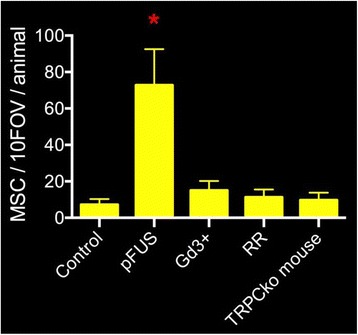
pFUS-induced MSC homing is blocked by inhibiting mechano-stretch receptors or in TRPC-ko mice

Statistically significant increases in COX2 were measured at 20, 40, 60, and 80 W compared to untreated muscle. Mean tissue displacement correlated with COX2 expression. Statistically significant increases in cavitation were only observed at 60 and 80 W. At 40 W, pFUS-induced COX2 increases and MSC homing were blocked in TRPC-ko mice or when Gd or RR were administered to wild-type mice before sonication (Fig. 91).

**Conclusions**

Mechanical pFUS forces interact with the mechanostretch receptor TRPC1 to drive molecular changes in tissue that are critical to stem cell homing processes. We have previously determined that COX2 expression is an acceptable proxy for molecular outcomes. At lower powers (20 and 40 W), cavitation from the sonications was not detectable, suggesting that cavitation-independent mechanical forces (i.e., acoustic radiation forces) drive COX2 expression and thus cell homing. At higher powers (60 and 80 W), cavitation was detectable and COX2 expression was elevated compared to sonications at 20 and 40 W. It is unclear whether cavitation at these elevated powers drives the additional COX2 expression, or if it is the result of increased acoustic radiation forces at higher powers. Regardless, 40 W was maximum power we previously determined not to cause detectable tissue damage and from the point of view of pFUS use in regenerative medicine, would be the maximum power.

## A90 Low-intensity ultrasound prolongs lifetimes of transplanted mesenchymal stem cells

### Scott Burks^1^, Matthew Nagle^1^, Saejeong Kim^2^, Blerta Milo^1^, Joseph Frank^1^

#### ^1^National Institutes of Health Clinical Center, Bethesda, Maryland, USA; ^2^National Institutes of Health, Bethesda, Maryland, USA

**Objectives**

Mesenchymal stem cells (MSC) are currently the most clinically applicable stem cell type for transplantation. Transplanted MSCs act as “drug-pumps” by continually releasing cytokines, chemokines, and trophic factors (CCTF) that reduce host inflammation and stimulate endogenous regeneration mechanisms. MSCs have shown promise for a wide range of diseases from ischemic events to graft-*versus*-host disease. While MSCs are considered immune privileged and evade immune surveillance to some extent, they typically do not engraft into host tissue and usually die within 3-10 days post-transplantation. Efforts to extend the lifetimes of MSCs previously have involved chemical or genetic manipulation of MSCs prior to transplantation, but these approaches are not readily translatable. We have found that pulsed Focused Ultrasound interacts with host tissues to upregulate a variety of anti-apoptotic, pro-mitotic factors that could increase the lifespan of transplanted MSCs. In this study we tested the hypothesis that the application of daily unfocused therapeutic ultrasound

**Methods**

106 human LMSCs were intramuscularly transplanted into each hamstring of C3H mice. MSCs were transfected with renilla luciferase using lentoviral vectors and expressed under the EF1 promoter. Beginning on the day of implantation (D1), mice received daily TUS sonication to one hamstring (1 MHz US, 2 W/cm2, 10 % duty cycle [1 ms “on”/9 ms “off”], 10 min total exposure time). After TUS, mice were subjected to bioluminescence imaging for 10s after receiving an intraperitoneal injection of D-luciferin (150 mg/kg). Mice were euthanized on D6. Temperature measurements were obtained in different group of mice using an implanted thermocouple.

**Results**

**Fig. 92 (abstract A90).**
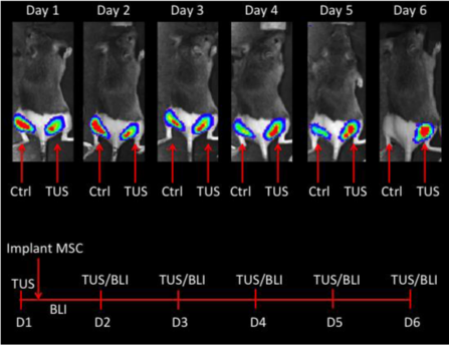
BLI signal decreases in control hamstrings (left) over 6 days while BLI signal in TUS-treated legs (right) is better preserved. Experimental timeline shown below

TUS exposures result in a combination of mechanical and thermal effects. TUS raised hamstring temperature from 36 to 41 during the 10 min exposure. Total bioluminescence in treated and control legs decayed each successive day, but decay was slower in the TUS-treated hamstrings. Accordingly, by D6, bioluminescence was nearly undetectable in untreated legs, while still imageable in TUS-treated legs (Fig. 92). The number of MSCs that remained in treated legs was nearly 10 times greater than in contralateral muscle.

**Conclusions**

The application of TUS to skeletal muscle containing transplanted MSCs extends the resident time of live MSCs in tissue by slowing MSC death/clearance rates. This has profound implications for a wide range of MSC therapies because TUS is noninvasive, safe, and inexpensive. Therefore, such a routine and uninvolved procedure to extend the survival of transplanted MSCs could easily and dramatically improve clinical cell therapies, extending the functional lifetimes of transplanted cells and reducing the number of transplantations/injections required for disease treatment.

## A91 Red blood cells as ultrasound-triggered drug delivery vehicles: Activation via perfluorocarbon nanodroplets, targeting and photoacoustic imaging

### Johnny Chen, Justin Farry, Adam Dixon, Zhongmin Du, Ali Dhanaliwala, John Hossack, Alexander Klibanov

#### University of Virginia, Charlottesville, Virginia, USA

**Objectives**

Red blood cells (RBC), when modified to operate as drug carriers, combine the biocompatibility of a patients’ own cells, long circulation time, large volume to sequester the drug, and ability to accumulate at the target sites (e.g., tumor vasculature) via molecular targeting. Recently point-of-care devices for RBC drug encapsulation have received CE approval mark in Europe. However, triggered release of the drug at the desired site is still complicated; therefore, we present in this study ultrasound-triggered release of entrapped materials from RBCs, by the preparation of RBC decorated with superheated perfluorocarbon (PFC) nanodroplets, and subsequent rupture of RBC membrane with contents release.

**Methods**

Fluorescent dyes, calcein and indocyanine green (ICG) and magnetic nanoparticles were loaded inside RBCs via hypotonic loading. After incubation in hypotonic media, the loaded RBCs were resealed by PIGPA buffer (adenine, glucose, inosine and pyruvate). Unincorporated material was removed by centrifugation (1000 g, 5 min). Nanoparticles made of decafluorobutane and dodecafluoropentane were prepared by sonication followed by the repeated Nuclepore filtration under elevated pressure; they were stabilized with a positively charged lipid shell, made of PEG stearate, DSPC and distearoyl trimethylammonium propane. Nanoparticles were attached externally to the membranes of dye-loaded RBCs. For molecular targeting, RBCs were decorated with biotin or alphaVbeta3-specific peptide cyclo-(RGDfK), via insertion of the molecules anchored to the cell membrane via a PEG spacer. To characterize the efficacy of ligand attachment, fluorescein-PEG-lipid was used as a model. Magnetic sensitivity of iron oxide-loaded RBCs was confirmed with a N52 neodymium magnet. Peptide-mediated targeting was confirmed in a solid phase binding system, where RGDfK-PEG-DSPE RBCs were incubated with polystyrene dishes carrying recombinant alphaVbeta3, or control albumin surface. Photoacoustic imaging of ICG-loaded RBCs was performed with a custom-built apparatus (Verasonics Vantage mated with a SpectraPhysics laser). Ultrasound-triggered contents release was monitored by fluorescence microscopy: dye-loaded RBCs carrying perfluorocarbon nanodroplets were subjected to insonation.

**Results**

Fluorescent dyes were loaded into RBCs with good stability (minor leakage over many hours). Fluorescent PFC nanodroplets demonstrated stable adherence to the RBC membrane. Upon a single ten-cycle 10 MHz ultrasound pulse, dye leaked from the RBC within a fraction of a second. Specific adhesion (~65-fold over control) of targeted RBCs to alphaVbeta3-coated surface was observed. Magnetic targeting demonstrated efficient pulling of magnetic RBCs in a tube and phantom, confirmed by photoacoustic imaging of ICG-loaded RBCs. RBC-nanodroplet complexes remained in the bloodstream for at least two hours following intravenous injection, as determined with Cr-51-radiolabeling.

**Conclusions**

Red blood cells decorated with perfluorocarbon nanodroplets rapidly release entrapped fluorescent dye after a short ultrasound pulse. As a drug carrier, modified RBCs stay in the bloodstream for extended time; they can be targeted by molecular or magnetic means, and monitored by photoacoustic imaging. Overall, PFC-RBCs may be an ideal drug carrier system for ultrasound-triggered drug delivery.

## A92 HIFU for targeted antibiotic delivery and therapy of chronic wounds and osteomyelitis

### Ashish Ranjan^1^, Danny Maples^1^, Rajiv Chopra^2^, Chenchen Bing^2^, Robert Staruch^3^, Rachel Wardlow^1^, Michelle Wodzak Staruch^4^, Jerry Malayer^1^, Akhilesh Ramachandran^1^, Joris Nofiele^2^

#### ^1^Oklahoma State University, Stillwater, Oklahoma, USA; ^2^University of Texas Southwestern Medical Center, Dallas, Texas, USA; ^3^Philips Research, Dallas, Texas, USA; ^4^University of Oklahoma Health Sciences Center, Oklahoma City, Oklahoma, USA

**Objectives**

**Fig. 93 (abstract A92).**
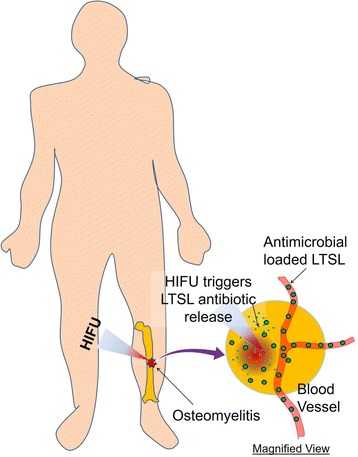
Development of low temperature sensitive liposomes for HIFU guided localized delivery of antimicrobial against chronic wound

Chronic non-healing wound infections require long duration antibiotic therapy, and are associated with significant morbidity and healthcare costs. This results in both systemic and local infection to deeper tissues (e.g bones), thereby requiring long duration treatment (generally >6 weeks), resection of tissues, and emergence of drug resistance. Novel approaches for efficient, readily-translatable targeted and localized antimicrobial delivery, as well as targeted ablation of pathogens are a critical clinical need. The objectives of this study were to: 1) develop low temperature-sensitive liposomes (LTSLs) containing an antimicrobial agent (ciprofloxacin) for induced release at mild hyperthermia (~42 °C) (Fig. 93), 2) characterize *in vitro* ciprofloxacin release, and efficacy against Staphylococcus aureus plankton and biofilms, and 3) determine the feasibility of localized ciprofloxacin delivery in combination with MR-HIFU hyperthermia in a rat model.

**Methods**

LTSLs were loaded actively with ciprofloxacin, and their efficacy was determined using a disc diffusion method, MBEC biofilm device, and scanning electron microscopy (SEM). Ciprofloxacin release from LTSLs was assessed in a physiologic buffer (serum and PBS), and *in vivo* in a rat model using MR-HIFU by fluorescence spectroscopy.

**Results**

Results indicated that > 95 % loading of ciprofloxacin was achieved. Further, < 5 % was released from the LTSL within 15 min at baseline (25 °C) and body (37 °C) temperature, while > 95 % was released at 42 °C. Sonication of rat muscles resulted in accurate and homogeneous temperature control within the heated ROI (42.0 ± 0.2 °C), with a 90th percentile (T10) and 10th percentile (T90) of 43.2 ± 0.3 °C and 41.0 ± 0.3 °C respectively

Precise hyperthermia exposures in the thigh of rats using MR-HIFU during IV administration of the LTSLs resulted in a 4-fold greater local concentration of ciprofloxacin compared to controls (free ciprofloxacin + MR-HIFU or LTSL alone). The biodistribution of ciprofloxacin in unheated tissues was fairly similar between treatment groups. Triggered release at 42 °C from LTSL achieved significantly greater S. aureus killing and induced membrane deformation and changes in biofilm matrix compared to free ciprofloxacin or LTSL at 37 °C.

**Conclusions**

This technique has potential as a method to deliver high concentration antimicrobials to chronic wounds.

## A93 Effectiveness of MR-guided Focused Ultrasound treatment for the tender points in patients with painful bone metastasis or with chronic osteoarthritic pain

### Hirofumi Namba^1^, Motohiro Kawasaki^1^, Masashi Izumi^1^, Katsuhito Kiyasu^1^, Ryuichi Takemasa^1^, Masahiko Ikeuchi^1^, Takahiro Ushida^2^

#### ^1^Kochi University, Kochi Medical School, Nankoku, Kochi, Japan; ^2^Multidisciplinary Pain Center, School of Medicine, Aichi Medical University, Nagakute, Aichi, Japan

**Objectives**

MR-guided Focused Ultrasound surgery (MRgFUS) has been recently demonstrated as a promising tool for pain alleviation of painful bone metastases (pBM) and lumbar facet joint pain. The mechanisms of pain alleviation are believed to be most likely local denervation of the nociceptors at the ablated area using the Focused Ultrasound. However, few previous studies have suggested an assessment method to estimate the denervation. Peripheral nociceptive sensitization resulting in bone metastatic pain and osteoarthritic pain is thought to make pain on pressure (tenderness) at the lesion sites too sensitive. Therefore, our hypothesis is that MRgFUS treatment of the sensitized tender points could improve the refractory chronic pain in patients with bone metastasis or osteoarthritis (OA). The aim of the present study is to clarify changes in the tenderness and the worst pain intensity at the lesion treated with MRgFUS.

**Methods**

We have conducted MRgFUS treatments using ExAblate® system (InSightec Ltd, Haifa, Israel) for 8patients (mean age; 64 years old) with pBM, 9 patients (72 y.o.) with chronic lumbar facet joint osteoarthritic pain (LFP), and 11 patients (78 y.o.) with chronic knee osteoarthritic pain (KP). Patients with the worst pain intensity (worst) ≥ 4 on the Numerical Rating Scale (NRS) and tenderness at the lesion site were enrolled. Treatments were applied to the bone surface of the most painful tender point of the metastasis in pBM group, the dorsal area of these joints which selected on the basis of 70 % reduction of pain with diagnostic block in LFP group, the bone surface around osteophyte of medial femorotibial joint tenderness in KP group. We assessed pressure pain threshold (PPT) using an electronic pressure algometer to evaluate tenderness at the lesion sites, and the subjective local worst pain intensity with NRS. PPT is defined as the minimal amount of pressure where a sensation of pressure first change to pain. We evaluated changes in the PPT on the treated site and at the contralateral non-treated site as a control and NRS before and 1 week, 1 month and 3 months after the treatments.

**Results**

PPTs at the treated sites were significantly increased from 107 kPa [40-432] (median [min-max]) at the baseline to 271 kPa [94-534] at the final follow-up in pBM group, 280 kPa [66-427] to 462 kPa [207-818] in OALFJP group, and 156 kPa [50-249] to 246 kPa [1146-427] in OAKP group. At the control sites, the PPTs were significantly higher than at the treated sites and there was no significant difference of PPTs between before and after the treatment in all groups. The NRS were significantly decreased from 6.5 [4-8] to1 [0-3] in pBM group, 8 [4-9] to 1.5 [0-7] in LFP group, and 6 [5-9] and 3 [1-6] in KP group. The treatment responder, defined as reduction of 2 or more in the NRS without increase analgesic intake in pBM, and a 50 % or greater decrease in the NRS in OA group, were all patients in pBM group, 14 of 20 patients in OA group. In OA group, the PPTs at the treated sites were significantly increased from 223 kPa [50-427] to 326 kPa [184-731] at 1 week after the treatment in responders, but there were no significant differences in non-responders.

**Conclusions**

These results illustrate the excellent pain-relieving effects of MRgFUS treatment for the tender points in patients with painful bone metastasis or with chronic osteoarthritic pain. Significant increase of PPTs speculated successful denervation effect on the nociceptive nerve terminals of the treated area. PPTs measurement is a useful tool for quantitative evaluation after MRgFUS and might be a useful parameter for assessing a treatment’s effect.

## A94 Passive acoustic mapping and MR thermometry for real-time multi-modality imaging of Focused Ultrasound ablation

### Calum Crake^1,2^, Iason T. Papademetriou^3^, Yong-Zhi Zhang^2^, Tyrone Porter^3^, Nathan McDannold^1,2^

#### ^1^Brigham and Women’s Hospital, Boston, Massachusetts, USA; ^2^Harvard Medical School, Boston, Massachusetts, USA; ^3^Boston University, Boston, Massachusetts, USA

**Objectives**

MRI provides outstanding soft-tissue contrast and ability to map temperature which are ideal for monitoring of Focused Ultrasound therapy. However, at the high intensities required for ablation acoustic cavitation may be nucleated, leading to an enhancement in ultrasound-mediated heating. The likelihood of cavitation may also vary significantly between adjacent tissue regions, which has frustrated efforts to control ablation over large volumes. As the onset of cavitation is accompanied by characteristic acoustic emissions, simultaneous acoustic mapping during treatment can provide critical information that may used for control of ultrasound output to achieve ablation of entire tumors. Previous work has shown that such monitoring is feasible within an MR scanner using post-processed ultrasound data. The objective of the current study was to optimize ultrasound data acquisition and processing to allow rapid real-time display of acoustic mapping data alongside MR thermometry.

**Methods**

**Fig. 94 (abstract A94).**
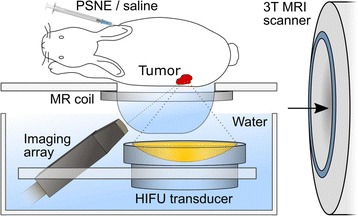
Setup for multi-modality imaging of focused ultrasound ablation

An ultrasound imaging array was aligned with the focus of a 1.5 MHz HIFU transducer (Fig. 94). The two components were clamped together, immersed in degassed water, and connected to a MR-compatible positioning stage. Rabbits with subcutaneous VX2 tumors were anaesthetized and placed on a platform with a cutout for ultrasound propagation and acoustically coupled using a bag filled with degassed water. A receive-only MR surface coil was placed in the air gap between the water and animal platform. The entire setup was placed within a clinical 3 T MR scanner (GE Signa Excite). The HIFU transducer was then driven from a waveform generator via a power amplifier. During sonication the MR scanner performed temperature mapping while an ultrasound research platform (Verasonics) performed B-mode and passive acoustic mapping (PAM) using the array. Temperature maps, B-mode and passive acoustic images were displayed in real time. The process was repeated in rabbits injected with phase-shift nanoemulsions (PSNE) to promote cavitation or saline as a control under a range of ultrasound conditions.

**Results**

**Fig. 95 (abstract A94).**
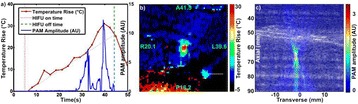
**a** Passive acoustic mapping (PAM) and MR-derived temperature rise over time. **b** MRI temperature map and (**c**) acoustic map for cavitation-enhanced focal heating

Real time passive acoustic mapping was successfully implemented using a rapid frequency-domain beamformer and employed simultaneously with MR thermometry for imaging of tumor ablation. Illustrative results are shown in Fig. 95. Over 50s (40s sonication, 10s baseline and cooling time) 477 frames of ultrasound data were acquired, processed and immediately displayed at an average frame rate of 10 fps. MR temperature maps were also acquired and displayed every 2.3 s (0.4 fps). Comparison of the two modalities (Fig. 95a) shows accelerated temperature rise following the onset of cavitation (30s) and decline thereafter as the amplitude of cavitation dropped (40s), perhaps due to depletion of cavitation nuclei or changes in tissue structure. MR temperature maps (Fig. 95b) and passive acoustic maps (Fig. 95c) show that heating and cavitation activity were both well localized to the intended focal region. Extraction of tumors after sonication showed lesions were successfully produced in the tumors.

**Conclusions**

These results show that rapid, real-time passive acoustic imaging coupled with PSNE for nucleating cavitation locally may be applied simultaneously with MR thermometry for enhancing Focused Ultrasound ablation. Future work will apply the method for optimization and control of PSNE enhanced ablation of whole tumors.

## A95 Acoustic characterization of a clinical MR-guided HIFU system inside and outside MR scanner

### Satya V. V. N. Kothapalli^1^, Wan Leighton^1^, Zhaorui Wang^1^, Ari Partanen^2^, H. Michael Gach^1^, William Straube^1^, Michael Altman^1^, Hong Chen^1^

#### ^1^Washington University in Saint Louis, Saint Louis, Missouri, USA; ^2^Philips, Bethesda, Maryland, USA

**Objectives**

With the rapid advancement of MR-guided HIFU clinical applications, calibration of HIFU pressure fields is an urgent need to ensure consistent and safe treatment outcomes. Few studies have been reported on the characterization of acoustic pressure fields of clinical MR-guided HIFU outside the MR scanner. To date, no study has been reported on acoustic field calibration inside the MR scanner, owing to combined effects of strong magnetic field and limited space in the MR bore that makes it difficult to perform the measurements inside the MR scanner. In this study, we present a method of acoustic field calibration and results for a clinical MR-HIFU system both inside and outside a clinical MR scanner.

**Methods**

**Fig. 96 (abstract A95).**
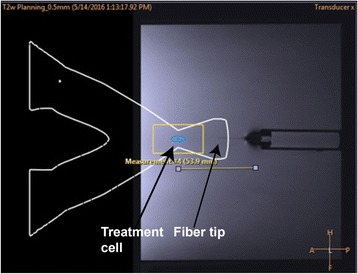
The location of fiber tip inside the water tank in the MR scanner was identified precisely with the help of MR imaging and the HIFU transducer was moved to the corresponding location for the acoustic field calibration

For hydrophone measurement outside the MR scanner (Ingenia 1.5 T, Philips), the HIFU patient table with an integrated 256-element transducer (Sonalleve V2, Philips) was moved outside the magnet room. A fiber-optic hydrophone (HFO-690, Onda) attached to a 3D motor stage was used for pressure field measurements in a deionized and degassed water tank placed above the transducer. A Matlab® program was developed to control the 3D stage, capture the hydrophone signals, and synchronize the data acquisition with the HIFU pulses. For measurements inside the MRI, an MRI-compatible water tank, a 20-meter long optical fiber was used for the hydrophone measurements and it was visualized in MR imaging (Fig. 96). The HIFU transducer was moved in 3D space while keeping the optical fiber fixed. The HIFU transducer was operated at a frequency of 1.2 MHz, a pulse length of 40 cycles and pulse repetition frequency of 10 Hz. The nominal acoustic power was varied from 50-650 W. Hydrophone measurements were performed in planes parallel and perpendicular to the beam path. The acoustic parameters such as peak positive/negative pressures (p+, p-), temporal and pulse average intensities (ITA, IPA) at the HIFU focus, and the full-widths of the pressure distribution along and perpendicular to the bean path were calculated.

**Results**

**Fig. 97 (abstract A95).**
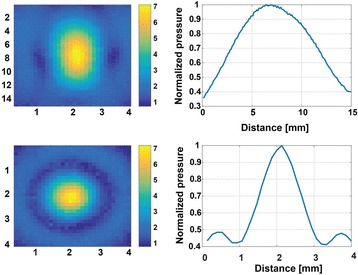
Results of pressure field measurements in YZ (along) (top left) and XY (perpendicular) (bottom left) planes to beam paths and right hand side plots are the normalized pressures with respect to distances to find the FWHM of the pressure distribution

**Fig. 98 (abstract A95).**
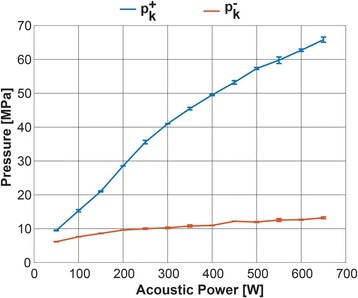
Peak positive and negative pressures at the HIFU transducer focus with respect to acoustic power between 50-650 W

Outside MRI Scanner: The HIFU focus was identified approximately 6.8 cm away from membrane (when the transducer was at the homing position and without steering) by scanning the hydrophone in planes parallel and perpendicular to the beam path (Fig. 97; left-hand side). The acoustic parameters p + (3.4 - 65.8 MPa), p- (3.1 - 13.2 MPa) (Fig. 98), ITA (0.06 - 6.2 W/cm2), IPA (78.5-20159 W/cm2) at the focus were measured with respect to nominal acoustical power (50-650 W). The FWHM of pressure profiles at the focus was 1.0 mm in the lateral direction and 9.5 mm in the axial direction (Fig. 97; right-hand side).

Inside MRI scanner: The acoustic pressure measurements inside MR scanner were found consistent with the outside measurements. The motor step-size of the HIFU transducer was 0.1 mm which allowed high spatial resolution scanning of the acoustic field inside the MR scanner.

**Conclusions**

In conclusion, HIFU pressure field measurements both outside and inside an MRI scanner provides a systemic method for ensuring safe and consistent treatment in a clinical MR-guided HIFU system. Moreover, the proposed inside MR scanner calibration method allowed characterization of the acoustic fields of a clinical MR-guided HIFU system without the need of moving the HIFU table outside of MR scanner, which is not feasible for most clinical systems.

## A96 Preservation of endometrium after Magnetic Resonance Imaging-guided High-Intensity Focused ultrasound (MR-HIFU) ablation for uterine fibroids of submucosal type

### Young-sun Kim, Hyo Keun Lim, Hyunchul Rhim

#### Samsung Medical Center, Seoul, Republic of Korea

**Objectives**

To evaluate the endometrial integrity after MR-HIFU ablation for submucosal uterine fibroids based on MRI findings and to know the risk factors associated with endometrial injury

**Methods**

A total of 117 submucosal uterine fibroids (diameter: 5.9 ± 3.0 cm, 1.0-12.9 cm) in 101 women (age: 43.6 ± 4.4 year, 33-55 year) treated with volumetric MR-HIFU ablation between November 2010 and December 2015 were retrospectively analyzed. Endometrial integrity was assessed with contrast-enhanced T1WI of immediate (n = 101), 3-month (n = 62), and 12-month (n = 15) follow-up. Endometrial injury was classified into one of grade 0 (continuous involved endometrial lining), grade 1 (pin-point, full-thickness discontinuity of involved endometrium), grade 2 (between grade 1 and 3) and grade 3 (full-thickness discontinuity of involved endometrium over 1 cm in size). Potential risk factors for endometrial injury (age, duration from LMP, history of full term delivery, GnRH agonist pretreatment, FIGO grading of endometrial protrusion, T2 signal intensity and perfusion degree of fibroid, and average acoustic power) were assessed with a generalized estimating equation (GEE) analysis.

**Results**

**Fig. 99 (abstract A96).**
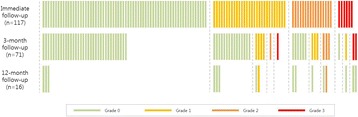
Follow-up results of endometrial injury after MR-HIFU ablation for submucosal uterine fibroids

**Fig. 100 (abstract A96).**
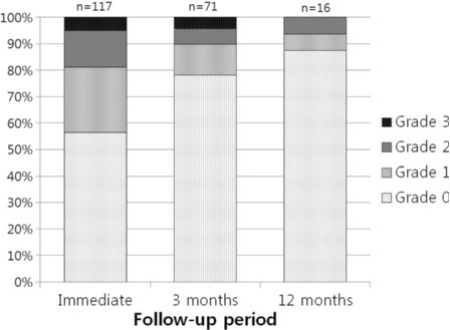
Follow-up results of endometrial injury after MR-HIFU ablation for submucosal uterine fibroids

Among 117 fibroids, endometrial injury was of grade 0 in 66 (56.4 %), grade 1 in 29 (24.8 %), grade 2 in 16 (13.7 %), and grade 3 in 5 (4.3 %) at immediate follow-up (Fig. 99). Among 37 fibroids of which endometrium was injured and underwent follow-up studies, 30 (81.1 %) showed improvements at 3- and/or 12-month follow-up. Among 14 fibroids in which endometrium was injured and followed-up till 12 months, 12 (85.7 %) were completely normalized (Fig. 100). GEE analysis revealed that the degree of endometrial protrusion were significantly associated with severer endometrial injury (p < 0.0001, B = -0.687).

**Conclusions**

After MR-HIFU ablation of submucosal uterine fibroids, endometrium is preserved or minimally injured in majority of the cases. Injured endometrium which is more frequently encountered after treating intracavitary fibroids usually recovers spontaneously.

## A97 Responses of uterine fibroids to Gonadotropin-Releasing Hormone agonist (GnRHa) as a pretreatment of MR-guided High-Intensity Focused Ultrasound (MR-HIFU) ablation

### Young-sun Kim, Hyo Keun Lim, Hyunchul Rhim

#### Samsung Medical Center, Seoul, Republic of Korea

**Objectives**

To evaluate changes in MR findings of uterine fibroids after using GnRHa as a pretreatment of MR-HIFU ablation and to know the influencing factors for favorable changes to MR-HIFU ablation

**Methods**

From August 2012 to March 2016, 60 women (age: 43.6 ± 5.7 years, 26-54 years) with 127 uterine fibroids (diameter, 5.9 ± 3.0 cm; volume, 121.1 ± 162.3 mL) underwent GnRHa injections as a pretreatment of MR-HIFU ablation (1-4 cycles; 5 women with 10 fibroids, 8 women with 17 fibroids, 40 women with 87 fibroids, and 7 women with 13 fibroids, respectively) and retrospectively analyzed. Fibroid volumes and T2 signal intensity ratios relative to skeletal muscle were compared between before and 2-3 weeks after the last GnRHa injection. Potential factors (age, BMI, history of full term delivery, multiplicity of fibroids, number of GnRHa injections, volume, T2 signal intensity ratio, and semiquantitative perfusion MR parameters of fibroid) were evaluated for the association with greater volume and T2 signal intensity changes. All statistical tests were performed using with model analyses.

**Results**

While fibroid volume significantly reduced after GnRHa therapy (volume reduction ratio = 74.8 ± 25.3 %, p < 0.001), T2 signal intensity ratios did not show a significant difference (T2 signal change ratio = 102.0 ± 32.2 %, p = 0.257). Increased number of GnRH injection (B = -0.067, p = 0.028), greater T2 signal intensity ratio (B = -0.014, p = 0.028) and greater wash-in rate (B = 0.0006, p = 0.012) were revealed to be independently significant for greater volume reduction of fibroids. However, no factor turned out to be associated with T2 signal intensity change.

**Conclusions**

After using GnRHa as a pretreatment of MR-HIFU ablation, uterine fibroids with higher T2 signal intensity and/or greater wash-in rate in semiquantitative perfusion MRI show greater volume reduction, of which effect is strengthened with an increased number of GnRHa cycles. T2 signal intensity does not show a significant change after GnRHa therapy.

## A98 Targeted vessel ablation with MR-HIFU for the treatment of type 3 uterine fibroids

### Johanna van Breugel, Manon Braat, Chrit Moonen, Maurice van den Bosch, Mario Ries

#### University Medical Center – Utrecht, Utrecht, Netherlands

**Objectives**

Type 3 uterine fibroids are considered highly perfused and/or to have a high extracellular fluid content and are known to have poor treatment outcome in terms of non-perfused volume (NPV), volume reduction, symptom relief and an increased risk of adverse events. Type 3 fibroids have therefore been excluded form HIFU treatment at our institutionfor the last two years. However, a novel approach using higher acoustic power to precisely target the feeding vessels might result in vessel occlusion rendering the bulk tissue less perfused and more susceptible to ‘lower’ powers, which in turn might lead to better treatment outcomes. Therefore, the aim of this research is the feasibility and safety of high power targeted vessel ablation of type 3 fibroids with MR-HIFU.

**Methods**

In this study 10 patients with a type 3 fibroid will be included. Screening MR scans include a T2w-scan to determine fibroid type, a dynamic contrast enhanced (DCE) series to assess perfusion, and a time-spatial labeling inversion pulse (TimeSLIP) sequence to visualize the vasculature without using a contrast agent. Areas of blood inflow are determined based on the DCE and TimeSLIP and are marked. The TimeSLIP is repeated prior to treatment, spatially matched with the screening DCE and TimeSLIP sequence, and used for planning of the high power (450 W) sonications. A Philips Sonalleve V2 system with skin cooling (water temperature ~ 15C) is used. After sonicating the inflow areas with high power cells the bulk volume is treated with ‘low power’ sonications (£300 W). NPV is assessed on contrast-enhanced T1w-images. Follow-up MRI is performed after three months.

As a preparation a patient with a type 2 fibroid was treated in a similar manner except that all sonication were at a power £300 W. Philips software was used to determine areas with a high wash-in rate (WIR) indicating the areas of early inflow and these regions were treated.

**Results**

**Fig. 101 (abstract A98).**
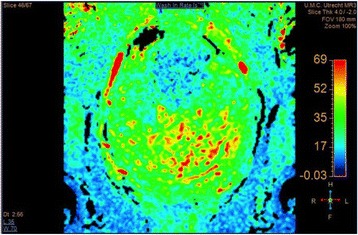
Wish-in-rate map shown in a coronal view. The lower (caudal) part of the fibroid shows higher inflow rates

**Fig. 102 (abstract A98).**
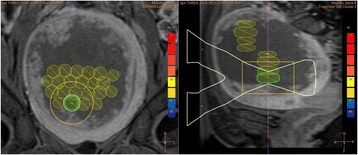
Left: coronal view Right: sagittal view of the treatment cells overlaid on the contrast-enhanced T1w-image showing the NPV (dark region). The NPV is much larger than the planned treatment volume most likely due to vessel occlusion by the sonications

The WIR map of the type 2 fibroid showed areas of higher inflow (Fig. 101). These regions were targeted with 12/14 mm sonications (maximum power of 230 W, 42-55 s). The resulting NPV was larger than the planned treatment volume (PTV) (Fig. 102).

**Fig. 103 (abstract A98).**
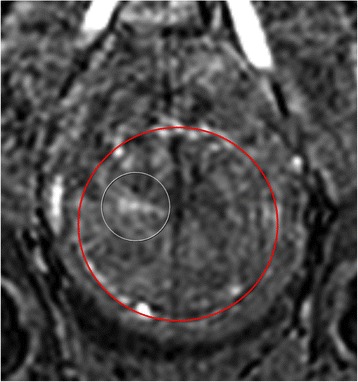
Dynamic contrast enhanced image showing the fibroid in the red circle and one of the targeted blood-inflow areas in the white circle

**Fig. 104 (abstract A98).**
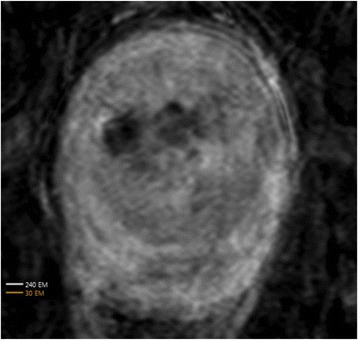
Contrast enhanced T1w-image showing the two non-perfused volumes created by the twp clusters of high power sonications

So far, one type 3 fibroid has been treated. Two inflow areas were determined (Fig. 103) and seven treatment cells (4 mm) were planned in each of these areas. Sonications were completed successfully. Hereafter, 8/12 mm volumetric feedback cells (max. 250 W) were used. Two NPVs corresponding to the two high-power clusters were observed post treatment (Fig. 104). Small NPVs (2-3 mm) could be observed in the bulk tissue volume. However, no confluent NPV similar in volume to the PTV could be observed. Three-month follow-up has not yet been performed. No adverse events occurred during treatment.

**Conclusions**

The results of the type 2 fibroid treatment using MR-HIFU showed the potential of targeted vessel ablation. A similar strategy was used for type 3 fibroids. Based on the first treatment the high power sonications might result in a non-perfused volume in type 3 fibroids. More data are expected soon and are needed to draw conclusions on the feasibility and safety of this approach.

## A99 A non-contrast method for evaluating non-perfused volume after MRgFUS treatment of uterine fibroids

### Cristina Marrocchio^1^, Susan Dababou^1^, Rachelle Bitton^2^, Kim Butts Pauly^2^, Pejman Ghanouni^2^

#### ^1^University of Rome – La Sapienza, Rome, Italy; ^2^Stanford University, Stanford, California, USA

**Objectives**

**Fig. 105 (abstract A99).**
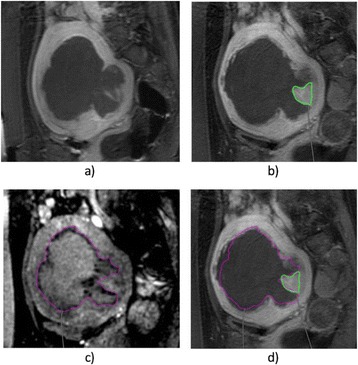
Comparison of **a**) NPV1 and **b**) NPV2 demonstrates an area of reperfusion (green outline). The BLV contour (**c**, pink), matches the NPV2 contour (**d**, pink), and excludes the reperfused contour (green) in Patient 3

The efficacy of MRgFUS treatment of uterine fibroids is evaluated post-operatively by measuring the non-perfused volume (NPV) on post-contrast images as an estimate of the ablated region. NPV is not a direct measurement of ablation but rather of non-perfused tissue. This may lead to an underestimate of the ultimately ablated volume, as some cell death may be delayed, or to an overestimate of the treatment effect, for example, in cases where temporary vasoconstriction contributes to the non-perfused volume (Fig. 105a,b). In addition, this can be confusing in cases where non-targeted tissue appears non-perfused; since there is no thermal dose predicted in these regions, it may be unclear whether the tissue is actually ablated.

A hypointense contour, which we call the Black-Line Volume (BLV), is present around the margin of fibroids on non-contrast images obtained at follow-up (Fig. 105c). Our aim was to quantitatively compare the BLV to the NPV.

**Methods**

Five patients with follow-up imaging after treatment were included in this study. Four of them had a second treatment within 7 to 14 days either because the fibroid was too large for a single treatment, or it appeared incompletely ablated. Patient 2 had no additional treatment. In three of the retreated patients, a reperfused volume was noted on follow-up images in regions that the NPV indicated as non-perfused at the end of the first treatment

The NPVs obtained at the end of each treatment (NPV1 and NPV2) and the reperfused volumes were measured and compared by multiplying slice-by-slice measurements of the manually contoured area by the slice thickness. Using the same methodology, the BLV was calculated using EPI thermometry magnitude images acquired during each sonication in follow-up images.

**Results**

The results are provided in Table 5. In these patients, the BLV contour is not present prior to treatment, but is reliably visualized on follow-up imaging obtained 7-43 days after treatment. Quantitation of the BLV demonstrates that it is very similar to the NPV. In cases where the BLV was less than NPV1, the discrepancy is explained by reperfusion (Fig. 105d).

In three cases, at the end of a second treatment, portions of the fibroid that were part of NPV1 were seen to have reperfused. In these cases, BLV consistently identified the reperfused volume, which was outside of the BLV.

**Conclusions**

These results show that BLV correlates well with the ablated region, and the non-perfused volume. In cases in which NPV1 overestimated the truly ablated volume, as defined during the follow-up imaging, BLV was a better predictor because it excluded the reperfused volume. Thus, BLV may prove to be a powerful tool to disclose inadequately treated regions during the planning session of a second treatment, allowing more tailored targeting of the area to ablate and preventing under-treatment.

We are currently assessing how soon after the initial treatment the BLV becomes visible. If further research demonstrates a BLV at the end of the first treatment, then it may be used to identify untreated parts of the fibroid and guide further, more complete treatment without contrast administration.

**Table 5 (abstract A99).** Comparison of non-perfused volume after first fibroid treatment (NPV1) and black line volume (BLV) observed on follow-up imaging

Patient	NPV1 (cc)	Days to Follow Up Imaging	BLV on Follow Up (cc)	Reperfused Volume on Follow Up (cc)
1	412	14	419	none
2	42	43	42	none
3	314	14	295	16
4	257	7	208	88
5	85	14	82	5

## A100 Portable Ultrasound-guided High Intensity Focused Ultrasound with 3D electronic steering and targeting forecast function: 2-Year prospective clinical trial for uterine fibroids

### Jae Young Lee^1^, Jae Young Lee^1^, Hyun Hoon Chung^1^, Soo Yeon Kang^1^, Kook Jin Kang^2^, Keon Ho Son^2^

#### ^1^Seoul National University Hospital, Seoul, Republic of Korea; ^2^Alpinion Medical Systems, Seoul, Republic of Korea

**Objectives**

Not released for publication

**Methods**

Not released for publication

**Results**

Not released for publication

**Conclusions**

Not released for publication

## A101 Preliminary clinical evaluation of Magnetic Resonance guided Focused Ultrasound Surgery (MRgFUS) combined with GnRH-a analog therapy in the treatment of diffuse adenomyosis

### Dandan Zhang

#### The First Affiliated Hospital of Harbin Medical University, Harbin, China

**Objectives**

Adenomyosis is the common gynecologic disorder that in the presence of endometrial glands and stroma deep within the myometrium. Treatment of diffuse Adenomyosis has always been difficult, especially for patients who desire subsequent pregnancy. GnRH-a is a ovarian suppression drug that has been effectively used for Adenomyosis, while as long time use will result in symptoms of low estrogen, it is not recommended for use more than 6 months. In previous reports, symptoms recurred after stopping the drugs, therefore GnRH-a is often used as an adjuvant prior to or after the surgery. MRgFUS is safe and feasible for the treatment of focal Adenomyosis, while it showed limited efficacy in the treatment of diffuse Adenomyosis. In this study, the preliminary clinical effects of combined MRgFUS and GnRH-a therapy in the treatment of diffuse Adenomyosis will be evaluated, and the fertility and pregnancy outcomes for patients who desired pregnancy will be investigated.

**Methods**

9 patients with diffuse Adenomyosis were treated in Harbin MRgFUS Center between April 2015 and May 2016. Including, 7 patients (1 patient has fertility requirements) received MRgFUS treatments only, while the rest 2 patients (1 patient has fertility requirements) received 3 months of GnRH-a prior to MRgFUS treatment. The treatment response was evaluated by the symptom severity score (SSS, 0-100), visual analogue scale score for assessment of pain (VAS, 0-10) and Pictorial Blood Loss Assessment Chart (PBAC). The questionnaires were evaluated on treatment day as well as before, 3 and 6 months after treatment. Uterine volume reduction was recorded before and 6 months after the treatments.

**Results**

During the 6-month follow up, 89 % (8/9) of the patients showed significant symptomatic relief (based on SSS and PBAC) and 67 % (6/9) of the patients experienced pain reduction (based on VAS). The statistic uterine volume reduction was 14.5 ± 1.2 % in patients treated with MRgFUS only. On the contrary, uterine volume reduction of 20.3 ± 1.8 % was observed in patients treated with MRgFUS combined with GnRH-a. 1 patient underwent single session of MRgFUS treatment had relapse 6 M after MRgFUS and enlarged uterus was found on FU and MR exam. Up to now, the 2 patients that desired fertility are still not pregnant, this study will extend the follow up period to 24 months.

**Conclusions**

MRgFUS combined with GnRH-a can potentially prevent recurrences after solely GnRH-a analog therapy, and can also enhance the treatment efficacy of MRgFUS for diffuse Adenomyosis. MRgFUS is capable of preserving the functionality of myometrial structures, hence may reduce the length of duration before pregnancy. MRgFUS is a very promising technique for Adenomyosis patients who desired fertility, while its short and long term impacts in the fertility and pregnancy outcomes are still required.

## A102 Endoluminal ultrasound applicators for thermal ablation of pancreatic cancer under MR-guidance: Preliminary investigations in an in vivo porcine model

### Matthew Adams^1^, Vasant Salgaonkar^1^, Juan Plata^2^, Peter Jones^1^, Aurea Pascal-Tenorio^2^, Donna Bouley^2^, Graham Sommer^2^, Kim Butts Pauly^2^, Chris Diederich^1^

#### ^1^University of California San Francisco, San Francisco, California, USA; ^2^Stanford University, Stanford, California, USA

**Objectives**

While conventional treatments remain limited, thermal ablation has been demonstrated to provide pain palliation and survival benefit to patients with advanced pancreatic cancer. In light of the anatomical challenges that limit safe and effective thermal ablation to the pancreas, endoluminal ultrasound may serve as a minimally invasive option for treating pancreatic tumors adjacent to the gastrointestinal (GI) tract, and could be compatible with MR-guidance for target confirmation and real-time thermometry. This study’s objective was to perform a preliminary investigation of endoluminal placement and heating performance of prototype ultrasound applicators and temperature monitoring capabilities in MR-guided *in vivo* porcine pancreas ablations.

**Methods**

**Fig. 106 (abstract A102).**
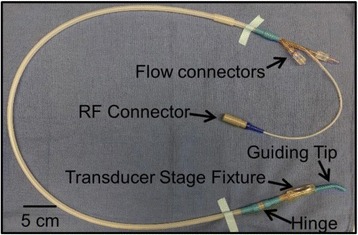
Prototype endoluminal ultrasound applicator assembly

A family of MR-compatible endoluminal applicators were fabricated and characterized using radiation force balance and hydrophone measurements. Each applicator possessed a distinct two-element transducer array (~3.2 MHz) covered by a cooling balloon at the tip of a flexible catheter assembly, as shown in Fig. 106. Three transducer geometries were considered: 10x10 mm (planar), 8x10 mm with 20 mm radius of curvature (ROC) along the short dimension (lightly-focused curvilinear), and 9.3x11.4 mm with 25 mm ROC along the long axis (strongly-focused curvilinear). In each *in vivo* porcine experiment, an applicator was orally introduced and positioned in the stomach adjacent to the splenic lobe of the pancreas. Sonications were performed through the luminal wall into the pancreas for ablation, at durations ranging from 3-16 minutes and applied intensities of 5-7 W/cm2. MR-guidance techniques were developed and evaluated for anatomical target identification, tracking/placement of the applicator, and PRF-based MR temperature imaging (MRTI), implemented in the real-time RTHawk software environment.

**Results**

**Fig. 107 (abstract A102).**
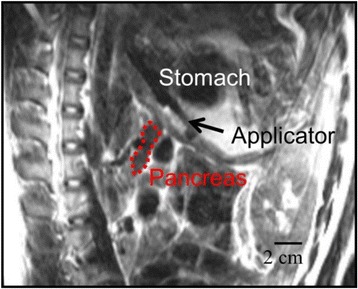
T2-weighted MRI image showing placement of endoluminal applicator in porcine stomach and alignment for treatment of pancreatic tissue

**Fig. 108 (abstract A102).**
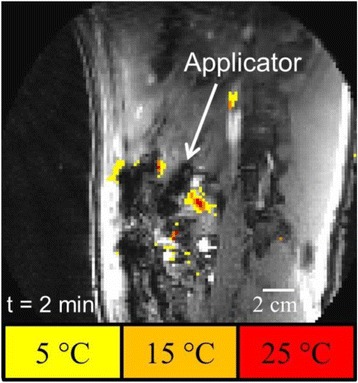
MRTI temperature map for in vivo porcine pancreatic ablation using an endoluminal ultrasound applicator with strongly focused curvilinear elements, at 6 W/cm2 applied intensity

**Fig. 109 (abstract A102).**
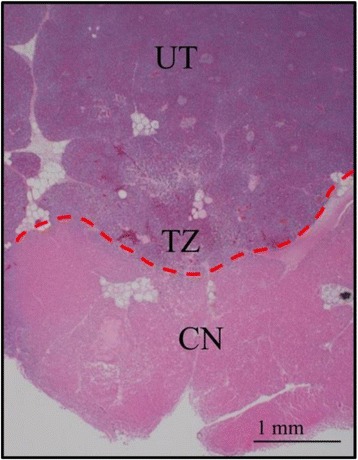
H&E staining of a section of excised pancreatic tissue following endoluminal ablation, showing regions of coagulative necrosis (CN) and the transition zone (TZ) to untreated tissue (UT)

Placement of the endoluminal applicators in the stomach adjacent to pancreatic tissue, as demonstrated in Fig. 107, was achieved in all porcine studies (n = 5). *In vivo* MRTI-guided heating trials demonstrated capability of ~15-20 °C temperature elevation in pancreatic tissue at 1-2 cm depths using the planar and lightly-curvilinear applicators (6-16 mins, 5-7 W/cm2). Strongly-focused curvilinear applicators were capable of reaching higher temperature elevations of ~25-35 °C at 2-3 cm depths and more spatially localized lesions in shorter treatment durations (3-5 mins, ~5 W/cm2), as shown in Fig. 108. Dimensions of thermal lesions in excised pancreas ranged from 9-28 mm, 3-10 mm, and 5-10 mm in length, width, and depth, respectively, as verified through histopathological analysis of tissue sections (Fig. 109). Multiple-baseline reconstruction and respiratory-gated acquisition were effective in suppressing motion artifacts and improving temperature precision for multi-slice MRTI in the *in vivo* studies.

**Conclusions**

This study demonstrates the technical feasibility of generating volumetric ablation in porcine pancreatic tissue using endoluminal ultrasound applicators positioned in the GI tract, with integrated MR-guidance for target identification, device positioning/alignment, and MRTI treatment monitoring. Future applicator development will involve incorporating endoscopic steering capabilities to enhance control over endoluminal placement, fixation, and coupling to the gastric wall to improve positional accuracy and luminal sparing.

## A103 A randomized, sham-controlled trial of transcranial MR guided Focused Ultrasound thalamotomy trial for the treatment of tremor-dominant, idiopathic Parkinson’s disease

### Aaron Bond^1^, Robert Dallapiazza^1^, Diane Huss^1^, Amy Warren^1^, Scott Sperling^1^, Ryder Gwinn^2^, Binit Shah^1^, W. Jeff Elias^1^

#### ^1^University of Virginia, Charlottesville, Virginia, USA; ^2^Swedish Neuroscience Institute, Seattle, Washington, USA

**Objectives**

Traditional stereotactic RF thalamotomy has been used with success in medication refractory tremor-dominant Parkinson’s disease. Recently, transcranial MR guided Focused Ultrasound (MRgFUS) has been used to successfully perform thalamotomy for essential tremor. We designed a double blinded, randomized controlled trial to investigate the effectiveness of MRgFUS thalamotomy in tremor-dominant PD.

**Methods**

Patients with medication refractory, tremor-dominant Parkinson’s disease were enrolled in the two center study and randomized 1:2 to receive either a sham procedure or treatment. After the 3 month blinded phase, the sham group was offered treatment. Outcome was measured with blinded CRST and UPDRS ratings. The primary outcome compared improvement in hand tremor between the treatment and sham procedure at 3 months. Secondary outcomes were measured with UPDRS and hand tremor at 12 months. Safety was assessed with MRI, adverse events, and comprehensive Neurocognitive assessment.

**Results**

Twenty-seven patients were enrolled and six were randomized to a sham procedure. For the primary outcome assessment, there was a mean 50 % improvement in hand tremor from MRgFUS thalamotomy at 3 months compared to a 22 % improvement from the sham procedures (p = 0.088). The 1 yr tremor scores for all 19 patients treated with 1 year follow up data (blinded and unblinded) showed a reduction in tremor scores of 40.6 % (p = 0.0154) and a mean reduction in medicated UPDRS motor scores of 3.7 (32 %, p = 0.0326). Sham patients had a notable placebo effect with a mean 21.5 % improvement in tremor scores at 3 months. Twenty seven patients completed the primary analysis, 19 patients completed the 12 months assessment, 3 patients opted for DBS, 3 were lost to follow up, 1 patient opted for no treatment, and 1 is pending 12 m evaluation.

**Conclusions**

Transcranial MRgFUS demonstrates a trend towards improvement in hand tremor, and a clinically significant reduction in mean UPDRS. A significant placebo response was noted in the randomized trial.

## A104 MR Image-guided delivery of non-viral miRNA-34a gene vectors via Focused Ultrasound inhibits tumor growth in a mouse glioma model

### Colleen Curley^1^, Ying Zhang^1^, Karina Negron^2^, Wilson Miller^1^, Alexander Klibanov^1^, Roger Abounader^1^, Jung Soo Suk^3^, Justin Hanes^3^, Richard Price^1^

#### ^1^University of Virginia, Charlottesville, Virginia, USA; ^2^Johns Hopkins University, Baltimore, Maryland, USA; ^3^Center for Nanomedicine/Wilmer Eye Institute, Johns Hopkins University, Baltimore, Maryland, USA

**Objectives**

Glioblastoma (GBM) is remarkably difficult to treat with systemically administered therapeutics because the blood brain/blood tumor barrier (BBB/BTB) blocks their diffusion from the bloodstream, while the bioadhesive and nanoporous ECM hinders their tissue dispersion. In addition, GBM is characterized by genetic complexity, molecular adaptability, and multiple deregulated pathways, all of which markedly limit conventional therapies. Here, we address these obstacles by combining Focused Ultrasound (FUS)-mediated disruption of the BBB/BTB with brain-penetrating non-viral gene vectors (DNA-BPN) bearing the plasmid for miRNA-34a, a miRNA that is downregulated in human GBM and predicted to regulate multiple tumor suppressive pathways.

**Methods**

U87 malignant glioma cells were injected into the brains of athymic nude mice. Five days later, anesthetized mice were coupled to an MRI compatible 1.14 MHz FUS system. T1 contrast MR imaging was used to visualize and target tumors. miR-34a plasmid-bearing DNA-BPN that had been densely PEGylated to permit brain penetration were i.v. co-injected with microbubbles. FUS was applied to tumors with a 0.5 % duty cycle for 2 minutes at either 0.4 or 0.5 MPa peak negative pressure (PNP). Immediately after sonication, additional contrast MR images confirmed BBB/BTB opening. Untreated mice and mice that were i.v. injected with miRNA-34a BPNs and MBs without FUS application served as controls. At 7 days and 15 days after treatment, corresponding to 12 and 20 days after tumor implantation, tumor volumes were determined from 7 T MR images.

**Results**

**Fig. 110 (abstract A104).**
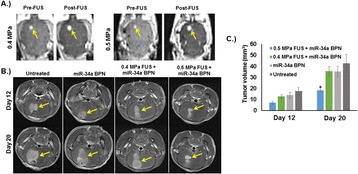
**a** Pre- and Post-FUS contrast MR images of U87 tumors (yellow arrows). **b** 7 T contrast MR images of U87 tumors (yellow arrows) at 12 and 20 days after tumor implantation. **c** U87 tumor volumes. P < 0.05 vs. all other groups at Day 20

Figure 110a shows representative pre- and post-FUS T1-weighted contrast MR images when using PNPs of 0.4 and 0.5 MPa. Enhanced signal in the post-FUS images indicates increased leakage of gadolinium contrast agent across the vasculature due to FUS-mediated BBB/BTB disruption. Note that BBB/BTB disruption appears to be further enhanced at 0.5 MPa when compared to 0.4 MPa. Figure 110b shows representative 7 T images of U87 tumors for each group taken at 12 and 20 days post tumor implantation. These images were used to quantify post-treatment tumor volume (Fig. 110c). At Day 20, mice treated with 0.5 MPa FUS + MB + miRNA-34a BPN (n = 3) showed a marked and statistically significant (p < 0.05) decrease in tumor volume when compared to the untreated (n = 5), miRNA-34a BPN + MB (n = 4), and 0.4 MPa FUS + MB + miRNA-34a BPN (n = 4) groups. For all groups, T2* MRI (not shown) confirmed that there was no leakage of blood products across the BBB/BTB.

**Conclusions**

We show that a single MR image-guided treatment, entailing the FUS (1.14 MHz; 0.5 MPa) mediated delivery of brain-penetrating non-viral miR-34a gene carriers across the BBB/BTB, can significantly inhibit glioma growth in mice. To the best of our knowledge, this represents the first time that therapeutic miRNA plasmid has been complexed into a non-viral gene nanocarrier and delivered using Focused Ultrasound for treatment of gliomas. Ongoing studies are centered on optimizing miRNA-34a expression levels, verifying changes in miRNA-34a oncogenic target protein expression, and assessing tumor cell apoptosis and proliferation.

## A105 Neurorestoration of the nigrostriatal pathway through multiple treatments with FUS-facilitated brain drug delivery

### Maria Eleni (Marilena) Karakatsani, Gesthimani Samiotaki, Shutao Wang, Tara Kugelman, Camilo Acosta, Elisa Konofagou

#### Columbia University, New York, New York, USA

**Objectives**

Current Central Nervous System (CNS) drug delivery techniques are confined to either targeted but invasive or to non-targeted and non-invasive. Focused Ultrasound (FUS) coupled with the systemic administration of microbubbles has been proven to open the Blood-Brain Barrier (BBB) locally, transiently and non-invasively, thus facilitating the diffusion of neurotrophic factors. IV injection of Neurturin, a member of the glial derived neurotrophic factors (GDNF) family has been demonstrated to activate the downstream signaling pathway following BBB opening. The promising previous findings set the milestone for investigating the effect of Neurturin on the depleted dopaminergic neurons *in vivo*. The aim of the current study was thus to investigate the neurorestorative effects of single and triple delivery sessions of the neurotrophic factor Neurturin in a Parkinsonian mouse model.

**Methods**

For this study a single control group was employed and three groups per treatment cascade; single and triple. Wild type mice (12 months old) were infused with sub-acute dosages of MPTP causing apoptotic degeneration in the nigrostriatal pathway. After stabilization of the MPTP lesions and the decontamination period, the treatment groups were sonicated on the left hemisphere targeting twice the Caudate Putamen region (CPu) and once the Substantia Nigra region (SN) while one received an IV injection of 0.5 mg Neurturin. The survival period after the last treatment was equal to 28 days allowing the neurotrophic factor to develop its restorative effects followed by coronal sectioning for tissue processing. The brain slices of both the SN and the CPu were stained for tyrosine hydroxylase positive cells (TH+) with a custom protocol. Brain sections were imaged to count the TH+ nerve cell bodies on the SN while the dendritic and terminal areas were quantified by a custom MATLAB algorithm by computing the percentage of the relative difference (RD) between the two hemispheres as for each mouse the contralateral side was compared to the ipsilateral side to eliminate across-mice variation in the number of nuclei and projections.

**Results**

Comparison between the neuronal cells on the contralateral side and the ipsilateral side revealed an increase in the cell number on the treated side only for the group that received Neurturin with FUS. The RD was found to be significantly higher in the SN region for the groups that received Neurturin, both in the cases of single and multiple treatments. An increase in RD for the group that received multiple treatments with Neurturin compared to the single administration group was not observed. On the other hand, significant increase in the RD was only found in the case of the triple-treatment group in the CPu region.

**Conclusions**

The increased RD at the SN region revealed the immense potential of the current treatment and the necessity of expanding the study into multiple administrations of Neurturin. The comparable findings in dendritic density at the SN region between the two treatment regimens pointed towards the direction of examining the treatment effect at the CPu region. The significantly increased CPu terminal density could be explained as the restoration of the neuronal processes through collateral sprouting. Finally, despite the increase in neuronal cells on the ipsilateral side, no statistical significance was observed. This finding is in accordance with our knowledge of Neurturin restoring impaired neurons and not regenerating them. The presented findings are essential considering the therapeutic effect of multiple treatments with FUS enhanced drug delivery in patients.

**Fig. 111 (abstract A105).**
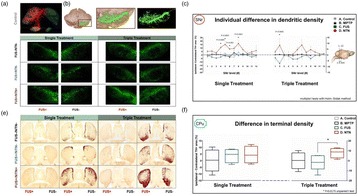
Substantia Nigra: **a** Atlas 3D representation of the nigrostriatal pathway and the involved structures, Substantia Nigra (SN) and Caudoputamen (CPu). **b** Coronal section at a cross-section of the SN in increasing magnification. **c** Fluorescent im

## A106 Long-term effects of Blood-Brain Barrier opening with pulsed Focused Ultrasound and microbubbles

### Zsofia Kovacs, Tsang-Wei Tu, Georgios Papadakis, Dima Hammoud, Joseph Frank

#### National Institutes of Health Clinical Center, Bethesda, Maryland, USA

**Objectives**

The objective of this study is to use advanced Magnetic Resonance Imaging (MRI) techniques to characterize the long-term effects of pulsed Focused Ultrasound (pFUS) and microbubbles (MB) in the rat brain. We evaluated the effects of single and repeated Blood-Brain Barrier (BBB) disruptions (D) on morphology in the rat striatum and hippocampusas monitored by MRI and histology over 12 weeks.

**Methods**

Female Sprague Dawley rats (n = 6/group) received either pFUS + MB once (Group A) or six weekly exposures (Group B) to the striatum and the contralateral hippocampus along with 100 μl of intravenous MB (OptisonTM, GE Healthcare). Prior to the first sonication rats received 3 daily doses of 300 mg/kg 5-Bromo-2′-deoxy-uridine (BrdU, Sigma Aldrich) intraperitoneally to label proliferating (neurogenesis) cells*in vivo*. 0.3-0.5 MPa acoustic pressures were applied in 10 ms burst length and 1 % duty cycle (9 focal points, 120 sec/9 focal points – striatum, 120 sec/4 focal points – hippocampus) using a single-element spherical FUS transducer (center frequency 589.636 kHz; FUS Instruments). T2w, T2*w and Gadofosveset (Gd)-enhanced T1w images were obtained by 3.0 T MRI (Philips) and T2w, T2*w, diffusion tensor imaging (DTI) was performed by 9.4 T MRI (Bruker). Animals were euthanized 6 or 12 weeks after the first pFUS treatment. Histological evaluation of brain and tracking of BrdU tagged cells was performed and compared to untreated contralateral brain.

**Results**

**Fig. 112 (abstract A106).**
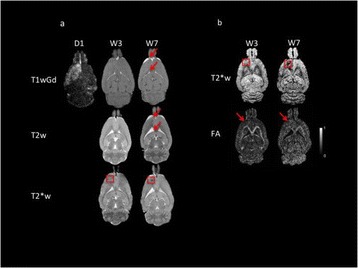
**a** T1wGd, T2w, T2*w MRI (3.0 T), and **b** T2*w and DTI MRI (9.4 T) of a representative brain after 6 weekly sonications

In groups A and B, contrast enhancement on T1w images was detected post sonication in the striatum and the hippocampus. Gd-extravasation, T2 and T2* abnormalities were not seen in the brain 1 day post pFUS + MB at 9.4 T MRI. Approximately 50 % of Group A and 100 % of Group B rats had hypointense voxels appeared on T2*w 3 T MRI 2-3 weeks post pFUS + MB (Fig. 112a) consistent with microhemorrhages or influx of metallophagocytic cells from the spleen within the parenchyma. Fractional anisotropic (FA) changes in white matter fiber structure- and gray matter-abnormalities on DTI MRI were detected in regions with the T2* abnormalities suggestive of increased astrogliosis and transient axonal damage along with increased cell density. Group B also demonstrated ventriculomegaly and meningeal abnormalities (Fig. 112a, b). Histologically, increase in numbers of Nissl positive cells along with activated microglia and BrdU labeled cells were detected in both cortex and hippocampus in greater amounts in Group B compared to Group A rats, consistent with multiple episodes of induced sterile inflammatory response (SIR) within the parenchyma.

**Conclusions**

We have observed a complex graded molecular and cellular SIR with increase in the brain up to 24 hrs after pFUS + MB. However, little has been reported on using advanced imaging techniques at high magnetic field strengths on the long-term effects of single and repeated pFUS + MB in the brain. Based on changes in FA, DTI MRI demonstrated low degree of structural injury at the location of both single and multiple sonication exposures. Multiple pFUS + MB exposures resulted in increased ventriculomegaly and numbers of activated microglia, Nissl positive cells and BrdU in both striatum and hippocampus. Increased numbers of BrdU+ cells would be consistent with the stimulation of neurogenesis indicative of increased damage secondary to repeated SIR episodes in the parenchyma.

## A107 Activatable nanodelivery combined with CpG-ODN and anti-PD-1 achieves a complete response in directly-treated and contralateral tumors in a murine breast cancer model

### Matthew Silvestrini

#### University of California Davis, Davis, California, USA

**Objectives**

Activatable nanotherapeutics are attractive since the toxicity of chemotherapeutics can be constrained to a small region; combining such a strategy with immunotherapy is the goal of this study. We have previously shown that administration of CpG-ODN as an adjuvant, together with local release of doxorubicin from temperature sensitive liposomes (TSL) resulted in regression of directly-treated tumors, suppressed growth of contralateral tumors and reduced chemotherapeutic-mediated toxicity in a murine breast cancer model.

**Methods**

The following is the protocol explored for the addition of anti-PD1: immune intact FVB/n mice with bilateral invasive neu deletion syngeneic transplanted tumors were treated with a combination of anti-PD1 (aPD-1, 200 μg, i.p.) and intratumoral administration of CpG-ODN (100 μg, i.t.) on days 0, 7, 14 and 0, 3, 7, 10, 17 and 24, respectively. Doxorubicin TSL were prepared from DPPC:MPPC:DSPE-PEG2k, 86:10:4 in the presence of copper (II) gluconate and triethanolamine at 0.2 mg-drug/mg-lipid and administrated i.v. at 6 mg doxorubicin/kg body weight on days 10, 17, 24. The formation of a complex between doxorubicin and copper was created to enhance the circulation and stability of TSL and to reduce systemic toxicity. To trigger drug release, hyperthermia was induced in the primary tumor with ultrasound (peak ultrasound pressure of 1.1 MPa at a frequency of 1.5 MHz) at 42 °C for 5 min prior to and 20 min post drug injection with a variable duty cycle. Immediately afterwards, 100 μg of CpG-ODN 1826 was administered intratumorally to the insonified tumor.

**Results**

Upon treatment with this combination of locally-released doxorubicin, local administration of CpG-ODN and systemic aPD-1, 100 % of treated and contralateral tumors regressed by at least 80 %; further, all of the directly-treated tumors and 50 % of the contralateral tumors were eliminated without recurrence. Thus, a 50 % complete response rate was achieved, with tumor regression observed immediately after the incorporation of the doxorubicin treatment. By contrast, administration of CpG-ODN and systemic aPD-1 alone resulted in regression of 66 % of treated and contralateral tumors.

**Conclusions**

We demonstrate for the first time that blocking of the programmed death-1 (PD-1) pathway in conjunction with immunogenic cell death induced by CpG-ODN and activatable nanodelivery of doxorubicin can generate curative responses in both primary and contralateral tumors. Increases in cytotoxic CD8+ T lymphocytes and a reduction in regulatory T cells and myeloid-derived suppressor cells were observed in both directly treated and contralateral tumors. This combinatorial approach was curative for directly-treated tumors and overall survival was significantly extended, however, the contralateral tumor returned in all treated mice.

## A108 Towards FUS lung cancer ablation: Aspects of MR guidance in flooded lung

### Frank Wolfram^1^, Daniel Güllmar^2^, Juergen Reichenbach^2^, Denis Hofmann^3^, Joachim Böttcher^3^, Harald Schubert^4^, Thomas G. Lesser^3^

#### ^1^SRH Wald-Klinikum Gera / Clinic of Thoracic Surgery, Gera, Germany; ^2^Institute of Diagnostic and Interventional Radiology, University Hospital - Friedrich Schiller University, Jena, Germany; ^3^SRH Wald-Klinikum Gera, Gera, Germany; ^4^Institute of Animal Experimentation, Friedrich Schiller University, Jena, Germany

**Objectives**

One-Lung flooding (OLF) enables HIFU application under sonographic guidance in lung. Additionally MRgFUS is superior for non-invasive ablation under thermal dose monitoring. FUS treatment of lung tumours requires complete gas- liquid exchange in the treated lung section, which will likely affect also imaging modalities. MRI is seldom used for diagnosis and staging of thoracic tumours because of low proton density with short T2* and field inhomogeneities in ventilated lung. So far no data is accessible for MR imaging of lung in flooded condition and therefore its use for FUS guidance on lung tumours. This study was aimed to investigate *in vivo* OLF in MR environment and to evaluate the capabilities of MR imaging on lung tumours (NSCLC).

**Methods**

OLF was performed on six pigs (female 35-60 kg) in 3 T MR (Prisma, Siemens AG, Germany). After narcosis, mechanical ventilation was performed with an ICU respirator (Servo 900, Siemens AG, Germany) through a 39 Ch double-lumen tube (Mallinckrodt, Ireland). After ventilation with FIO2 = 1.0 for 30 min the left lung wing was flooded with isotonic saline (0,9 %@ 35 °C). Ventilation of the right lung was maintained for 60-90 min, followed by re-ventilation for 30 min. MR imaging was performed with spine and body array coils in lateral position using T2 HASTE, and T1 weighted GRE sequences. As a cancerous *ex vivo* model, MR imaging was performed on ten human lung lobes containing lung cancer (NSCLC). Lobes where flooded immediately after resection with saline (35 °C) and imaged using GRE and T2 HASTE sequences in 1,5 T MR (Achieva, Philips, NL).

**Results**

**Fig. 113 (abstract A108).**
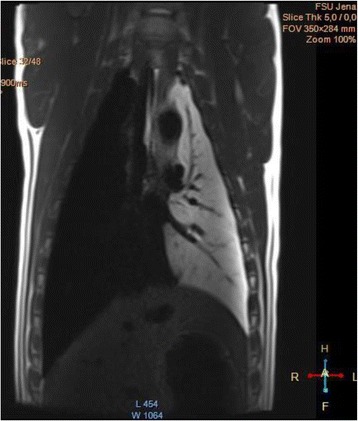
In-vivo T2w (84 ms/900 ms) image of porcine lung, right ventilated, left flooded lung

**Fig. 114 (abstract A108).**
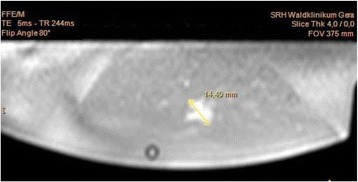
Ex-vivo T1w GRE (5 ms/244 ms) image of flooded human lung lobe with hyperintense NSCLC-Adenocarcinoma

OLF was successfully performed in MR environment on all animals (6/6). Flooded lung appears hyperintense in T2 weighted images (Fig. 113) and hypointense in T1w. Bronchial wall and vascular structures in flooded lung clearly appear as hypointense structures in T2 weighted image at high level of detail. GRE phase images show a homogenous phase in bronchial and alveolar tissue of the flooded lung wing. NSCLC tissue appear in flooded lung as strongly hyperintense lesions in T1 (Fig. 114) and hypointense in T2 weighted images with clear demarcation from lung parenchyma.

**Conclusions**

This study shows that *in vivo* OLF is operable in MR environment, all animals survived the procedure. The absence of susceptibility changes in GRE phase images suggests a complete gas to saline exchange in the flooded lung wing. For FUS guidance a demarcation of tumor to parenchymal structures is essential. In T1w images the lung tumour appears bright hyperintense to surrounding lung in all tumours. Further in T2w images a good demarcation between bronchus and parenchyma can be used for anatomic orientation. It can be concluded that MR imaging based on general fast GRE and TSE sequences is sufficient for FUS guidance on lung cancer during OLF. Further research investigating nodule size detection limits and the use of PRFS thermometry in flooded lung is required.

## A109 Towards patient-specific acoustic scattering models for use in transcranial HIFU treatments

### Scott Almquist, Dennis Parker, Douglas Christensen

#### University of Utah, Salt Lake City, Utah, USA

**Objectives**

In transcranial HIFU treatments, scattering that occurs in the porous skull can contribute to a significant portion of the ultrasonic attenuation. In many acoustic models, scattering is neglected or considered homogenous across the skull. However, significant variations in skull anatomy may require patent-specific models of scattering for treatment planning. These patient-specific models are based on clinical CT scans that do not have high enough resolution to resolve many of the scattering sites. In this work, we relate the scattering properties of the skull, as simulated using a high-resolution microCT scan, to quantitative Hounsfield Units (HU) as measured by a clinical CT.

**Methods**

A high-resolution (23.6 micrometer isotropic) microCT scan was obtained of an *ex vivo* human skull section in a holder and thresholded into cortical bone and pores. The same skull was imaged with a clinical-resolution (0.23 x 0.23 x 0.4 mm) CT scan. The images were registered using hand-selected correspondence points. A database of pores was created from the microCT scan via connected components analysis and associated with the corresponding HU values in the clinical CT scan based on the center of mass. Additionally, the ratio of cortical bone *versus* porous material in the microCT was calculated for each associated HU range in the clinical CT. Models for various HU ranges were constructed by randomly selecting hole shape and sizes associated with the HU range and placing them in uniform bone until the ratio of cortical bone to porous material was the same as measured for the HU range. Simulations were then performed using the Hybrid Angular Spectrum^1^ technique assuming no absorption. The ratio of power at the focus compared to the input power at the face of the model is representative of scattering and reflections.

**Results**

**Fig. 115 (abstract A109).**
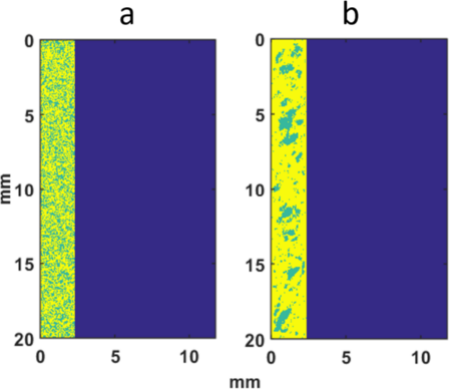
Generated scattering model for Hounsfield Units of **a**) 301-400 and **b**) 801-900. Bone is represented as yellow, while porous material is represented as green and simulated as marrow. Blue is water. The transducer is to the left and focused in the water

**Fig. 116 (abstract A109).**
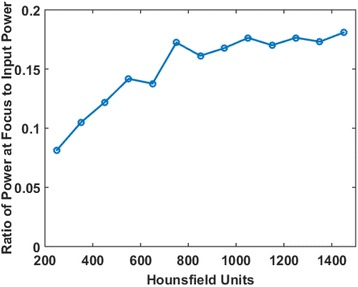
The ratio of the power at the focal plane to the power input for various models representing different Hounsfield units

Figure 115 shows example models for 301-400 and 801-900 HU ranges respectively. For simulations, the transducer is to the left of the model with the focus in the water to the right. These representative models clearly show the differences in shape distribution of the pores as well as overall density of the different HU ranges. Figure 116 shows the ratio of power at the focal plane compared to the power input to the simulation as a function of HU range. The power lost is due to both scattering and reflections.

**Conclusions**

The work presented here is the first step towards patient-specific models of acoustic scattering. The models created represent the differences in scattering and reflections for various HU ranges. Future work will be focused on applying these results in simulations at a clinical resolution to validate the method.

**Reference**

1. U. Vyas and D. Christensen, “Ultrasound beam simulations in inhomogeneous tissue geometries using the hybrid angular spectrum method,” IEEE Trans. Ultrason. Ferroelectr. Freq. Control, 2012 59(6):1093–1100.

## A110 Study of aberrations at the focus of an ultrasonic beam due to the propagation across different areas of the skull

### Francisco Camarena^1^, Sergio Jiménez-Gambín^1^, Noé Jiménez^2^, Elisa Konofagou^3^

#### ^1^Universitat Politècnica de València, Gandia, Spain; ^2^LUNAM Universite. Université du Maine, Le Mans, Spain; ^3^Columbia University, New York, New York, USA

**Objectives**

Over 95 % of useful therapeutically molecules cannot penetrate to the brain parenchyma in central nervous system due to the Blood-Brain Barrier. In recent years it has been developed a promising ultrasound assisted technique for noninvasive, targeted and safe BBB disruption for drug delivery. This technique employs a transcranial focused ultrasound beam focalized on targeted BBB areas to disrupt. However, there are many limitations of the technique: the skull shows very high acoustic absorption, the acoustic impedance of the skull bone is highly different than the inner brain and outer media and the shape of the skull produces aberrations in the acoustic field generated by focused acoustic beams that cause unexpected targeted focal areas. The aim of this work is to study the transcranial targeting of whatever part of the brain taking into account normal skull surface incidence, in order to find the best incidence zone which generates less beam aberrations.

**Methods**

A 500 KHz focused transducer (aperture = 100 mm; radius = 140 mm) was simulated with K-Wave Matlab toolbox, which is based on the k-space pseudospectral method. A 3-D fluid media was considered with CT-based acoustic maps of the skull. Geometrical algorithms were developed to select the position of the transducer normal to the skull from occipital to frontal areas and from temporal to parietal; and several physical criteria have been applied to quantify the aberration level by comparing the focal area of the beam when it propagates with and without skull.

**Results**

**Fig. 117 (abstract A110).**
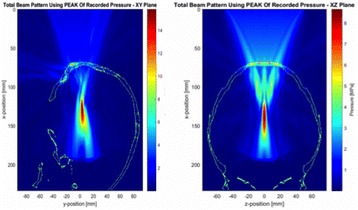
Occipital incidence of the ultrasonic beam. The focus suffers a higher deviation in the XY plane than in the XZ one

Refraction has proven to be one of the most important effects in the propagation through the skull. Figure 117 shows the effect of the skull shape in the focus of the single element transducer simulated, both in a XY plane and in the YZ plane. We provide an evaluation of the aberration of the focus of the beam in each plane following a plath from the occipital to the frontal areas. Aberration in each plane are different, and more important in the occipital area, were irregularities of the skull shape affects the propagation due to the refraction phenomenon. Here, also, the existence of muscle adhered to the bone increases the refraction of the beam.

**Conclusions**

The shift in the location of the focus respect to the propagation of the free ultrasonic beam changes when the beam targets different areas of the skull. It can be also affected by the presence of muscles attached to the bone, and of course, when not normal incidence is considered. The best target areas to minimize aberration in the beam have been selected and characterized.

## A111 Bilateral thermal capsulotomy with Focused Ultrasound for treatment-refractory obsessive-compulsive disorder: 1-year follow-up

### Jin Woo Chang

#### YUMC Severance Hospital, Seoul, Republic of Korea

**Objectives**

We investigated the efficacy and safety of bilateral thermal lesioning of the anterior limb of internal capsule (ALIC) using magnetic resonance-guided focused ultrasound (MRgFUS) in patients with treatment-refractory obsessive-compulsive disorder (OCD).

**Methods**

Eleven patients with treatment-refractory OCD were included. Clinical outcomes were evaluated using the Yale-Brown Obsessive-Compulsive Scale (Y-BOCS), Hamilton Rating Scale for Depression (HAM-D), and Hamilton Rating Scale for Anxiety (HAM-A) at 1 week and 1, 3, 6, and 12 months following MRgFUS. Neuropsychological functioning, Global Assessment of Functioning (GAF), and adverse events were also assessed. This trial is registered with ClinicalTrials.gov number, NCT01986296.

**Results**

After bilateral thermal lesioning of the ALIC using MRgFUS, Y-BOCS scores significantly decreased across the 12-month follow-up period (median Y-BOCS score, 34 vs. 22, p < 0.001). HAM-D and HAM-A scores also significantly decreased (median HAM-D score, 19 vs. 6, p < 0.001; median HAM-A score, 20 vs. 6, p < 0.001). GAF score also significantly improved (median GAF score, 35 vs. 48, p < 0.001). Wechsler Adult Intelligence Scale-Korean version and Memory Quotient scores significantly improved after MRgFUS, and Controlled Oral Word Association Test, Stroop Test, and Digit Span Test scores were unchanged. Adverse events were mild and transient, including headache, nausea, and anxiety during sonication.

**Conclusions**

The results of this pilot study suggest that bilateral thermal lesioning of the ALIC using MRgFUS may improve obsessive-compulsive, depressive, and anxiety symptoms in treatment-refractory OCD patients without serious adverse events

## A112 Development of an optically-guided system for transcranial Focused Ultrasound

### Vandiver Chaplin^1^, Rebekah Griesenauer^2^, Michael Miga^2^, Charles Caskey^2^

#### ^1^Vanderbilt University Institute of Imaging Science, Nashville, Tennessee, USA; ^2^Vanderbilt University, Nashville, Tennessee, USA

**Objectives**

Focused ultrasound is a promising method for non-invasive neural stimulation therapies in the brain. Early animal and human studies have shown that neurological conditions such as essential tremors can be effectively treated with ultrasound ablation therapy, while sub-thermal neuromodulation has therapeutic effects that could benefit a variety of neurological diseases, including manic depressive disorder, Alzheimer’s and epilepsy. Ultrasound treatments typically occur using image-guidance within a magnetic resonance imaging (MR) system, but working outside the MR environment is often desirable. Conventional image-guided surgery can be used to enable ultrasound neurosurgery outside the magnet by using preoperative MR data registered to the physical patient, fixing tracked markers to the ultrasound probe and registering the tracked space to pre-acquired images. The accuracy of this system is determined by calibration between the tracked markers and acoustic focus. Here, we present a pulse-echo based method of calibration and characterize its accuracy using MR thermometry.

**Methods**

**Fig. 118 (abstract A112).**
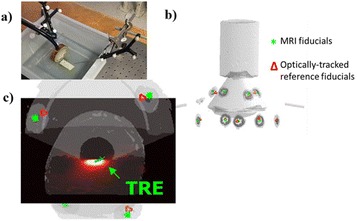
**a** Optically track calibration target (right) and FUS transducer (left) operated in pulse-echo mode. **b** Iso-surface of the phantom + transducer scan showing registration of multi-modal fiducials. **c** Results comparing thermal scan maximum and pulse-echo maximum, with the displacement between representing total registration error

We localized the tip of an optically-tracked stylus relative to the HIFU transducer (Sonic Concepts H-115, center frequency 250 kHz) by using the transducer in pulse-echo mode, while simultaneously tracking a rigid target attached to the transducer (Fig. 118a). A standard optical tracking system (NDI Polaris, Waterloo, ON) was used to record the transducer and probe locations during pulse-echo acquisition. The stylus was mounted to a 3D translation stage to function as an echo target, and near-infrared reflectors with well-characterized geometry were affixed to the transducer with custom mounting hardware to make a rigid body for registration. We acquired locations of the rigid bodies as the probe was moved in 3-directions to maximize the echo signal, providing an estimate of the ultrasound focus position in optical coordinates. Next, multi-modal fiducials (visible via MRI) were attached to the transducer, and their positions measured with the Polaris system. Finally, the transducer and a sonication phantom were placed in the MR scanner. The thermal focus position was measured with MR thermometry, and the fiducial locations in MR coordinates were used to register the focus position to optical coordinates. The difference of MRI-derived thermal focus and the pulse-echo focus was the target registration error (TRE).

**Results**

MRI (green stars) and optically tracked fiducials (red triangles) were registered to coincident locations (Fig. 118b), yielding a fiducial registration error of 2.8 mm. After registration, the mean TRE between the pulse-echo focus and thermal focus across three phantoms was 5.7 +/- 1.5 mm (Fig. 118c). This error is comparable to published methods, but work is ongoing to achieve better results by optimizing placement of optical tracking targets on the HIFU transducer. The TRE of this method in the context of trans-cranial sonication is currently being evaluated.

**Conclusions**

Optical tracking allows prediction of FUS focal zone location. Future experiments will assess the target registration error by performing optically-guided transcranial ablation of a brain-equivalent phantom and assessing the lesion location in a post-procedural MRI.

## A113 The feasibility of MR-guided FUS BBB disruption through an intact human skull with a clinical body transducer: Pre-clinical results in a rat model

### Nicholas Ellens^1^, Raag Airan^2^, Alfredo Quinones-Hinojosa^1^, Keyvan Farahani^3^, Ari Partanen^4^

#### ^1^Johns Hopkins University, Baltimore, Maryland, USA; ^2^Stanford University, Stanford, California, USA; ^3^National Cancer Institute, Bethesda, Maryland, USA; ^4^Philips, Bethesda, Maryland, USA

**Objectives**

MR-guided Focused Ultrasound (MRgFUS) has been used for many non-invasive mechanical and thermal therapies throughout the body. One of the most promising applications in the brain is the use of MRgFUS for safe, local, and transient Blood-Brain Barrier (BBB) disruption.

Current transcranial MRgFUS therapy is performed using a hemispherical, 1024-element transducer. For thermal therapy, this design is warranted as a large aperture is required to obtain enough gain for focal heating without overheating the skull. For low duty cycle and low pressure applications like BBB disruption, it may be possible to use smaller-aperture transducer arrays like those found in clinical body MRgFUS systems. The goal of this study is to characterize a clinical body MRgFUS system in regards to ultrasound transmission through a human cranium and in an application to disrupt the BBB *in vivo* in rats.

**Methods**

**Fig. 119 (abstract A113).**
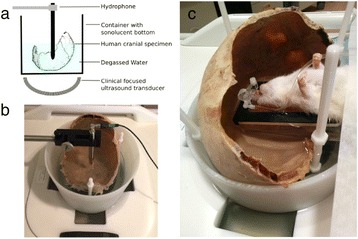
Schematic (**a**) and picture (**b**) of the calibration setup with the hydrophone positioned over an ex vivo cranial specimen. **c** A picture showing a rat positioned with its brain near to a known pressure calibration location within the skull

A clean, degassed human cranium was mounted in a bath of degassed water over a 14 cm, 256-element clinical body transducer (Sonalleve V2, Philips). Short, 40 cycle ultrasound bursts at 1 MHz were transmitted through the skull, and acoustic pressures were measured using a hydrophone mounted to a positioning stage (Fig. 119). Three different skull orientations were evaluated at 3-4 different sonication depths, along with evaluating the sensitivity of ultrasound transmission to small transducer translations.

Six anesthetized rats were shaved and positioned supine on an acoustic window with their brains at the location of prior pressure calibration. MR imaging was used for targeting. Microbubbles (0.02 mL/kg, Definity) were injected concurrently with sonications (0.4-0.6 MPa *in situ* based on hydrophone calibration, 10 ms bursts at 1 Hz pulse repetition frequency for 120 s). Gadolinium was administered (0.2 mL/kg, Magnevist) and follow-up T1-weighted imaging was performed to evaluate BBB disruption. Three brains were further examined with histology to look for damage.

**Results**

Hydrophone measurements demonstrated a great deal of variation in ultrasound transmission with change in skull orientation, though the change was gradual. Attenuation ranged from ¬ 3.7 dB to 9.3 dB (11 % to 43 % transmission). For a single skull orientation, the average variation in transmission was 3 % for a 5 mm transducer translation in-plane.

**Fig. 120 (abstract A113).**
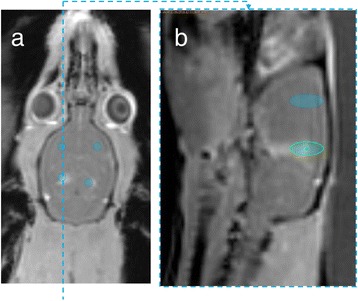
Axial (**a**) and sagittal (**b**) contrast-enhanced T1-weighted MRI scans indicating focal BBB disruption with intended targets overlaid in blue. From the top-left and clockwise in (**a**), the four targets had in situ pressures of 0.4, 0.5, 0.45, and 0.55 MPa

**Fig. 121 (abstract A113).**
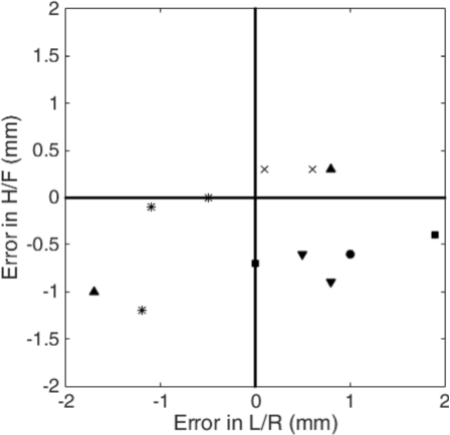
In-plane error of BBB disruption location. Different symbols represents a different animals

Contrast enhancement in the brain indicated focal BBB disruption (Fig. 120). The error in the disruption location perpendicular to the ultrasound beam was 1.0 +/- 0.5 mm, (Fig. 121). The error in the dorsal/ventral direction was 2 +/- 2 mm. The Lilliefors normality test did not reject the null hypothesis that these samples were drawn from a normal distribution (p < 0.05). In each animal, enhancement increased with *in situ* sonication pressure, though there was a large variation.

**Conclusions**

Transcranial MRgFUS is feasible with a smaller-aperture transducer for BBB disruption. The accuracy of sonication was 1 mm in plane and 2 mm out of plane without any corrections for the skull. For clinical feasibility and safety, however, the attenuation of the skull must be taken into account so as to apply appropriate pressures to disrupt the BBB without causing damage.

## A114 Towards real-time *in vivo* MR thermometry

### Xue Feng^1^, Samuel Fielden^1^, Li Zhao^1^, Wilson Miller^1^, Max Wintermark^1^, Kim Butts Pauly^2^, Craig Meyer^1^

#### ^1^University of Virginia, Charlottesville, Virginia, USA; ^2^Stanford University, Stanford, California, USA

**Objectives**

Real time MR thermometry, usually based on the proton-resonance frequency shift, is a key aspect of MR-guided ultrasound procedures. The desire to monitor the entire insonicated volume has led the field towards the development of rapid, 3D methods; however, acquiring fully sampled 3D volumetric data to monitor heating is time consuming, and so fast methods must be developed in order to meet the spatial and temporal requirements for adequate monitoring of thermal therapy. Spiral k-space trajectories have higher data acquisition efficiency than the traditional Cartesian scanning so that they are an attractive way to improve temporal resolution while maintaining spatial resolution in MR thermometry. Furthermore, temporal redundancy can be exploited with real-time reconstruction models such as the Kalman filter to further accelerate the scan without sacrificing the real-time tracking capabilities. The purpose of this study was to implement the 3D spiral thermometry sequence on a real-time platform to demonstrate its effectiveness.

**Methods**

A non-accelerated 3D retraced spiral-in/out (RIO) thermometry sequence was implemented on the RTHawk platform (HeartVista, Inc.) to enable real-time sequence control and monitoring of a FUS insonication. RTHawk interfaced with a GE Discovery MR750T 3 T scanner at the UVA Focused Ultrasound Center, where an Insightec ExAblate 650 focused ultrasound transducer was used to induce focal heating. Three Yorkshire pigs were used for initial sequence evaluation. Non-ablative power settings were used to compare the temperature measured by the 3D sequence to those measured by the standard 2D GRE thermometry sequence provided by Insightec. Maximum temperature, measured by the single hottest pixel, and mean temperature, measured as the average of the 9 pixels centered at the hottest pixel in a single plane, were recorded. After FUS, the animals were moved into a head coil array for high quality post-ablation assessment, which induced T2-, Diffusion, and T2*-weighted imaging using standard sequences.

To explore the acceleration technique, a Kalman filter model was developed given as:

$$ \mathrm{x}\left(\mathrm{k}\right) = \mathrm{x}\left(\mathrm{k}\hbox{-} 1\right) + \mathrm{w}\left(\mathrm{k}\hbox{-} 1\right) $$

$$ \mathrm{z}\left(\mathrm{k}\right) = \mathrm{U}\left(\mathrm{k}\right)\mathrm{F}\mathrm{x}\left(\mathrm{k}\right) + \mathrm{v}\left(\mathrm{k}\right) $$

in which x(k) is the target image, z(k) is the measurement from undersampling pattern U(k) and Fourier transform matrix F. The model can give optimal estimation of x(k) based on undersampled measurement and previous images.

**Results**

**Fig. 122 (abstract A114).**
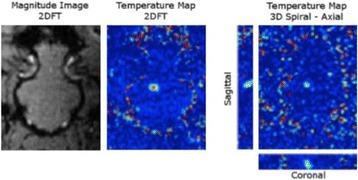
In-plane error of BBB disruption location. Different symbols represents a different animals

**Fig. 123 (abstract A114).**
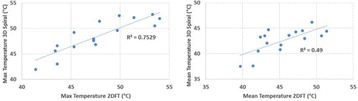
2D/3D correlations between the maximum (left) and mean (right) measured hot spot data

Sequence parameters for 3D RIO thermometry were: FA = 5-10°, TR/TE = 22.0/12.8 ms, readout length = 8 ms, interleaves = 24 over a FOV of 280 mm2 for an in-plane resolution of 1.5 mm2. 3D phase encodes = 12 with through-plane resolution of 3 mm so that the through-plane FOV was 36 mm. Total acquisition time per volume was 6.3 seconds. Sequence parameters for the 2D thermometry method were: TR/TE = 27.6/12.8 ms, pixel bandwidth = 44 Hz/px, matrix size 256 x 128 over a FOV of 280 mm2 for a resolution of 1.1 x 2.2 mm2, slice width of 3 mm, and update interval 3.5 seconds. Real-time *in vivo* thermometry images using the non-accelerated 3D RIO thermometry sequence are shown in Fig. 122. The hot spot is resolved in volumetric space, albeit with lower SNR with the spiral 3D sequence, due in part to reduced voxel size. The maximum temperatures measured by the 3D sequence correlate well with those measured by the 2D sequence (Fig. 123). The mean temporal standard deviations of the 2D and 3D sequences were 0.8 °C and 1.3 °C, respectively.

**Conclusions:** The efficiency of spiral readouts supports rapid generation of 3D temperature maps. *In vivo*, we have successfully monitored the entire ablation with adequate spatial resolution to qualitatively compare the ablation area with temperature. The temporal resolution of the 3D sequence can be further improved with the application of the Kalman filter model to match that of the standard 2D Cartesian sequence. If this combination performs well *in vivo*, the result will be a self-training 3D real-time thermometry method with excellent temporal resolution and no focal spot shift.

## A115 Water diffusivity changes with varying focused ultrasound exposure levels in rat brains

### Sijia Guo, Xin Lu, Jiachen Zhuo, Su Xu, Rao Gullapalli, Dheeraj Gandhi

#### University of Maryland Baltimore, Baltimore, Maryland, USA

**Objectives**

Focused ultrasound (FUS) for neuro-internventions is gaining popularity not only for ablating tissues as in the case of the treatment of essential tremors and Parkinson’s disease, but also to study the effects of neuromodulation, and temporary opening of Blood-Brain Barrier non-invasively. However, the biological effects of such low level exposure on the brain tissue are less understood especially at locations that are removed from the focal spot. In this study, we used *in vivo* diffusion kurtosis imaging (DKI) to measure brain water diffusion changes *in vivo* in rats at varying FUS exposure levels at the focal spot and away from the focal spot.

**Methods**

To systematically understand the rain structural, functional, and biochemical changes from focused ultrasound intervention, rats (n = 5; 300 ~ 400 g) were exposed to various FUS energies. Prior to FUS exposure, rats were imaged using an MR scanner (Bruker, Germany) for small animals. The rats were then subject to various levels of MR guided FUS exposure ranging from 1-3 W using an eight array transducer from IGT, France. Localization for the exposure was determined using ARFI technique. Once the focal spot was identified, each animal was subject to exposure at a specific energy level. Temperature monitoring was performed during the exposure to identify no over exposure or cavitation occurs in other areas of the brain. Following the exposure, high-resolution images similar to the pre-exposure were obtained within two hours after FUS. The axial temperature distribution of the focal spot was visualized with MRI during ultrasound exposure. To acquire the coronal temperature distribution of the focal spot, a k-space time-domain method was employed to simulate the acoustic field during the exposure by solving the full Westervelt equation. Temperature simulation was estimated by solving inhomogeneous Pennes equation of heat conduction.

**Results**

**Fig. 124 (abstract A115).**
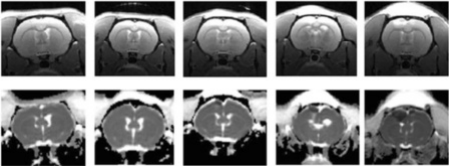
The T2 weighted (upper) and diffusion kurtosis (lower) images after 1 min ultrasound exposure are shown. The employed power from left to right is 1 W, 1.5 W, 2 W, 2.5 W and 3 W, respectively

**Fig. 125 (abstract A115).**
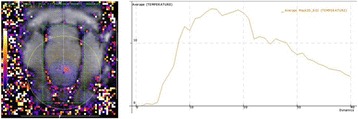
With 3 W acoustic power for 1 min, recorded temperature from MRgFUS system is shown, in which the maximum temperature rise was found to be 15 °C

**Fig. 126 (abstract A115).**
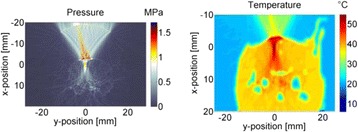
Acoustic pressure field from simulations using the bio-heat equation (left), and tissue temperature changes after taking into account the skull and tissue characteristics (right)

**Fig. 127 (abstract A115).**
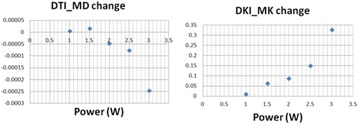
Increased acoustic power leads to a decrease in mean diffusivity (MD) and increased mean kurtosis (MK) in the focal area

The T2 weighted and diffusion tensor images after a minute of ultrasound exposure with varying energy are shown in Fig. 124. As an example, recorded temperature with 3 W acoustic power is shown in Fig. 125, in which the maximum temperature rise was found to be 15 °C. This was also confirmed from the simulations using the bio-heat equation which also takes into consideration of the skull and tissue map from CT images. The tissue temperature distribution from the simulation over the exposure time was also found to be about 15 °C (acoustic power is 3 W; base temperature is 37 °C) as well (Fig. 126). With increasing acoustic power, increased mean kurtosis (MK) and reduced mean diffusion (MD) were observed both at the focal spot and away from the focal spot (Fig. 127). Increased MK typically means that there is increased heterogeneity in the tissue microstructure suggesting increased astroglial reactivity associated with the rise in temperature. These results suggest that at even low exposure rates and away from the focal spot, where the pressures reaches ~400-500 kPa, water diffusion changes occur that can be detected by diffusion tensor imaging in the absence of any visible damage observed on T1 or T2-weighted image.

**Conclusions**

We hypothesize that these water diffusivity changes induced by focused ultrasound are mainly due to shearing forces exerted on the brain tissue that have an effect on the tissue environment if only temporarily. Ultrasound simulations could provide information on the pressure distribution and temperature distribution throughout the brain to enable correlation with water diffusion changes. In the future we will be studying the long term effects of such low level exposure to understand if such changes are similar to those observed on animals with mild traumatic brain injury follow exposure to blast pressures.

## A116 Cavitation localization using a modified trilateration method: Proof of concept

### Changzhu Jin^1^, Omer Brokman^2^, Matt Eames^1^, John Snell^1^, Dong-Guk Paeng^1^

#### ^1^Focused Ultrasound Foundation, Charlottesville, Virginia, USA; ^2^InSightec Ltd, Tirat Carmel, Israel

**Objectives**

Since cavitation is a potential risk factor that must be monitored and limited, the power measurement and frequency response of the acquired signal through passive cavitation detectors (PCDs) are utilized to detect cavitation activity. Currently, the sonication system was designed to automatically shut down when the power of the cavitation signal exceeds a certain threshold. However, cavitation caused by the bubbles on the skin could also contribute to the appearance of large broad band signal resulting in unexpected stop of sonication. Localizing the cavitation source may improve the monitoring of the cavitation activity at the focal area and provide more reliable sonication with known cavitation location. The purpose of this study is to localize cavitation near the focus using onboard PCD set without change of a current clinical system setup and to try to distinguish if the cavitation is activated in region of interest.

**Methods**

**Fig. 128 (abstract A116).**
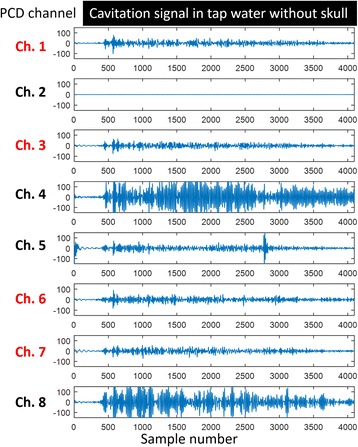
Pulse signal acquired by eight on board PCDs. The signal from channel 2 was abended because it seems not recording the cavitation signal. The signal on channel 4 and channel 8 were also excluded to the cavitation localization because its higher sensitivity may cause different pattern on time domain signal compare to others. A large unknown signal was frequently appeared at the begging of the recorded signal on channel 5. The signal from channel 1, 3, 6 and 7 shows similar pattern and carried for further cavitation localization process

**Fig. 129 (abstract A116).**
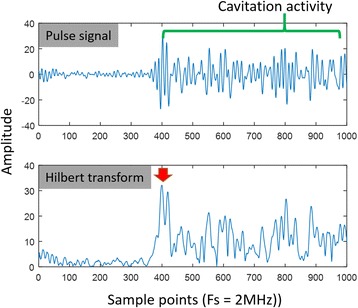
Time domain signal of one pulse. The large signal represents the cavitation activity and we assume the first peak of the large signal group as the initiation of the cavitation activity

In order to generate the cavitation intentionally, high power sonication was targeted on the geometrical center in tap water with and without a human skull. Eight PCDs on a 220 kHz hemispherical transducer (ExAblate Neuro system, InSightec) were synchronized with the pulse sonication. The PCD signals were recorded each sonication and saved into a log file. One of the pulse signal (4096 sample points with 2 MHz sampling rate) acquired by eight the PCDs are shown in Fig. 128. The relatively large signals are from cavitation and we assume the first peak (marked with red arrow in Fig. 129) of this large signal train as initiation of the cavitation activity. The mean value of the first 200 samples and a constant value was used as a threshold to track the first arrived signal. A trilateration, which is normally utilized as a standard localization method on global positioning system (GPS), was modified to localize the cavitation spot. The cavitation signals from 4 PCDs were read in MATLAB software to implement the cavitation localization. The distance between tracked location and sonication location was calculated to verify the localization error.

**Results**

**Fig. 130 (abstract A116).**
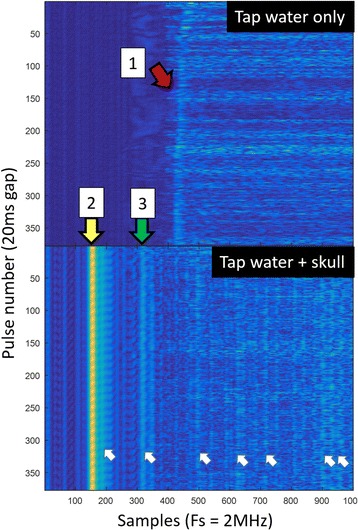
The sampled signals on one passive cavitation detector (PCD) in tap water with and without skull. The x axis denotes the sample points and y axis denotes the pulse number, and the brighter color represent the larger amplitude of the signal

**Fig. 131 (abstract A116).**
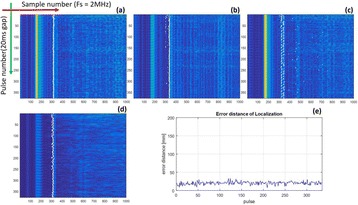
Tracking of the signals of cavitation initiation for 4 PCDs and the corresponding localization errors. (**a**) to (**d**) denotes the tracking result of the cavitation signals from 4 PCDs marked with white dots. The error distances between the known source positions and localized ones with a modified trilateration algorithm are shown in (**e**)

We found time shifting of the cavitated signals through a skull compared to the data without skull. As shown in Fig. 130, the red arrow denotes the arriving time on tap water data (without skull) and green arrow shows the shifted signals through the skull. And the strong signals pointed by yellow arrow is the reflected signals from skull. The simulation signals using a k-wave model result in cavitation localization with an initial errors of 3 ± 3 mm. Implementation of this localization algorithm to experimental data made the cavitation localization with larger errors of 49 ± 21 mm in tap water (without skull) compared to the water with skull (20 ± 4 mm shown in Fig. 131). It may be because of the signal patterns of the skull embedded data containing aforementioned discernible-vertical line (marked with white arrow in Fig. 130) and has higher SNR near the arriving time.

**Conclusions**

In conclusion, the cavitation localization could be possible with an error range of 20 ± 4 mm with skull with some limitations. So far, we verified the localization algorithm based on the experimental data targeted at the geometrical center only, and more off-centered data are required to confirm the robustness of this localization algorithm.

## A117 Anesthetic depth challenges for ultrasonic neuromodulation: An electroencephalographic index monitoring

### Hongchae Baek, Hyungmin Kim

#### Korea Institute of Science and Technology, Seoul, Republic of Korea

**Objectives**

Transcranial application of low-intensity focused ultrasound (FUS) for brain stimulation has proven to be powerful tool to manipulate brain activity with a few millimeters of spatial resolution while keeping it completely non-invasive (Yoo et al., 2011). Until recently, however, transcranial FUS-mediated brain stimulation conceded some room for variability and inconsistency in exerting its neuromodulatory effects; it is unclear how different types of anesthetic drugs and varying anesthetic depth intervene in the process of ultrasonic neuromodulation. For these reasons, it is necessary to discover the effective time window for neuromodulation. The purpose of the present study is to monitor the anesthetic depth with electroencephalogram (EEG) to optimize the working parameters for neuromodulation in different anesthetic periods.

**Methods**

**Fig. 132 (abstract A117).**
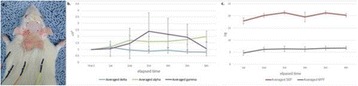
**a** Placement two frontal EEG electrodes with ground and reference electrodes. **b** The normalized percentage of total power in delta, alpha, and gamma frequency bins. **c** SEF 95 % and MPF indexes were calculated from each period

Sprague-Dawley rats (female, n = 8) were induced to anesthesia with 5 % of isoflurane, and sustained anesthesia by intraperitoneal injection of 0.1 ml/100 g ketamine/xylazine cocktail. Power spectra of two EEG channels were obtained from prefrontal cortex (Fp1, Fp2), and one ground electrode were placed at midline of the cerebellum (Fig. 132a). We adopted 350 kHz of fundamental frequency, 50 % of duty cycle, 1 kHz of pulse-repetition frequency, 0.5 ms of tone-burst duration, and 300 ms of sonication duration, and acoustic intensity of 3.3 W/cm2 Ispta (spatial-peak temporal-average intensity) as the sonication parameters for inducing the tail movement (Kim et al., 2014). EEG was recorded approximately for an hour until the animal wakes up, and each experimental recording was divided into 6 consecutive periods to calculate the depth of anesthesia. From EEG spectrum analysis, SEF 95 % (spectral edge frequency), MPF (median power frequency), and vigilance index, and other spectral parameters were used to estimate the depth of anesthesia.

**Results**

At the 3thperiod, EEG delta and alpha activity were strongly suppressed while gamma activity was significantly enhanced during the time (Fig. 132b), which indicate rapid eyeball movement (REM) sleep like EEG activity (Ferri et al., 2001). At this stage of anesthesia, even at the intensity level above the previously confirmed excitation threshold did not elicit any tail movement as stimulating the motor cortex. This may be translated as the results of REM stage characteristics where the external stimuli are strongly inhibited. During the 5th and 6th periods, SEF 95 % and MPF repeated increasing tendency (Fig. 132c) from briefly deepened 4thanesthetic period after 3th period (REM stage) implying lightened anesthetic depth (Kortelainen et al., 2012).

**Conclusions**

We observed voluntary whisker movements at 5th and 6th period which is a typical sign for superficial depth of anesthesia (Brecht et al., 2004), and the ultrasound induced tail movement success rates accompanying the whisker movements were higher at both periods than the rest of the anesthetic stages.

## A118 The effect of skull model resolution on phase aberration correction

### Steven Leung, Taylor Webb, Kim Butts Pauly

#### Stanford University, Stanford, California, USA

**Objectives**

Skull bone is heterogeneous and distorts ultrasound waves. This alters the location, shape, and itensity of the beam’s focus. To compensate for skull distortions, phase corrections may be applied to each element - these corrections are estimated using simulations of the patient skull.

Traditionally, models of the patient skull are derived from high resolution computed tomography (CT) images. However, there is a move towards ultra-short echo time (UTE) magnetic resonance imaging to reduce patient exposure to ionizing radiation.

UTE cannot achieve the same resolution as CT, therefore it is unclear whether simulations based on UTE-derived skull models will yield accurate phase corrections. To determine the feasibility of the lower resolution UTE-based simulations, we investigate the trade-offs between skull model resolution and characteristics of the corrected beam. In this study, we use CT images to create skull models of varying resolution.

**Methods**

We performed 3D simulations using the hybrid angular spectrum (HAS) method at 680 kHz. Skull models were generated by down-sampling high resolution skull CT images with cubic convolution interpolation. Isotropic resolutions of 0.5 mm to 3 mm (in 0.5 mm steps) were used.

To compensate for phase distortion introduced by the skull, the phase of each transducer element was estimated at the intended location. We applied the conjugate of this phase to achieve a phase correction. A different set of phases was calculated at each resolution, and the resulting beam was simulated using the full resolution CT model.

We define a global efficiency metric as the ratio of focal spot intensity to total intensity in the brain; this metric detects the presence of large sidelobes or multiple focal spots.

**Results**

**Fig. 133 (abstract A118).**
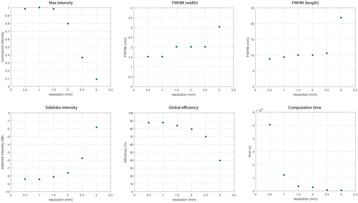
Metrics used to characterize trade-offs between skull model resolution and characteristics of the corrected beam

**Fig. 134 (abstract A118).**
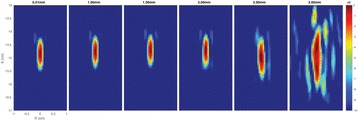
Coronal beam profiles at each skull model resolution. A different set of phases was calculated at each resolution, and the resulting beam was simulated using the full resolution CT model

With decreasing resolution, full width at half maximum and sidelobe level increase whereas focal intensity and global efficiency decrease (Fig. 133). There is minimal blurring of the focal spot for resolutions 0.5 mm to 1.5 mm. However, the focal spot quickly degrades as the voxel size exceeds 1.5 mm (Fig. 134).

**Conclusions**

Focal intensity, sidelobe level, and global efficiency worsen at resolutions 2 mm and below. Even though computation time is drastically shorter, the deterioration of the focal spot is unacceptable.

To avoid frequency aliasing in the HAS method, the minimum resolution should be 1.1 mm (assuming 680 kHz frequency and 1500 m/s speed of sound). The 1.5 mm and 2 mm resolutions will thus lead to aliasing. The 1.5 mm option, however, appears to yield similar results to the higher resolution options. This may be plausible considering that the acoustic wavelength in bone is approximately 3.4 mm (assuming 2300 m/s speed of sound). 1.5 mm would satisfy the resolution requirement in the skull, which is the dominant contributor to phase aberrations. Therefore, a resolution cutoff is suggested at around 1.5 mm, with additional work recommended to determine the minimum effective resolution.

## A119 The effects of oxygen on ultrasound-induced Blood-Brain Barrier disruption in mice

### Nathan McDannold^1^, Yong-Zhi Zhang^2^, Natalia Vykhodtseva^1^

#### ^1^Brigham and Women’s Hospital, Harvard Medical School, Boston, Massachusetts, USA; ^2^Harvard Medical School, Boston, Massachusetts, USA

**Objectives**

This study investigated the impact of oxygen *versus* medical air as a carrier gas for isoflurane anesthesia on Blood-Brain Barrier (BBB) disruption, a method under investigation that can enable and target the delivery of drugs to the brain.

**Methods**

In experiments in mice (N = 7), four brain targets were sonicated in combination with administration of Optison microbubbles (100 μl/kg) under isoflurane anesthesia. A 690 kHz focused ultrasound transducer applied 10 ms bursts at 1 Hz for two minutes at applied peak pressure amplitudes of 0.51-0.54 MPa (N = 2) or 0.34-0.36 MPa (N = 5). After sonication of two locations in one hemisphere, the carrier gas for the anesthesia was changed from oxygen to medical air, or vice versa, and the sonications were repeated in the contralateral hemisphere. Acoustic emissions were recorded during each sonication, and the resulting BBB disruption was assessed using contrast-enhanced MRI.

**Results**

**Fig. 135 (abstract A119).**
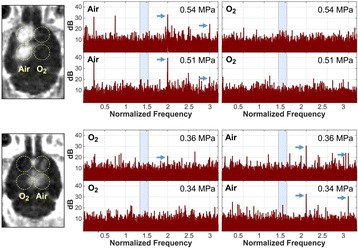
Example MRI contrast enhancement (*left*) and acoustic emission spectra (*right*) in 2 mice. Only animals breathing medical air showed detectable BBB disruption and large harmonic emissions (*arrows*)

**Fig. 136 (abstract A119).**
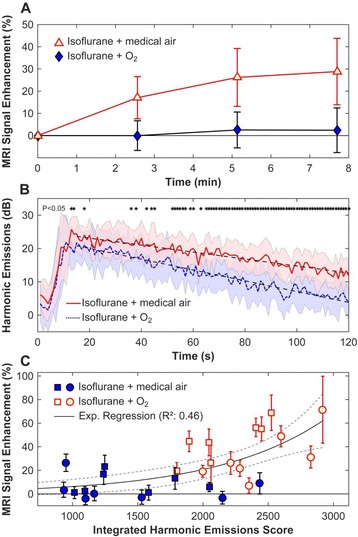
**a** Mean MRI signal enhancement vs time for 0.34-0.36 MPa sonication. **b** Mean harmonic emission strength (relative to recordings obtained without microbubbles) vs time for 0.34-0.36 MPa sonication. **c** MRI enhancement vs harmonics for all sonications

Example findings are shown in Fig. 135. BBB disruption, as measured by MRI signal enhancement after Gd-DTPA administration, was significantly greater (P < 0.001) in animals breathing medical air (Fig. 136b). MRI signal enhancement was detected in 13/14 locations sonicated in animals breathing medical air, but only in 7/14 locations with oxygen. Harmonic emissions were also greater with medical air (P < 0.001), and the decay rate of the harmonic emissions was 1.5 times higher with oxygen (Fig. 136b). A good correlation (R^2^: 0.46) was observed between MRI signal enhancement after Gd-DTPA administration and harmonic emissions (Fig. 136c). At 0.51-0.54 MPa, both the occurrence and strength of wideband emissions with greater with medical air. However, at 0.34-0.36 MPa, the strength and probability for such emissions was higher with oxygen. Little or no effects were observed in histology at 0.34-0.36 MPa.

**Conclusions**

The use of oxygen as a carrier gas has a profound impact on BBB disruption. These findings are consistent with earlier work comparing ketamine/xylazine *versus* isoflurane and oxygen anesthesia on BBB disruption as well as imaging studies showing increased clearance rates of ultrasound contrast agents in animals breathing oxygen. The increase in wideband emissions at the lower exposure levels with oxygen without significant BBThis work was supported by NIH grants R01EB003268, P01CA174645B disruption is an interesting finding that should be pursued further. Overall these findings are important for comparing results from different research groups and should be taken into account in translation to clinical trials.

## A120 Using ray tracing to predict histotripsy focal shift in a transcranial setting

### Thai-Son Nguyen, Jonathan Sukovich, Timothy Hall, Zhen Xu, Charles Cain

#### University of Michigan, Ann Arbor, Michigan, USA

**Objectives**

Previous experiments with histotripsy has shown that it can be used to generate targeted lesions through the skullcap without aberration correction. However, before therapy is applied, one would like be sure that the focal point of the transducer through the skullcap is within the confines of the treatment volume. The results from this study demonstrate how ray tracing can be used to predict the location of the focal point in a transcranial setting.

**Methods**

Histotripsy pulses for the experiments were delivered through human skullcaps using a 256-element 500 kHz hemispherical phased array transducer. Three excised human skullcaps were used for the experiments. During experiments, measurements of pressure profiles and focal shifts of the transducer through the skull were acquired using a needle hydrophone. The pressure profiles were measured by scanning the hydrophone along 2 cm long lines across the axial and transverse directions with respect to the origin of array. The experiments were performed without aberration correction. 3D surface scans of the excised skullcaps were acquired for the simulations. During simulations, straight line rays from each transducer element of the array were propagated through the skull. Calculations of ray trajectories based on Snell’s law, the time of flight of the rays, and losses due to attenuation as the rays propagated through the skull were made during simulations. The rays were then simulated as plane waves that propagated across the origin of the array to calculate the pressure field about origin. The results from the simulations were then compared to the results from the experiments.

**Results**

Experimental results revealed that the focal point of the transducer remained confined to less than 1 mm of the geometric origin of the array. The experiments also revealed that the propagation direction of acoustic pulses from each element of the transducer were not significantly altered when traveling through the skull. The simulations using ray tracing were able to accurately predict the overall shape of the pressure profile measured experimentally and the focal position of transducer through the skullcap.

**Conclusions**

The focal location of the transducer was not significantly affected by the skull even when the skullcap was moved within the array. Ray tracing was able to accurately predict the major features of the pressure profile and focal locations through the skull.

## A121 Bilateral anterior capsulotomy with MR-guided Focused Ultrasound for refractory obsessive-compulsive disorder: 1 year follow-up results

### Chang Kyu Park^1^, Sang Man Park^1^, Na Young Jung^1^, Min Soo Kim^1^, Won Seok Chang^1^, Hyun Ho Jung^1^, Jin Woo Chang^2^

#### ^1^Severance Hospital, Yonsei University, Seoul, Republic of Korea; ^2^YUMC Severance Hospital, Seoul, Republic of Korea

**Objectives**

Obsessive-compulsive disorder (OCD) is a chronic and debilitating psychiatric condition. Traditionally, anterior capsulotomy was an established procedure for treatment of patients with refractory OCD. However, despite the potential benefits of these surgical procedures, patients show significant surgery-related complications. Previously, we demonstrated that bilateral thermal capsulotomy using magnetic resonance-guided focused ultrasound (MRgFUS), a non-invasive technique with surgical precision for thermal ablation, is a safe and effective therapy for patients with refractory OCD. In the present study, we investigated the long-term outcome of the patients who underwent MRgFUS for refractory OCD.

**Methods**

Eleven medically intractable OCD patients who underwent MRgFUS bilateral thermal anterior capsulotomy from February 2013 to June 2014 were included in this study. Pre- and post-operative (1 week, 1 month, 3 months, 6 months, 12 months) Yale-Brown Obsessive-Compulsive Scale (Y-BOCS), Hamilton Depression Scale (HAMD) and Hamilton Anxiety Scale (HAMA) scores were recorded. Brain MRI was performed pre-operatively, 1 week, 1 month, 3 months and 6 months after the operation to assess the effect of the operation.

**Results**

There was a 36 % improvement in Y-BOCS at 12 months after MRgFUS. The HAMD and the HAMA were showed 54 % and 62 % improvement compared to pre-operative score. The improvement according to time had shown statistical significance (all p value was less than 0.001, repeated ANOVA test). There was a marked difference in MRI findings before and 6 months after the operation. Moreover, there were no neurological, psychological complications with the exception of mild headache and nausea.

**Conclusions**

The optimal treatment for refractory OCD has been unclear; however, in this study, MRgFUS was an effective and safe treatment for refractory OCD. Thus, MRgFUS may be an effective treatment modality for refractory OCD in high risk surgery patients.

## A122 Prediction of shear-mode conversion for transcranial ultrasound using a viscoelastic model for sound propagation: First experimental results

### Samuel Pichardo^1^, Kullervo Hynynen^2^

#### ^1^Thunder Bay Regional Research Institute, Thunder Bay, Ontario, Canada; ^2^Sunnybrook Health Sciences Centre, Toronto, Ontario, Canada

**Objectives**

The skull barrier represents the main limitation for many therapeutic applications of High Intensity Focused Ultrasound (HIFU) in the brain tissue. A better understanding and modelling of this barrier facilitates an optimal design of transcranial HIFU applications. An important aspect not yet fully explored in transcranial ultrasound is the use of shear-mode conversion, which in principle can result in less defocusing effects caused by the skull barrier.

However, there is not yet a well-established modeling method that can take into account both longitudinal and shear waves in the skull bone. In this study we present our first results on the validation of a sound transmission model based on the viscoelastic wave propagation adapted for the case of transcranial ultrasound. We compared the predictions obtained with this model with measurements in human calvaria where shear mode conversion was present in the transcranial sound transmission.

**Methods**

Characterization of transcranial acoustic transmission. Human skull caps were immersed in degassed water and put under vacuum at −0.7 MPa for 8 h prior to measurements. A transducer (diameter of 5 cm and a focal length of 10 cm) was used to send 15 ultrasound pulses with a frequency of 270 kHz. A stereoscopic fixation system was used to ensure an incident angle of ultrasound of 40°. The skull was located 12 cm from the transducer. Measurements were performed in the presence of the skull and in water-only conditions using a 2 mm-diameter hydrophone over a scan plan of 35 mm x 35 mm with a spatial step of half-wavelength and located 15 cm from the transducer.

Viscoelastic wave propagation modeling for transcranial ultrasound transmission. The propagation model is based on a staggered grid scheme (Geophysics, 1986; 51(4): 889-90). We included refinements to the original model to consider transmission between fluids and solids (Computers & Geosciences, 2002; 28(8): 887-99) and attenuation effects (Geophysics, 1995; 60(1): 176-184). The equations were solved using a Finite Difference Time Difference (FDTD) solution using approximations of second order in time and fourth order in space.

**Results**

**Fig. 137 (abstract A122).**
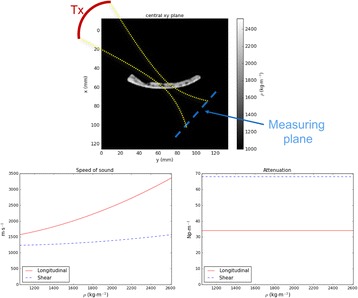
Experimental setup (top) for shear-mode transcranial transmission and functions (bottom) based on the skull density for longitudinal and shear speed of sound, and longitudinal and shear attenuation. Tx refers to the transducer

Figure 137 shows a scheme for the experimental setup and the relationships in function of the skull density of the longitudinal and shear speed of sound, and longitudinal and shear attenuation, which were used for this feasibility study. The simulated delay DS on the scan plane between water-only and skull measurements was compared to the corresponding experimental delay DE. Negative values of delay indicated that signals in the presence of the skull arrived before their respective water-only counterparts. Comparison was done in ten measurement locations from three different skull samples.

**Fig. 138 (abstract A122).**
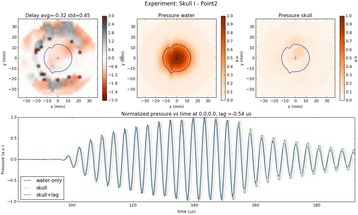
Example of experimental maps of delay and pressure amplitude for skull location showing shear-mode conversion. Average of delay was calculated in the isoregion of 50 % or more (contour showed in maps) of the maximal pressure in the water measurement

**Fig. 139 (abstract A122).**
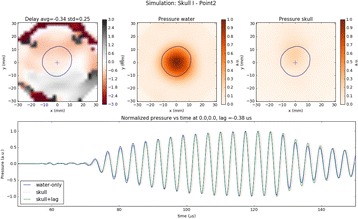
Corresponding simulation of maps of delay and pressure amplitude for skull location showing shear-mode conversion. Average of delay was calculated in the isoregion of 50 % or more (contour showed in maps) of the maximal pressure in the water measurement

**Fig. 140 (abstract A122).**
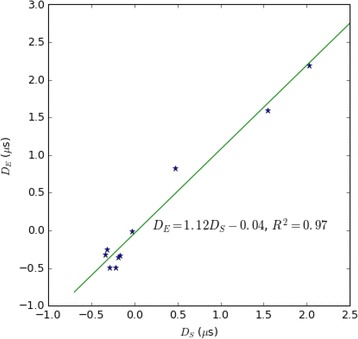
Linear regression of experimental and simulated delay

Figures 138 and 139 shows an example of experimental and simulated results where shear-mode conversion was clearly observed. Each figure shows the delay and normalized pressure maps. Also, plots of the acoustic signals at the center of scan plane are shown in water conditions, in the presence of the skull and in the presence of the skull plus the calculated delay. All measurements combined, seven of ten measurements showed shear-mode conversion (negative delay) while three others (all from skull #3) still showed that longitudinal transmission dominated (positive delay). The linear regression analysis (Fig. 140) of DS *versus* DE indicated that viscoelastic model predicted accurately the delay for all cases with R2 = 0.97 and a significance of p < 1e-5.

**Conclusions**

The proposed model represents a significant progress for a more accurate prediction of transcranial ultrasound transmission. The comparison with experimental results indicated that the model is adequate to predict sound propagation when either shear or longitudinal transmission dominates. In our tests, two of three skull samples clearly showed shear-mode conversion while the other did not. Closer inspection of skull #3 indicated a less differentiated cortical bone layer and a density 20 % lower compared to the other two specimens. As a result, the average longitudinal speed of sound of skull #3 was slower compared to the other two specimens, implying that its critical angle for shear-mode conversion should be higher than 40°. These results indicated how important can be the degree of differentiation among different subjects. An adequate modelling that considers the skull bone as a viscoelastic medium will be an important asset to better design and execute transcranial HIFU therapy.

## A123 Comprehensive dissection of ultrasonic neuromodulation biophysics predicts cell-type-selective stimulation

### Michael Plaksin, Yoni Weissler, Shy Shoham, Eitan Kimmel

#### Technion – Israel Institute of Technology, Haifa, Israel

**Objectives**

US can noninvasively suppress or excite central nervous system (CNS) activity using different combinations of stimulation parameters. While applications are already emerging, the underlying biophysics remains unclear regarding the relative contribution of possible mechanisms: extracellular bubble cavitation, thermal effects, acoustic radiation pressure and US-induced intramembrane cavitation within the bilayer membrane (the bilayer sonophore or BLS model). Interestingly, temperature transients, radiation pressure and intramembrane cavitation can induce plasma membrane capacitance changes. Here, we use detailed predictive modelling and find that only intramembrane cavitation can explain all the observed aspects of ultrasonic neuromodulation.

**Methods**

We analyzed the relevant experimental literature using modified Rayleigh–Plesset intramembrane cavitation BLS biomechanics and acoustic radiation pressure gradients (RPG) - induced membrane dynamics. By coupling these biomechanical models to biophysical membrane models we predict dynamical biophysical responses of artificial bilayer membranes, and of three common neocortical single cell Hodgkin-Huxley type models: i) Regular Spiking (RS) cortical pyramidal neuron, ii) Fast Spiking (FS) cortical inhibitory neuron and iii) Low Threshold Spiking (LTS) cortical inhibitory neuron, RS-FS-LTS Hodgkin- Huxley based network model and CNS axon model. In addition, live brain tissue RPG subjected areal strains were evaluated in a viscoelastic brain model. To explore high intensity US-related thermal effects on membranes’ biophysics a Gouy-Chapman-Stern-based model was developed.

**Results**

Only the Neuronal Intramembrane Cavitation Excitation (NICE) models were able to explain US-induced action potential generation through BLS-type pulsating nano-bubbles inside the bilayer plasma membrane: the leaflets’ periodic vibrations induce US-frequency membrane capacitance and potential oscillations, leading to slow charge accumulation across the membrane (on a time scale of tens of milliseconds), until action potentials are generated. In contrast, the analysis of RPG-induced membrane capacitance variations associated with membrane area changes explain artificial membrane results, but were found to be highly unlikely sources for neural excitation, when considering the areal strains expected to form in brain tissue during normal sonication. Further, the NICE-LTS inhibitory neurons show a much higher relative sensitivity to sparse ultrasonic stimulation compared to the other neurons, resulting from their T-type voltage gated calcium channels. This model-based prediction was found to explain the results of a significant body of suppression and excitation experimental studies, including in humans. Finally, a rigorous analysis of thermal effects using Gouy-Chapman-Stern theory reveals that an elevation in membrane capacitance coupled with surface charge-related thermal processes can be associated with high intensity US’s temperature transients.

**Conclusions**

This study provides a unified theoretical framework for a large body of experiments in multiple preparations across the field of US neuromodulation, lending further support to the hypothesis that intramembrane cavitation is responsible for ultrasonic neuromodulation. They could thus pave the way towards new CNS therapeutic protocols, using the only method that currently allows targeted non-invasive neuromodulation with millimeter spatial resolution essentially anywhere in the brain.

## A124 Proportional integral temperature control during Focused Ultrasound treatment with clinical systems in the brain

### Anders Quigg^1^, John Snell^1^, Dong-Guk Paeng^1,2,3^, Matt Eames^1^

#### ^1^Focused Ultrasound Foundation, Charlottesville, Virginia, USA; ^2^University of Virginia, Charlottesville, Virginia, USA; ^3^Jeju National University, Jeju, Republic of Korea

**Objectives**

Current clinical systems for Focused Ultrasound (FUS) brain surgeries are not designed to precisely or accurately deliver a desired thermal dose to a target in brain tissue. This study attempts to modulate the power applied during sonications to maintain a target temperature in order to accumulate thermal dose in a predictable and controllable fashion, allowing researchers and clinicians to prescribe a delivered thermal dose.

**Methods**

The regular gating and control of an MRI-guided FUS brain system (ExAblate 4000 Neuro 650 kHz system, InSightec) was bypassed and fed into a proportional-integral-derivative (PID) control loop, and empirically tuned during sonications of gel phantoms with a proportional gain of 5 and an integral gain of 1.5. The system was then used for an animal trial with pigs *in vivo*, performing thalamus ablation following a craniectomy.

**Results**

Trials in gel phantoms demonstrated the effectiveness of PID temperature control with the brain system, with different settings for proportional and integral gain affecting the time to reach the target temperature and the magnitude of the temperature overshoot. When used *in vivo*, the system was able to deliver a desired thermal dose within 10 CEM, with lower temperatures demonstrating a lower rate of accumulation of dose.

**Conclusions**

In conclusion, PID temperature control is possible to implement in current FUS brain systems, and can be used to deliver a desired thermal dose with the principle limitations of uncertainty in starting temperature and the low signal to noise ratio of the MRI-thermometry.

## A125 Evaluation of nonlinear effects in the acoustic field of a hemispherical focusing transducer for ultrasound applications in brain

### Oleg Sapozhnikov^1^, Pavel B. Rosnitskiy^2^, Vera Khokhlova^1^

#### ^1^University of Washington, Seattle, Washington, USA; ^2^Moscow State University, Moscow, Russian Federation

**Objectives**

Ultrasound treatment of the brain is typically performed using large-aperture submegahertz-range sources of a hemispherical shape to achieve better energy concentration. Heat deposition at the focus depends on the frequency content of the ultrasound wave that can be different from the source single-frequency signal due to nonlinear generation of harmonics on the way to the focus. Several theoretical models exist for simulating generation of higher harmonics for weakly or moderately focused waves. In this case the acoustic field can be considered as a directed beam. In the case of the hemispherical sources this assumption is no longer valid, so different strategies are needed.

**Methods**

Theoretical methods are proposed for evaluating the amplitude level of the second harmonic generated in hemispherically focused fields. Two approaches are used: 1) a simplified nonlinear 1D model; and 2) a more accurate model that accounts for the combined effect of diffraction and nonlinearity. In the first approach, the nonlinear propagation is studied by considering a spherically converging nonlinear wave for which an exact solution of the generalized Burgers equation can be obtained [R.V. Khokhlov et al., Acustica 14 (1964)]. The solution is used to predict the degree of nonlinear effects when the wave arrives to the focal region. In the second approach, the calculation consists of several steps and employs the successive approximations method. First, the linear acoustic problem is solved for a hemispherical source using a spherical harmonics series expansion [O.A. Sapozhnikov and T.V. Sinilo, Acoust. Phys. 48 (2002)]. Then the volume sources for the second harmonic generation are calculated based on the solution to the linear problem. Finally, these sources are used for obtaining an analytic solution for the second harmonic amplitude at the focus.

**Results**

The developed methods were applied to study the acoustic field of the ExAblate 3000 clinical system (InSightec, Haifa, Israel). This source has the following parameters: 512 equal area elements, hemispherical shape of 30 cm diameter, working frequency of 670 kHz, and acoustic power of several hundreds watts [K. Hynynen et al., Europ. J. Radiology 59 (2006)]. The spherical harmonics expansion approach shows that the linear field can be accurately calculated using the Rayleigh integral. Calculations by both models predict that nonlinear effects are weak even for the free-field focusing in water at the acoustic power up to approximately 1 kW. Around this power level, a noticeable waveform distortion, with 10 % of the wave power being transferred to the higher harmonics, is predicted at the focus. The focal pressure in this regime is on the order of tens of MPa.

**Conclusions**

Typical hemispherical sources of submegahertz frequencies (ExAblate 3000) operate in almost linear regime up to the acoustic power levels of several kilowatts. In clinical applications, even less effect of nonlinearity is expected, as the effective power is much lower due to losses in the skull and brain tissue.

## A126 Research platform for rodent studies of wavefront engineered ultrasonic neuromodulation

### Shy Shoham^1^, Steve Krupa^2^, Eilon Hazan^2^, Omer Naor^2^, Yoav Levy^1^, Noam Maimon^1^, Inbar Brosh^2^, Eitan Kimmel^2^, Itamar Kahn^2^

#### ^1^InSightec Ltd, Tirat Carmel, Israel; ^2^Technion - Israel Institute of Technology, Haifa, Israel

**Objectives**

Recent years have seen intensifying scientific and clinical interest in US neuromodulation of CNS circuits, and relevant empirical data is rapidly accumulating. To date, studies in this field, and in particular rodent studies, employed single-element transducers. The introduction of wavefront engineering techniques based on phased ultrasonic arrays could open up new vistas for this field, including the generation of multi-focal patterned stimulation.

**Methods**

We adapted an Insightec prostate phased array (986 phase elements over 2.5x4 cm^2, 2.3 MHz center frequency) to interface with several custom holders for either *in vitro* and *in vivo* work. For *in vitro* recording we coupled the transducer to a 256-channel planar multi-electrode array (Multichannel systems) and recording system (Ripple Technologies). For *in vivo* work we designed custom couplers and calibration procedures for operating the system over head-fixed rodents, allowing high-accuracy targeting of the sub-mm focal spot using either marker-based or MR-guidance in a 9.4 T small-animal MRI (Bruker). For generating accurate distributed acoustic phase holograms we applied our recent mathematical framework, based on adaptations from optical computer-generated holography.

**Results**

The system generates steerable foci whose dimensions are 0.6 x 0.8 x 3 mm (FWHM) in an open water bath, and 0.7 x 0.9 x 4.5 mm through the top portion of a mouse skull, with a sub-mm targeting accuracy. Excitation foci and holograms can be flexibly generated and switched every 100 ms.

We observed and characterized stimulus-locked neuronal responses in extended-duration US pulses to different neural systems. In isolated mouse retinas, we observed highly structured off-responses from retinal ganglion cell single-units. In live rats and mice we observed motor responses of the animal’s limbs and distributed BOLD responses to cortical stimulation.

**Conclusions**

Our early results and the system’s versatile ability to steer and pattern acoustic fields on a sub-mm scale may open new vistas in the study of causal US neuromodulation in relevant small animal models. These abilities can possibly help advance multiple aspects of US neuromodulation, including target optimization and dissecting the underlying biophysical mechanisms.

## A127 Efficacy and treatment envelope of transcranial histotripsy therapy without using aberration correction

### Jonathan Sukovich, Zhen Xu, Timothy Hall, Steven Allen, Charles Cain

#### University of Michigan, Ann Arbor, Michigan, USA

**Objectives**

Histotripsy is an ultrasound therapy that generates cavitation bubble clouds to fractionate soft tissue using short duration, high-amplitude ultrasound pulses. Previous studies have shown that histotripsy is capable of producing lesions through the skullcap using aberration correction. The goals of this study are to demonstrate the ability of histotripsy to generate targeted, precise single lesions through the skull without using aberration correction and to show that larger lesions of arbitrary shapes and sizes can be generated through the skull using either mechanical or phased array steering without aberration correction. We also plan to establish the treatment location envelope, e.g. proximity to the skull surface, over which lesion generation may be accomplished using histotripsy through the skull without aberration correction.

**Methods**

Histotripsy therapy was delivered using a 500 kHz, 256-element hemispherical phased array transducer with an aperture diameter of 30 cm and a focal distance of 15 cm fabricated in our lab. This transducer is capable of producing estimated peak rarefactional pressures in the free field in excess of 200 MPa with pulse durations of <2 acoustic cycles. For the single lesion generation study, targeted single lesions were generated in red-blood cell (RBC) tissue phantoms through three excised human skulls using histotripsy pulses delivered at a PRF of 1Hz, with estimated peak rarefactional pressures in the focal zone through the skull of 31-38 MPa. Lesions were examined by optical imaging during treatment. During the treatment envelope and large lesion studies, histotripsy pulses were delivered at a PRF of 200Hz using a combination of electrical focal steering and mechanical steering to target volumes with characteristic dimensions up to 50 mm, extending up to the skull’s interior surface, at locations near the front, back, top, side, and base of the skull. During these experiments, the skull’s volume was filled with RBC phantom, and lesion generation was evaluated following treatment via MR imaging. No aberration correction was used.

**Results**

Histotripsy was able to generate precise, single lesions with radii ≤1 mm in the RBC phantoms without using aberration correction through all skull samples tested. Lesion generation was consistently observed at the geometric focus of the transducer even as the position of the skullcap with respect to the transducer was varied. During large lesion studies, lesions with characteristic dimensions up to 5 cm were generated. Treatment envelope studies revealed that lesion generation was consistently possible at depths of 5-10 mm from the interior skull surface at all locations targeted.

**Conclusions**

These results show that histotripsy may be used to generate targeted, arbitrarily shaped lesions through intact human skulls without using aberration correction. They demonstrate that both mechanical and phased array steering strategies may be used for lesion generation, and that the treatment envelope for lesion generations extends to within as little as 5 mm from the skull’s interior surface. Such capability has the potential to greatly simplify and expand the target window of transcranial ultrasound therapies for non-invasive transcranial applications.

## A128 Non-invasive targeted delivery of GABA for neuromodulation: evaluation by resting state functional MRI

### Jessica Cahill^1^, Tao Sun^2^, Yong-Zhi Zhang^3^, Chanikarn Power^2^, Margaret Livingstone^3^, Nathan McDannold^2^, Nick Todd^4^

#### ^1^Georgia Institute of Technology, Atlanta, Georgia, USA; ^2^Brigham and Women’s Hospital, Harvard Medical School, Boston, Massachusetts, USA; ^3^Harvard Medical School, Boston, Massachusetts, USA; ^4^Brigham and Women’s Hospital, Boston, Massachusetts, USA

**Objectives**

We aim to develop novel methodology to non-invasively deliver neurotransmitter chemicals to spatially localized brain regions for modulation of neuronal activity. In this approach, focused ultrasound in conjunction with microbubble injection is used to transiently open the Blood-Brain Barrier (BBB) in a localized region of the rat brain. Neurotransmitter chemicals, such as GABA or glutamate, are then systemically injected such that they leak out and have an effect on neuronal activity only at the site of BBB disruption.

We have previously used electrophysiology measurements to demonstrate functional blockade via BBB disruption and GABA administration. The current study will use resting state functional MRI (rs-fMRI) to evaluate the effects of GABA delivery to the rodent thalamus on resting state networks.

**Methods**

5 - 10 Sprague-Dawley rats will be imaged under resting state conditions using a 2x2 factorial design with conditions of BBB Open vs BBB Closed and GABA Injected vs No GABA Injected. The case of BBB Closed and No GABA Injected will be considered the baseline state. The other three cases will be compared against this baseline for changes in activation patterns.

BBB opening: Right thalamus targeted. Microbubbles injected (Optison, 200 μl/kg), 690 kHz transcranial FUS with 10 ms bursts applied at 1 Hz for 60 seconds.

GABA delivery: Systemic tail vein injection of 20 mg/kg bolus followed by 5 mg/kg/min infusion.

rs-fMRI: Images acquired on a Bruker 7 T scanner with a single shot EPI sequence (TR = 1.5 s, TE = 18 ms, 18 slices, 280 images). Continuous infusion of Dexmedetomidine used for anesthesia to ensure light anesthetic state. rs-fMRI temporal correlation analysis performed as described by Pawela et al (MRM 2008).

**Results**

This is a prospective study to be carried out by Jessica Cahill during her summer internship at the Focused Ultrasound Laboratory. We hypothesize the following: 1) that we will see robust resting state network activation in the baseline case; 2) that the resting state networks seen for the cases of BBB Open and No GABA Injected, and BBB Closed and GABA Injected will show little to no differences relative to the baseline; 3) that the resting state networks seen for the case of BBB Open and GABA Injected will be significantly different than the baseline case. We will not speculate on how these differences will manifest.

**Conclusions**

Resting state fMRI consistently shows that BOLD signal fluctuations are temporally correlated in brain regions that make up functional networks, such as the visual or sensorimotor systems. Demonstrating that non-invasive targeted delivery of GABA via FUS-mediated BBB opening can alter the activation patterns seen in these networks will be convincing evidence that the approach can modulate brain function.

## A129 Acoustic characterization of two pediatric skull phantoms

### Elodie Constanciel Colas^1^, Adrian Wydra^2^, Adam Waspe^3^, Thomas Looi^1^, Roman Maev^2^, Samuel Pichardo^4^, James Drake^3^

#### ^1^Centre for Image Guided Innovation and Therapeutic Intervention, Toronto, Ontario, Canada; ^2^The Institute for Diagnostic Imaging Research, Windsor, Ontario, Canada; ^3^Hospital for Sick Children, Toronto, Ontario, Canada; ^4^Thunder Bay Regional Research Institute, Thunder Bay, Ontario, Canada

**Objectives**

Clinical trials for neurological disorders are ongoing using MR guided Focused Ultrasound (MRgFUS) on adult patients. Pediatric patients have a thinner skull and could highly benefit from this new technique. However, the acoustic properties of their skull have not been reported extensively in the literature. Patient growth affects the structure and shape of the skull. Designing an MRgFUS system dedicated to pediatric patients requires a full understanding of the evolution of the skull with the age of the patient. Acoustic characterization of *ex vivo* pediatric skulls is ongoing but specimens are rare and cannot represent the entire population. Creating a library of skull phantoms based on CT or MRI scans could offset the lack of *ex vivo* specimens. The aim of this study was to compare two pediatric skull phantoms to their corresponding *ex vivo* skulls to validate the material properties and the manufacturing process.

**Methods**

A neonate *ex vivo* skull and an 8-year old (8-y.o.) *ex vivo* skull were scanned using a micro-CT scanner and a clinical CT scanner, respectively. Skull phantoms were manufactured, based on these CT datasets, using a bone-mimicking material previously described (Wydra and Maev, 2013), and in turn CT scanned. Variations in the composition of the material were used to mimic purely cortical bone but also mixte cortical and trabecular bone structures. The 2 skulls and their phantoms were acoustically characterized using a clinical MRgFUS system (Philips Sonalleve V1) and a needle hydrophone at 1.2 MHz. Using the 256 elements of the transducer, a mapping of the Time-Of-Flight (ToF) delays after insertion of the skull was obtained. The thickness of the skulls and the phantoms was estimated using the CT datasets. The average speeds of sound (SoS) of the phantoms were compared to the SoS of the real skulls.

**Results**

Visually, phantoms and *ex vivo* skulls were similar. The neonate phantom even featured a fontanelle made of an ultrasound-transparent material. CT dataset comparisons showed a discrepancy between the thickness of the real skulls and the phantoms. The neonate phantom was on average 100 % thicker than the *ex vivo* skull. The 8-y.o. skull phantom was 50 % thicker than the *ex vivo* skull. The ToF delays obtained reflected these differences with an average of -0.4 μs for the neonate phantom vs -0.15 μs for the *ex vivo* skull and an average of -1.1 μs for the 8-y.o. skull phantom vs -0.7 μs for the *ex vivo* skull. The average SoS of the neonate phantom was 2250 m.s-1 vs 2080 m.s-1 for the *ex vivo* skull. The average SoS of the 8-y.o. skull phantom was 2500 m.s-1 vs 2300 m.s-1 for the *ex vivo* skull.

**Conclusions**

The bone-mimicking phantoms are a good alternative to rarely available *ex vivo* pediatric skulls. The thickness of the phantoms will be further adjusted in order to better mimic the real skulls. Future work will focus on the attenuation properties of these phantoms.

## A130 Long-term transgene expression in rat brain after intranasal administration of hGDNF DNA nanoparticles and enhancement by Focused Ultrasound (FUS)

### Amirah Aly^1^, Tao Sun^2^, Yong-Zhi Zhang^3^, Ozge Sesenoglu-Laird^4^, Linas Padegimas^4^, Mark Cooper^4^, Nathan McDannold^2^, Barbara Waszczak^1^

#### ^1^Northeastern University, Boston, Massachusetts, USA; ^2^Brigham and Women’s Hospital, Harvard Medical School, Boston, Massachusetts, USA; ^3^Harvard Medical School, Boston, Massachusetts, USA; ^4^Coprenicus Therapeutics, Inc., Cleveland, Ohio, USA

**Objectives**

The therapeutic potential of glial cell-line derived neurotrophic factor (GDNF) for treating Parkinson’s disease (PD) has been limited thus far by its inability to cross the Blood-Brain Barrier (BBB). We have previously shown that intranasal administration of PEGylated lysine 30-mer (CK30PEG10K) DNA nanoparticles (NPs) encoding hGDNF, developed by Copernicus Therapeutics, Inc., can transfect brain cells *in vivo*, induce transgene expression, and provide neuroprotection of substantia nigra (SN) dopamine neurons in the rat 6-hydroxydopamine model of PD. We have also shown using double-label immunohistochemistry that transgene expression in the rat brain occurs primarily in cells lining the vasculature, most likely pericytes. Although intranasal administration allows large biomolecules to bypass the BBB, it is a low efficiency route and results in widespread distribution with no means of targeting specific brain regions. Here we investigated whether focused ultrasound (FUS) combined with circulating microbubbles can enhance intranasal delivery of pGDNF NPs to the brain.

**Methods**

The first goal of our current study was to assess the duration of transgene expression after intranasal administration of DNA NPs. We used a reporter plasmid (pUGG), which produces an eGFP-GDNF fusion protein. All rats received 90 μg of pUGG NPs, a dose that we previously showed yields significant eGFP expression throughout the rat brain 1 week after intranasal administration. Groups of 8 rats were sacrificed at 1 week, 3 months and 6 months after intranasal dosing. Separate groups received intranasal saline and were sacrificed at the same time points.

Our second goal was to evaluate whether FUS can be combined with intranasal administration of pUGG NPs, as well as the naked plasmid, to yield greater overall delivery, improved tissue penetration, and enrichment of transgene expression in the desired location(s) in brain. To date, we have conducted a pilot study in 3 rats given 180 μg of naked pUGG DNA intranasally. FUS bursts (32 msec bursts at 4 Hz for 100 sec) at 274 kHz were applied during infusion of Optison microbubbles to one brain hemisphere before intranasal delivery, and repeated 1 hour later at a caudal location on the same side after intranasal delivery. The rats were sacrificed 1 week later.

Expression of eGFP was assessed throughout the brain by eGFP-ELISA.

**Results**

In the time course study, ELISA revealed that whole brain eGFP expression (above background in saline-treated controls) was highest at one week, and persisted at ~30 % of maximal expression at both the 3 and 6 month time points (p < 0.05 for 1 week *versus* 3 and 6 months). These results provided evidence that intranasal delivery of Copernicus’ pDNA NPs results in significant and prolonged transgene expression in rat brain, with highest levels at 1 week and continued expression over at least 6 months.

Results from the pilot study in rats which received naked plasmid intranasally, in conjunction with FUS and microbubbles, indicated that the amount of transgene expression was increased on the sonicated *versus* the unsonicated side of the brain, and overall whole brain eGFP weighted averages were significantly increased in the sonicated rats. These results suggest that FUS with microbubbles may increase the overall delivery of DNA NPs to the sonicated brain regions.

**Conclusions**

In conclusion, FUS may enable agents with poor capabilities of crossing the BBB, e.g. neurotrophic factors, and viral and non-viral vectors encoding them, to become disease-altering therapies by a non-invasive route of administration. Our ongoing studies are examining the effect of FUS on transgene expression in the sonicated regions, and will assess tissue penetration and cell types transfected after intranasal administration of our DNA NPs.

## A131 The comparison of different FUS parameters mediated therapeutic responses in a subcutaneous model of melanoma

### Seruz Tehrani, Wilson Miller, Craig Slingluff, James Larner, Kumari Andarawewa

#### University of Virginia, Charlottesville, Virginia, USA

**Objectives**

Melanoma has a 5-year survival rate of <10 %, primarily due to therapeutic resistance and immune tolerance. Durable responses to therapy of metastatic melanoma are enhanced when the anti-tumor immune response is activated. FUS is an emerging non-invasive treatment modality for localized treatment of cancers. Damaged tumor cells have been shown to release endogenous danger signals, which could stimulate an immune response, implying that the patient’s dying cancer cells serve as a therapeutic vaccine that stimulates an anti-tumor immune response. Specifically, we hypothesize that FUS will augment tumor antigen release and danger signals to mediate rejection of both FUS treated and untreated tumors. We believe that this approach will increase the percentage of patients who will respond to immunotherapies by stimulating a melanoma-specific immune response. However the optimum parameters to simulate this response is mostly unknown. Therefore we conducted a preliminary experiment to compare different FUS parameters with biological responses.

**Methods**

Studies using C57BL6 mice inoculated subcutaneously with B16 melanoma cells were approved by the institutional animal care and use committee. When tumors became palpable, mice received FUS treatments (two sites per tumor) using a 1.14 MHz transducer (FUS Instruments, Canada). FUS treatment was applied either in pulsed (group-1: 44 W, 10 ms pulse duration, 1 s intervals, 60s total duration at 7 MPa or group-2: 44 W, 14 ms pulse duration, 0.5 s intervals, 120 s total duration at 7 MPa or in continuous mode (group-3: 15 W, 10s, 4 MPa or group-4: 44 W, 3.5 s, 7 MPa). The temperature maps were generated for treated tumors. Following FUS treatment, necrosis, and apoptosis were measured. Standard H&E staining was performed to evaluate histological changes that may have occurred as a result of the FUS exposures. The detection of apoptotic cells was performed using staining for Caspase-3.

**Results**

We observed that the temperature rise for tumors treated in continuous mode (group-3) were in the range of 55-65 °C and temperature of pulsed tumors (group-1 and 2) were approximately ~40 °C. We were unable to measure the temperature of group-4, due to the tumor movement as result of FUS treatment. We observed increase in both necrosis and apoptosis with FUS treatment (group-1, 2 and 3) when compared to untreated tumors.

**Conclusions**

Our data shows that FUS parameter used in the group-4 cannot be adopted due to technical difficulties that arise due to movement of the tumor. However our data shows that group-3 conditions give rise to increase in temperature when compared groups-1 and 2. Additional mouse studies are currently underway to confirm these results and to characterize and compare the immune response elicited in the group-1, 2 and 3.

## A132 Evaluation of bone ablation size as a function of sonication time during MRgFUS

### Matthew Bucknor, Eugene Ozhinsky, Rutwik Shah, Roland Krug, Viola Rieke

#### University of California San Francisco, San Francisco, California, USA

**Objectives**

One challenge in MRgFUS ablation of bone tumors is extending the depth of the ablation beyond the cortical surface, which rapidly attenuates sound. Anecdotally, providers will lower ultrasound frequency or increase sonication duration, in order to achieve better penetration. The purpose of this study was to evaluate the effect of longer *versus* shorter sonication durations on the size of the post-ablation appearance in a swine model of MR guided Focused Ultrasound ablation of bone, for a given total energy.

**Methods**

Experimental procedures received approval from the institutional committee on animal research. MRgFUS was used to create two ablation foci (distal and proximal) in the left femoral diaphysis of 6 pigs. The spacing of sonications was equivalent between the two foci. Both targets were subjected to six sonications with 400 J of energy each: the distal targets were dosed with 20 W for 20 seconds (standard time) and the proximal targets were dosed with 10 W for 40 seconds (long duration). The hypoenehanced ablation zone was then measured on post-contrast MRI sequences in three dimensions.

**Results**

w?>MRgFUS created focal hypoenhanced lesions at the distal and proximal targets. Interestingly, the use of the conventional 20 second duration sonication resulted in the largest depth of the transverse intramedullary hypoenhanced zone and the craniocaudal dimension of the ablations, which measured up to an average of 7.3 mm and 26.7 mm, respectively. By comparison, the mean ablation measurements for the 40 second long duration sonication group were 4.5 mm and 21.0 mm: these differences reached statistical significance (paired t-test, p = 0.026 and 0.006). There was no significant difference in the anteroposterior measurements between the two groups.

**Conclusions**

While different techniques can and should be used to maximize the size of the ablation zone in MRgFUS of bone lesions, these results suggest that increasing the sonication duration, while concomitantly decreasing the acoustic power to maintain a given total energy, is not an effective technique and may be counterproductive. These results can be used to achieve more complete MRgFUS ablations of bone lesions.

## A133 Image-guided targeted doxorubicin delivery with hyperthermia to optimize loco-regional control in breast cancer; the i-GO feasibility study

### Roel Deckers^1^, Sabine Linn^1^, Britt Suelmann^1^, Manon Braat^1^, Arjen Witkamp^1^, Paul Vaessen^1^, Paul van Diest^1^, Lambertus W. Bartels^1^, Clemens Bos^1^, Maurice van den Bosch^1^, Nicolas Borys^2^, Gert Storm^3^, Elsken Van der Wall^1^, Chrit Moonen^1^

#### ^1^University Medical Center – Utrecht, Utrecht, Netherlands; ^2^Celsion Corporation, Lawrenceville, New Jersey, USA; ^3^Utrecht University, Utrecht, Netherlands

**Objectives**

Since advances in systemic treatment have led to improved overall survival in patients with metastatic breast cancer obtaining optimal local control of the primary tumor has become more important. Furthermore, various studies have suggested that as a consequence of effective locoregional treatment, survival can further be improved.

Doxorubicin is an effective drug in breast cancer treatment and is included in most chemotherapy regimens today. At present, the achievable dose of doxorubicin to the primary tumor is limited by its systemic effects.

Based on pre-clinical investigations we hypothesize that, doxorubicin loaded thermos-sensitive liposomes (ThermoDox) in combination with MR-HIFU induced local hyperthermia can be safely substituted for free doxorubicin in the AC regimen for treating metastatic breast cancer without compromising systemic toxicity and efficacy. Here, we present the design of the i-GO study that aims to investigate the safety and tolerability of this combination.

**Methods**

Patients with stage IV, HER2-negative breast cancer at diagnosis, who have not received any breast cancer therapy, will be included. For each cycle, six in total, patients will receive a combination of ThermoDox and HIFU-induced hyperthermia followed by cyclophosphamide. All MR-HIFU experiments will be performed on a dedicated breast MR-HIFU system (Philips Healthcare, Vantaa, Finland) integrated with a clinical 1.5 T MRI scanner (Achieva, Philips Healthcare, Best, The Netherlands) [6, 7]. Four to six weeks after finishing the last cycle of chemotherapy the patients will undergo surgery. From here on subjects receive standard of care treatment for metastatic breast cancer. The primary endpoints of this study are safety and tolerability. The secondary objectives are to assess the pathologic response of the primary tumor and the clinical objective response of metastases and primary tumor.

**Results**

We anticipate increased shrinkage of the primary tumor when free doxorubicin is substituted by ThermoDox combined with local HIFU-induced hyperthermia, because this approach leads to a higher drug deposition in tumor tissue. At the same time, we expect that the control of distant disease is maintained, because the systemic effect of ThermoDox is similar to free doxorubicin.

**Conclusions**

Establishing whether ThermoDox combined with local HIFU-induced hyperthermia is safe and tolerable, is the first step towards potential future replacement of surgery in a selected group of high-risk, primary breast cancer patients.

## A134 Retrospective study of patient suitability for MR-HIFU liver therapy

### Navid Farr^1^, Moez Alnazeer^1^, Pavel Yarmolenko^2^, Prateek Katti^1^, Ari Partanen^3^, Avinash Eranki^4^, Peter Kim^4^, Bradford Wood^1^

#### ^1^National Institutes of Health, Bethesda, Maryland, USA; ^2^The Sheikh Zayed Institute for Pediatric Surgical Innovation, Washington, DC, USA; ^3^Philips, Bethesda, Maryland, USA; ^4^Children’s National Health System, Washington, DC, USA

**Objectives**

Liver cancer treatments range from radical surgery to minimally-invasive treatments like radiofrequency ablation. Severe side effects of liver surgery limit its use to possibly curable patients, while inadequate precision and monitoring of image-guided minimally-invasive methods limit their effectiveness. These methods also carry the risks of ionizing radiation, infection, and inadvertent off-target treatment. Shortcomings of current therapies may be addressed with MR-HIFU, which permits exacting, non-invasive tumor ablation without ionizing radiation. Following recent advancements in organ motion tracking and treatment approaches to MR-HIFU treatment of highly perfused organs, a need exists to define eligibility criteria for liver MR-HIFU. However, patient suitability for liver MR-HIFU is currently not well defined. Factors including the degree of underlying liver dysfunction, tumor extent, and anatomic accessibility may inform MR-HIFU inclusion and exclusion criteria. This study seeks to identify suitable candidates for MR-HIFU and its possible combination therapies based solely on HIFU beam access to tumor.

**Methods**

We assess the theoretical suitability of patients for MR-HIFU by retrospectively evaluating the medical and treatment-planning records of patients with liver tumor. All patients were enrolled at the Center for Interventional Oncology at the Clinical Center in National Institutes of Health between January 2015 and January 2016 and have had known, non-MR-HIFU treatments. The institutional review board approved this retrospective chart review study and informed consent was waived. All medical records were anonymized and de-identified. A blinded clinician segmented computed tomography (CT) scans with thin slices using 3DSlicer. Segmented volumes were exported to MatLab where a semi-automated algorithm evaluated whether the tumor(s) would be accessible by an MR-HIFU platform. Treatment feasibility was evaluated on both a clinical tabletop HIFU system (Sonalleve V2, Philips, Vantaa, Finland) and a freely positionable extracorporeal mock HIFU transducer. Patients were categorized into the following treatment groups: 1) full ablation of the tumor; 2) combination of HIFU and trans-arterial chemoembolization (TACE); 3) full or partial sonication of tumor for immune response evaluation; and 4) HIFU mediated targeted drug delivery. The categorization of patients into these therapeutic arms was based upon size, volume, number, and distribution of disease.

**Results**

Data for 250 patients with liver mass were analyzed, and suitability for HIFU therapy evaluated. We aim to report patient and tumor characteristics, percentage of patients eligible for MR-HIFU, and the distribution of non-HIFU treatments the patients received. Data analysis is pending.

**Conclusions**

MR-HIFU may improve existing treatment algorithms by offering patients a precise, non-invasive choice in both curative and palliative settings. Randomized control studies are required to evaluate the true potential of HIFU as a therapy modality. To better inform patient enrollment strategies, our retrospective study aims to define characteristics of a suitable candidate for MR-HIFU liver therapy.

## A135 Experimental assessment of phase aberration in MRgFUS breast treatments

### Alexis Farrer, Scott Almquist, Christopher Dillon, Dennis Parker, Douglas Christensen, Allison Payne

#### University of Utah, Salt Lake City, Utah, USA

**Objectives**

MR-guided Focused Ultrasound (MRgFUS) has the potential to be used as a non-invasive breast cancer therapy. Clinical feasibility has been demonstrated in multiple studies and hardware development has progressed in addressing the challenges of treating the breast. It has been demonstrated in a simulation study that phase aberration caused by the patient-specific heterogeneous anatomy of the breast may decrease the efficiency of MRgFUS breast treatments by causing unwanted heating when using a small phased-array transducer (256-element, 1-MHz, 14.4 x 9.8-cm aperture). The purpose of this work is to develop tissue-mimicking heterogeneous breast phantoms in which the simulated effects of phase aberration and phase aberration correction techniques can be experimentally verified.

**Methods**

Phantom: A heterogeneous phantom was constructed that mimics the breast anatomy and adequately replicates the acoustic properties of breast fibroglandular and fat tissue. The phantom consists of 250-bloom gelatin with a 1 to 1 mixture of water and evaporated milk. Six latex balloons (diameter = 1.5-2.0 cm) filled with canola oil were dispersed throughout the phantom. The phantom was mounted in our breast MRgFUS system and 3D MR images were obtained to create a numerical model of the phantom (3D 2-point Dixon). MRgFUS experiment: To assess the effect of phase aberrations, two MRgFUS heating schemes were tested: a single-point sonication (23 W, 30 s) and a 9-point raster-scanned trajectory (2-mm spacing, 32 W, 20 s/point, no cooling). Both heating schemes were performed with occlusions in the near field. To evaluate different acoustic windows, these sonications were repeated at two different transducer locations. MR thermometry was used for real-time monitoring (3D seg-EPI GRE). Simulations: Using the hybrid angular spectrum method applied to the generated numerical model, simulated pressure patterns were obtained both with and without applying a phase correction technique. Temperature response was modeled with a finite-difference implementation of the Pennes bioheat equation.

**Results**

**Fig. 141 (abstract A122).**
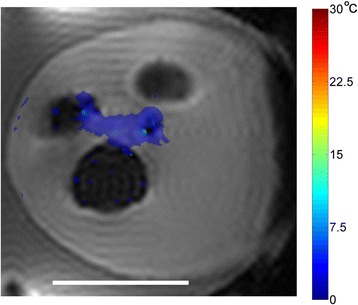
A coronal oblique slice of the heterogeneous aberrating breast phantom is shown with the experimental MR temperature map (PRF-shift method) overlaid on a T1-weighted image. The white scale bar represents 5 cm

**Fig. 142 (abstract A135).**
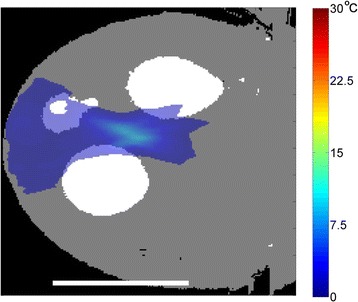
The simulation on the segmented numerical model obtained from the same aberrating phantom as shown in Fig. 141 is shown with temperature maps overlaid for uncorrected phases. The white scale bar represents 5 cm

**Fig. 143 (abstract A135).**
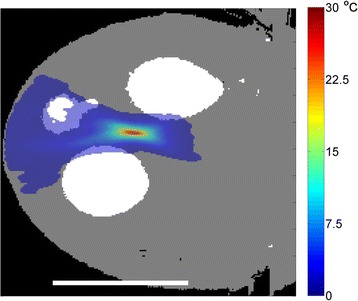
The simulation on the segmented numerical model obtained from the same aberrating phantom as shown in Fig. 141 is shown with temperature maps overlaid with phase correction applied. The white scale bar represents 5 cm

Figures 141, 142 and 143 show the comparison of experimental and simulated results for the single sonication case. In all cases the temperature map is overlaid on the phantom’s magnitude image or the numerical model, and the transducer (not shown) is located to the left for all images. The experimental temperature response is shown in an oblique slice in Fig. 141. The focal spot is clearly aberrated and resulted in low temperatures (peak of 9.5 °C) at the desired focus. Figures 142 and 143 show the simulated temperature maps for a single sonication at the same location without and with phase aberration correction, respectively. The simulation of the focal spot after phase correction is applied shows a dramatic improvement in energy delivery. The aberrated focal spot resulted in a much lower temperature rise (scaled to match experimental) when compared to the focal spot with phase correction applied (peak of 27.6 °C). Also, the peak temperature was 3 mm in front of the desired focal location for the single sonication without phase correction and at the desired location with the correction applied. A similar trend for the peak temperatures and focal location accuracy was seen for the 9-point trajectory.

**Conclusions**

This work presents a heterogeneous anatomy-mimicking breast phantom that produced aberrations both experimentally and in simulation. The correction of these aberrations in simulation demonstrated an increased temperature rise and more accurate focal location. Work is ongoing to apply the phase correction technique experimentally. Additional heterogeneous aberrating breast phantoms are under construction to further validate this technique.

## A136 Patient preparation influence on respiratory compensated PRFS based MR thermometry performance in the pancreas

### Cyril Ferrer, Lambertus W. Bartels, Baudouin Denis de Senneville, Marijn van Stralen, Chrit Moonen, Clemens Bos

#### University Medical Center – Utrecht, Utrecht, Netherlands

**Objectives**

Magnetic Resonance Imaging-guided High Intensity Focused Ultrasound (MR-HIFU) has recently been suggested as an alternative treatment modality, for pancreatic cancer patients in cases where surgery is not an option. MR-guidance allows to monitor temperature for feedback control, however using proton resonance frequency shift (PRFS) based thermometry is challenging, because of unpredictable motion (peristaltic) and the presence of air in the digestive tract near the head of the pancreas. This can lead to changes in the magnetic volume susceptibility distribution resulting in artifacts in the temperature maps. Solutions have been proposed to this end, and even if it is tempting to transfer methods from one organ to another it is not necessarily straightforward.

In this study, we propose an approach aimed at reducing susceptibility heterogeneity by filling the stomach and duodenum with fluid, and evaluate its effect on PRFS based temperature mapping using different known methods for respiratory compensation.

**Methods**

Experiments were performed on a 1.5-T MRI scanner (Achieva, Philips Healthcare, The Netherlands) equipped with a clinical MR-HIFU system. Experiments were executed twice on healthy volunteers (n = 7), first, without any special preparation. Then during a different exam, volunteers were asked to ingest 2 ´ 500 mL of fluid with favorable relaxation times (pineapple juice) to fill the tract, 5 hours and 2 to 5 minutes prior to scan, respectively. A breathhold 3D fat suppressed T1-weighted scan was acquired for planning. For temperature mapping, a dynamic gradient echo series consisting of 3 coronal slices covering the pancreas was acquired. Scan parameters: TR = 100 ms, TE = 19 ms, FA = 21, reconstructed voxel size = 2.5x2.5x8 mm3, dynamic scan time = 400 ms, 500 dynamics.

Statistical analysis: Datasets were analyzed offline using three techniques, viz. gating2, multibaseline4, and referenceless6. The temperature standard deviation (TSD) over time was calculated voxel-by-voxel. As reference the whole pancreas was semi automatically segmented on the T1 image and used as ROI for statistical analysis. For each technique the volume of pancreas under the threshold value of 2 °C precision was compared to the total pancreas volume in our images leading to a percentage of coverage at 2 °C.

**Results**

**Fig. 144 (abstract A136).**
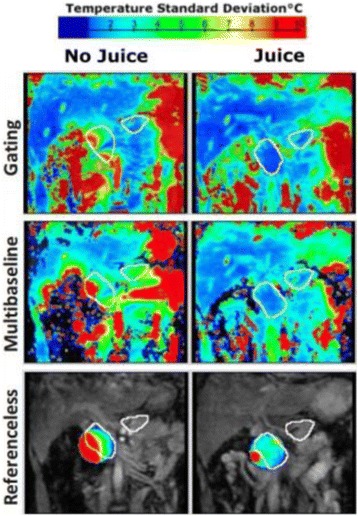
Standard Deviation maps computed with our different respiratory compensated methods, overlayed on anatomical image of the same volunteer (contour of the pancreas is outlined in white, the head and a part of the body is always shown). Volunteers were imaged without or after intake of 2 times 500 mL of pineapple juice

Figure 144 shows an example of the juice filling effect in one of the volunteer. Without juice the TSD varied from 2 to 10 °C, within our three methods. After juice intake, the TSD range went down to values between 1 to 2 °C and the spatial distribution became clearly more homogeneous.

**Fig. 145 (abstract A136).**
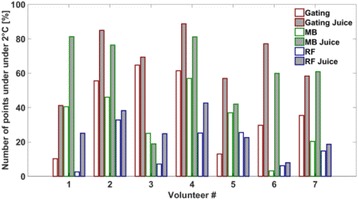
Percentage of coverage of the pancreas at +/-2 °C precision for our different respiratory compensated and filling methods. Preparation without liquid (white bars) is compared to preparation with juice (grey bars). Respectively borders of each bar graph represent different respiratory compensation strategies, in red gating, green multibaseline and blue referenceless

In Figure 145, preparation without liquid is compared to preparation with juice, for every technique the coverage of the pancreas with a precision under 2 °C is increased by 25 % in average. It allows to reach values, up to 80 % with multibaseline and gating.

**Conclusions**

In all volunteers, oral ingestion of pineapple juice for patient preparation reduced the range of values in the TSD leading to an increase of the precision coverage in the pancreas. Filling the digestive tract in this way could be an attractive solution for more precise temperature monitoring during MR-HIFU therapy in the pancreas.

## A137 Quantitative magnetic resonance imaging of ultrasound induced transient shear waves

### Yu Liu, Jingfei Liu, Brett Fite, Josquin Foiret, J. Kent Leach, Katherine Ferrara

#### University of California Davis, Davis, California, USA

**Objectives**

Tissue mechanical properties are altered both in disease states and following ablative, or other, therapy. Changes in mechanical tissue properties are manifested in changes to the speed of a shear wave propagating through the tissue. Transient shear waves can be induced in tissue via application of short bursts of focused ultrasound (US). Imaging the propagating shear wave with MR confers the benefits of that modality’s excellent soft tissue contrast, 3D visualization, and the ability to assess tissue immediately following US ablation. Here, we quantify tissue elasticity using constructive multi-pulse transmission under MR and US guidance and validate with mechanical testing.

**Methods**

**Fig. 146 (abstract A137).**
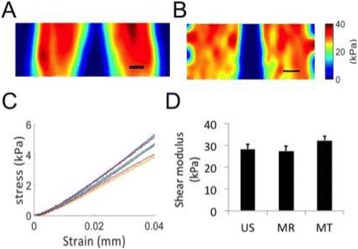
Elasticity maps from **a** MR and **b** ultrasound. **c** Strain-stress results based on mechanical testing. **e** The estimated shear moduli were 28.2 ± 2.3, 27.3 ± 2.3 and 32.3 ± 1.8 kPa in US, MR and mechanical testing, respectively

MR measurement of transient shear wave (SW) propagation was performed using a 7 T MRI (Bruker Biospin, Germany) and a 3 MHz 16-element annular array (IMASONIC SAS, France, 48 mm diameter, 35 mm radius of curvature, 0.5 × 0.5 × 2 mm3 focal volume at -6 dB), controlled by an embedded MR-compatible 2D positioning and US system (Image-Guided Therapy, France). Three foci (3 MHz, 450 ms pulses with 4.5 mm spacing between transmit foci, 12.5 MPa PNP) were sequentially excited along the beam axis creating a planar SW which was imaged with MRI (Fig. 146a, TR/TE/FA = 500 ms/26.1 ms/180°). A series of images was acquired with different propagation times, varying from 0.45 ms to 4.05 ms and the shear modulus was estimated using the time-of-flight (TOF) method. The US-based SW measurement was performed with a Vantage 256 (Verasonics, WA, USA) and a Phillips L7-4 probe. To generate the SW, 4.3 MHz, 10 MPa PNP, 100 μs pulses were transmitted with 5 mm spacing between transmit foci. US images were obtained with a 5.2 MHz center frequency and 10 kHz PRF and the local inversion method (LIM) was used to estimate the shear modulus. Phantom cylinders (9.7 mm in diameter and 8.5 mm

**Results**

The propagating SW was visualized with both MRI (Fig. 146a) and US (Fig. 146b) and quantitative elasticity maps (Fig. 146c) were generated. Using multi-pulse transmission, the shear modulus in a 1.5 % agar and 1.5 % gelatin phantom was estimated as 27.3 ± 2.3 kPa with MR and 28.2 ± 2.3 kPa with US, which agreed with 32.3 ± 1.8 kPa yielded by compressive testing (Fif. 1D).

**Conclusions**

In conclusion, the shear modulus in tissue-mimicking phantoms was accurately estimated using the combination of an MR shear imaging sequence and multi-pulse transmission.

## A138 In vivo biodistribution studies of drug-loaded nanodroplets for prostate cancer management using ultrasound-mediated drug delivery under MR-guidance

### Roohi Gupta, Dusica Cvetkovic, Charlie Ma, Lili Chen

#### Fox Chase Cancer Center, Philadelphia, Pennsylvania, USA

**Objectives**

Our ultimate goal to initiate the present study is to develop a novel approach for treating prostate cancer with improved efficiency while reducing normal tissue toxicities. In this treatment approach, the drug is delivered into the tumor via encapsulation inside an ultrasound-sensitive, drug delivery system (nanodroplets). Once the system reaches tumor, MR-guided pulsed High Intensity Focused Ultrasound (MRg-pHIFU) is applied to the tumor to help activate the release of the drug from nanodroplets inside the tumor, thus increasing the bioavailability of the drug.

The purpose of the present work is to study the biodistribution of paclitaxel-loaded nanodroplets *in vivo* in nude mice. We consider our present study to be of high impact because it tries to answer specifically for the first time an important question of appropriate timing to apply tumor-targeted focused-ultrasound for activating release of drug from these drug carriers for effective prostate cancer treatment.

**Methods**

Poly {ethylene oxide}-co-poly {D, L-lactide} (PDLA) nanodroplets loaded with fluorescently labeled-paclitaxel (F-PTX) were synthesized using solid dispersion technique. Human prostate cancer, LNCaP cells were implanted orthotopically in prostates of male nude mice. Tumor-bearing mice (n = 3) were injected with 0.1 % F-PTX, 2 % PDLA nanodroplets using tail vein. At chosen time points (30 min; 2 h; 4 h; 6 h; 12 h; 24 h) animals were anesthetized, blood was collected by eye bleed, animals were sacrificed, tumor and vital organs (liver, spleen, kidney lung and heart) were excised, cut and weighed. Then the organ was homogenized, incubated in lysis buffer and centrifuged. The lysates were read using fluorescence spectrophotometer (Excitation- 496 nm, Emission– 524 nm). Fluorescence readings were compared with values from the standard calibration curve. Tumor-bearing mice (n = 3) were also injected with 0.1 % F-PTX solution (fluorescent drug in free form).

**Results**

Quantitative analysis showed 20 % of the injected drug-loaded nanodroplets in the tumor, 30 min after systemic injection; which increased to 30 % after 2 h; 35 % after 4 h; then decreased to 27 % after 6 h; 11 % after 12 h and 5 % after 24 h. When drug in free form was injected, drug accumulation in the tumor was 7 % within 30mnt; 8 % within 2 h; 10 % within 4 h; 9 % within 6 h, 3 % within 12 h and 2 % within 24 h. Liver showed major drug accumulation for drug-loaded nanodroplets while heart and lung showed less. For drug in free form, liver and spleen showed maximum drug concentrations while heart showed concentrations similar to that in tumor.

**Conclusions**

Treating prostate cancer with MRg-pHIFU mediated drug-delivery using ultrasound-responsive, drug-delivery vehicle (nanodroplets) is innovative and aims at minimizing toxic effects to healthy tissues, as caused by chemotherapeutics drugs and maximizing better treatment efficacy. Present bio-distribution indicates that the optimum time for applying focused ultrasound is after 4 h of systemically injecting drug-loaded nanodroplets. Using this optimum time point, *In vivo* experiments in nude mice are being carried out to test the efficacy of combined MRg-pHIFU exposures and chemotherapeutic agents encapsulated nanodroplets for prostate cancer therapy.

## A139 From research to clinics: an example of clinical translation of MR guided FUS treatment of the liver

### Sabrina Haase^1^, Stephan Zidowitz^1^, Andreas Melzer^2^, Tobias Preusser^1,3^

#### ^1^Fraunhofer MEVIS, Bremen, Germany; ^2^University of Dundee, Dundee, UK; ^3^Jacobs University Bremen, Bremen, Germany

**Objectives**

There are many groups around the world working on MR guided FUS research topics to enhance the treatment for patients and to advance the technologies. The main goals of the research is to provide faster and more accurate algorithms and tools while ensuring the clinical safety, efficiency and effectiveness. In the European funded TRANS-FUSIMO project (www.trans-fusimo.eu), the consortium is currently developing a quality assured treatment system for MR guided Focused Ultrasound in moving abdominal organs which will be validated and pre-clinically tested. The target of the project is to enable a clinical study using the developed technologies. Here we will discuss the quality assurance that is needed for translation of a research tool towards a clinical study.

**Methods**

If newly developed features or technologies from research shall be translated to clinical use, there are many obstacles on the way. In particular if the research system shall be used in a clinical study it has to comply with high quality standards. It is also possible to perform clinical studies without a CE mark but the medical device must have a quality status that is CE mark ready. The most important norm is 93/42/EEC which is the European medical devices directive. This directive also asks for an underlying quality management system which can be EN ISO 13485. This means that the parties, which are responsible for this medical device have to be qualified according to EN ISO 13485. If a software tool is the critical part of the clinical device, its development and implementation has to conform to IEC 62304, which is the international standard for medical device software. Furthermore, a risk management for the medical device according to ISO 14971 has to be performed. Only if development and validation are conducted according to these standards authorities can approve a clinical investigation of the medical device.

**Results**

In the work presented here, we are translating software, which is able to communicate with an MR device as well as an HIFU device. Both MR device and HIFU system have CE marks or are at least CE mark ready for their intended use, the new research communication software not, still it shall be used in a clinical trial. The software shall have the ability to follow the breathing motion of the patient and steer a focal spot in the liver accordingly. Since this software without CE mark shall be used in a clinical study we must follow the above described quality assurance. In our case the software with its subcomponents is developed according to the EN ISO 13485 norm and also the preclinical *in vitro* and *ex vivo* validation is performed according to this quality management system. Furthermore, since the software acts as the control system of the HIFU device it is a critical part of the system and thus the development and implementation has to conform to IEC 62304. Finally, a risk management for the medical device according to ISO 14971 was created.

**Conclusions**

If a clinical investigation is anticipated, CE mark status is necessary. The example presented in this work shows how to use a non CE marked software tool in conjunction with existing qualified hardware. Also a preclinical validation is mandatory in order to ensure the safety, efficiency and effectiveness for the patients during the treatment.

## A140 Magnetic resonance-guided focused ultrasound in treating painful bone metastasis: preliminary results of a phase IV study

### Hsin-Lun Lee, Fang-Chi Hsu, Chia-Chun Kuo, Shiu-Chen Jeng, Tung-Ho Chen, Nai-Yi Yang, Jeng-Fong Chiou

#### Taipei Medical University Healthcare System, Taipei, Taiwan

**Objectives**

Bone metastasis represents one of the major causes resulting in cancer pain. Traditionally, conventional radiotherapy serves as the standard treatment for painful bone metastasis. Recently, magnetic resonance-guided focused ultrasound (MRgFUS) has been demonstrated as an alternative therapy for pain palliation of bone metastasis. Therefore, this phase IV study was conducted to evaluate the long-term safety and efficacy of MRgFUS treatment for patients with painful bone metastasis.

**Methods**

Current ongoing single-institute phase IV study has enrolled 20 patients at Taipei Cancer Center, Taipei Medical University in Taiwan since Nov 15th, 2014. The MRgFUS is delivered by ExAblate 2000 system (InSightec Ltd., Israel) which integrates a high-intensity focused ultrasound compartment for hyperthermia treatment with a magnetic resonance imaging scanner for real-time position and temperature monitoring. The treatment response is evaluated by the interval change of numerical rating scale (NRS) before and after treatment. Pain relief is defined as a reduction of 2 points or more on NRS pain scoring system without increasing analgesics use. Patient’s quality of life is assessed by the Brief Pain Inventory (BPI-QoL) score. The adverse event is also documented.

**Results**

The median age of the 20 treated patients (12 male and 8 female) was 62 years old (range, 40–83). Each patient underwent MRgFUS treatment for single painful bone metastasis. Sixteen targeted lesions were located in the pelvic bone, whereas 3 at rib cage and 1 at scapula. At a median follow-up duration of six months, none of the patients experienced treatment related adverse events above grade 2 according to Common Terminology Criteria for Adverse Events version 4.0. Significant improvement of NRS pain score and BPI-QoL score is observed after treatment.

**Conclusions**

The initial results of current study demonstrate the safety and efficacy of MRgFUS in treating painful bone metastasis. MRgFUS has the potential to serve as a first-line treatment in selective patients with painful bone metastasis.

## A141 Heating position accuracy assurance of a clinical HIFU treatment for bone metastasis via MR-guided Focused Ultrasound system

### Shiu-Chen Jeng, Yi-tzu Kao, Chia-Hsin Pan, Jing-Fu Wu, Tung-Ho Chen, Fang-Chi Hsu, Hsin-Lun Lee, Jeng-Fong Chiou

#### Taipei Medical University Healthcare System, Taipei, Taiwan

**Objectives**

Utilizing magnetic Resonance-guided Focused Ultrasound Surgery (MRgFUS) to treat metastatic bone tumor is an advance therapy for palliative pain control. MRgFUS system performs High Intensity Focused Ultrasound (HIFU) with real-time MRI monitoring and results in local high temperature hyperthermia. In this study, we applied quality assurance concepts to endorse accuracy of thermal dose and position stability. Furthermore, this research tried to share experience of calibration protocol, and to provide permissible criteria according to cases treated so far. Moreover, the goal is to establish standard operating procedure of examining accuracy of focal spots, in order to provide patients a more qualified treatment.

**Methods**

In this research, we measured focusing error distances between focal spots and real heating spots from 31 cases in Taipei Medical University Hospital to identify the effectiveness of Daily Quality Assurance (DQA) for preventing focusing error in MRgFUS. MRgFUS applies Magnetic Resonance Imaging (MRI) to locate metastatic bone tumors, uses heating technique of focused ultrasound to convey ultrasound energy to painful bone area. The energy would heat the area up to 60 degrees Celsius and ablate nerve fibers on the surface of bone to achieve the purpose of palliative pain control. Advantages of this treatment include non-invasive, non-irradiative, and long-term pain relief in single treatment.

**Results**

Comparing the differences before and after quality assurance, focusing errors in DQA group were observed consistent diminutions, which reached statistical significance (p-value < 0.01). Nevertheless, DQA procedures also benefited heating accuracy in practice. The 95 % confident interval of accuracy were reported from 1.73 ± 0.43 mm (Pre-DQA groups) to 0.43 ± 0.12 mm (DQA groups).

**Conclusions**

In summary, a comprehensive DQA standard guarantees treatment accuracy and patient safety. As a suitable calibration procedure, DQA was evaluated its advantages to reduce focusing errors and unexpected heating spots. Therefore, DQA procedures were highly recommended to adjust each distance between focal spot and real heating spot within 1 mm before treatment.

## A142 Simulation and evaluation of enhanced thermal necrosis distribution via high intensity focused ultrasound hyperthermia therapy

### Fang-Chi Hsu, Yi-Chieh Tsai, Hsin-Lun Lee, Jeng-Fong Chiou

#### Taipei Medical University Healthcare System, Taipei, Taiwan

**Objectives**

High intensity focused ultrasound (HIFU) has been reported for its non-invasive heating capability in tumour treatment. As an advance application, magnetic Resonance guided focused ultrasound (MRgFUS) system performs High Intensity Focused Ultrasound (HIFU) with real-time MRI monitoring and results in local high temperature hyperthermia. However, despite correct delivery of thermal dose to treatment area within a short period, to protect nearby critical organs real time temperature should also be monitored. Since local temperature over 56 °C heat shock may lead to thermal tissue necrosis. In this study, gold based micro particles were designed as an enhanced for ultrasound energy absorption in tumor lesions. Through our thermal dose simulation, we found that intra-artery micro enhancer is able to improve thermal dose response during routine HIFU treatment. Furthermore, we tried to share experience of thermal necrosis, and provided a feasible strategy to establish novel treatment.

**Methods**

ExAblate 2000 system (Insightec, Israel) installed in Taipei Medical University and was used to treat bone metastasis for severe pain patients. This system contented a 1.5 T GE magnetic resonance system and a phase array transducer with 208 independent HIFU sonication elements. We simultaneously used an insulated thermocouple wire (Thermoway co., Taiwan) to detect true temperature and compared with PRF temperature. Based on this HIFU system, SAS human oral cancer cell xenograft in nude mouse (National Laboratory Animal Center, NLAC, Taiwan) served as the animal model in this experiment. 6 weeks old nude mice were injected SAS cancer cells in both hind legs and formed about 1 cm tumor. Micro particles conjugated polyethylene glycol (PEG) were inject via intraportal injection and conducted higher affinity with tumor. The ablation target was located in the center of pathology tumor volume (PTV) and sonicated by 300 J energy. Statistical analysis distinguished significant differences for various dose of particles and PRF temperature. Moreover, multiple regression and Matlab models validated correlation of dose bias.

**Results**

It is observed that micro particles promote both MR image resolution and focused ultrasound absorption in target soft tissue. As was shown by man MR signal, that photon intensity (PD) calculated within pathology tumor volume (PTV) in particle injected group (PD = 71.2) was higher than that of sham group (PD = 47.7) and blank group (PD = 67.8).

After HIFU sonication, via implementation of PRF algorithm, temperature distribution at target treatment area was observed to increase form 58.1 ± 3.6 °C (control group) to 63.5 ± 6.3 °C (particle injected group), with statistical significance from Wilcoxon rank-sum test (p = 0.03) between control group and particle group. In addition, from imagine evaluation, post sonicated tissue also shown a dark area in the center of tumor which indicates thermal necrosis of ablation, implying thermal damages that block blood circulation in tumor vessel.

**Conclusions**

In summary, a comprehensive simulation has demonstrated that enhanced tumor necrosis in local lesions generated benefit effects for deep tumor controlling. Nevertheless, we also discovered that micro enhancer is able to improve thermal energy absorption during routine HIFU treatment. This finding indicated that enhanced thermal dose distribution within solid tumor is feasible for first line tumor treatment.

## A143 Quantitative T1 mapping of breast cancer xenografts during HIFU ablation

### Sara Johnson, Dennis Parker, Allison Payne

#### University of Utah, Salt Lake City, Utah, USA

**Objectives**

A current interest in the field of clinical MR-guided HIFU tumor treatments is the ability to acutely assess the extent of tumor necrosis. Previous studies have investigated MR-derived multi-parametrics such as the apparent diffusion coefficient and contrast-enhanced T1 and T2 maps as indicators of cell death within minutes of ablation. In the field of radio-frequency ablation, investigators have used the non-contrast T1 mapping technique, modified look-locker inversion recovery (MOLLI) to predict areas of complete thermal ablation in cardiac tissue. The following study investigates the use of this quantitative T1 imaging technique as a single-parametric predictor of thermal dose achieved in human breast cancer tumors injected in mice.

**Methods**

T1 maps were acquired using the MOLLI method pre- and post- partial thermal HIFU ablation of human breast cancer tumors grown in NOD-SCID mice. Cell suspensions (1 x 106 cells) of one of two human-derived triple negative breast cancer cell lines, HCI002 and, were surgically injected into the mammary fat pads of 16 female NOD-SCID mice (n = 8/cell line), and grown to an average volume of 1609 mm2 or 558 mm2 (HCI002, HCI019, respectively). After localization, a single slice pre-ablation T1 map was acquired (MOLLI). For HIFU treatment, four to nine ablation points (8.3-14.9 W, 30 s) were applied to the center region of each tumor with a small animal MR-compatible HIFU system (Image Guided Therapy, f = 3.0 MHz, FWHM = 1x3 mm), while temperature rise was measured with MR temperature imaging (segmented EPI GRE). Finally a post-ablation T1 map was acquired post-ablation at 20 or 120 s post-ablation (HCI002, HCI019, respectively). Thermal dose of the total treatment was calculated using the CEM@43 °C metric from the MR temperature data, assuming a tumor baseline temperature of 30 °C. ROIs for T1 maps were selected via manual registration with temperature imaging DICOMs (Osirix).

**Results**

**Fig. 147 (abstract A143).**
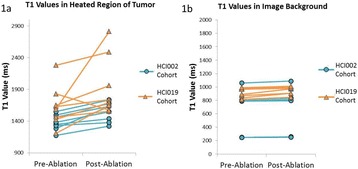
Average T1 values from the heated tumor ROI and a background (control) ROI in a homogenous medium surrounding the tumor (either agar gel or pork loin) pre- and post-ablation (n = 16)

**Fig. 148 (abstract A143).**
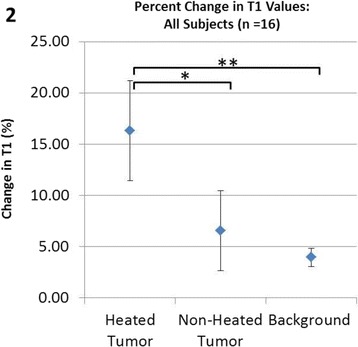
Average percent change in T1 value between pre- and post-ablation in the heated tumor ROI, the non-heated tumor ROI, and the background control ROI, with error bars representing the standard error (N = 16)

**Fig. 149 (abstract A143).**
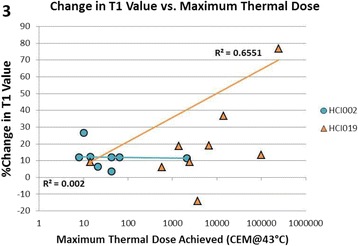
Percent change in average T1 values plotted as a function of maximum thermal dose reached in each animal. R-squared values refer to the linear regression applied to a logarithmic scale of thermal dose (x-axis)

As shown by Fig. 147, there is a consistent trend of increased T1 values in the heated region of the tumors post-ablation. In contrast, the background (control) signal remained relatively constant. Additionally, a small ROI in a region of each tumor not directly heated with HIFU was selected for comparison to the heated regions (Fig. 148). Calculating the percent change in T1 values resulted in P-values of 0.0709 and 0.0136 between the heated and non-heated tumor and control ROIs, respectively. The less significant difference between the heated and non-heated ROIs may be due to the large variance in local tumor changes for both groups, as well as a limited ability to select non-heated regions distant (>3 mm) from the heated regions, since the diffusion of HIFU heating likely contributed to local T1 changes. Finally, the correlation between thermal dose and T1 change is shown (Fig. 149). While there is a correlation between thermal dose and T1 value for the HCI019 cohort, which accumulated higher thermal dose levels and also at a later time post-ablation, the HCI002 cohort, which accumulated a lower thermal dose, shows no correlation.

**Conclusions**

Preliminary results of this breast cancer xenograft study shows that localized changes in quantitative T1 maps can be observed as a result of HIFU ablation within two minutes of treatment completion. Additionally, T1 values were not shown to significantly decrease with increased cooling time, indicating presence of a T1 effect independent of temperature change (data not shown). Finally, the observed T1 change may be correlated with achieved thermal dose, particularly if time after treatment is increased to two minutes or at high thermal doses. Histology for directly determining cell viability in these tumors is ongoing.

## A144 Improve immunity by high intensity focused ultrasound

### Dawei Li, Ye He

#### Shanghai A&S Science Technology Development Co., LTD., Shanghai, China

**Objectives**

HIFU is a method which cures local tumor by thermal effect of ultrasound. But Cancer is systemic disease. Even the local cancer is removed, the rest of the cancer cells will still be transferred to other parts, resulting in the lesion of other parts. How to further improve the treatment effect is still a problem.

**Fig. 150 (abstract A144).**
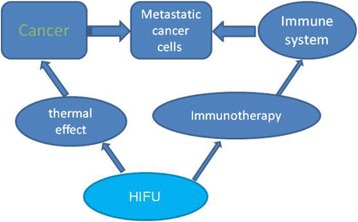
Combine thermal and immune effect to enhance effect of HIFU

This paper discusses how to improve immunity by HIFU, to further kill the metastasis of cancer cells by immune system, and improve the effect of HIFU. Immune system can kill some of the cancer cells. It can prolong the patients’ life and improve the quality of life (Fig. 150.).

HIFU immune function has been mentioned for many years, but there is no specific program. This paper put forward a method to activate the immune function of the human body, and kill tumor cells by immune function. The specific method is that controls the temperature 46 °C for half an hour at the tumor edge.

**Methods**

The method of this paper is based on the production of magnetic resonance imaging guided HIFU. The MRI guided HIFU device use MRI as positioning system and HIFU to treat cancer. This is a new generation of HIFU device, MRI image is much clearer than the ultrasound scanner as the positioning system. At the same time, because the MRI can measure temperature. So the device can measure temperature by MRI and generate heat by HIFU, in order to achieve the treatment method of this program.

The HIFU is focused on the center of the tumor. When temperature is near 46 °C, the dose is reduced to maintain the temperature. This paper uses improved PID arithmetic to control temperature and discusses a lot of experiments *in vitro*, to verify the specific method.

**Results**

The device has achieved the HIFU immunotherapy of control the temperature of tumor edge which is in line with the intended purpose of the design. For irregular margin tumor, the error of the temperature will be increased. For the position of the vessel, the error of temperature will be further increased.

**Conclusions**

The immunotherapy can increase the effect of HIFU. So we should research this method further. Next step is to do clinical trials to verify the efficacy and safety of HIFU immunotherapy.

## A145 First steps of pre-clinical validation of Transfusimo MRgFUS treatment system for liver tumors

### Senay Mihcin^1^, Ioannis Karakitsios^2^, Jan Strehlow^3^, Michael Schwenke^3^, Sabrina Haase^3^, Daniel Demedts^3^, Yoav Levy^4^, Tobias Preusser^3,5^, Andreas Melzer^2^

#### ^1^IMSAT, Dundee, UK; ^2^University of Dundee, Dundee, UK; ^3^Fraunhofer MEVIS, Bremen, Germany; ^4^InSightec Ltd, Tirat Carmel, Israel; ^5^Jacobs University Bremen, Bremen, Germany

**Objectives**

Trans-fusimo Treatment System (TTS) (Fraunhofer MEVIS) aims at applying Magnetic Resonance guided Focused Ultra Sound (MRgFUS) therapy to ablate liver tumours under respiratory motion through the rib cage. Sonicating of a moving target is a very challenging task. TTS controls the Conformal Bone System (CBS) transducer of ( ExAblate 2100, Insightec, Ltd, Tirat Carmel, Israel) for ablation of human tissue while monitoring using the 1.5 T GE MR Scanner. TTS is classified as high risk software as it controls high energy levels emitted by the CBS transducer into human body. For safe clinical use of the system, thorough pre-clinical validation of the software is mandatory. Preclinical validation is divided into two parts; first static case and as next step based on the working evidence of the first part, moving case scenarios. Specs were set with tolerance levels for delivered acoustic power, sonication duration, thermometry and sonicating to a planned position

**Methods**

**Fig. 151 (abstract A145).**
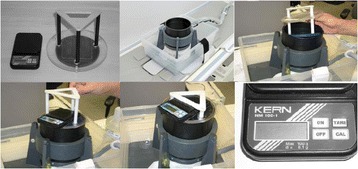
Experimental set up for acoustic power measurements by using weight scale holder assembly for the application of power measurement protocol

**Fig. 152 (abstract A145).**
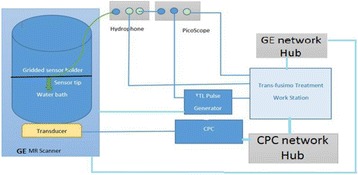
Schematic view of experimental set up for sonication duration

**Fig. 153 (abstract A145).**
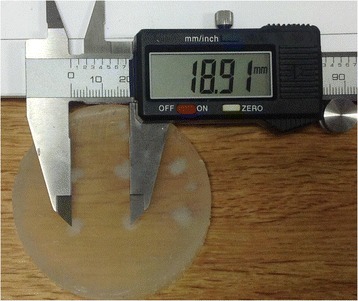
Measurement of sonication deviation actual versus planned (20 mm distance planned)

To validate TTS , novel protocols were designed and feasibility studies were completed for each spec. Acoustic power measurements were completed using the radiation force balance; following the standard IEC 61161(Fig. 151). Results were cross validated against the measured acoustic power delivery of ExAblate 2100 (Insightec, Ltd, Tirat Carmel, Israel). Sonication duration measurements were completed using the hydrophone for measuring the duration of actual sonication deviation from the planned, and the delay after the emergency stop (Fig. 152). Thermometry measured by TTS was validated against measurements of ExAblate 2100. Sonication positioning experiments were completed using of polyacrylamide (PAA) egg white phantom by measuring the position of the scar after grid surface sonication’s. Measurements were completed both using external calliper (Fig. 153) and grey scale DICOM images. Each of these described protocols were repeated by two independent operators for 30 times for repeatability purposes. The Quality Management System ISO13485 has been applied to provide the required documentation for the approval for labelling the new system

**Results**

For safe usage of the system, delivery of acoustic power should be within the tolerance level of efficiency (70 %-90 %). TTS can deliver the acoustic power reliably in a controlled way within this specs (75 % and 80 %) as well as ExAblate 2100 (75 % and 83 %). Actual sonication deviation from the planned sonication duration was maximum -0.17 (Std 0.33) seconds meeting the spec being less than 1 seconds. Maximum sonication stop delay was 0.14 ms (0.16). Measured temperature values by TTS were relatively lower than ExAblate but still efficient enough to provide a scar for coagulation process to measure the deviation. Grey scale images were able to provide better resolution for distance measurement when compared to external calliper, maximum deviation being less than 1 mm. Results show that for static validations TTS successfully meets the criteria for the defined specs

**Conclusions**

Due to regulatory requirements, it is mandatory to provide pre-clinical validation before any tests on animals take place. Designed protocols were able to provide traceability of the input and the output in a controllable way. Repeatability studies provide statistical evidence for the reliability of the static application of TTS software in pre-clinical settings. Based on these results, motion validation protocols are planned as a next step.

## A146 Pre-clinical protocol development for validation of MRgFUS application for liver under breathing motion

### Senay Mihcin^1^, Sven Rothluebbers^2^, Ioannis Karakitsios^3^, Xu Xiao^4^, Jan Strehlow^2^, Daniel Demedts^2^, Ian Cavin^5^, Giora Sat^6^, Tobias Preusser^2,7^, Andreas Melzer^3^

#### ^1^IMSAT, Dundee, UK; ^2^Fraunhofer MEVIS, Bremen, Germany; ^3^University of Dundee, Dundee, UK; ^4^University of Dundee - FUTURA, Dundee, UK; ^5^NHS, Dundee, UK; ^6^GE Medical Systems Israel Ltd., Tirat Carmel, Israel; ^7^Jacobs University Bremen, Bremen, Germany

**Objectives**

The novel Trans-fusimo Treatment System (TTS) (Fraunhofer MEVIS) is designed for the purpose of applying Magnetic Resonance guided Focused Ultrasound (MRgFUS) therapy to ablate liver tumours trhough the rib cage. Application of sonication under breathing motion to a moving target is a very challenging task. TTS is classified as a high-risk software as it controls the Conformal Bone System (CBS) transducer (Exablate 2100, Insightec, Ltd, Tirat Carmel, Israel) for ablation of human tissue as well as the MRI tracking and temperature mapping. For this reason, it requires preclinical validation. The first step in pre-clinical validation of TTS required static validation. Static validation was completed by cross-validating against ExAblate 2100 (Insightec, Ltd, Tirat Carmel, Israel) software. TTS has successfully met the set of specifications for delivering acoustic power, sonication duration, thermometry and sonicating to a planned location by using the designed protocols for validation in pre-clinical settings. The next step after

**Methods**

**Fig. 154 (abstract A146).**
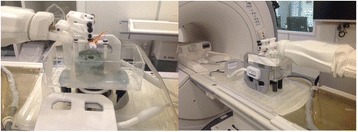
Experiment set up showing the phantom, phantom holder, dual flex MR coils and the INNOMOTION Robotic arm (IBSMM, Prague) to provide 1 Dimentional range of motion

A novel phantom, providing both temperature information and detecting sufficient amount of landmarks for tracking algorithm was developed. For measuring thermometry and observing coagulation procedure, the middle part of the phantom consisted of polyacrylamide (PAA) egg white material surrounded by 2 % agar and samphire to replicate the vein structure of liver. Echo Planar Imaging (EPI) was used during sonication to provide real time thermometry information using 1.5 T GE Scanner. To replicate breathing motion, phantom was moved within 20 -30 mm range by using the INNOMOTION Robotic arm (IBSMM, Prague). A special phantom holder was designed to provide the linear motion with the robotic arm while sonicating using the transducer of CBS. The experiment setup is as shown in Fig. 154. With this designed phantom holder, dual flex MR coils were positioned at a closer distance to each other for better image quality. Sonication was planned for 100 W and 30 seconds to observe the sonication scar. The Quality Management System ISO13485 has been applied to provide the required documentation for the approval for labelling the new system

**Results**

**Fig. 155 (abstract A146).**
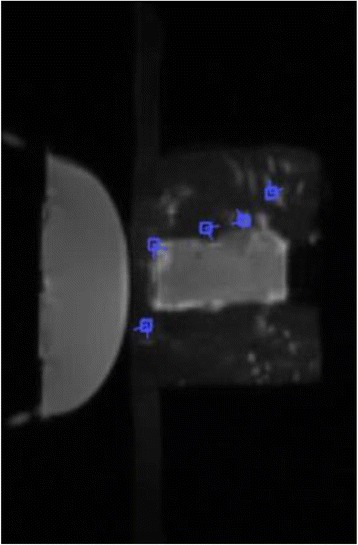
Land marks detected by Transfusimo Treatment Software for tracking for the application of treatment by using the improved planning scan set up under EPI imaging

Initial results show that samphire can replicate the vein structure for tracking algorithm in EPI scanning, without using any contrast agents. TTS can detect the vein structure as landmarks (Fig. 155) and sonicate to a planned location, by leaving a permanent scar to observe coagulation. The distance of coils to each other play important role in pre-clinical validation, due to noise problem caused by air in between the coils, which is not supposed be a problem in clinical settings. Designed phantom holder to place coils at a near location (16 cm in between distance) led into the successful application of tracking algorithm for sonication to a planned location. TTS can monitor the prescribed range of motion provided by the robotic arm during sonication, replicating the breathing motion for abdominal organs

**Conclusions**

Pre-clinical validation is the mandatory step in qualifying a software controller TTS of MRI and the FUS system a class 3 device. Initial results show that TTS can function to track land marks and provide thermometry information in real-time in pre-clinical settings. As a next step, repeatability experiments are planned in more detail to provide statistical evidence and prepare the first in human trial.

## A147 Effect of transducer ground plane configuration on Magnetic Resonance radio-frequency coils and corresponding significance on image SNR during MRgFUS treatments

### Emilee Minalga, Allison Payne, Robb Merrill, Dennis Parker, Rock Hadley

#### University of Utah, Salt Lake City, Utah, USA

**Objectives**

In MRI, the highest signal-to-noise ratio (SNR) is achieved by placing the radio-frequency (RF) coils as close to the region of interest as possible. In MRgFUS treatments, the transducer is often within the volume that would contain the ideal RF coil placement. In addition, if the transducer has a single, continuous ground plane, eddy currents can reduce the SNR in the region of interest. This study compliments the previous work that has been done to reduce gradient induced eddy currents. Using a similar approach this work investigates effects of ground plane eddy currents on RF coils by quantifying the resulting reduction of SNR. Finally, design strategies are outlined that are required to reinstate the diminished SNR.

**Methods**

**Fig. 156 (abstract A147).**
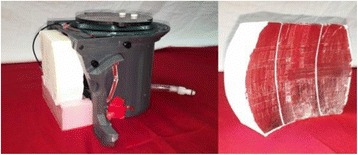
Left image showing imaging setup with mock transducer (white) radio-frequency coils (copper) phantom holder (gray) on the right is a mock transducer with 1x3 ground plane configuration

A rectangular radio-frequency coil (20 cm x 13 cm) was built on a homogeneous cylindrical phantom (12 cm x 11 cm). The transducer ground plane was simulated by placing copper tape on the face of a 3-D printed plastic transducer (aperture: 13 x 10 cm, 10 cm radius of curvature). The ground plane was then broken into multiple copper patch elements by removing different numbers of thin copper strips (1 mm) in the short and long axis directions (Table 6 and Fig. 156). For each ground plane configuration, the RF coil loop was tuned and matched with the ground plane in position at 123 MHz with an insertion loss better than -35 dB. Active and preamp detuning were better than -35 dB and -20 dB, respectively. Using a Siemens 3 T MRI scanner (PrismaFit), SNR measurements were made using standard gradient echo sequences (TR/TE/FA/FOV = 500 ms/10 ms/90°/256 mm, 256 x 256 mm matrix). The homogenous cylindrical phantom was imaged with the single loop using each of the variations of the ground plane. SNR was calculated in the center of the phantom as well as near the transducer face using a sum of squares calculation.

**Results**

**Fig. 157 (abstract A147).**
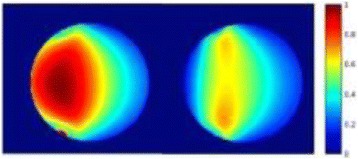
Coronal SNR plots of a homogenous phantom under different transducer ground plane configurations. No transducer present (Left) and a Solid transducer 1x1 ground plane configuration (Right)

**Fig. 158 (abstract A147).**
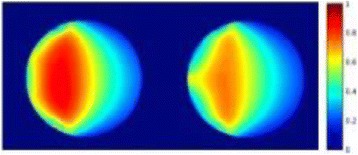
Coronal SNR plots of a homogenous phantom with a 3x3 transducer ground plane configuration (Left) and 1x2 transducer ground plane configuration (Right)

**Fig. 159 (abstract A147):**
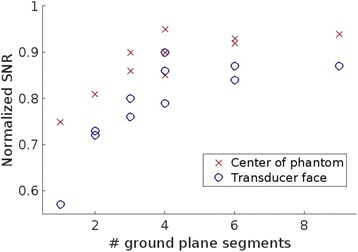
Plot of number of ground plane segments versus the normalized SNR in the phantom (center Xs and Transducer face Os)

SNR results of various ground plane conditions are shown in Figs. 157 and 158. Quantitative SNR values measured in the center of the phantom and at the transducer’s face are plotted in Fig. 159. SNR was reduced by 57 % at the transducer face when comparing the single element ground plane case to the no ground plane case. By segmenting the ground plane, the SNR was nearly recovered (95 %) while still maintaining a ground plane.

**Conclusions**

In general, increasing the segments in the ground plane increased the normalized SNR both at the center of the phantom and at the transducer face, as seen in Fig. 159. By incorporating this design principle into transducer ground plane design, eddy current effects could be reduced allowing for increase image SNR that would positively enhance both temperature and anatomic imaging during MRgFUS treatments.

**Table 6 (abstract A147):** Description of ground plane conditions

*Ground plane element configuration*											
No ground plane	4x1	1x4	3x3	3x2	2x3	3x1	1x3	2x2	2x1	1x2	1x1
*Total number of ground plane segments*											
No segments	4	4	9	6	6	3	3	4	2	2	1

## A148 Improving intercostal HIFU through a novel transducer design: a numerical evaluation

### Pascal Ramaekers, Mario Ries, Chrit Moonen, Martijn de Greef

#### University Medical Center – Utrecht, Utrecht, Netherlands

**Objectives**

Not released for publication

**Methods**

Not released for publication

**Results**

Not released for publication

**Conclusions**

Not released for publication

## A149 High-Intensity Focused Ultrasound ablation in the treatment of heptacellular caricinoma

### Kian Shahriari^1^, Mohammad Hossein Parvizi^1^, Kiana Asadnia^1^, Marzieh Chamanara^1^, Seyed Kamran Kamrava^2^, Hamid Reza Chabok^3^

#### ^1^Islamic Azad University, Science and Research Branch, Tehran, Iran; ^2^Iran University of Medical Sciences, Tehran, Iran; ^3^University of Southern California Los Angeles, Los Angeles, California, USA

**Objectives**

Nonalcoholic Fatty Liver Disease (NAFLD) is a chronic liver disease which is manifested by steatosis, steatohepatitis, cirrhosis, and hepatocellular carcinoma. Patients with Nonalcoholic Steatohepatitis (NASH), which is a subset of NAFLD, have high risks of progressive liver disease and in need of effective treatment.

**Methods**

High-intensity Focused Ultrasound also known as HIFU can be delivered with precision resulting in necrosis of hepatocellular carcinoma (HCC) without a dysfunction liver. HIFU has some important advantages such as non-invasion and non-ionization. The heating effect produced by a focused ultrasound transducer should be checked by using a positioning system. Sonoelastography is a way to quantify fibrosis and it might be useful for predicting liver histology. To obtain more efficient results HIFU Simulation has been used in MATLAB software. In addition, *in vitro* cases has been examined.

**Results**

The results of this simulation indicated that with thermal analysis, according to tissue accoustic parameters in different frequencies, the desired temperature can be achieved for the destruction of cancer tissues and also the destruction of tumors.

**Conclusions**

The aim of this research was to evaluate the safety and efficacy of High-Intensity Focused Ultrasound therapeutic ablation of HCC as primary tumors in liver lesion that is in difficult location s such as main blood vessels, the heart, the gallbladder and bile ducts, or the stomach.

## A150 A real-time data processing framework for steered FUS

### Michael Schwenke^1^, Jan Strehlow^1^, Daniel Demedts^1^, Christine Tanner^2^, Sven Rothluebbers^1^, Tobias Preusser^1,3^

#### ^1^Fraunhofer MEVIS, Bremen, Germany; ^2^ETH Zurich, Computer Vision Laboratory, Zurich, Switzerland; ^3^Jacobs University Bremen, Bremen, Germany

**Objectives**

FUS in moving organs can be realized by updating the focal spot position to follow the target motion (steered FUS). A key feature of such a system is the ability to predict the target motion for the treatment time. Real-time 2D imaging enables capturing some of the organ motion, which is quantified by consecutive tracking of anatomical features in the image stream (observation). All occurring delays need to be compensated for by introducing a spatio-temporal prediction of the potentially unobserved target region and surrounding structures. The final step in the processing pipeline is the update of the FUS control parameter values derived from the prediction (control). A common problem is the tight coupling between observation and control loop.

**Methods**

While the observation rate is restricted by the update frequency of the imaging device, it is desirable to update the FUS focal spot position as often as possible to achieve the best possible approximation of the target trajectory given the hardware constraints.

We propose an approach for decoupling observation and control loop by inserting an asynchronous motion model between the two loops. The motion model is continuously updated using tracking information while an independently running control loop queries the model for target position predictions and controls the FUS device. The system continuously measures the delays caused by all components (imaging, processing, FUS hardware upload) of both loops to calculate the time between the observed target motion and the actual arrival of FUS energy at the target (time of action). A spatio-temporal prediction is then calculated using the motion model for the time of action to compensate the delays.

**Results**

We propose a concept for decoupling observation and control in a steered FUS system. This enables the use of multiple imaging modalities with varying update rates to observe target motion. Further the update rate of the focus position is thereby limited only by the FUS hardware and not by the imaging update rate.

**Conclusions**

Our System may enable a better compensation of the target motion by the steered FUS system. All delays between observation and control are measured continuously by the system and are being compensated for.

## A151 Library sorting for real-time multi-baseline thermometry

### Jan Strehlow^1^, Ruben Stein^1^, Daniel Demedts^1^, Michael Schwenke^1^, Sven Rothluebbers^1^, Tobias Preusser^1,2^

#### ^1^Fraunhofer MEVIS, Bremen, Germany; ^2^Jacobs University Bremen, Bremen, Germany

**Objectives**

MRgFUS is typically monitored by MR thermometry, where temperature differences are calculated from phase differences with respect to a baseline-scan. For periodically moving scenarios, such as FUS in liver treatments, a popular method is the so-called multi-baseline method, where a set of baseline images covering the whole motion cycle (library) is acquired prior to heating. For a given monitoring image, temperature differences are then calculated with respect to the most similar phase-image within the library. Sparse sampling of the motion cycle will thus lead to error in temperature estimation, dense sampling on the other hand leads to another problem: Searching through hundreds of library-images, acquired over several motion cycles, to find the most similar baseline image is computationally expensive and impedes real time applicability. We propose a library sorting method that reorganizes the library-images acquired over several motion cycles to represent a single, but temporally densely sampled motion cycle.

**Methods**

In our method baseline library-images I_i, acquired over several motion cycles, are sorted into a single motion cycle by a three step pipeline: First, a motion cycles extreme position I_e is identified by computing an image similarity (S) between the first library image I_0 and all other images and choosing the most dissimilar I_e = ARGMIN_i (S(I_0,I_i)). In the second pass all library-images are labelled with their similarity to I_e: S_e(i) = S(I_e,I_i). A peak detection is used to find peaks and valleys in S_e. Library-images between a valley and a peak are tagged ascending, images between peak and valley are tagged descending. All ascending and all descending images can then be grouped and sorted according to S_e. Appending the sorted ascending to the sorted descending images yields the sorted library.

With a sorted library the search for the best fitting library image can be narrowed down, e.g. by taking the time between successive monitoring images into account.

**Results**

The proposed method is work-in-progress and currently evaluated on data of several FUS experiments in moving phantoms and an animal experiment.

We expect to considerably lower time to find the most similar baseline image as compared to search over an unsorted library.

**Conclusions**

The method accounts for hysteresis in the motion cycle and may increase robustness of multi baseline thermometry. It may enable the real time use of big libraries that densely sample the motion cycle.

## A152 Training and learning software in the field of Focused Ultrasound therapy of the liver

### Daniel Demedts^1^, Sabrina Haase^1^, Sébastien Muller^2^, Jan Strehlow^1^, Thomas Langø^2^, Tobias Preusser^1,3^

#### ^1^Fraunhofer MEVIS, Bremen, Germany; ^2^Stiftelsen SINTEF, Trondheim, Norway; ^3^Jacobs University Bremen, Bremen, Germany

**Objectives**

Currently, non-invasive treatment using MR guided Focused Ultrasound (FUS) is an emerging technology. Especially in the field of moving abdominal organs there are ongoing developments to compensate for breathing motion while targeting a certain location in a liver tumor. Furthermore, up to our knowledge there is currently no product on the market which could perform such treatments. Within an EU FP7-funded project (TRANS-FUSIMO), a treatment software was developed capable of compensating for breathing motion during FUS. The presented work serves as a possibility to train the usage of the software without being dependent on MR or FUS hardware. In addition, the training and learning software enables the operator to review previously stored cases from a case database which was also implemented in the course of these developments. Furthermore, also trainees can learn from previous procedures to perform safe, efficient and effective treatments in the future.

**Methods**

A modified version of the TRANS-FUSIMO treatment software is presented that acts as training and learning system. Since the TRANS-FUSIMO treatment system has a modular software architecture with respect to several parts like motion compensation, MR image acquisition, and FUS beam steering, components may easily be exchanged without changing neither core functionality nor user interface of the software. For the training and learning system, MR image acquisition and FUS beam steering components were exchanged by emulating software pieces. Images are then loaded from a database instead of being acquired by a real MRI whereas FUS beam steering commands are either just logged or used for a real-time simulation of temperature. This way operators can train to use the software in a safe and effective way. If also trainees will perform such FUS treatments it is important to learn from previous procedures about, i.e., cooling effects by the vessels. A secure case database with encrypted communication has been developed and access to it is possible with the training and learning system. That enables querying of cases which can then be reviewed or trained on. This approach also enables storing data which was not processed with the TRANS-FUSIMO treatment software.

**Results**

A training and learning system is a necessary part when introducing new and complex software tools to be used in clinical studies. It enables the operator to work with the software before treating a patient while using the same interface and functionality like in the real world scenario. To guarantee these requirements, our software implementation is based on the same code as the actual TRANS-FUSIMO treatment software. However, only the connection to the hardware components are replaced by emulated hardware components. In the future, this training and learning system can be extended in such a way that the system itself learns from the previous procedures and provides treatment proposals for the actual cases. However, from the quality assurance point of view, there should be no direct connection between the training and learning system and the TRANS-FUSIMO treatment software.

**Conclusions**

In the course of the TRANS-FUSIMO project a training and learning system was developed to train the clinical personnel performing the anticipated clinical studies using a database of previously performed interventions. The main goal is to provide training and knowledge about the used TRANS-FUSIMO treatment software to ensure a safe, efficient and effective focused ultrasound treatment for the patient.

## A153 Optical flow based motion tracking in the abdomen: Ultrasound vs. MRI; A direct comparison

### Jeremy Tan, Cornel Zachiu, Pascal Ramaekers, Chrit Moonen, Mario Ries

#### University Medical Center – Utrecht, Utrecht, Netherlands

**Objectives**

High-intensity focused ultrasound (HIFU) therapies in the abdomen usually employ motion tracking to ensure that sufficient thermal dose is delivered while minimizing collateral damage. Previous work proposed optical flow based tracking schemes using either ultrasound (US) or magnetic resonance (MR) imaging for this purpose. While MRI has thereby the advantage of superior soft tissue contrast, ultrasound imaging can achieve higher frame-rates. However, HIFU guidance with US penalizes the image quality by the requirement to perform standoff-imaging. This study directly compares the accuracy and precision of motion tracking of real-time MRI and standoff-US imaging using a hybrid imaging setup that can observe with both modalities simultaneously.

**Methods**

Simultaneous US-MR imaging was performed using an MR-compatible 128-element phased-array US imager installed adjacent to the HIFU transducer of a Philips Sonalleve MR-HIFU system. Two data sets were acquired: one on a phantom undergoing a known motion pattern and a second one on the abdomen of a healthy volunteer. Tracking was performed separately on the US and MR images using an optical flow algorithm. The precision and accuracy of the displacements estimated on the US data were then evaluated relative to the injected motion pattern for the phantom dataset and relative to the displacements estimated on the MR data for the *in vivo* case.

US acquisition was performed at 4 MHz with tissue harmonic imaging while the MR Echo Planar Imaging (EPI) sequence employed the following parameters: TE = 9.0 ms, TR = 33 ms, matrix size = 176x176, FA = 25°, voxel size = 2x2x7mm3, EPI-Factor = 13. A total of 500 images were acquired for both phantom and *in vivo* studies. US-MR synchronization was achieved via a TTL pulse generated by the MR-scanner.

**Results**

**Fig. 160 (abstract A153).**
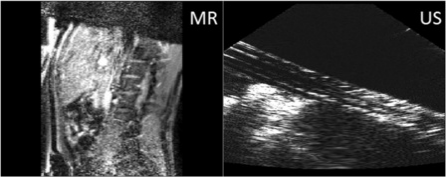
Sagittal images of the liver and the upper intestinal tract of a volunteer with T2*-weighted MRI on the left and tissue harmonic imaging ultrasound on the right

**Fig. 161 (abstract A153).**
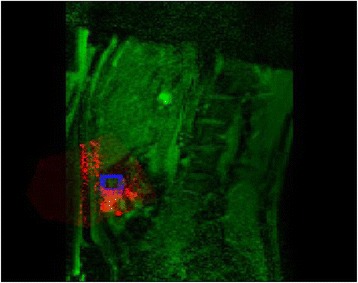
MR (green) and US (red) images overlapped in the same coordinate system. The blue ROI outlines prominent features tracked by the optical flow algorithm

**Fig. 162 (abstract A153).**
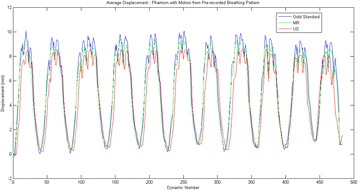
Average displacement of phantom within an ROI, estimated on the MR (green) and US (red) datasets. The blue curve corresponds to the injected motion pattern

**Fig. 163 (abstract A153).**
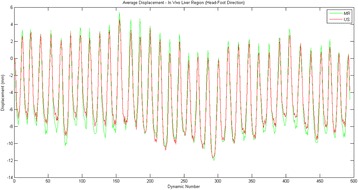
Average displacement, for the healthy volunteer dataset, within an ROI (see blue outline in Fig. 161), estimated on the MR (green) and US (red) images

Figures 160 and 161 respectively illustrate, for the healthy volunteer dataset, an example of raw and overlapped images of both modalities. Precision and accuracy evaluation of the US and MR motion estimates were restricted to a region of interest (ROI), for both the *in vivo* and phantom scenarios. Figure 162 displays the average displacements estimated for the phantom dataset. Relative to the injected motion pattern, an accuracy and precision of 0.71 mm and 0.32 mm respectively were achieved for the MR data, and 1.26 mm and 0.85 mm respectively for the US images. Figure 163 shows the average motion in a ROI defined on the *in vivo* images. Relative to the MR estimates, the accuracy and precision of the US displacements are 1.57 mm and 0.42 mm respectively.

**Conclusions**

This investigation validates the accuracy and precision of optical flow in standoff US imaging. Phantom and *in vivo* experiments show that sub-pixel performance can be achieved (<2 mm). As such, standoff US imaging is a viable alternative to MR tracking and can allow more flexibility in MR protocol design for additional scans or optimization. It can also be a powerful tool for triggering dynamic switching in MR scans. Further work includes optimization of US parameters and real-time applications of this dual modality system.

## A154 Trans-pulmonary FUS ablation of liver using lung flooding, aspects of lung penetration and diaphragm motion

### Frank Wolfram^1^, Daniel Güllmar^2^, Harald Schubert^3^, Thomas G. Lesser^1^

#### ^1^SRH Wald-Klinikum Gera, Germany; ^2^Institute of Diagnostic and Interventional Radiology, Friedrich Schiller University, Jena, Germany; ^3^Institute of Animal Experimentation, Friedrich Schiller University, Jena, Germany

**Objectives**

Several patient studies investigated HIFU treatment of liver tumours. Its location and respiratory induced motion affects the ability to treat. The surrounding lung inhibits extracostal HIFU penetration and respiration induced motion requires compensational guiding and thermometry techniques. It has been shown that One-Lung Flooding (OLF) is safe and originates a superior acoustic path for FUS ablation of lung cancer. FUS treatment in liver could benefit from a direct trans-pulmonary HIFU path and a stable target fixation. Therefore the effects of OLF, enabling a direct transpulmonary HIFU liver ablation and reducing diaphragm motion were investigated.

**Methods**

One-Lung flooding was performed using a porcine model (female, 30-60 kg). Ultrasound guided HIFU was applied transthoracic, transpulmonary into liver on three animals. HIFU exposure (SU102, Sonic Concepts) for 5 sec (3 MHz, 75 W) was sonographical targeted at each animal five times into the upper liver dome. During autopsy, examination of induced ablation zones in liver was performed as well as on lung and diaphragm tissue out of the beam path.

For diaphragm motion, MR imaging on five animals was performed during OLF under mechanical ventilation at the ex-inspirational endpoints. Images based on T2w HASTE (TE84ms/TR900ms) sequence was merged after edge detection and the diaphragmatic deviation in cranial direction was extracted.

**Results**

**Fig. 164 (abstract A154).**
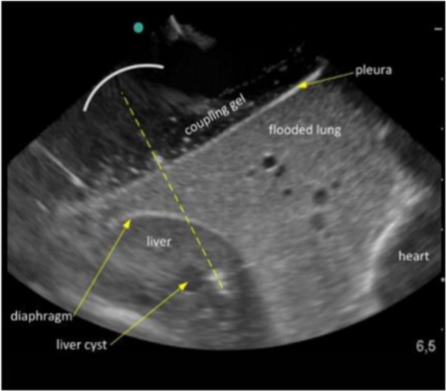
B-mode image after transpulmonary HIFU exposure during OLF showing a strongly hyperechoic lesion within the liver. The yellow dashed line indicates the HIFU beam path, diaphragm and liver cyst

**Fig. 165 (abstract A154).**
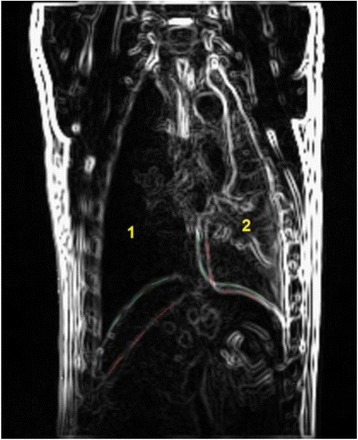
Merged MR images at endex-inspration after edge detection, diaphragm position in ex- (green) and inspiration (red), ventilated (1) and flooded (2) lung

Fifteen of fifteen (100 %) thermal lesions were generated by transpulmonary HIFU sonication in liver. After HIFU exposure the focal zone appeared bright in B mode (Fig. 164). Makroskopically the lesions appeared elliptical with a mean length of 11.3 mm and 3.8 mm width. Histology showed no signs of diaphragm and lung tissue damage. On MRI examinations the largest diaphragm movement (35 mm) was measured in the latero-posterior diaphragm on the ventilated side. The movement wanes towards the flooded side down to 15 mm in the central area. At the lateral edge of the flooded side diaphragmatic movement becomes deterred (Fig. 165).

**Conclusions**

OLF provides a direct transpulmonary acoustic pathway for HIFU ablation in liver. Therefore the therapeutic window for transcostal FUS can be enlarged, which increases the ablation speed and enables treatment of liver tumors in the proximity to lung. Further, the reduced diaphragm and therefore liver motion simplifies targeting strategies. Alternatively pleural effusion has been reported for acoustic coupling of the upper liver dome during FUS treatment. But OLF might comprise several advantages to pleural effusion. So a lower invasivity, a position independend diaphragma location and the mentioned reduced target motion. It needs to be discussed whether the advantages for a transcostal - transpulmonary FUS ablation of liver would justify the increased procedural efforts of OLF.

## A155 Feasibility study of boiling histotripsy for cardiac applications using an MR-guided clinical HIFU system

### Hans-Peter Erasmus^1,3^, Elodie Constanciel Colas^2^, Adam Waspe^3^, Charles Mougenot^4^, Thomas Looi^2^, Glen Van Arsdell^3^, Lee Benson^3^, James Drake^3^

#### ^1^University of Rome – La Sapienza, Rome, Italy; ^2^Centre for Image Guided Innovation and Therapeutic Intervention, Toronto, Ontario, Canada; ^3^Hospital for Sick Children, Toronto, Ontario, Canada; ^4^Philips Healthcare Canada, Toronto, Ontario, Canada

**Objectives**

Hypertrophic Obstructive Cardiomyopathy (HOCM) is characterized by abnormal thickening of the ventricular walls leading to dynamic outflow obstruction. Existing intervention techniques are invasive and present risks, such as complete heart block or arrhythmias due to scarring. Histotripsy is used to perform mechanical fractionation of tissue and has been proven effective in the creation of atrial septum defect on animals. When applied in bulk tissue, the lesions obtained are in a liquid state and can stay inside the lesion to be reabsorbed by the surrounding tissue. Thus, the creation of several, relatively small histotriptic lesions is hypothesized to potentially contribute to the reduction of myocardial volume non-invasively and with minimal scarring and risk of thrombus formation, relieving HOCM.

**Methods**

A boiling histotripsy feasibility study was conducted on *ex vivo* tissue using a clinical MR-guided HIFU system (Sonalleve V1, Philips Healthcare) to determine the optimal sonication parameters and to study the guidance and monitoring capabilities of MRI for this application. Sonications of beef steaks and heart and pig heart were performed at 1.2 MHz using duty cycles (DC) varying from 0.3 % - 2 %, power from 200 W to 850 W and sonication duration from 20s to 70s.

**Results**

**Fig. 166 (abstract A155).**
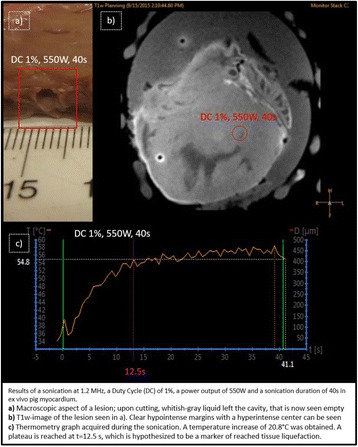
See text for description

The MR-guided HIFU system was effective at delivering shocks sufficient to create lesions of 3 - 5 mm in diameter without causing visible thermal injury (see Fig. 166a). Optimal results were reached at 0.5 - 0.7 % DC, 50 seconds of sonication duration and power output between 550 - 750 W. MRI was proven efficient for guidance and could be used for lesion assessment for DC more than 0.5 % and power output more than 300 W (see Fig. 166b).

**Conclusions**

Boiling histotripsy was proven feasible in *ex vivo* cardiac tissue under MRI guidance and MRI monitoring. Preliminary results also showed that thermometry data could be of value in predicting lesion aspect (see Fig. 166c). Further research is needed to evaluate the feasibility of *in vivo* ablation through transthoracic application of HIFU.

## A156 Proteomic and histological analysis of the rat myocardium following MR-guided pulsed Focused Ultrasound

### Kee W. Jang^1^, Tsang-Wei Tu^1^, Neekita Jikaria^2^, Matthew Nagle^1^, Mary Angstadt^1^, Bobbi Lewis^1^, Farhan Qureshi^1^, Scott Burks^1^, Joseph Frank^1^

#### ^1^National Institutes of Health Clinical Center, Bethesda, Maryland, USA; ^2^National Institutes of Health, Bethesda, Maryland, USA

**Objectives**

Pulsed focused ultrasound (pFUS) has been shown to alter molecular microenvionment of kidney and muscle and has the potential to enhance homing permeability and retention of intravenously (IV) administered mesenchymal stem cells (MSCs) to target tissue. In this study, we examined the mechanotransductive induced proteomic and histological changes following MRI-guided pFUS on rat myocardium.

**Methods**

Female Sprague Dawley rats (8-10 weeks old) were imaged on a 3 T clinical MR scanner (Philips) and T2-weighted MR images were acquired with 8.9 ms repetition and 4.5 ms echo time in 1 mm slice thickness. The images were used as guidance for pFUS to target heart apex (1 MHz; 0 to 8 MPa; 10 ms bursts; 1Hz PRF; RK-100, FUS Instruments). Post-pFUS changes were evaluated *ex vivo* using 7 T MR microimaging system (Bruker) with 7,000 ms TR and 50 ms TE in 250 μm slice thickness followed by fluorescence immunohistochemical (fIHC) analysis of albumin, Evans-blue dye (EBD) extravasation and haematoxylin and eosin (H&E). Proteomic analysis was performed using ELISA kits (Rat Cytokine 24-plex, Bio-Rad, US) and cardiac injury markers (cardiac troponin I (cTnI), pro b-type natriuretic peptide (pro-BNP) and heart fatty acid-binding proteins (hBAFP)).

**Results**

**Fig. 167 (abstract A156).**
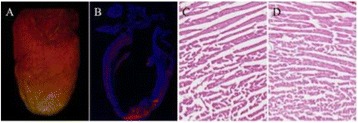
(**a** and **b**) Reconstructed from T2w images demonstrating hyperintensity in the apex of heart that is consistent with edema following pFUS at 8 MPa. Fluorescent image of left ventricle showing EBD extravasation into the apex of left ventricle. (**c** and **d**) H&E stain of treated and SHAM control myocardium showing no difference in muscle morphology

Targeted pFUS resulted in interstitial edema in the myocardium based on 3D reconstructed T2w MRI. Significant EBD extravasation and fIHC staining for rat albumin was observed in the left ventricular apex. H&E staining showed no morphological changes in myocardium in pFUS targeted region (Fig. 167). Temporal proteomic analysis revealed that pFUS treatment significantly elevated pro-inflammatory cytokines consistent with sterile inflammatory response with the levels returning to baseline after 24 hours post pFUS. Cardiac injury markers, cTnI, pro-BNP and hBAFP, demonstrated no significant difference compared to non-pFUS treated SHAM.

**Conclusions**

Noninvasive attempts to the repair of heart diseases as part of a regenerative medicine strategy has been slow. Recent studies suggest that targeted pFUS in combination with IV administered MSCs has the potential to modulate inflammatory response and improve clinical outcomes. In this study, the mechanotransductive effects of pFUS treatment triggered transient increases in pro-inflammatory and anti-inflammatory cytokines (TNF-a, IL1a IL1b, IL10 and IL13) consistent with sterile inflammation without histological damage to myocardium or changes in cardiac injury markers. Histology reveals leakage of plasma albumin and edema into pFUS targeted myocardium in absence of microhemorrhages or muscle damage. pFUS in combination with MSCs maybe used as non-invasive technique for regenerative medicine for delivering stem cells in myocardial diseases.

## A157 A retrospective simulation study of renal denervation using magnetic resonance guided focused ultrasound in a porcine model

### Hailey McLean, Allison Payne

#### University of Utah, Salt Lake City, Utah, USA

**Objectives**

Approximately 30 % of adults in America are affected by hypertension. Because of the established role the sympathetic renal system has in hypertension, renal sympathetic denervation (RSD), disruption of the renal nerves found in the arterial wall and perivascular soft tissue, has emerged as a potential treatment for hypertension. The feasibility of using magnetic resonance guided focused ultrasound (MRgFUS) to non-invasively perform RSD has been demonstrated in both pre-clinical and clinical studies. A porcine animal study was recently done to test the efficacy of MRgFUS in performing RSD. The efficacy of the procedure was determined through assessing kidney medulla norepinephrine in 24 pigs. Using the MRI data obtained during these procedures, 3D models of the treated animals were created and acoustic simulations are underway to correlate the simulated pressure patterns to the experimental efficacy results and determine the effects of phase aberration.

**Methods**

**Fig. 168 (abstract A157).**
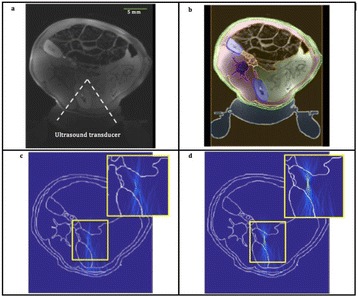
**a** Single axial slice of 3D T1–w VIBE image used for segmentation. **b** Segmentation process using Seg3D. Simulated power deposition (scale bar from 0 to 1.5x107 W/m3) for the model using (**c**) heterogeneous and (**d**) artificially homogeneous acoustic parameters

Segmentation: For each of the 24 animals, a T1-weighted VIBE (T1w Volumetric Interpolated Breath hold Examination) image series is used to perform the segmentation (Fig. 168a). Segmentation is done using Seg3D (Fig. 168b), a volume segmentation and processing tool (Center for Integrative Biomedical Computing at the University of Utah). Twelve tissue types are segmented and a correction algorithm is used to prepare the resulting models for acoustic simulations.

Acoustic Simulations: Each tissue type is assigned an attenuation, speed of sound, and density value consistent with the literature. Using the Hybrid Angular Spectrum (HAS) method, pressure patterns for each experimental sonication treatment location are obtained. The global maximum pressure as well as the maximum pressure achieved within 5 cm of the intended focal point is identified. Additionally, a phase spread metric (PSM) is calculated to quantify the heterogeneity of each model. A PSM value of 0 indicates no phase spread while a value of 1 denotes full phase spread. For comparison, simulations are also performed for a homogenous model where all tissue types are assumed to be uniform (an average value of all 12 tissue types).

**Results**

At the time of submittal, simulations have been completed for 7/24 animals. Initial results demonstrate that phase aberration due to the heterogeneous anatomical structure of the pig decreased the maximum pressure achieved at the focal location by 36 % when comparing the heterogeneous to artificially homogeneous segmented models. Additionally there is an average 10 % increase in the PSM, indicating that the heterogeneity of the segmented models increases the phase spread. The distance between the intended focal point and maximum pressure location was also affected by the heterogeneity of the model. The mean average distance between intended focal point and maximum pressure location was found to be significantly larger (p < 0.001) when comparing the heterogonous (18.4 mm) and artificially homogeneous models (5.6 mm).

**Conclusions**

These initial results indicate it would be beneficial to perform phase aberration correction when performing RSD with MRgFUS in order to increase the accuracy and efficacy of the procedure.

## A158 Pathologic effects of MR-guided boiling histotripsy in various tumor models

### Martijn Hoogenboom, Dylan Eikelenboom, Martijn den Brok, Pieter Wesseling, Arend Heerschap, Jurgen Fütterer, Gosse Adema

#### Radboud University Medical Center, Nijmegen, Netherlands

**Objectives**

Not relesed for publication

**Methods**

Not relesed for publication

**Results**

Not relesed for publication

**Conclusions**

Not relesed for publication

## A159 Multiphase fluid-solid coupled computational analysis of shock-bubble-stone interaction in shockwave lithotripsy

### Kevin Wang^1^, Ying Zhang^2^, Pei Zhong^2^

#### ^1^Virginia Tech, Blacksburg, Virginia, USA; ^2^Duke University, Durham, North Carolina, USA

**Objectives**

Nearly 30 million people in the United States suffer from kidney stones. For these patients, shockwave lithotripsy (SWL; litho-: “stone”, -tripsy: “crushing”) is a first-line treatment, in which extracorporeally generated acoustic waves are focused on the stone to fragment it. Despite the extensive clinical use, SWL is to some extent still a “black box” to the treating urologist: the in-depth mechanisms of stone fragmentation and tissue injury are still unclear; and there is no rational approach to predict treatment outcome. We present a preliminary computational study towards a better understanding of the stone fragmentation process. Specifically, we apply a novel multiphase fluid-solid coupled computational framework to investigate the interaction of a kidney stone immersed in liquid with a lithotripter-generated shock wave and a gas bubble near the stone. The main objective is to elucidate the effects of a bubble in the shock path to stone fracture.

**Methods**

The computational framework used in this work couples a three-dimensional (3D) finite volume two-phase computational fluid dynamics (CFD) solver with a three-dimensional finite element (FE) computational solid dynamics (CSD) solver. The surface of the stone is represented as a dynamic embedded boundary in the CFD solver. The evolution of the bubble surface is captured by solving the levelset equation. The interface conditions at the surfaces of the stone and the bubble are enforced through the construction and solution of local fluid-solid and two-fluid Riemann problems. The computational analysis reported in this presentation are performed on the BlueRidge supercomputer at Virginia Tech, using 200 to 400 processor cores.

**Results**

**Fig. 169 (abstract A159).**
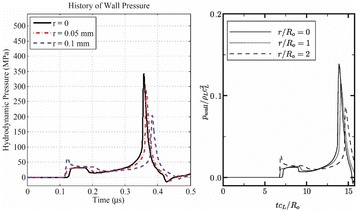
A shock-bubble interaction problem: comparison of the present result (left) and the previous result presented in Johnsen et al. (2008) (right) for the time history of hydrodynamic pressure at three sensor locations

**Fig. 170 (abstract A159).**
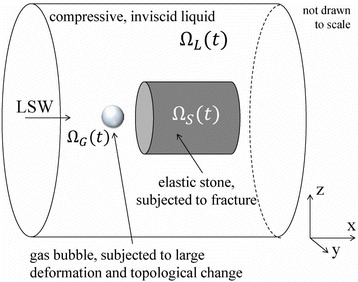
A shock-bubble-stone interaction problem

**Fig. 171 (abstract A159).**
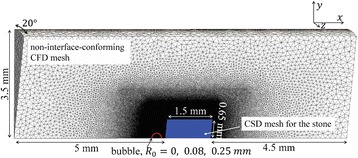
Computational setup of a shock-bubble-stone interaction problem

**Fig. 172 (abstract A159).**
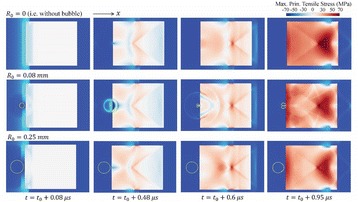
Snapshots of numerical solutions at four time instances. R0 denotes the initial bubble radius. t0 denotes the time instance when the lithotripter-generated shock wave reaches the front surface of the stone

The first step in this study is to verify the computational framework. To this end, we present a 1D multi-material Riemann problem, and two 3D problems on shock-stone and shockbubble interactions. For all the three problems, our numerical solution is in close agreement with the reference (Fig. 169). Next, we present a series of 3D shock-bubble-stone coupled simulations (Figs. 170, 171 and 172).

**Conclusions**

Our computational analysis suggests that the dynamic response of a bubble to an LSW, as well as its impact to the stone, heavily depends on the initial size of the bubble. Bubbles smaller than a certain threshold implode within 1 s after the passage of LSW; whereas larger bubbles do not. For a specific (and realistic) setting, we find this threshold, in terms of bubble radius, to be 0:12 0:02 mm. Moreover, our results suggest that a non-imploding bubble imposes a negative effect on stone fracture, as it shields part of the LSW from the stone. The effect of an imploding bubble is a bit more complex: it may promote fracture on the proximal surface of the stone, yet hinder fracture from stone interior.

## A160 A perfused breathing kidney phantom

### Xu Xiao^1^, Joyce Joy^2^, Helen McLeod^2^, Andreas Melzer^2^

#### ^1^University of Dundee - FUTURA, Dundee, United Kingdom; ^2^University of Dundee, Dundee, United Kingdom

**Objectives**

One of the main challenges during FUS treatment of abdominal organs is organ motion due to respiration. If motion is ignored, the thermal dose spreads over a larger area, leading to insufficient heat for ablative necrosis. The current FUS treatment systems lack compensation of breathing motion. Research requires *ex vivo* moving organ models with appropriate perfusion. The work reports preparation of a ‘perfused, breathing kidney phantom’ using fresh and preserved porcine kidneys.

**Methods**

**Fig. 173 (abstract A160).**
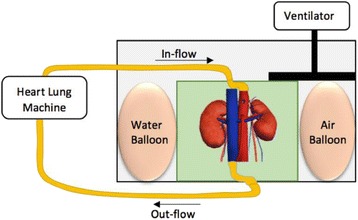
Respiratory perfused kidney schematic

Fresh *ex vivo* porcine kidneys are prepared carefully to remove excess fat and to make the ureters, artery and vein clearly visible. The vessels are then flushed out repeatedly using saline water to remove blood. The process is then continued using ‘Thiel’ preserving fluid and then the prepared tissue is immersed in the preservation fluid for up to one week. This process will leave the tissue re-usable for months and thus making it suitable for repeated sonication and testing. The vessels in the kidneys are then attached to connectors to allow perfusion. The preserved kidneys with the connected connectors are embedded between two layers of agar phantom. The agar block with the embedded kidneys were attached to balloons to mimic breathing motion as seen in Fig. 173. Heart-lung machine is used for perfusion. The inflow from the heart-lung machine is connected to the renal artery and the outflow from the kidney is taken back to the heart-lung machine from the renal vein. Two balloons each side of the phantom are filled with water and air respectively and one of the balloons connected to the ventilator to mimic the breathing motion.

**Results**

**Fig. 174 (abstract A160).**
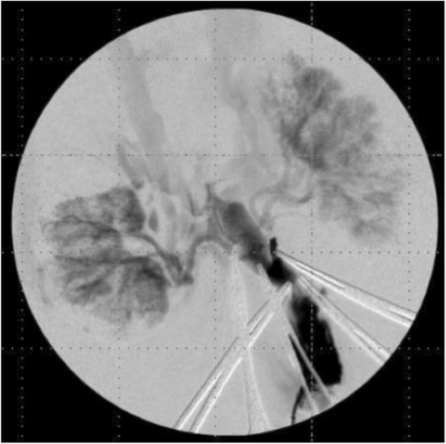
X-ray image of the perfused porcine kidneys

The perfusion of the kidneys was imaged using x-rays (Fig. 174) and it showed very good circulation in the kidneys. The blood flow volumes can be controlled from 100 ~ 300 ml/minutes. The phantom was scanned with real time MRI under respiratory motion. The respiratory motion range along superior-inferior direction could be adjusted within 10 ~ 40 mm. Meantime, the motion speed was adjusted according to different applications as well.

**Conclusions**

A perusable, breathing kidney phantom was prepared using *ex vivo* porcine kidneys. The circulation was images using x-rays and the breathing motion was accomplished using the ventilator. This phantom uses Thiel preserved *ex vivo* kidneys accounting for its increased longevity.

## A161 Breath-hold MR-HIFU hyperthermia: Phantom and in vivo feasibility

### Chenchen Bing^1^, Robert Staruch^2^, Joris Nofiele^1^, Debra Szczepanski^1^, Michelle Wodzak Staruch^3^, Theodore Laetsch^1^, Rajiv Chopra^1^

#### ^1^University of Texas Southwestern Medical Center, Dallas, Texas, USA; ^2^Philips Research, Dallas, Texas, USA; ^3^University of Oklahoma Health Sciences Center, Oklahoma City, Oklahoma, USA

**Objectives**

The use of MR-HIFU to deliver mild hyperthermia (40-45 °C for 10-60 minutes) requires stable MR temperature mapping for long durations. While this can be achieved in the extremities, for targets in the pelvis, abdomen, chest, and neck, respiratory motion causes magnetic field variations that induce unacceptably large temperature mapping errors. Pediatric MRI examinations are routinely performed under general anesthesia with mechanical ventilation, where forced breath holds of 1-2 minutes interrupted by 20-30 seconds of hyperventilation. Our goal is to investigate the use of forced breath holds to deliver mild hyperthermia to pediatric solid tumors. In this study we evaluate the precision of MR thermometry acquired in phantoms near a moving structure and in pig back muscle near the lungs.

**Methods**

Experiments were performed using a 3 T MRI (Ingenia, Philips Healthcare) equipped with a clinical MR-HIFU system (Sonalleve V2, Philips Healthcare). Temperature maps were acquired over 10-20 minutes and optical temperature sensors (Neoptix T1) were embedded to validate MR thermometry.

In phantom studies, breathing motion was simulated using an MRI-compatible motion platform programmed to move an aluminum block along a sinusoidal one-dimensional trajectory. Gating was simulated by halting the motion at the furthest distance away from the phantom.

*In vivo* experiments were performed in ventilated, anesthetized pigs lying with their back muscles above the acoustic window of the MR-HIFU system. The lungs and diaphragm were outside of the imaging field of view, but close enough to trigger the motion artifacts. Sterilized temperature sensors were embedded in the muscle adjacent to the target region. Breathing was controlled at 20-24 breaths per minute. End expiration breath holds of 40-60 seconds were implemented by switching off the ventilator, during which mild hyperthermia with a target temperature of 42 °C was performed in an 18 mm diameter region. A recovery period of 20-40 seconds with HIFU disabled was performed after each breath hold. This cycle continued for 10 minutes hyperthermia and another 10 minutes of cooling.

**Results**

**Fig. 175 (abstract A161).**
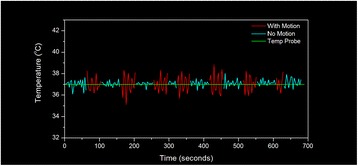
Temperature measured with MR thermometry and optical temperature probe in phantom motion study. The temporal precision is 0.8 °C during motion holds compared to 1.6 °C during free motion

In phantoms next to a moving structure with a large relative susceptibility, MR temperature measurements acquired during a series of motion holds demonstrated a precision of 0.8 °C, compared to measurements acquired while the structure was moving had a precision of 1.6 °C (Fig. 175).

**Fig. 176 (abstract A161).**
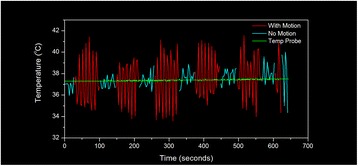
Temperature measured with MR thermometry and optical temperature probe in pig without heating. The temporal precision is 0.9 °C during breath holds compared to 2.0 °C during ventilation

**Fig. 177 (abstract A161).**
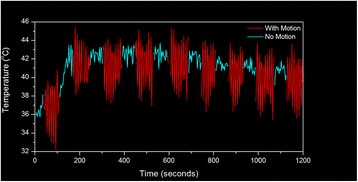
Temperature measured with MR thermometry in pig with HIFU enabled. Heating above 41 °C was achieved within the target region for 11.6 minutes, confirming the feasibility of breath-hold mild hyperthermia treatment

In pig back muscle with no HIFU heating, precision during breath holds was 0.9 °C compared to 2.0 °C during ventilation (Fig. 176). MR-HIFU hyperthermia delivered during intermittent breath holds over a 10 minute duration heated the 18 mm diameter target region above 41 °C for 11.6 minutes, demonstrating the feasibility of breath-hold MR-HIFU hyperthermia (Fig. 177).

**Conclusions**

The use of forced breath holds enables precise control of MR-HIFU hyperthermia in targets near moving structures, and may allow MR-HIFU hyperthermia to be extended to pediatric solid tumors of the pelvis, abdomen, chest, and neck.

## A162 Non-invasive growth plate ablation treatment in rodent models using MR-guided High-Intensity Focused Ultrasound

### Chenchen Bing^1^, Robert Staruch^2^, Pavel Yarmolenko^2^, Haydar Celik^3^, Joris Nofiele^1^, Debra Szczepanski^1^, Peter Kim^4^, Harry Kim^5^, Matthew Lewis^1^, Rajiv Chopra^1^

#### ^1^University of Texas Southwestern Medical Center, Dallas, Texas, USA; ^2^Philips Research, Dallas, Texas, USA; ^3^Sheikh Zayed Institute for Pediatric Surgical Innovation, Washington, DC, USA; ^4^Children’s National Health System, Washington, DC, USA; ^5^Texas Scottish Rite Hospital for Children, Dallas, Texas, USA

**Objectives**

Leg length discrepancy is an orthopaedic problem that usually appears in childhood. Without treatment, the patients will have noticeable limp, back pain and other symptoms. Traditional treatment includes surgically injuring the growth plate to shorten the longer leg. While effective, this procedure is invasive and associated with the risks of surgery. MR-guided high-intensity focused ultrasound (MR-HIFU) could provide a non-invasive approach for growth plate ablation. The hypothesis of this study was that exposure of the growth plate to HIFU would achieve a reduction in limb growth, as an alternative to surgical destruction. To test this hypothesis, we adapted a pre-clinical small animal MR-HIFU system to perform growth plate ablation treatment in rodent models.

**Methods**

Experiment were performed with a 3 T MRI (Ingenia, Philips Healthcare) with a preclinical HIFU system (RK 100, FUS Instruments). Growth plate ablation treatment was performed on young animals in the growth phase of their limbs (Sprague Dawley rats, male, 4-6 weeks old, n = 8). All *in vivo* experiments were approved by UT Southwestern Institutional Animal Care and Use Committee. Anaesthetised animals were stabilized on the HIFU platform, with one side of the thigh facing the acoustic window. The target region was selected as the growth plate of the proximal femur, with the contralateral leg acting as a control. Approximately 3 W of acoustic power was delivered to the bone for 20-30 seconds for the ablation. Temperature was measured with MR thermometry within the target region during the treatment. The animals were allowed to recover and were observed for 8 weeks. Contrast-enhanced MR images were acquired on the day of sacrifice. Femurs on both sides were collected and micro-CT images were obtained to evaluate the treatment effect on bone growth.

**Results**

**Fig. 178 (abstract A162).**
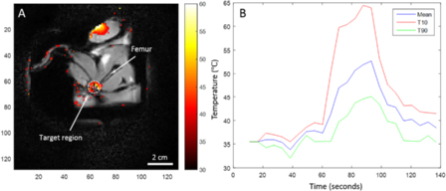
MR thermometry during the treatment and temperature measurement within the target region

**Fig. 179 (abstract A162).**
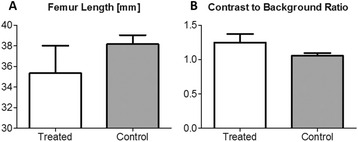
8-weeks post treatment evaluation by contrast-enhanced MR images and micro-CT scans

A localized temperature increase was observed in the targeted region and appeared to cover the entire region of the growth plates (Fig. 178). All animals recovered quickly and did not exhibit any deficits in gait or function during the 8-week observation period. An average length difference of -6.4 ± 6.2 % (p = 0.02, paired t test, Fig. 179a) between treated and control femur was observed from the micro-CT images. Post-treatment MR contrast imaging (n = 8 animals) showed a signal increase of 16.7 ± 12.1 % (p < 0.01, paired t-test, Fig. 179b) in the exposed region, suggesting residual scar tissue in the region of exposure.

**Conclusions**

In this study, we have confirmed that growth plate ablation treatment in rodent models can be achieved with a pre-clinical MR-HIFU system. The procedure was safe and resulted in a reduction in the growth rate of the exposed leg. These results support further investigation of HIFU as a non-invasive treatment for pediatric leg length discrepancy.

## A163 A phantom study of a far field skin protection strategy for MRgFUS in the extremities

### Rutwik Shah, Eugene Ozhinsky, Viola Rieke, Matthew Bucknor

#### University of California San Francisco, San Francisco, California, USA

**Objectives**

One challenge in using magnetic resonance focused ultrasound (MRgFUS) in the extremities is heating of the tissue at the far field skin-air interface where reflection of sound waves occurs. Heating in this region can be difficult to detect by MR thermometry and can potentially result in high grade skin injuries.

In clinical practice, clinicians will sometimes use a water bag or gel pad device coupled to the far-field skin in order to prevent excess heating in this region. The purpose of the current study was to determine the amount of heating prevented in the far field by use of a coupling device, as a function of time to temperature rise and maximum temperature reached.

**Methods**

MRgFUS was used to perform sonications on a tissue mimicking gel phantom (ATS Labs Inc, Bridgport, CT) in two experimental scenarios. In the first scenario, the phantom was sonicated directly without a coupling device attached to the far field of the phantom. In the second scenario, a room temperature water bag was attached to the far field of the phantom.

**Fig. 180 (abstract A163).**
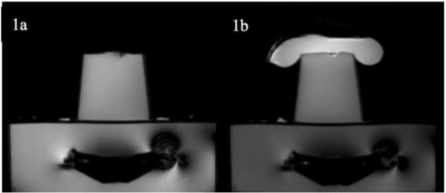
Coronal T2 fast spin echo images demonstrating the two set-ups used during this experiment, one with and one without a waterbag at the far field air interface

In both scenarios, two fiber-optic temperature probes were placed approximately 1 cm below the far field air-phantom interface, 5 cm above and distal to the planned level of sonication, with the tip of both probes at approximately the center of the phantom’s cross-sectional surface area (Fig. 180). The probes provided continuous thermal readings. A mock flat circular target of 5 cm was prescribed 4 cm from the base of the phantom on planning images. A small amount of degassed water was placed at the near field interface in both scenarios.

Each phantom was sonicated a minimum of 15 times, and additional sonications were performed if necessary to produce a 10 °C temperature rise. Maximum temperature rise following each sonication was recorded.

**Results**

**Fig. 181 (abstract A163).**
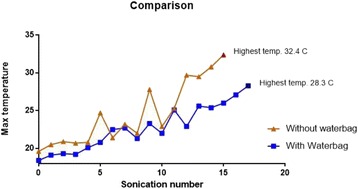
Comparison of temperature curves over time for each experimental set-up

In the first scenario (no water bag coupling device), the temperature began at 19.6 °C and rose to a maximum of 32.4 °C after 15 sonications (Fig. 181). An increase of 10 °C was seen after 12 sonications. Intermittent temperature spikes were also noted, most strikingly after sonications 5, 9, and 12. In the second scenario (with water bag coupling device in the far field) the temperature began at 18.4 °C and rose to a maximum of 26 °C after 15 sonications. Additional sonications were performed and an increase of 10 °C was seen only after 18 sonications, rising to a temperature of 28.4 °C. No temperature spikes were seen in the second scenario with a relatively smooth/gradual increase in temperature noted.

**Conclusions**

This experiment demonstrates that a water bag coupling device in the far field significantly delays the rate of heating and maximum heating in this region during MRgFUS. These results suggest that this type of device can both allow for more complete treatments and help minimize risk of far field skin injury.

## A164 Low Intensity Pulsed Ultrasound (LIPUS) for the treatment of spinal disc degeneration — ultrasound exposimetry system and in vivo implementation in a rat tail model

### Chris Diederich^1^, Vasant Salgaonkar^2^, Peter Jones^1^, Matthew Adams^1^, Arda Ozilgen^1^, Peter Zahos^3^, Dezba Coughlin^1^, Xinyan Tang^1^, Jeff Lotz^1^

#### ^1^University of California San Francisco, San Francisco, California, USA; ^2^University of California San Francisco, Sunnyvale, California, USA; ^3^University of California San Francisco, Napa, California, USA

**Objectives**

Discogenic back pain presents a major health issue, with current therapeutic interventions limited to short term symptom relief without providing regenerative remedies for diseased intervertebral discs (IVD). LIPUS or non-thermal pulsed focused ultrasound (p-FUS) offers a potential treatment option, where ultrasound mechanical energy targeted to a damaged IVD may produce a favorable biological response to modify, stall and even reverse IVD degeneration in a non-invasive manner. LIPUS has been shown clinically to be an effective noninvasive method for the stimulation of bioactivity and favorable tissue remodeling, such as wound and fracture healing. Recent *in vitro* studies have shown that LIPUS stimulation can induce desirable effects within cultured intervertebral disc cells such as an increase in extracellular matrix metabolism, upregulation of proteoglycan synthesis, stimulated collagen synthesis, and induction of anti-inflammatory factors. The objective of our “work in progress” presented herein is to develop a LIPUS exposimetry system specific to targeting degenerated

**Methods**

**Fig. 182 (abstract A164).**
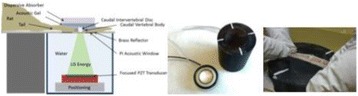
**a** Generalized schema of the exposimetry system for LIPUS delivery at calibrated intensity exposures to damaged rat-tail discs in vivo; **b** focused with integrated housing and mylar membrane to position focus; **c** US energy penetrating tail

**Fig. 183 (abstract A164).**
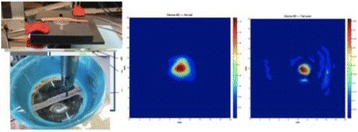
**a** Positioning platform and p-FUS system for disc exposure with sectioned rat tail; **b** hydrophone scanning setup without absorbers; and beam plots of (**c**) no tail and (**d**) tail demonstrating penetration through the IVD as targeted

Ultrasound exposimetry systems were devised and fabricated specific to delivering LIPUS to damaged caudal discs in the rat tail (Fig. 182). The two configurations consisted of 2.5 cm diameter spherically focused PZT4 transducers (1.0 MHz, f = 1; 1.6 MHz, f = 3.8), integrated within a water-filled 3D printed plastic housing with a mylar window w/ cross-hairs, designed as an acoustic standoff to place the focus within the targeted rat tail disc. The applicators/apparatus were evaluated with comparative beam plot measurements with/without insertion of sectioned rat tails *ex vivo* (Fig. 183a, b). *In vivo* studies using the disc damage model are performed following standard procedures approved by UCSF IACUC, with stab incisions applied within 16 Sprague-Dawley rats to generate damage/inflammation in tail discs. Five daily p-FUS exposures (ISPTA120 mW cm-2) are to be applied to stab discs and compared to stab only and normal controls. Histology and microarray gene analysis are performed at 5 days and 28 days after injury, also assessing changes to cartilage, fibrous tissue, and bone.

**Results**

Intensity measurements and radiation force observations with and without the tail sections (Fig. 182c, 183c) demonstrate the focused systems can deliver LIPUS (at 30-50 % of mylar surface intensity) through the skin and connective tissue, to the center of the narrow ~1.5 mm x 5 mm rat tail disc, while reducing exposure to significant portions of the adjacent bone and negligible temperature elevation. The LIPUS exposimetry to damaged rat tail discs, and subsequent histological and microarray analysis are currently ongoing and will be presented.

**Conclusions**

This study demonstrated the technical feasibility of delivering ultrasound isolated to a targeted rat tail disc for studies of LIPUS exposure. In summary, this LIPUS exposimetry apparatus and disc damage model can be implemented for further study *in vivo* to demonstrate potential of ultrasound to increase cellularity, reduce inflammation, and improve remodeling to acute or degenerative disc-related back injury.

## A165 Experimental evaluation of MR-guided HIFU for ablation of sacroiliac joint using a model of human sacrum

### Kathleen Jedruszczuk, Amitabh Gulati, Stephen Solomon, Elena Kaye

#### Memorial Sloan-Kettering Cancer Center, New York, New York, USA

**Objectives**

**Fig. 184 (abstract A165).**
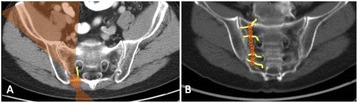
Schematic of approach to MR-HIFU treatment of SIJ. A. Oblique axial view of patient in supine position. HIFU beam (orange) aimed nerves (yellow) at SIJ B. Oblique coronal view of sacrum showing HIFU ablation targets (orange), lateral branch nerves

**Fig. 185 (abstract A165).**
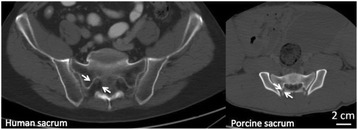
Axial CT images of human and porcine sacrum. White arrows point at the sacral foramen

Chronic low back pain (CLBP) is a leading cause of work disability. In 15-30 % of CLBP patients, the source is the dysfunction of the Sacroiliac Joint (SIJ). [1] One method providing therapeutic effectiveness is radiofrequency ablation (RFA), however this invasive and laborious procedure has seen slow clinical adoption, limiting effective treatments for SIJ pain.[2-3] HIFU could provide a non-invasive alternative by creating a continuous lesion lateral to the sacral foramina (Fig. 184). The main risk of using HIFU for SI joint ablation is damaging adjacent neural structures. A pilot study in swine demonstrated that MR-guided HIFU (MRgHIFU) ablation of SIJ is feasible, and lateral branch nerves can be ablated without damaging adjacent nerve roots. [4] The anatomy of human sacral foramina, however, is not identical to that of swine (Fig. 185). Hence, translating preclinical safety results into human patients may be prone to risk and further evaluation is needed.

**Methods**

Phantom: Images from an adult pelvic CT scan will be used to segment the sacrum bone from adjacent muscle tissue. The segmented model will be manufactured in a 3-D printer using (Acrylonitrile Butadiene Styrene) ABS plastic.[5] The muscle tissue will be mimicked by a high-temperature hydrogel matrix (gellan gum) combined with different sizes of aluminum oxide particles and other chemicals.[6]

Experiment: MR-HIFU experiment will be performed using a clinical HIFU system (InSightec, Ltd., Israel) installed in a 1.5 Tesla MRI scanner (GE Healthcare, USA). HIFU beam will be applied as shown in Fig. 184. Temperature will be monitored using MR thermometry and four fiberoptic temperature sensors (Luxtron, USA) inserted into a foramen.

**Results**

For L5 to S3 vertebral levels, we will determine the range of the acoustic parameters (focal spot size and position) and HIFU beam orientations within which thermal ablation of the SIJ can be achieved without causing thermal damage inside the sacral foramina.

**Conclusions**

Detailed investigation of the safety of HIFU of SIJ using human-sacrum-mimicking phantom will inform future studies aimed at applying MR-HIFU clinically to treat sacroiliac low back pain.

**References**

1. Cohen SP. Sacroiliac joint pain: a review of anatomy, diagnosis, and treatment. Anesthesia & Analgesia. 2005;101(5):1440-53.

2. Yin W, et al. Sensory stimulation-guided sacroiliac joint radiofrequency neurotomy: technique based on neuroanatomy of the dorsal sacral plexus. Spine. 2003;28(20):2419-25.

3. Roberts SL, et al. Cadaveric study of SIJ innervation: implications for diagnostic blocks and radiofrequency ablation. Regional anesthesia and pain medicine. 2014;39(6):456-64.

4. Kaye E., et al. Novel Application of MR-HIFU for ablation of sacroiliac joint in sub-acute swine model. Presented at ISTU 2016, Tel Aviv.

5. Menikou G., et al. MRI compatible head phantom for ultrasound surgery. Ultrasonics. 2015;57:144-152

6. King R., et al. Development and Characterization of a Tissue-Mimicking Material for HIFU. IEEE Trans Ultrason Ferroelectr Freq Control. 2011;58(7):1397-1405.

## A166 A 3D spiral ultrashort echo-time sequence to detect signal level changes in heated bone

### Samuel Fielden^1^, John Mugler^1^, Wilson Miller^1^, Kim Butts Pauly^2^, Craig Meyer^1^

#### ^1^University of Virginia, Charlottesville, Virginia, USA; ^2^Stanford University, Stanford, California, USA

**Objectives**

Currently, there is no feasible way to measure bone heating *in vivo*. Tissue close to bone can be monitored and temperature models can be used to determine when it is safe to apply another burst of ultrasound energy. However, a noninvasive MR-based bone thermometry method would be useful to directly monitor bone heating during FUS treatment.

Because of its short T2/T2*, the popular PRF shift thermometry technique cannot be performed in bone. A method proposed by Miller and further demonstrated by Han uses UTE imaging to detect signal changes in bone caused by changes in the relaxation rates due to heating. Here, we have used a 3D spiral-based UTE sequence to rapidly collect UTE images and detect signal changes in bone as it cools down inside the bore of the scanner. Furthermore, we have used this sequence in a proof-of-concept UTE skull imaging experiment using a human volunteer.

**Methods**

**Fig. 186 (abstract A166).**
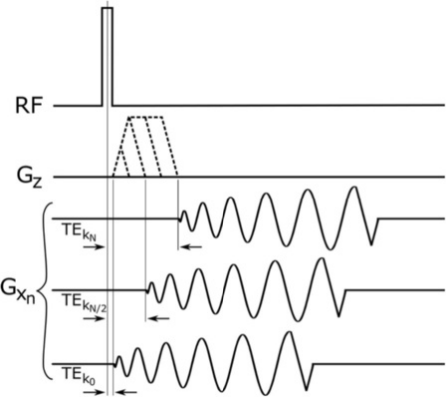
Variable TE 3D spiral pulse sequence diagram. Very short TEs are achievable near the center of k-space, where the through-plane PE gradients are small (or nonexistent)

A 3D spiral UTE sequence was used for rapid UTE imaging. Briefly, a 3D stack-of-spirals acquisition was modified to achieve very short echo times by beginning each spiral readout immediately after the through-plane phase-encoding gradient waveform is complete. This results in a variable TE in the through-plane direction. For the center of k-space where the PE gradients are small (or nonexistent), the minimum TE achievable is approximately 50 μs (Fig. 186).

A room-temperature bone sample was first placed into the scanner and the spiral UTE sequence was used to acquire volumetric images every 75 seconds for 12.6 minutes. Following this acquisition, the bone was placed into a 55 °C water bath for 5 minutes, then imaged again with the same protocol. After imaging, cortical bone was manually segmented and the mean signal intensity recorded at each time point.

To demonstrate the sequence’s potential for human skull imaging, a volunteer was scanned with parameters set to achieve a 67-second acquisition time with an in-plane resolution of 2.5 mm2. A second volumetric image was obtained with a TE of 5.1 ms (to preserve fat/water phase) to provide late-TE comparison images. No heating was performed in this experiment.

**Results**

**Fig. 187 (abstract A166).**
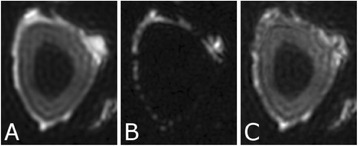
Spiral UTE of bovine tibia. **a** Minimum TE. **b** Late TE. **c**) Subtraction, highlighting cortical bone (and connective tissue surrounding the sample). One slice is shown from the 3D volume. Cortical bone is only detectable in the minimum TE image

**Fig. 188 (abstract A166).**
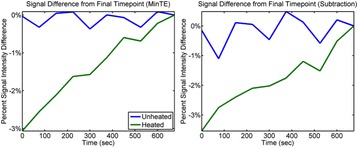
Mean signal difference of cortical bone, referenced from final time point. The signal of the heated sample increases as the bone cools in the bore of the magnet over a 12-minute timespan. The unheated sample shows no signal change

**Fig. 189 (abstract A166).**
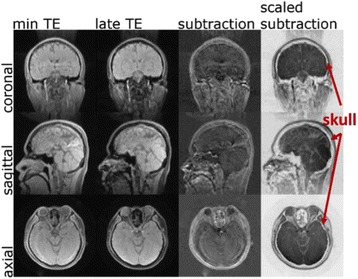
Whole-head UTE images acquired in 67 seconds. While visible in the subtraction image, the skull is better visualized with a scaled subtraction

In Fig. 187, the early and late TE images from the bovine tibia experiment are shown, along with the subtraction image of the two. Cortical bone signal is only visualized with the early TE configuration. Figure 188 shows the mean signal of the heated sample increasing as the bone cools in the bore of the magnet over a 12-minute timespan. In contrast, the sample that remained at room temperature shows no change in signal. Figure 189 shows whole-head spiral UTE images alongside late-echo images to illustrate the difference in contrast achievable with this sequence. Direct subtractions as well as scaled subtractions are shown, highlighting the bone signal. SNR, measured in a region of the frontal bone, is 54 in the minimum-TE image, 23 in the direct-subtraction image, and 43 in the scaled-subtraction image.

**Conclusions**

This work shows the feasibility of temperature monitoring for bone across potentially large fields of view; however, the small phantom size and number of experiments presented here call for further study. Volumetric UTE sequences have typically required scan times of about 10 minutes, which may preclude their use in focused ultrasound procedures; by comparison, the 3D spiral UTE sequence can acquire large volumetric data in about one minute. The ultimate goal is to monitor the entire skull and skull base between therapeutic insonications, which are typically separated by a few minutes.

## A167 A versatile ultrasound system for in vitro experiments on cells

### Gaetano Barbato, Gian Luca Scoarughi, Cristiano Corso, Alessandro Gorgone, Ilaria Giuseppina Migliore

#### Promedica Bioelectronics SRL, Rome, Italy

**Objectives**

One of the most difficult tasks to achieve with available instrumentation used to study the interaction between ultrasound (US) and cellular model systems is to design an experiment, where the effects of only one physical parameter is evaluated, while all the others are kept constant. The set-ups are usually custom made, often use instruments intended for a different purpose. The outcome are results not always well reproducible, and rarely comparable with those obtained with techniques considered standard in molecular and cellular biology. Sterility, as well as temperature, are not well controlled. All these considerations translate into poor reproducibility of US *in vitro* data, leading to standard deviations which are often larger than the values themselves. We present US experiments performed on cell samples with our designed bench-top US apparatus versatile to be adapted for several in vitro experiments and show easy and robust reproducibility and low Standard Deviations data, using standard set-ups for the cell samples.

**Methods**

One main feature of our bench-top US system is that it has been designed in order to minimize interferences and degradation of the acoustic energy using the standard plasticware that are commonly used in molecular biology labs, ensure temperature controls and sterility conditions needed in the field. We present a set-up where the simultaneous use of a set of transducers operating at different frequencies on the same plate, allows the comparison of the deposition of the same acoustic pressure, while evaluating the effect of frequency alone on the readout of the cell experiments. The apparatus modular design allows also to use a set of transducers operating at the same frequency, in experiments where the throughput is a relevant factor. We show that it is possible to define the position of the target within all the achievable areas of the acoustic field with sub-millimetric accuracy.

Tests for several applications based on biologic effects by ultrasound have been carried out by varying the acoustic parameters such as the power, the frequency range, the sonication time and the duty cycle, all controlled within robust protocols executed in automation.

**Results**

We show data that demonstrate that it is possible to perform in vitro experiments for different purpose (i.e. drug or gene delivery via cellular sonoporation, tissue regeneration, neuronal cell stimulation, bacterial growth etc.) keeping constant the relevant physical parameters of sonication, for instance acoustic pressure, but varying one at the time the others parameters (i.e. frequency, pulse length or duty cycle etc).

**Conclusions**

We show that with our apparatus it is possible to obtain robust and reproducible results on cellular experiments using all the standard devices that are commonly available in biological labs. The improvement on the side of reproducibility and portability of the experiments allows a straightforward comparison between our results and those obtained with other techniques.

## A168 Stability of a low-cost thermochromic quality assurance phantom for FUS devices

### Zachary Larrabee^1,2^, Arik Hananel^3^, Matt Eames^1,2^, Jean-Francois Aubry^4^

#### ^1^Focused Ultrasound Foundation, Charlottesville, Virginia, USA; ^2^University of Virginia, Charlottesville, Virginia, USA; ^3^FUS Mobile Inc., Alpharetta, Georgia, USA; ^4^Institut Langevin Ondes et Images, ESPCI ParisTech, CNRS UMR 7587, INSERM U979, Paris, France

**Objectives**

A simple phantom was designed, constructed and tested for daily quality assurance (DQA) of ultrasound therapy transducers. Regular checking of the therapeutic transducer is indeed a key safety test as differences in power output can either result in harm to the patient or ineffective treatment. We introduced recently a DQA phantom which allows the user to determine if the power output of a transducer has changed after calibration through visual inspection. We present here repeated experiments over 6 months on a thermochromic material-based phantom, with two identical transducers, in order to investigate the repeatability and the stability of the DQA phantom. The phantom is made of an attenuating ultrasound absorber (AptFlex F28, Precision Acoustics Ltd, Dorchester, UK) with a surface layer of thermochromic liquid crystals (TLCs) (R35C5B, LCR Hallcrest LLC, Glenview, USA). The TLC changes color when temperature exceeds 35 °C

**Methods**

Two phantoms made from the same sample of AptFlex F28 were tested respectively with two high-power 1.1 MHz transducers (Sonic Concepts, Bothell, USA). A waveform generator (DG1022, Rigol, Beaverton, USA) supplied a 1.1 MHz sinusoid to a power amplifier (100A100, Amplifier Research Corp., Souderton, PA, USA). The amplified signal was monitored by an oscilloscope (TDS2001C, Tektronix, Beaverton, OR, USA) and connected to the input of a Sonic Concepts matching network and ultrasound transducer. Measurements of the time until first TLC material color change were recorded to across a 0.4-11 W range of power in ten sessions spanning over a six-month period.

In addition, the pressure field of the transducers was measured in a water tank (AIMS III, Onda Corp., Sunnyvale, CA, USA) and used as input to a time-domain finite difference simulation of the bio heat equation to simulate the thermal rise in the absorbing material. Simulations were performed with an in house Matlab code (Mathworks, Natwick, USA) operated on a laptop. Table 7 provides material properties used in the simulation. The time required to achieve a color-change temperature of 35 °C is recorded, and the simulation is re-run across electrical intensities ranging between 0.5 and 12 W, based on 85 % efficiency.

**Results**

**Fig. 190 (abstract A168).**
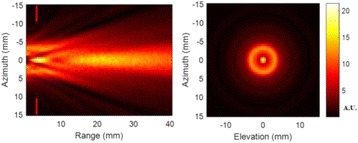
Measured pressure in x-z plane and computed x-y plane pressure

**Fig. 191 (abstract A168).**
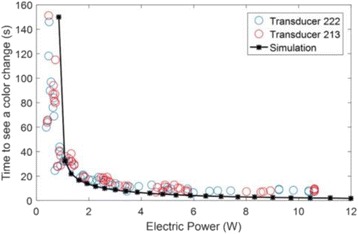
Time required for observable DQA color-change at different driving electrical power levels

Transverse and longitudinal maps of the pressure fields are displayed on Fig. 190. All measurements of time to see a thermal change on both DQA phantoms are plotted in Fig. 191 as a function of electrical power, as well as the corresponding simulated curve. Good agreement is observed between simulation and experimental results. For optimal sensitivity, it is recommended to select an electrical power of 1.5 – 2.0 W. Here, DQA assessments are complete within 20 seconds, and the typical user (with reaction time of 0.5 s) can detect a 10 % deviation in acoustic power output.

**Conclusions**

Long term (6-month) testing of two DQA phantoms on two identical transducers proved to be stable enough to compile all measurements on a single graph (Fig. 190). This system is a cheap and effective way to produce DQA phantoms, both because of the ease of use, and because the use of TLC makes visual changes in the phantom reversible and reproducible over a long period of time. Next steps involve refining the simulation methods used to better validate the DQA design and deploying custom-mounted DQA phantoms to end users and iterate upon the design based on their scenario-specific experiences.

**Table 7 (abstract A168).** Simulation material properties

	Apt-Flex	Coupling Gel	Ceramic	Air
Density (kg/m3)	1010	1050	3800	1.21
Speed of sound (m/s)	1500	1500	9900	330
Attenuation (Np/m)	330	2.5x10-3	2.5x10-3	18.9
Specific heat (J/kg*K)	1800	4200	775	0.001
Thermal diffusivity (mm2/s)	0.091	0.086	0.13	0.21
Thermal conductivity (W/m*K)	0.165	0.38	39	0.026

## A169 Quantification of cavitation histotripsy lesion volumes on a clinical MR-HIFU system

### Avinash Eranki^1^, Navid Farr^2^, Ari Partanen^3^, Karun Sharma^1^, Pavel Yarmolenko^4^, Bradford Wood^2^, Peter Kim^1^

#### ^1^Children’s National Health System, Washington, DC, USA; ^2^National Institutes of Health, Bethesda, Maryland, USA; ^3^Philips, Bethesda, Maryland, USA; ^4^The Sheikh Zayed Institute for Pediatric Surgical Innovation, Washington, DC, USA

**Objectives**

High intensity focused ultrasound (HIFU) is a non-invasive therapeutic technique currently used in the clinic to thermally ablate tissues, However, there exists a likelihood for significant collateral tissue damage and/or incomplete treatment. Alternate HIFU regimes such as cavitation histotripsy (CH) utilize microsecond-long pulses, low duty cycles, and high acoustic pressures to mechanically fractionate tissue. This approach could help disrupt tissues while sparing nearby critical structures from thermal effects and increase the clinical applicability of HIFU. To understand the effect of CH parameters on lesion volumes, we explored a wide range of sonication parameters, such as acoustic power, total sonication time, number of cycles/pulse (pulse width), and pulse repetition frequency (PRF). The goal of this work was to quantify lesion volumes in tissue-mimicking phantoms for various CH sonication parameters.

**Methods**

Sonications were performed on a clinical MR-HIFU system (Sonalleve V1, Philips, Vantaa, Finland) with a 256-element, phased-array transducer (focal length = 12 cm, f-number = 0.94, frequency = 1.2 MHz). Different sonication parameters were tested in polyacrylamide tissue-mimicking gel phantoms, which were placed in a custom 3D-printed holder within a deionized and degassed water bath. A pattern consisting of 27 foci separated by 1 mm, in a 3x3x3 matrix was produced. Sonication parameters were: acoustic power 300 W to 800 W in steps of 100 W, pulse length 200-1200cycles/pulse in steps of 200, total sonication time 270 to 1630 seconds, and PRF 10 to 60Hz in 10Hz increments. Post sonication, 3D ultrasound (X6-1, iU22, Philips, Bothell, WA) was used to obtain the lesion volumes.

**Results**

CH sonications produced varying lesion volumes in the tissue-mimicking phantoms. Increasing the peak acoustic power from 500 to 800 W linearly increased the lesion volumes from 790 to 2780 mm3(R2 = 0.83). On the other hand, 300 W did not produce a visible lesion and 400 W produced a lesion that was visible to the naked eye, but not spatially resolvable using a 6 MHz ultrasound imaging transducer. A linear increase in lesion volume from 600 to 2711 mm3 (R2 = 0.76) was also observed for the tested range of number of cycles/pulse. When increasing total sonication time from 271 to 1630s, lesion volume increased from 1392 to 1500 mm3. Varying PRF from 10 to 60Hz also increased lesion volumes (1688-1802 mm3). Both 0.17 and 0.33 % duty cycle sonications produced small lesion volumes (<0.5 cm3), while sonications at higher duty cycles produced lesion volumes greater than 2 cm3.

**Conclusions**

Cavitation histotripsy was performed on a clinical MR-HIFU system and resulting lesion volumes in tissue-mimicking phantoms were quantified across a broad range of sonication parameters. Results indicate that acoustic power and number of cycles/pulse strongly affect lesion volume, while total sonication time and PRF do not. This study informs future CH work in tissue-mimicking phantoms as well as*ex vivo* and *in vivo* studies. The use of a commercial, clinical HIFU system may speed up clinical translation of histotripsy compared to custom-built, preclinical HIFU systems.

## A170 Tissue-mimicking thermochromic rib phantom for characterization of intercostal HIFU sonications and beam-shaping methods

### Navid Farr^1^, Satya V.V.N. Kothapalli^2^, Avinash Eranki^3^, Ayele Negussie^1^, Emmanuel Wilson^3^, Reza Seifabadi^1^, Peter Kim^3^, Hong Chen^2^, Bradford Wood^1^, Ari Partanen^4^

#### ^1^National Institutes of Health, Bethesda, Maryland, USA; ^2^Washington University in Saint Louis, Saint Louis, Missouri, USA; ^3^Children’s National Health System, Washington, DC, USA; ^4^Philips, Bethesda, Maryland, USA

**Objectives**

High intensity focused ultrasound (HIFU) is a non-invasive therapy modality, offering a unique method of tumor thermoablation. HIFU treatment of hepatic tumors requires intercostal transmission of sufficient energy while minimizing rib and adjacent tissue heating. Assessment and experimental validation of beam-shaping methods and numerical models may aid in clinical translation of HIFU for hepatic tumor therapy. The objective of this study was to develop and produce a tissue-mimicking thermochromic phantom (TMTCP) with embedded rib-mimics to characterize sonications and evaluate beam-shaping methods for transcostal HIFU therapy.

**Methods**

3D-printable ribcage was based on rib renderings from computed tomography. Rib-mimics were manufactured using 3D-printed UV-curing photopolymer with acoustic properties comparable to human ribs. Phantoms were produced by fixing a rib-mimic within a plastic container and then filling it with TMTCP gel. Before HIFU, rib-mimic was visualized on T2-weighted MRI and manually segmented. During sonication, MR images were acquired and temperature was calculated using the PRFS method. Beam-shaping was performed by projecting the ribs onto the transducer surface by ray tracing from the focal point. Transducer elements shadowed by ribs were disabled, while power for active elements was compensated accordingly. Locations 3 cm behind the ribs were targeted with all elements enabled or by using beam-shaping. Temperatures within the targeted region and around rib-mimic were assessed. Phantoms were sonicated by electronic steering (target = 8 mm) at 1.2 MHz frequency and 80 W power for 60s under MRI guidance using a clinical MR-HIFU system (Sonalleve V2, Philips, Finland). Post-HIFU, TMTCP enabled evaluation of temperature distribution within the targeted location and at the rib-mimic. Moreover, acoustic pressure measurements were performed in water by scanning a hydrophone within: 1) focal plane behind rib-mimics, 2) same location with beam-shaping, and 3) plane at the rib-mimic level.

**Results**

TMTCP material with embedded rib mimics was developed and produced. Real-time MRI temperature monitoring at the targeted location and around the rib-mimic was successful. Qualitative and quantitative assessment of temperature was also obtained by phantom color change. Temperatures over the rib-mimics were reduced using beam-shaping, i.e., selectively disabling transducer elements whilst maintaining equivalent acoustic power. Acoustic pressure fields were evaluated using a hydrophone (results pending).

**Conclusions**

TMTCP rib phantom was developed and applied in characterization of intercostal HIFU sonications. The utility of beam-shaping techniques to minimize temperature rise on the ribs while maintaining focus quality during transcostal sonications was demonstrated by monitoring heating patterns. This phantom may be useful in characterization and validation of intercostal HIFU sonication methods and numerical models.

## A171 Multifunctional theranostic contrast agent for ultrasound and photoacoustic image guided tumor therapy with Focused Ultrasound triggered local delivery

### Hyungwon Moon^1^, Jeeun Kang^2^, Changbeom Sim^2^, Jin Ho Chang^2^, Hyuncheol Kim^2^, Hak Jong Lee^1^

#### ^1^Seoul National University Bundang Hospital, Seongnam, Republic of Korea; ^2^Sogang University, Seoul, Republic of Korea

**Objectives**

Not released for publication

**Methods**

Not released for publication

**Results**

Not released for publication

**Conclusions**

Not released for publication

## A172 Low intensity ultrasound and microbubbles increase cellular cisplatin and enhances cisplatin effect in a bladder cancer mimicking culture model

### Noboru Sasaki, Mitsuyoshi Takiguchi

#### Hokkaido University, Sapporo, Hokkaido, Japan

**Objectives**

Multi-modality treatment for muscle invasive bladder cancer aims selective organ preservation with favorable outcome. Cisplatin-based chemotherapy has a survival benefit in combination with radiotherapy. Local delivery of cisplatin may improve local disease control and survival rates. Ultrasound triggered microbubble cavitation enhances chemotherapeutic agents including cisplatin both *in vitro* and *in vivo*. However, it is not fully elucidated whether the enhancement is related to cisplatin concentration in tumor cells. The purpose of this work is to clarify the relation between enhanced cisplatin effect and cellular cisplatin concentration in a 3D culture system.

**Methods**

**Fig. 192 (abstract A172).**
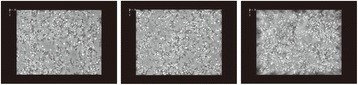
Microscopic images of the 3D model. Bladder cancer cells are embedded into collagen type-I. left, 50 microL; middle 100 microL; right 200 microL

Human urinary bladder cancer cells were mixed with collagen type-I and were seeded into a culture dish at the volume of 50, 100, and 200 microL (Fig. 192). The gel mixture was exposed to non-focused ultrasound (center frequency 1 MHz, duty factor 50 %, intensity 0.9 mW/cm2, time 1 min) in the presence of cisplatin and microbubbles. For platinum measurement, cells were harvested from the gel right after the sonication and were separated from the dissolved gel. Cisplatin concentration was determined by measuring 195Pt with an inductively coupled plasma mass spectrometer. For assessing cell viability, cells were culture for 4 days in the gel after the sonication; thereafter cells were harvested and counted manually.

**Results**

The combination of ultrasound and microbubbles increased the cellular platinum concentration at all tested gel volume. Ultrasound alone did not significantly increase the cellular platinum concentration. Sonication did not increase the platinum concentration of the dissolved gel (i.e. without cells). The platinum concentration of the dissolved gel increased with the gel volume. The enhanced cytotoxic effect was observed at the gel volumes of 50 and 100 microL.

**Conclusions**

This study shows the feasibility of our culture model for assessing the relation of the intracellular delivery and the cytotoxic effect. With our ultrasound parameter, ultrasound-triggered microbubble cavitation has a potential to deliver cisplatin to cancer cells. Meanwhile, the results of this study suggest cisplatin in the extracellular matrix gradually enters cells and shows the cytotoxic effect even without ultrasound. Because cisplatin is a small molecular hydrophilic drug, it rapidly diffuses and may remain in the collagen. The thicker gel retained the higher platinum concentration. Further studies on ultrasound parameter may improve efficiency of the intracellular delivery and contribute to the local delivery of cisplatin in bladder cancer treatment.

## A173 Model predictive control algorithm for large-area regional hyperthermia

### Lukas Sebeke^1^, Xi Luo^2^, Bram de Jager^2^, Maurice Heemels^2^, Edwin Heijman^3^, Holger Grüll^2^

#### ^1^Uniklinik Koeln, Cologne, Germany; ^2^Eindhoven University of Technology, Eindhoven, Netherlands; ^3^Philips Research, Eindhoven, Netherlands

**Objectives**

Hyperthermia has been shown in clinical trials to strongly enhance therapeutic efficacy of radio- and chemotherapy. Main challenge in hyperthermia is to achieve temperatures between ca. 41-43 oC for ca. one hour. While current RF-based hyperthermia devices allow heating of larger volumes, they cannot heat specific subvolumes that are colder due to perfusion. HIFU has been used in preclinical and recently also in a clinical study for hyperthermia applications. The typical approach is to scan the focus point through the tissue using a real-time MRI thermometry feedback for temperature control (MR-HIFU). Here, we are currently developing a new regulatory algorithm based on Model Predictive Control (MPC) for stable HIFU-hyperthermia, which is designed to predict the temperature evolution under the influence of perfusion and diffusion inside the tissue and to plan a power application pattern, which will keep the temperature distribution within a predefined set of parameters.

**Methods**

**Fig. 193 (abstract A173).**
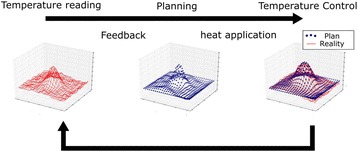
The information of the MR-thermometry image is used as feedback for the control algorithm to plan a heating pattern. The first of these heating steps is applied to control the temperature and a new measurement is taken

The MPC algorithm aims to heat the target tissue to a predefined temperature profile using a heating model derived from the bioheat equation. A system of partial differential equations (PDE) is generated by transferring the heat equation to the Fourier domain. This PDE system is then used by the MPC algorithm to calculate the temperature distribution in the next timestep. The actual MR temperature readout functions as feedback for controlling the heating power (Fig. 193).

**Fig. 194 (abstract A173).**
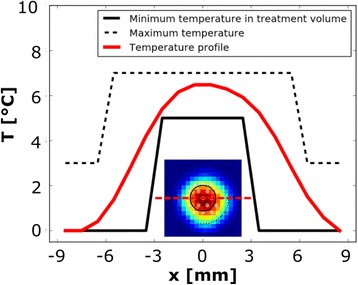
Scheme of Temperature thresholds given to the algorithm (black) and one possible temperature distribution outcome (red). Heatmap inset shows reading line for temperature profile displayed in graph and possible application points

The control objective is to minimize the cost function of the soft constraints imposed on the temperature distributions. Their violation occurs if the modeled temperature doesn’t fit in between the temperature profiles representing the maximum and minimum desired temperatures (Fig. 194). The algorithm was implemented with the Python interface of Gurobi.

To test the model, the predicted heating pattern was compared with MR-thermometry data acquired during sonications performed in a polyacrylamide phantom using the following HIFU-parameters: cell diameter 14 mm, time 45 s and power 30 W. A pixel-wise comparison was made between the acquired sagittal temperature maps and the modeled temperature per time step.

**Results**

**Fig. 195 (abstract A173).**
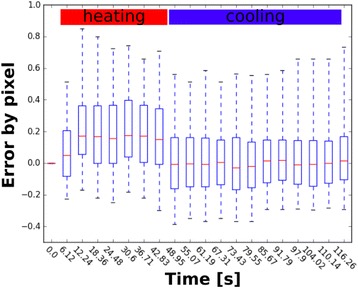
Boxplots of pixel-wise differences between experimental data and the model’s prediction as boxplot versus the measurement time. Whiskers show the 90th percentile

First, the temperature diffusion terms of the PDE system were determined by fitting our model to the cooling curve of a test measurement. The pixel-wise temperature difference between the measurement temperature profile in the phantom and the model’s prediction are shown in Fig. 195 as a boxplot in time, enabling us to see tendencies of over- or underestimation in the predictions. The whiskers were chosen to represent the range in which 90 % of the measurements lie. The upshifted boxes reveal that the model slightly overestimates most of the temperature values for the subsequent temperature distribution during the heating phase. Comparing the reach of the upper and the lower whiskers, we also see that under-estimations mostly stay below 0.4 °C, while over-estimations mostly stay below 0.8 °C. It follows that the algorithm, using the model in its current implementation, will have a tendency to under-treat rather than to cause immediate thermal damage. It also reveals that the algorithm would benefit from a refined mechanism for online-adjustment of the heating estimation.

**Conclusions**

We have implemented the basic functionalities of a MPC-based algorithm for hyperthermia using HIFU. Given a treatment region and a safety margin, it computes a HIFU treatment sequence which meets the minimum required treatment temperature while respecting a given maximum temperature to avoid thermal damage. Using the model in its current implementation, the algorithm will likely have a tendency to under-treat rather than causing thermal damage as temperature estimation errors are more extreme in over-estimations.

We have yet to prove the viability of the algorithm in an experiment which uses the algorithm to steer the transducer. The good agreement between a real temperature development and the used model’s prediction, however, gives us reason to believe that we will be able to present the working prototype of an MPC-based HIFU hyperthermia system in the near future.

## A174 Image-based spatial HIFU transducer calibration for MRgFUS

### Jan Strehlow^1^, Michael Schwenke^1^, Daniel Demedts^1^, Tobias Preusser^1,2^

#### ^1^Fraunhofer MEVIS, Bremen, Germany; ^2^Jacobs University Bremen, Bremen, Germany

**Objectives**

In MRgFUS applications with movable transducers it is a common problem to establish a connection between scanner- and transducer-coordinate systems (spatial calibration). Vendor calibration tools are often only available in proprietary software and not accessible in research applications. Thus, transducer position and orientation are often prescribed in a tedious and error prone manual process. The current work presents an image-based spatial transducer calibration method that transforms a transducer model automatically into a given setup. Our method is based on the idea, that the water systems tubes are typically well visible in MR imaging and attached to one side of the transducer, giving information on the transducers position and orientation. An MR image of the transducer and its periphery can thus be used for an automatic calibration method.

**Methods**

Inputs for the method are an MR image of the transducer in a given setup and a transducer model. A transducer model consists of all relevant transducer information (such as e.g. transducer center, transducer orientation, or relative element positions) and a corresponding MR image. Note that this transducer model has to be established only once and can afterwards be used on any given setup. Both model- and setup-image should contain the transducer and its direct periphery, slice orientation should be roughly parallel to the transducer surface.

The transducer calibration is implemented as two-step process: An initialization is used to coarsely align setup and model image, a subsequent image registration aligns the images precisely. The accumulated transformation can then be used to map from transducer to MR coordinates and vice versa.

The initialization is a series of image registrations that subsequently align transducer to skin coupling, transducer center, and cable orientation. The initial estimate is used as a starting point for an NCC-based, rigid image registration.

**Results**

After setting up a transducer model once, the proposed method can be used to automatically determine transducer position and orientation from a single MR scan within 2-4 seconds. The method was used in a several validation experiments as a suggestion to the user. In most cases the proposed calibration was accepted, only a few times minor adjustments were necessary. We currently evaluate the method on the basis of manual ground-truth and with different transducer models.

**Conclusions**

A hardware- and vendor-independent, image based transducer calibration may be useful in a variety of research applications. Our method was implemented with publicly available modules in MeVisLab and can be provided for research purposes.

## A175 Functionalised human Thiel embalmed cadavers – a clinical model for focused ultrasound development & testing

### Helen McLeod

#### Univerisity of Dundee, Dundee, United Kingdom

**Objectives**

Human Thiel embalmed cadavers have a number of advantages over traditional formalin embalmed cadavers. Properties such as flexibility, tissue tone and a patent vascular system provide the basis for an anatomically accurate model. Researchers at University of Dundee have developed techniques to provide extracorporeal perfusion to the cadaver vascular system, facilitating multimodal imaging techniques such as CT, Xray, Ultrasound and MR. Model complexity is further improved by the addition of respiratory motion, produced by mechanical ventilation. Applications for perfused cadaveric models in the development and validation of Focused Ultrasound Technologies have been demonstrated.

**Methods**

Input and output (if required) connections are placed into the cadaveric target vessels and secured. Connections are made to a heart lung bypass machine to provide extracorporeal flow into the vessels, with flow conditions input to mimic the native flow expected. The fluid selected during perfusion is dependent upon the imaging modality, for example vascular ultrasound requires contrast therefore, a specific blood mimic solution is used. X ray, CT and MRI can successfully use a variety of fluids, with contrast being delivered to target vessel as bolus dose, mimicking clinical practice. Respiratory motion is created by intubating the cadaver with ET tube and ventilating with a mechanical ventilator.

**Results**

Exemplar images from all imaging modalities demonstrate the application of multi modal imaging techniques within Thiel embalmed human cadavers. The perfused cadaveric model has been used within the preclinical development and validation of Focused Ultrasound therapies targeting liver and kidneys.

**Conclusions**

The perfused Thiel embalmed human cadaver can be imaged in multiple modalities, CT, fluoroscopy, angiography, MRI and Ultrasound. It offers a robust and versatile model, ideal for development and evaluation of advanced technologies such as focused ultrasound therapy in moving organs.

## A176 Magnetic resonance guided high intensity foccused ultrasound ergonomic animal experiment platform

### Christopher Abraham, Samuel Pichardo, Laura Curiel

#### Thunder Bay Regional Research Institute, Thunder Bay, Ontario, Canada

**Objectives**

Magnetic-Resonance Guided High Intensity Focused Ultrasound (MR-HIFU) is a minimally-invasive treatment modality that is gaining clinical use. It is used to non-invasively ablate tissue or disrupt the Blood-Brain Barrier. Translation of this technique can be accelerated by facilitating animal experiments, and the development of ergonomic platforms can achieve this purpose. MR-HIFU exposure is performed under water requiring unique engineering solutions to waterproof and tune RF coils. Custom radiofrequency (RF) coils are necessary to obtain optimal signal-to-noise ratio (SNR) in this setting. Without careful tuning and matching of RF coils, targeting, temperature monitoring and thermal dose assessment may not be accurate enough.

The purpose of this study is to provide an engineering solution to treat mice using MR-HIFU using custom RF coils submerged in water with sufficient SNR that allowed for accurate thermal mapping.

**Methods**

An MR-HIFU animal treatment system was designed so that:

- SNR was increased compared to commercial coils
- Tissues were acoustically coupled while the animal remained appropriately anesthetized and warm
- HIFU beam could pass-through window to target
- The animal could be placed for treatment with good reproducibility

The developed system was compared to the Small Flex Coil (Philips, USA). An *ex vivo* mouse was imaged to compare systems. Two parameters were used to determine quality of new system: SNR of targeting images and temperature stability from thermal mapping. The system was tested at two different locations, TBRHSC (Thunder Bay, Ontario, Canada) and Sickkids (Toronto, Ontario, Canada) to confirm reproducibility.

**Results**

An ergonomic treatment bed was designed with a waterproofed coil embedded. After first tuning, RF coils experienced significant loading from the surrounding water, with a frequency shift as high as 7.3 MHz when submerged in water. A tunable matching circuit was added on the circuit to achieve further fine tuning.

**Fig. 196 (abstract A176).**
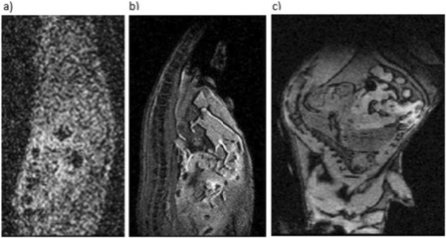
**a** Image of mouse with Philips Small Flex Coil. **b** Image of mouse with custom RF coil. Images performed on a 3 T Achieva system with identical parameters. **c** Custom RF coil tested at SickKids (Toronto, Ontario, Canada)

Figure 196 shows a comparison between the Flex coil and the developed coil. SNR of the in-house developed coil was 28 and 23.5 compared to an SNR of 6 from the Small Flex coil during TBRHSC and SickKids tests respectively. (Gradient Echo, TE = 4 ms, TR = 8 ms, FOV = 50 mm x 50 mm, 2.5 mm slice thickness NEX = 4, FA = 10o.

**Fig. 197 (abstract A176).**
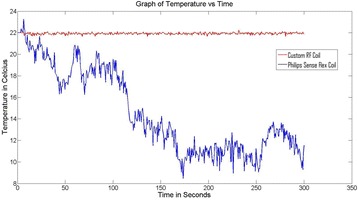
Thermal Calculation of Custom RF Coil (*red*) and Philips Small Flex Coil (*blue*). Temperature calculation are based on the proton frequency shift method and averaged over a region of interest for 300 seconds. Reference temperature was set at 22 °C

Figure 197 shows a 1D thermal calculation over time for both coils. The custom coil showed a stable temperature where the Flex coil recorded a decrease over time for the temperature.

**Fig. 198 (abstract A176).**
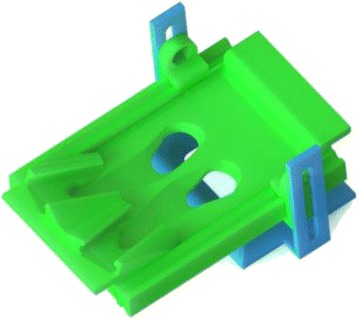
Device used to ergonomically hold mice for focused ultrasound exposure

Figure 198 shows the device used to ergonomically hold mice for focused ultrasound exposure.

**Conclusions**

We successfully designed an ergonomic treatment bed designed for HIFU exposure that can be used underwater while providing sufficient SNR for targeting and thermal mapping. This treatment bed will further facilitate research for focused ultrasound small animal experiments.

## A177 Numerical and experimental validation of a novel therapeutic phased array transducer aimed at sparing of the near-field: the Voronoi-tessellated Fermat’s Spiral

### Pascal Ramaekers^1^, Martijn de Greef^1^, Rémi Berriet^2^, Chrit Moonen^1^, Mario Ries^1^

#### ^1^University Medical Center – Utrecht, Utrecht, Netherlands; ^2^IMASONIC SAS, Voray sur l’Ognon, France

**Objectives**

Not released for publication

**Methods**

Not released for publication

**Results**

Not released for publication

**Conclusions**

Not released for publication

## A178 A unique tissue preparation for characterizing uterine fibroid tissue properties for MRgFUS thermal therapies

### Christopher Dillon, Margit Janát-Amsbury, Allison Payne

#### University of Utah, Salt Lake City, Utah, USA

**Objectives**

Magnetic resonance-guided focused ultrasound (MRgFUS) has repeatedly been demonstrated as a safe, non-invasive therapy for uterine fibroids. However, the treatment response of fibroids with high T2-weighted signal intensity (T2wSI) on pretreatment MRI is poor. While blood flow and cellularity have been hypothesized to play a role, there is a lack of research investigating the cause of poor outcomes in high T2wSI fibroids. This abstract presents an innovative excised, perfused human uterine fibroid model designed to address this clinical challenge. Implementation of this model will allow for characterization of the MR, physiological, thermal, and acoustic properties of uterine fibroids and clarify the reason for variable efficacy in MRgFUS fibroid treatments.

**Methods**

In this study, women undergoing hysterectomy due to symptomatic fibroids receive a preoperative MRI (Siemens 3 T Trio). Immediately following surgical removal, both uterine arteries are cannulated, and the specimen is perfused and immersed in a Modified Krebs Henseleit Buffer maintained at a temperature of 37 °C. Tissue pH, temperature, and interstitial pressure are monitored throughout. This setup keeps tissue viable up to 8 hours post surgery, during which time, the preoperative MRI protocol is repeated on the *ex vivo* uterus. Comparing *in vivo* and *ex vivo* images reveals how/if MR tissue properties and organ perfusion have changed. Additional property measurements include thermal diffusivity and conductivity, acoustic properties of density, speed of sound, and attenuation, and histological examination of vessel density, cellularity, and fibrosis.

**Results**

**Fig. 199 (abstract A178).**
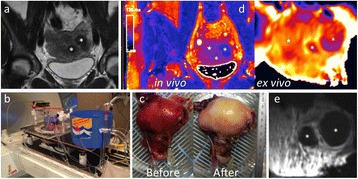
**a** In vivo MRI of uterus with hypointense T2wSI fibroids (asterisks). Ex vivo perfusion model. **c** Photos of Fig. 199. the excised uterus before and after perfusion. (**d**) MR T2 maps: fibroids are hypointense relative to myometrium (star). (**e**) Ex vivo TWIST MRI

Figure 199a shows an *in vivo* coronal MRI identifying two fibroids with hypointense T2wSI. The *ex vivo* model (Fig. 199b) includes heated water baths and fiberoptic probes for temperature control, perfusion-line manometers, and bubble traps; it is MR-compatible and integrated with the MRgFUS system. Photos of the excised uterus before and after perfusion (Fig. 199c) demonstrate effective clearing of blood. While the fibroids’ T2 values increase slightly *ex vivo* (Fig. 199d, TrueFISP MRI), they remain hypointense relative to the myometrium.

Figure 199e shows an *ex vivo* T1 TWIST MRI where contrast agent does not extravasate into the fibroids, indicating the low vascularity of these fibroids. Thermal diffusivity and thermal conductivity of the fibroid tissue were 0.173 mm2/s and 0.431 W/m/°C, respectively. Participant recruitment and data collection are ongoing.

**Conclusions**

This unique setup for the characterization of fibroid properties has great potential for explaining the variable treatment response of fibroids, for improving MRgFUS planning, monitoring, and control, and for identifying the patient population most likely to benefit from MRgFUS therapy.

## A179 Acoustically transparent surface coil arrays for MR guided HIFU

### Joseph Corea^1^, Patrick Peiyong Ye^1^, Ana Clauda Arias^1^, Kim Butts Pauly^2^, Micheal Lustig^1^

#### ^1^University of California Berkeley, Berkeley, California, USA; ^2^Stanford University, Stanford, California, USA

**Objectives**

**Fig. 200 (abstract A179).**
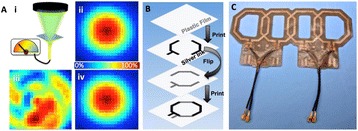
**a**) a(i) Acoustic energy measured at the focal point with (ii) no obstruction, (iii) typical tuning capacitor and conductor, and (iv) thin printed tuning capacitor. B) Fabrication of printed coil C) Photograph of acoustically transparent array

In MR imaging, a surface coil array is most sensitive to tissue close to it. So for MR guided HIFU, placing the array between the transducer and the patient gives the highest signal-to-noise ratio (SNR), and in turn the best quality images to track the heating. During therapy, the transducer is moved in the water bath, which would pass acoustic energy directly through different parts of an array between patient and transducer. Ultrasonic energy easily scatters and attenuates in the materials commonly used in array construction (Fig. 200a). As a result, lower SNR body or non-fitting arrays are used during therapy. This limits the SNR for accurate temperature estimation and resolution compared to specifically designed surface arrays. We address this by creating a surface array using ultra-flexible low attenuation materials that allow it to be placed in the beam path of an ultrasonic transducer with negligible attenuation and signal distortion.

**Methods**

We used additive manufacturing techniques to print very thin receive coils making our array nearly transparent to the acoustic energies common in HIFU (~650Khz-1Mhz). In printing, coils are fabricated layer-by-layer creating the conductors and capacitors needed for tuning (Fig. 200b).

**Fig. 201 (abstract A179).**
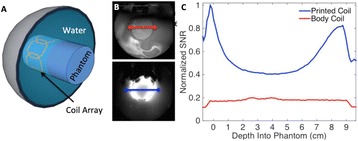
A) Printed array wrapped around gel phantom submerged in water inside head transducer. B) Scan of phantom with (i) body coil and (ii) 4-channel array. Lines highlight where SNR was calculated. C) SNR comparison of printed array and body coil

A water resistant 4-channel array was fabricated using 75 μm of PEEK (polyether ether ketone) film encapsulated in 75 μm polytetrafluoroethylene film with 20 μm Dupont 5064H conductive ink (Fig. 200c). The SNR of our array was compared to the SNR of the body coil of a 3 T scanner on a gel phantom inside the head transducer (Fig. 201).

**Fig. 202 (abstract A179).**
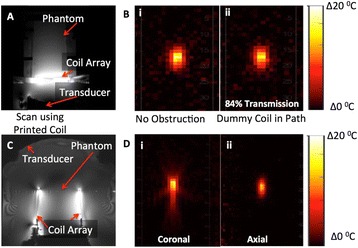
**a** Scan of phantom on a fibroid transducer. **b** Heating point inside of phantom (i) without and (ii) with the array present. **c**) Phantom inside head transducer D. (i) Coronal and (ii) axial scans of heating point in phantom tracked with array

**Fig. 203. (abstract A179).**
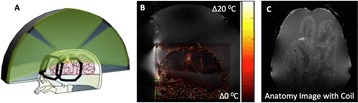
**a** Location of array wrapped around 3D printed skull containing bovine brain/gel submerged in water in head transducer. **b** Sagittal of brain phantom showing heating tracked with array. **c** Axial scan of skull and brain phantom from 4-channel array

To characterize acoustic attenuation, an area inside a gel phantom was heated with an in-table system (Insightec ExAblate 2100) with and without the array present (Fig. 203a and b). Temperature increase was tracked with the body coil of a 3 T MR scanner (General Electric) in both cases. For beam distortion characterization, a spot was heated in a phantom in a brain system (InSightec Exablate 4000) and tracked with the 4-channel array (Fig. 202c and d).

For system proof-of-concept, our array tracked the heating of a bovine brain immersed in gel inside a 3D printed human skull with 200 W of acoustic power (Fig. 203).

**Results**

The signal-to-noise ratio of our array was found to be twice as high as the body coil in the center of the image where heating occurred. Furthermore it showed up to 5 times the SNR at the surface of the phantom (Fig. 201). The array displayed 84 % transmission of acoustic energy and no measureable beam distortion, allowing it to be arbitrarily placed in front of the transducer (Fig. 202).

The 4-channel array was easily able to track the heating inside the skull phantom, imparting no significant attenuation or distortion (Fig. 203).

**Conclusions**

Utilizing the materials and processes described here it is possible to create high SNR arrays that minimally interact with heating in MR guided HIFU, giving clinicians excellent images to guide therapy.

## A180 Multi-echo pseudo-golden angle stack of stars thermometry with high spatial and temporal resolution

### Bryant Svedin, Allison Payne, Dennis Parker

#### University of Utah, Salt Lake City, Utah, USA

**Objectives**

Radial acquisitions offer several unique advantages for proton resonance frequency (PRF) shift thermometry for guiding focused ultrasound therapy in the breast. Frequently sampling the k-space center provides motion robust images, as well as the ability to correct for respiration induced off resonance.^1^ It has been shown that arbitrary high spatial and temporal resolution can be achieved in dynamic MRI by acquiring successive radial spokes separated by the golden angle and applying a sliding k-space weighted image contrast (KWIC) filter,^2^ to the reconstruction.^3^ This work investigates a pseudo-golden angle 3D multi-echo stack of stars acquisition to simultaneously measure PRF shift temperature, T2* and ρ (signal magnitude at TE = 0), correct respiration induced off resonance, and provide water/fat separation with high spatial and temporal resolution.

**Methods**

A 3D multi-echo stack of stars spoiled gradient echo (GRE) sequence was modified to use a pseudo-golden angle increment to repeat the k-space trajectory after 377 views, ~137.56°. Experiments were performed in a normal breast volunteer on a Siemens 3 T Trio scanner to assess the effectiveness of this sequence and reconstruction technique in coronal and sagittal slabs (1.3x1.3x3mm, FOV = 166 mm, Matrix Size = 128x128x8, Flip Angle = 10°, TR = 20 ms, 13 Echoes, TE’s = 2.46/3.69/4.92/6.15/7.38/8.61/8.84/11.07/12.3/13.53/14.76/15.99/17.22 ms). Using the same imaging parameters and Ambu bag movement to simulate respiration, a pork phantom was heated with FUS. Reconstruction: After accurately locating the k-space center,^4^ the slope of the phase with TE at the k-space center was used for respiration correction.^1^ Data was then reconstructed using a sliding filter with 13 lines in the innermost ring (1.56 s temporal resolution), with each successive ring using enough lines to meet the Nyquist criteria. The KWIC filtered k-space distribution repeats after 29 reconstructed time points. Echo phase was combined using a weighted least squares estimate. PRF temperature was also calculated using the combined echo phase. T2*/ρ maps were calculated using linear regression of the log of the magnitude images along the echo dimension.

**Results**

**Fig. 204 (abstract A180).**
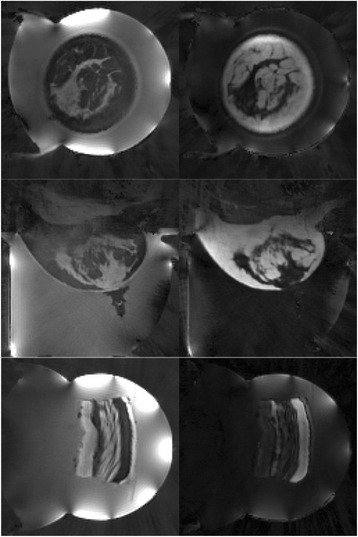
Three point Dixon images produced by the sequence and reconstruction. Top: Coronal breast. Middle: Sagittal breast. Bottom: Pork phantom

**Fig. 205 (abstract A180).**
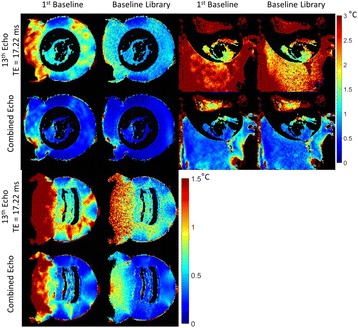
Standard deviation of temperature through time maps for Top: Coronal and sagittal breast. Bottom: Pork phantom. Maps are derived for four cases. Using just the phase information from the 13th echo or from the combined echo phase, and using the 1st time point as the reference phase or a sampling pattern based library of phase references

**Fig. 206 (abstract A180).**
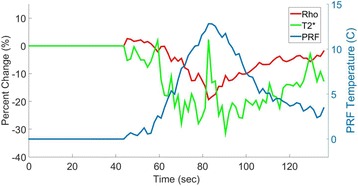
Percent change in ρ (rho - red) and T2* (green) and calculated PRF temperature change (blue) for the hottest voxel during heating in tissue with FUS in the pork phantom. A sampling pattern based library was used for all 3

Figure 204 shows the water and fat images produced from the sequence using the three point Dixon method. Figure 205 shows the standard deviation through time images of the PRF temperature measurements for four cases: PRF temperatures were calculated using both the first time point and the time point with the same k-space sampling pattern as the reference phase for the 13th echo phase and the combined echo phase. Table 8 lists the spatially averaged precision of all tissue voxels in the breast and pork for the four cases. Figure 206 shows the PRF temperature, and percent change in ρ and T2* values *vs*. time of the hottest voxel while heating with FUS in pork.

**Conclusions**

All measurements display a structured artifact based on the k-space sampling pattern used for reconstruction. For this reason, a pseudo and not pure golden angle increment was chosen to cause the artifact to repeat and thus be removable in temperature difference measurements. The method shown here provides promising results for this sequence and reconstruction method for use in free breathing interventional treatments. The respiration correction also corrects main field drift.^1^ The high bandwidth multi-echo readout offers several advantages as it removes the need for fat saturation, provides fat/water images, and T2*/ρ measurements which could be used as another possible measure of temperature change, especially in adipose tissues which do not exhibit a PRF shift with temperature.^5^ Echo combination significantly improves PRF temperature precision.

**References**

1. Svedin BT, et al. MRM. 2015; doi:10.1002/mrm.25860.

2. Song HK, et al. MRM, 2000;44(6), 825-832.

3. Winkelmann S, et al. IEEE Trans Med Imaging, 2007;26(1), 68-76.

4. Block KT, Uecker M. Simple Method for Adaptive GradientDelay Compensation in Radial MRI. Proceedings of ISMRM, Montreal, 2011. Abstract # 2816.

5. Baron P, Ries M, Deckers R, de Greef M, et al. In vivo T2 -based MR thermometry in adipose tissue layers for high-intensity focused ultrasound near-field monitoring. Magn Reson Med, 2014;72(4), 1057-1064.

**Table 8 (abstract A180).** Spatially Averaged PRF Standard Deviation °C

		1st Image Phase Reference	Library Phase Reference
Breast Coronal	13th Echo Phase, TE = 17.22 ms	1.18	0.90
	Combined Echo Phase	0.56	0.44
Breast Sagittal	13th Echo Phase, TE = 17.22 ms	1.9	1.86
	Combined Echo Phase	0.71	0.81
Pork Phantom	13th Echo Phase, TE = 17.22 ms	0.69	0.63
	Combined Echo Phase	0.41	0.24

